# High-pulse-energy integrated mode-locked laser using a Mamyshev oscillator

**DOI:** 10.1038/s41586-026-10517-4

**Published:** 2026-06-03

**Authors:** Zheru Qiu, Xuan Yang, Xurong Li, Jianqi Hu, Zhongshu Liu, Yichi Zhang, Xinru Ji, Jiale Sun, Grigory Lihachev, Zihan Li, Ulrich Kentsch, Tobias J. Kippenberg

**Affiliations:** 1 Institute of Physics, Swiss Federal Institute of Technology Lausanne (EPFL), Lausanne, Switzerland; 2 Institute of Electrical and Microengineering, Swiss Federal Institute of Technology Lausanne (EPFL), Lausanne, Switzerland; 3 Helmholtz-Zentrum Dresden-Rossendorf (HZDR), Dresden, Germany; 4 Present Address: EDWATEC SA, EPFL Innovation Park, Lausanne, Switzerland

**Keywords:** Optics and photonics, Electrical and electronic engineering, Lasers, LEDs and light sources, Integrated optics

## Abstract

Ultrafast lasers have led to numerous advances across science and technology: they enabled corneal surgery^1^, revealed chemical reaction dynamics^2^ and triggered the development of optical atomic clocks^3^. Over the past decades, extensive efforts have aimed to realize mode-locked lasers based on photonic integrated circuits (PICs) that are compact, manufactured at wafer scale and are compatible with further on-chip functionalities^4–6^. Yet, existing demonstrations to date lack the pulse energy required to drive nonlinear processes, such as supercontinuum generation. Here we demonstrate a mode-locked laser that overcomes this challenge through the use of erbium-ion-implanted silicon nitride PICs^7^. The laser is based on the Mamyshev oscillator architecture^8^, in which alternating spectral filtering and self-phase modulation enable mode-locking and can support large nonlinear phase shifts^9^. It operates without external seeding, delivering a 176-MHz pulse train with nanojoule pulse energy, comparable with fibre lasers and exceeding previous PIC-based sources by two orders of magnitude. The output exhibits high coherence, can be linearly compressed to 147 fs and can directly drive a 1.5-octave-spanning supercontinuum in a Si_3_N_4_ waveguide, without any further amplification. A compact terahertz time-domain spectrometer driven by this source achieved a bandwidth of 5 THz and a 90-dB dynamic range. We demonstrate its application in non-contact chemical analysis and inspection. Our results show the potential of an integrated ultrafast laser, with applications ranging from chip-scale frequency metrology to portable spectroscopy systems.

## Abstract (web summary)

A Mamyshev oscillator mode-locked laser compactly integrated on a photonic-chip delivers nanojoule, femtosecond pulses.

## Main

Mode-locked lasers (MLLs) are optical sources that generate trains of intense, ultrashort pulses by locking the phases of different longitudinal modes^10,11^. Since their inception, MLLs have become an indispensable tool across numerous fields, enabling advances in material processing, metrology^12^, biological imaging^13^, ophthalmic surgery^1^, femtochemistry^2^, optical spectroscopy and high-resolution ranging^14^. Recently, considerable efforts have been devoted to developing ultrafast pulse sources on photonic integrated circuits (PICs) with the aim of replicating tabletop-laser performance within a compact footprint while allowing for the integration of other functionalities on the same chip. For example, mode-locking of integrated III-V semiconductor lasers has been demonstrated both monolithically^15,16^ and through heterogeneous integrations with silicon nitride or lithium niobate photonics, which uses saturable absorption in semiconductors^5,17–19^ or radio-frequency modulation^6,20^. By using external semiconductor saturable absorber mirrors, mode-locking has also been achieved in low-confinement erbium-doped planar light-wave circuits^4^. Moreover, Kerr microcombs^21,22^ and electro-optic frequency combs^23^ have been shown to generate femtosecond pulses on-chip. However, all existing PIC-based ultrafast sources still fall far short of the performance of tabletop MLLs, in terms of noise, peak power, pulse duration and pulse energy. In particular, pulse energies reported in existing works remain at the few-picojoule level, limiting peak power for nonlinear optical processes such as octave-spanning supercontinuum generation.

Recent advances in high-confinement doped waveguides offer a promising way to scale up pulse energy. Demonstrations of high-power optical amplifiers in erbium-ion-implanted silicon nitride (Er:Si_3_N_4_)^7^, thulium-doped aluminium oxide (Tm:Al_2_O_3_)^24^ and titanium-doped sapphire waveguides^25,26^ highlight their potential as efficient gain media for integrated MLLs. These low-loss doped waveguides also support sub-metre-long laser cavities within compact chips, thus enabling low repetition rates of a few hundred megahertz, which are crucial for achieving a high pulse energy at a moderate average power. However, stable mode-locking has not yet been attained with these active waveguides, as previous attempts have been limited to Q-switched operation^27,28^.

**Fig. 1:**
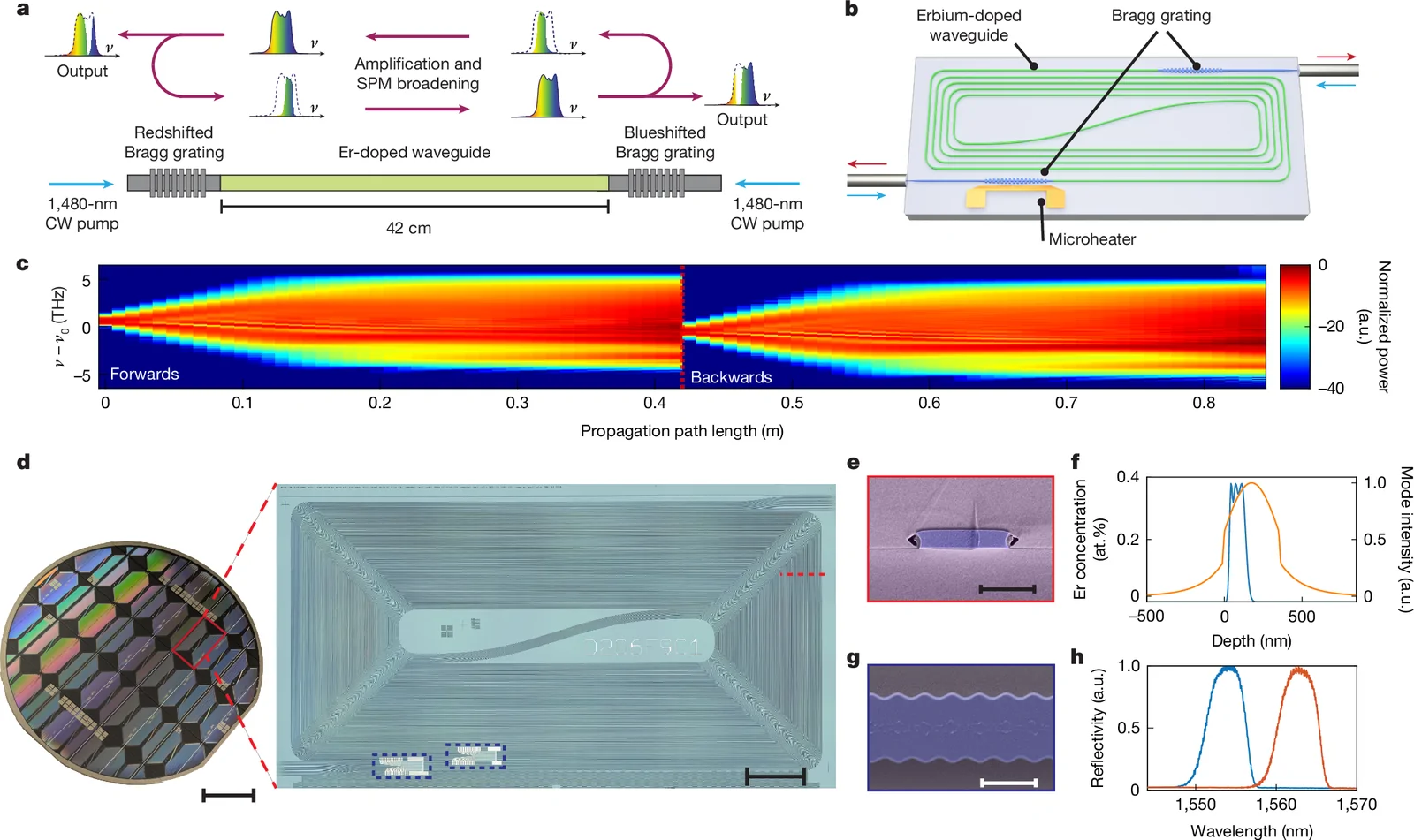
Principle and fabrication of the integrated MLLs based on a Mamyshev oscillator. **a**, Illustration of the working principle of the integrated Mamyshev oscillator, which consists of a 42-cm Er:Si_3_N_4_ waveguide sandwiched between two spectrally offset WBGs. SPM, self-phase modulation; *ν*, optical frequency; *ν*_0_, central frequency. **b**, Schematic of the integrated Mamyshev oscillator, showing the erbium-doped waveguide arranged in a spiral and the WBGs with an integrated microheater for tuning. **c**, Numerically simulated optical spectrum of the Mamyshev oscillator as a function of propagation path length in the linear cavity. **d**, Photographs of the fabricated wafer (left) and a chip that contains 26 individual MLLs (right). The blue boxes indicate the location of the WBGs. The internal tracking number for the MLL sample used in the experiment is D20602F8C2. **e**, False-coloured scanning electron microscope (SEM) image of the cross section of the doped waveguide. **f**, Simulated profile of the fundamental transverse-electric optical mode (orange) and the erbium concentration in the Si_3_N_4_ waveguide (blue). **g**, False-coloured SEM image of the corrugated WBG. **h**, Overlaid reflection spectra of the two WBGs, showing the spectral offset between the passbands. a.u., arbitrary units; CW, continuous wave. Scale bars, 2 cm (**d**, left), 2 mm (**d**, right), 1 μm (**e**,**g**).

To overcome the limitations of current integrated MLLs, we consider alternative mode-locking mechanisms^29^. The Mamyshev oscillator stands out as a particularly promising approach ^8,9^, as it enables record megawatt peak powers^30^ and few-cycle pulses^31^ in fibre-based lasers. The concept builds on the Mamyshev regenerators, originally proposed by P. V. Mamyshev for optical signal regeneration in soliton transmission, which exploits self-phase modulation and spectral filtering to produce a nonlinear transfer function. By concatenating two such regenerators that feature a nonlinear waveguide and two spectrally offset band-pass filters, a Mamyshev oscillator cavity can be formed (Fig. 1a), as first demonstrated in refs. ^32,33^. Unlike conventional mode-locking schemes, Mamyshev oscillators eliminate the need for a physical saturable absorber^8,9,34^. Instead, mode-locking arises from a combination of nonlinear broadening and filtering: low-power light is suppressed by the non-overlapping filters, while high-power pulses broaden spectrally in the nonlinear segment and pass through both filters to sustain lasing. A key advantage of the Mamyshev oscillator is its intrinsic tolerance to large intracavity nonlinear phase shifts as high as 60π (ref. ^9^), which can otherwise cause pulse break-up in conventional MLLs^35^. This challenge is particularly pronounced in high-confinement integrated waveguides, as their effective nonlinearities are three orders of magnitude higher than in fibres. Furthermore, a Mamyshev oscillator requires only two critical components: nonlinear waveguides and band-pass filters. The latter can simply be implemented on-chip using waveguide Bragg gratings (WBGs). This eliminates the need to integrate semiconductor saturable absorbers or engineer artificial ones, thus substantially reducing the complexity of integrated MLLs.

In this work, we demonstrate a PIC-based Mamyshev oscillator MLL with erbium-doped silicon nitride waveguides. This system can be self-seeded and achieves a high pulse energy for a PIC-based MLL (Supplementary Note 1). The oscillator delivers coherent nanojoule-level pulses that are linearly dechirped to 147 fs. We demonstrate the utility of the integrated Mamyshev oscillator pulses by demonstrating a terahertz time-domain spectrometer and the on-chip generation of 1.5-octave-spanning supercontinuum, both directly driven by the laser.

## An integrated Mamyshev oscillator

Figure 1a illustrates the working principle of an integrated MLL based on a Mamyshev oscillator. A 42-cm-long section of an Er:Si_3_N_4_ waveguide is placed between two spectrally offset WBGs to form a simple linear cavity with a 175.5-MHz free spectral range. As simulated in Fig. 1c (Supplementary Note 2), pulses reflected by one grating undergo simultaneous amplification and self-phase modulation, which broaden their spectrum to bridge the spectral gap between the WBGs and eventually establish stable mode-locked operation.

**Extended Data Fig. 1:**
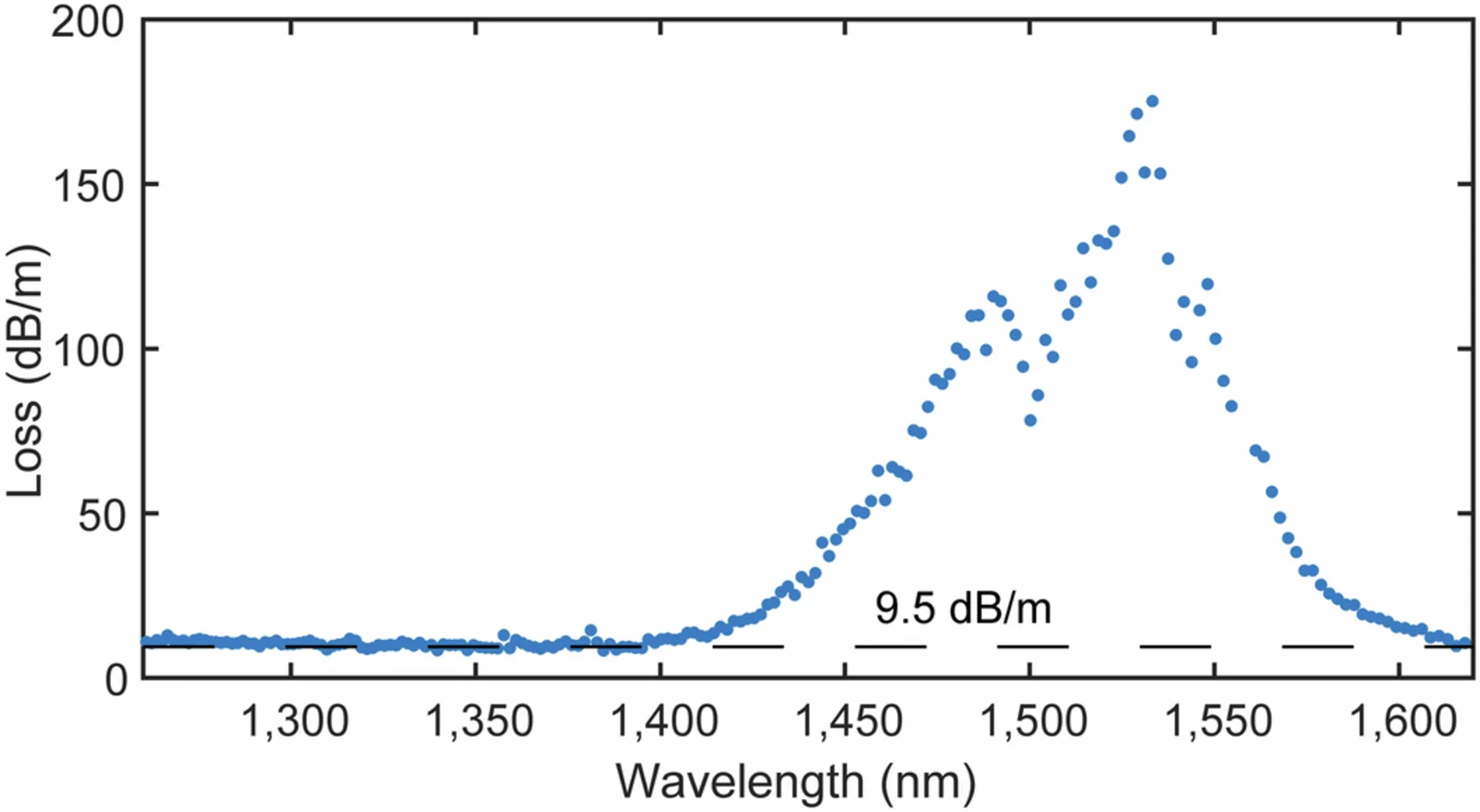
Waveguide loss after ion implantation measured by OFDR. The dashed line denotes the estimated background loss outside of the erbium absorption band.

To maximize nonlinear interactions while maintaining low optical loss (Extended Data Fig. 1), we use a narrow few-mode Si_3_N_4_ waveguide with a cross section of 1.6 μm × 0.35 μm (Fig. 1e). Erbium ions are implanted into the waveguide to provide an optical gain^7^ using a maximum implantation energy of 500 keV and a total fluence of 3.87 × 10^15^ cm^−2^. This results in an estimated peak doping concentration of approximately 0.4 at.% and a 22% overlap between the mode and the dopant profile^7^ (Fig. 1f). The WBGs, shown in Fig. 1g, serve as narrowband reflectors for the pulses while simultaneously enabling injection of the 1,480-nm pump and partial transmission for the MLL output. The fabricated WBGs exhibit 3-dB bandwidths of approximately 5 nm and a centre wavelength separation of 8.9 nm (Fig. 1h). The reflection bands were designed to be near 1,557 nm to optimally use the erbium gain bandwidth. Microheaters are placed above the WBGs to facilitate fine-tuning of their reflection wavelengths (Fig. 1b). For prototyping, 26 independent MLLs were placed in a parallel waveguide bundle and collectively routed in a spiral layout on a 2 cm × 1.1 cm chip (Fig. 1d), which yielded over 300 MLLs per wafer.

## Mode-locking operation

**Fig. 2:**
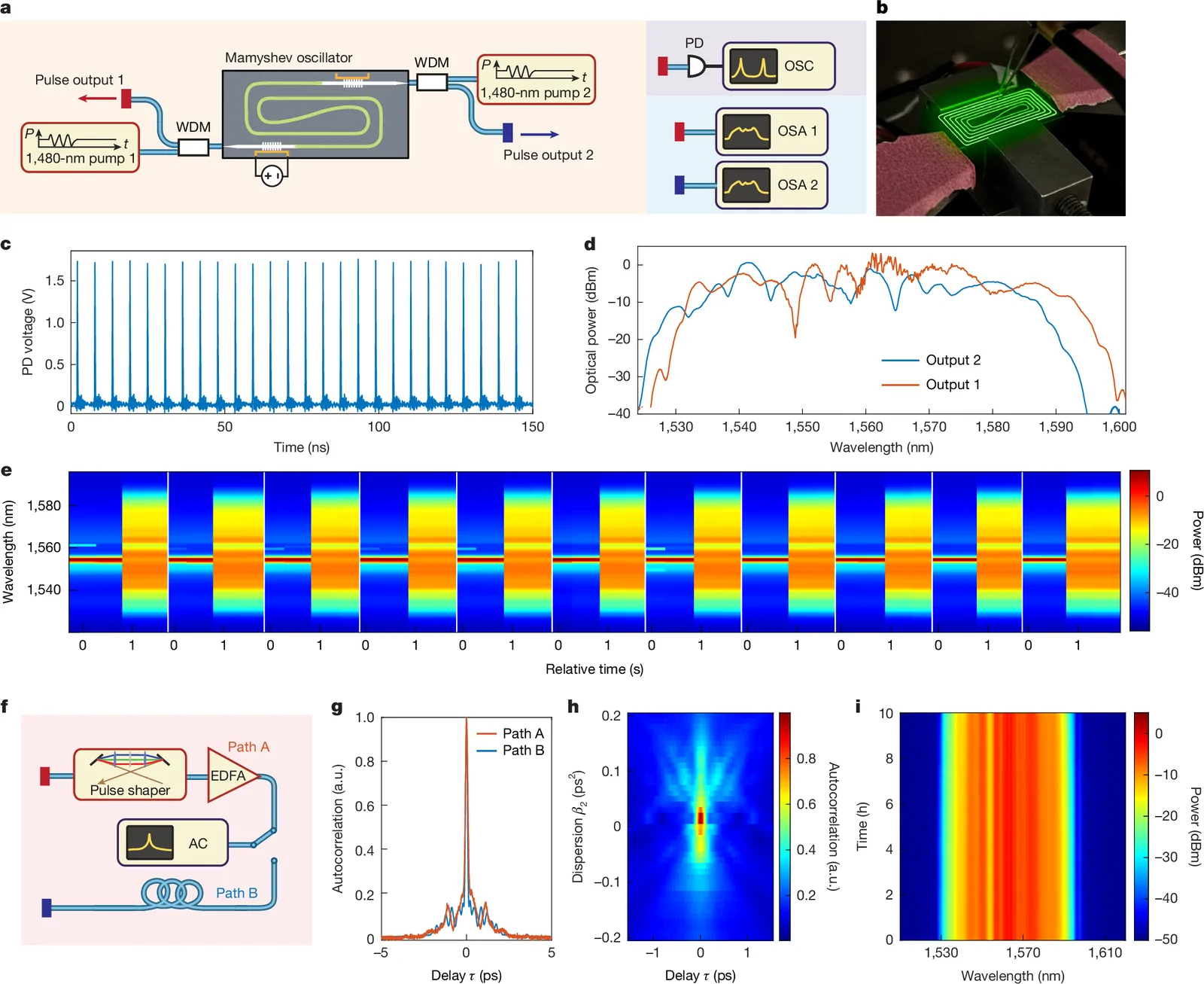
Characterization of a photonic integrated circuit-based Mamyshev oscillator. **a**, Experimental set-up for MLL operation and basic characterization. The on-chip Mamyshev oscillator is pumped by 1,480-nm diode lasers through the WBGs, and it is self-seeded by temporarily modulating the pump current. The optical spectra and temporal characteristics are measured using optical spectrum analysers and a fast photodetector with an oscilloscope. **b**, Photograph of the integrated MLL in operation. **c**, Stable pulse train acquired through direct photodetection of the MLL output after disabling the self-seeding pump modulation. **d**, Optical spectra acquired at the two output ports of the integrated MLL at high pump power, normalized to a resolution bandwidth of 0.2 nm. **e**, Stacked spectrograms showing the self-seeding processes. **f**, Experimental set-up for characterizing the second-order intensity autocorrelation function. **g**, Intensity autocorrelation traces after optimized linear chirp compression using either a programmable filter (pulse shaper) and erbium-doped fibre amplifier (path A) or through approximately 10 m of a single-mode silica fibre (path B). **h**, Evolution of the autocorrelation function under varying compensation for the group delay dispersion. **i**, Spectrogram of output 1 recorded continuously over 10 h when pumped with 820-mW pump 2. AC, non-collinear second-order intensity autocorrelator; EDFA, erbium-doped fibre amplifier; OSA, optical spectrum analyser; OSC, oscilloscope; PD, photodetector; WDM, wavelength (de-)multiplexer.

Figure 2a illustrates the experimental set-up used to operate and characterize the integrated MLL (Supplementary Note 3). For maximum output power, the MLL is optically pumped from both ends of the waveguide with two 1,480-nm lasers (pumps 1 and 2), which deliver 820 mW and 959 mW off-chip optical power, respectively. These power levels are readily achievable using commercially available laser diodes in butterfly packages after polarization combining, thus offering possibilities for future hybrid integration of the pump source.

The MLL can either be self-seeded by a temporary modulation to the pump power or be seeded with a single pulse from an external MLL (Supplementary Notes 5 and 6), like fibre-based Mamyshev oscillators^8,9,36^. For self-seeding, a modulation of 1.2 MHz to 2.8 MHz is applied to the drive current of the pump laser diodes for a duration of less than 10 s and is then disabled when the mode-locked pulses appear. The self-seeding process is highly repeatable and has been shown to yield identical mode-locked states in each experiment (Fig. 2e).

**Extended Data Fig. 2:**
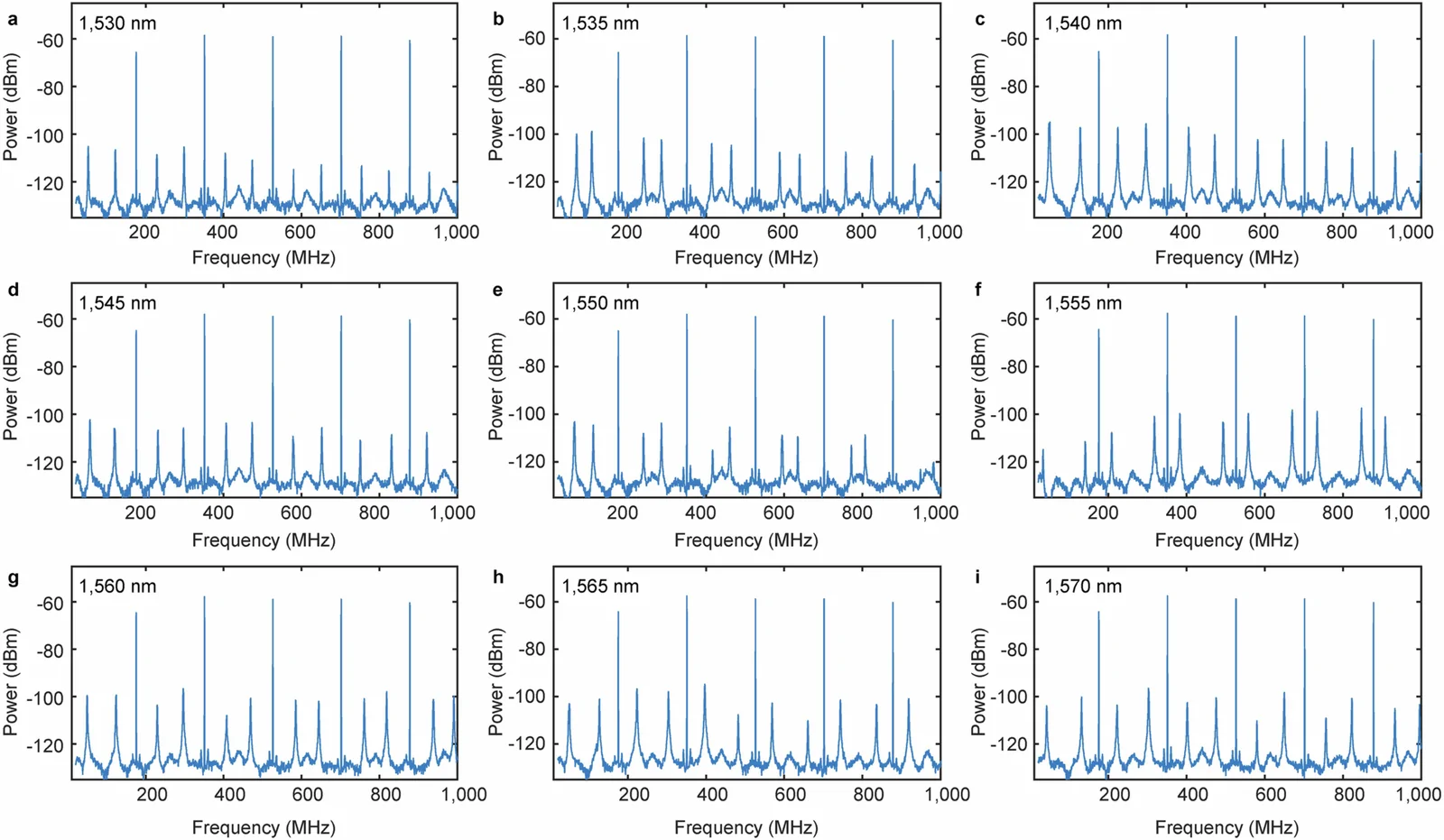
Heterodyne beat note. **a** to **i**, Heterodyne beat note at 9 individual wavelengths from 1530 nm to 1570 nm.

Upon initiation, self-sustained mode-locking is established without a continuous-wave background or Q-switching instabilities, as confirmed by the stable pulse train detected using a d.c.-coupled fast photodetector (Fig. 2c). The optical spectra collected from both waveguide outputs (Fig. 2d) are centred at approximately 1.56 μm and show 64 nm and 47 nm of 20 dB bandwidth, thus spanning the entire telecom C-band and extending into the L-band. This corresponds to over 40,000 comb lines (Methods and Extended Data Fig. 2). Average output powers of 136 mW (output 1) and 138 mW (output 2) were measured from the two ends of the linear cavity, corresponding to on-chip powers of 182 mW and 184 mW after accounting for coupling loss (Supplementary Note 7), yielding a pump-to-output efficiency of 27.5% when the powers are summed for the two outputs. With a repetition rate *f*_rep_ = 175.5 MHz, the pulse energies at each output reached 1.04 nJ and 1.05 nJ, over two orders of magnitude higher than previously reported for PIC-based ultrafast sources. For power-constrained applications, stable mode-locking is also achieved using only the 820-mW pump power delivered by a diode laser (pump 2). In this lower power configuration, the oscillator produces on-chip pulse energies of 397 pJ and 347 pJ from outputs 1 and 2, respectively, still surpassing all previous integrated ultrafast sources by a wide margin. As shown by a spectrogram (Fig. 2i), the integrated MLL can maintain stable mode-locking for over 10 h, demonstrating the robustness of the mode-locked state and the long-term integrity of the waveguide.

The output pulses from the integrated Mamyshev oscillator are predominantly linearly chirped, in agreement with numerical simulations. To characterize the pulses, we used an intensity autocorrelator in conjunction with a programmable filter to apply a variable group delay dispersion and a home-built erbium-doped fibre amplifier to compensate for loss in the filter. Sweeping the applied dispersion, the pulse undergoes compression and rebroadening (Fig. 2h), which allowed us to identify the optimal dispersion for pulse compression. Moreover, the pulse waveform can be computationally reconstructed from the dispersion sweep data (Supplementary Note 8). With the optimized group delay dispersion, the intensity autocorrelation function of the output pulse from output 1 is compressed to 187 fs full-width at half-maximum (Fig. 2g). Alternatively, using a single-mode silica fibre as a delay line (approximately 10 m long, including other components in the set-up) for dispersion compensation, the pulse from output 2 is directly compressed to an autocorrelation width of 147 fs. Such dispersion compensation can be implemented on the Si_3_N_4_ platform using chirped Bragg gratings, although this was not implemented in the present device.

## Coherence characterization

**Fig. 3:**
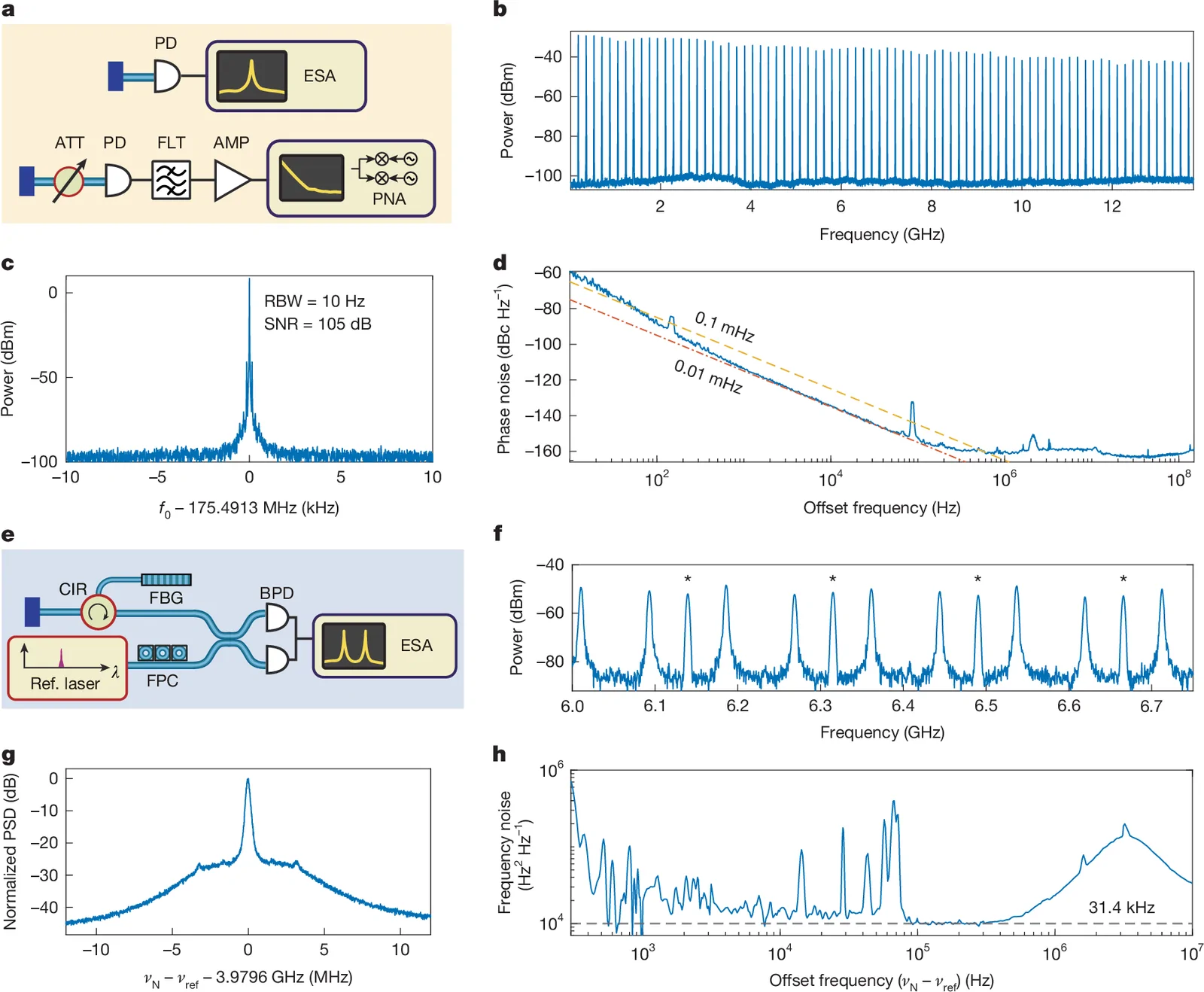
Characterizing the coherence of an MLL with a photonic integrated Mamyshev oscillator. **a**, Schematic for the characterization of the repetition-rate beat note through direct photodetection. **b**, Spectrum of the photodetected signal at 100 kHz RBW, showing several harmonics of the repetition rate *f*_rep_. **c**, Measured *f*_rep_ beat note with SNR of 105 dB at 10-Hz resolution bandwidth. *f*_0_ , radio frequency. **d**, Phase noise of the *f*_rep_ beat note, with reference lines corresponding to Lorentzian linewidths of 0.1 mHz and 0.01 mHz. **e**, Schematic of the set-up for characterizing the heterodyne beat note of the integrated MLL. A portion of the MLL spectrum is preselected by a fibre Bragg grating and interferes with a reference continuous-wave laser. **f**, Spectrum of the heterodyne beat note between the reference laser and the integrated MLL at 3 MHz RBW. Asterisks denote residual *f*_rep_ harmonics resulting from imperfect common-mode rejection in the balanced photodetector. **g**, PSD of the heterodyne beat note centred at 3.9796 GHz with an RBWof 12.47 kHz. *ν*_N_ and *ν*_0_, optical frequencies of the comb lines and the reference laser, respectively. The linewidth for a measurement time of 10 ms is 174 kHz. **h**, Frequency noise of the heterodyne beat note in **g**, indicating the 31.4-kHz Lorentzian linewidth for the optical comb line of the integrated MLL. AMP, amplifier; ATT, attenuator; BPD, balanced photodetector; CIR, circulator; ESA, electronic signal analyser; FBG, fibre Bragg grating; FPC, fibre polarization controller; FLT, filter; PNA, phase noise analyser; RBW, resolution bandwidth.

For many applications, MLLs are desired to be low noise in terms of pulse interval and pulse-to-pulse phase slip^37^. We evaluated the coherence of the integrated Mamyshev oscillator with direct photodetection (Fig. 3a) and heterodyne beating with a reference laser (Fig. 3e). The photodetected signal exhibits tens of harmonics of *f*_rep_, which extend well into the microwave range (Fig. 3b). Using a low-noise amplifier and appropriate filtering (Supplementary Note 3), the signal-to-noise ratio (SNR) of the fundamental beat note at *f*_rep_ was measured to be 105 dB at 10-Hz resolution bandwidth, approaching the limit of the instrument. To estimate the timing jitter of the MLL, the power spectral density (PSD) of the single-sideband phase noise of the sixth repetition-rate harmonic was characterized with a phase noise analyser. After scaling down to the fundamental 175.5-MHz beat note, the single-sideband phase noise at 10 kHz offset reached −134 dBc Hz^−1^. The integrated timing jitter between 10 kHz and 10 MHz was 59.1 fs. Moreover, by fitting the 1/*f*^2^ slope to the *f*_rep_ phase noise between 1 kHz and 50 kHz offset, we extracted a Lorentzian linewidth of 0.012 mHz, which represents a notable improvement over the best reported value from integrated semiconductor MLLs^5^.

The heterodyne beat note between the MLL and a continuous-wave reference laser at 1,552 nm (Fig. 3f) reveals a series of regularly spaced beat notes, indicating a stable frequency comb with a well-defined carrier-envelope offset frequency over the measurement duration. To quantify the linewidth of individual comb lines (Fig. 3g), we acquired the beat signal with an electronic signal analyser and extracted the PSD of its single-sideband frequency noise (Fig. 3h). By fitting the noise floor near a 200 kHz offset, we determined a Lorentzian linewidth of 31.4 kHz. Spurs observed between 10 kHz and 100 kHz were probably caused by the intensity noise of the 1,480-nm pump laser, and the bump between 1 MHz and 10 MHz may be attributed to a relaxation oscillation. The frequency noise between 1 kHz and 100 kHz offset remained below 5 × 10^4^ Hz^2^ Hz^−1^, which we attribute to the large cavity volume and, thus, low thermorefractive noise^38^.

**Fig. 4:**
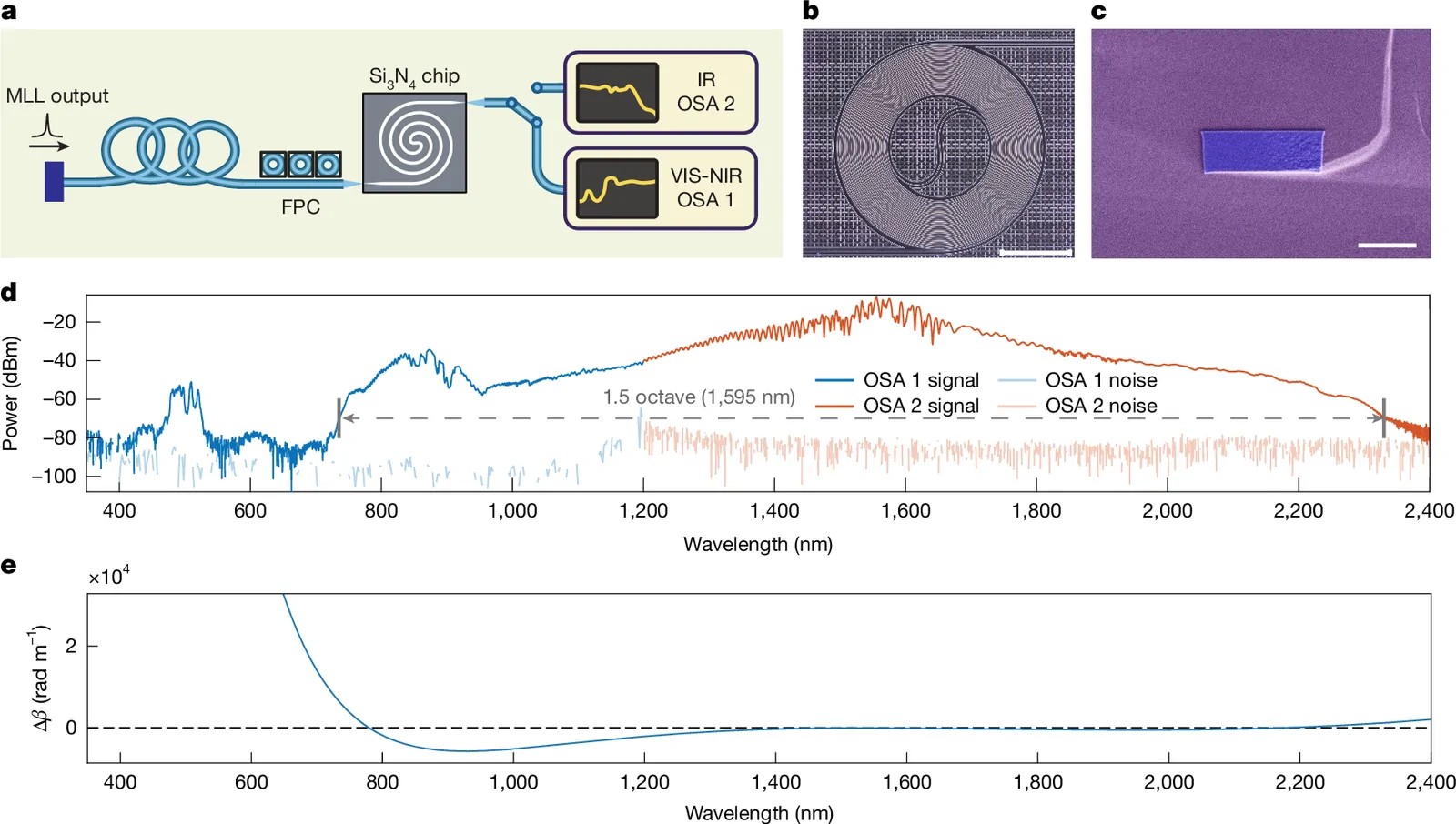
Supercontinuum generation directly driven by the integrated Mamyshev oscillator. **a**, Experimental set-up for supercontinuum generation in a Si_3_N_4_ spiral waveguide, directly driven by the integrated MLL without post-amplification. **b**, Photograph of a Si_3_N_4_ spiral waveguide chip identical to the one used for supercontinuum generation. The internal tracking number of the sample used in the experiment is D7802F4C4. **c**, False-coloured SEM image of the cross section of the waveguide. **d**, Spectrum of the generated 1.5-octave-spanning supercontinuum, normalized to a resolution bandwidth of 2 nm. The 20-dB bandwidth is 18.7 THz. **e**, Simulated integrated dispersion Δ*β* of the spiral waveguide used in the experiment. IR, infrared; VIS-NIR, visible to near-infrared. Scale bars, 200 μm (**b**), 1 μm (**c**).

## Direct supercontinuum generation

One application of the high-energy on-chip pulse sources is supercontinuum generation, which is an efficient route for producing spatially and temporally coherent optical spectra that can span an octave. Crucially, our integrated Mamyshev oscillator provides sufficient pulse energy such that it does not require any further external amplification. As a proof of concept, we routed the output of the integrated MLL through approximately 10 m of a single-mode silica fibre, followed by coupling into a 43.7-mm Si_3_N_4_ spiral waveguide on a separate chip to generate a supercontinuum (Fig. 4a). This fibre link provides dispersion compensation for the chirped MLL pulses and linearly compresses them to reach kilowatt-level peak power. The Si_3_N_4_ spiral waveguide (Fig. 4b) features a 2.07 μm × 0.70 μm cross section (Fig. 4c) and a relatively flat integrated dispersion near the telecom C-band (Fig. 4e). Without optical amplification, the MLL output generated a 1.5-octave-spanning supercontinuum from 736 nm to 2,331 nm (Fig. 4d), which matches numerical simulations (Supplementary Note 9). Such a supercontinuum can serve as an ideal wideband high-brightness source for infrared spectroscopy with integrated spectrometers^39^ and for optical coherence tomography^40^, and it is a step towards an integrated self-referenced frequency comb^12^.

## Application for THz-time domain spectroscopy

Our photonic integrated Mamyshev oscillator can serve as a high-performance, compact replacement for conventional MLLs in a range of applications. One compelling example is in terahertz time-domain spectroscopy (THz-TDS). The terahertz band lies between the microwave and infrared regions of the electromagnetic spectrum and offers several distinctive advantages: terahertz radiation is non-ionizing, penetrates many optically opaque materials, provides higher spatial resolution than millimetre waves and contains rich spectroscopic signatures of molecules^41^. These properties enable applications including security screening^42^, non-destructive testing^43^ and art conservation^44^.

A central technique in terahertz spectroscopy is THz-TDS, in which an ultrafast optical pulse train generates broadband terahertz transients and samples their electric field in the time domain, commonly using photoconductive antennas or nonlinear optical processes^41^. However, most THz-TDS systems rely on a solid-state or fibre-based MLL, which is a primary contributor to system cost and footprint, thereby limiting their broader deployment.

**Fig. 5:**
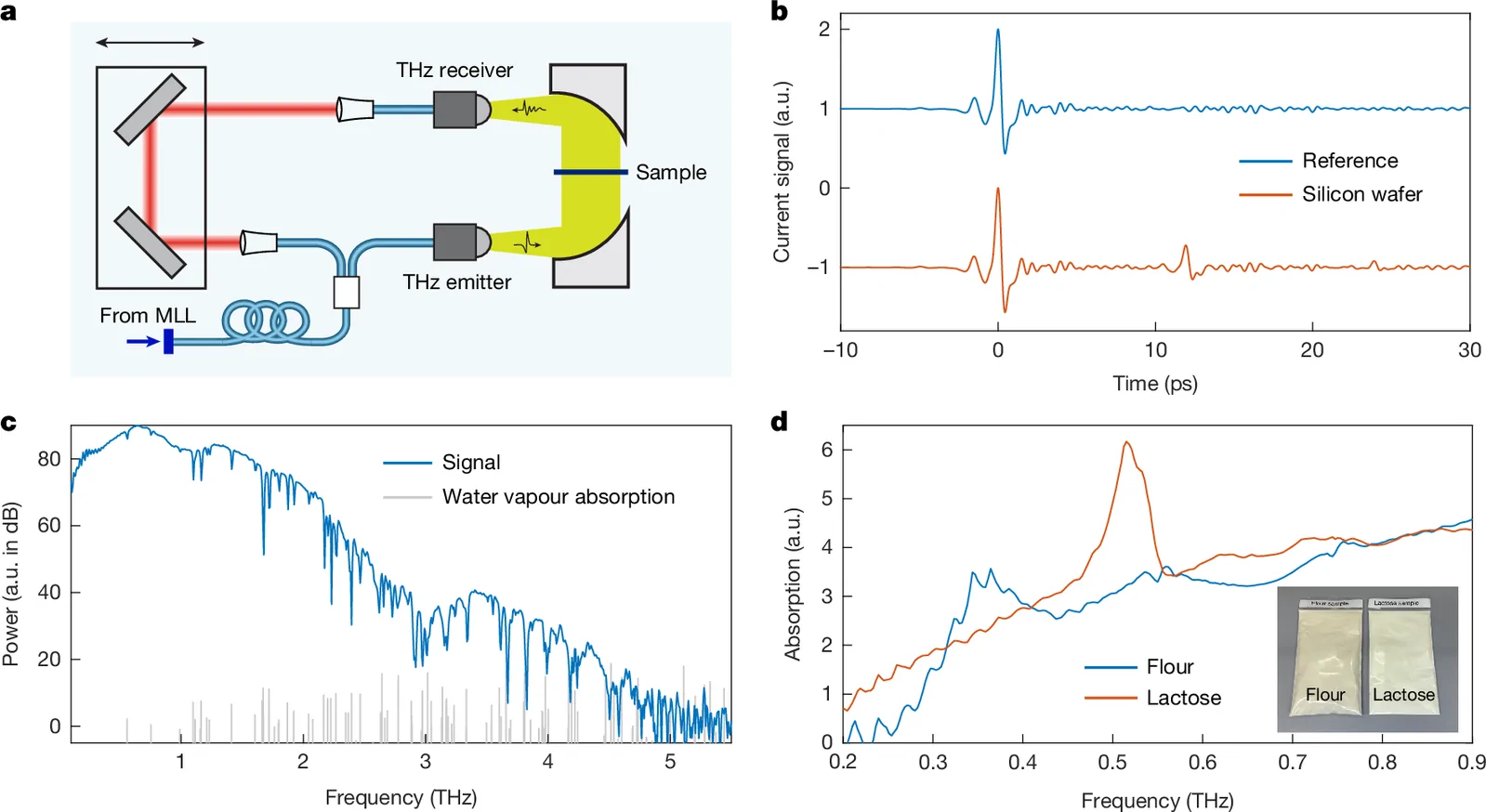
THz-TDS system directly driven by the integrated Mamyshev oscillator. **a**, Experimental set-up of a transmissive THz-TDS system driven by the integrated MLL. **b**, Measured THz-TDS electric-field waveforms with a clear beam path (reference) and with a silicon wafer inserted, showing delayed replicas arising from several internal reflections. **c**, Terahertz power spectra with approximately 7-GHz resolution obtained from the Fourier transform of the reference signal in **b**. Absorption lines for water vapour are from the HITRAN database^49^. **d**, Terahertz absorption spectra of flour and lactose samples, showing a clear lactose absorption feature near 0.53 THz. Inset, photograph of the powders used in the measurement.

In this work, we used our photonic integrated Mamyshev oscillator to directly drive a THz-TDS system (Fig. 5a). We achieved a peak dynamic range of 90 dB and a detection bandwidth of 5 THz (Fig. 5c) with approximately 5 minutes of averaging, which were enabled by the high average power and the short pulse duration after linear dechirping. Absorption lines from atmospheric water vapour in the terahertz beam path are clearly resolved in the acquired spectrum.

We further demonstrate two proof-of-concept applications of the system (Supplementary Note 3). First, we performed thickness metrology in a time-of-flight measurement by transmitting terahertz waves through a silicon wafer. Delayed replicas of the main pulse appeared due to internal reflection. The 11.93-ps delay measured between the main pulse and the first replica corresponds to a thickness of 523.3 μm (Fig. 5b), consistent with the nominal thickness of the silicon wafer (525 μm). Second, in a material identification application, the measured absorption spectra clearly distinguish lactose powder from flour by resolving the characteristic feature near 0.53 THz (ref. ^45^; Fig. 5d). These proof-of-concept demonstrations highlight the potential of integrated-MLL-driven THz-TDS as a path towards portable, cost-effective systems for wide industrial deployment.

## Discussion and conclusions

We demonstrate an integrated Mamyshev oscillator MLL based on erbium-ion-implanted Si_3_N_4_ PICs. It can generate ultrafast pulses with nanojoule-level energy at a 175.5-MHz repetition rate, a parameter regime so far attained only with non-chip-scale MLLs, such as those based on fibres. The integrated MLL enables the generation of a 1.5-octave-spanning supercontinuum in a separate dispersion-engineered Si_3_N_4_ waveguide without extra amplification, which lays the foundation for on-chip self-referenced frequency combs, with future work to study reducing and understanding the phase noise of the free-running laser. The laser delivers pulses that can be compressed to 147 fs and exhibits excellent coherence compared with existing integrated MLLs. A compelling future direction is to combine the MLL with octave-spanning supercontinuum generation directly on the same photonic chip by integrating a dispersion-engineered waveguide and pulse compression. The latter could unlock octave-spanning optical frequency combs to create a phase-coherent radio-frequency-to-optical link. Optical pulses from an integrated Mamyshev oscillator could also directly drive a THz-TDS system, with performance comparable with state-of-the-art commercial systems and superior to all existing THz-TDS demonstrations driven by integrated sources (see Supplementary Note 10 for a comparison). Although our demonstration is based on silicon nitride waveguides and in the 1.55-μm band, the Mamyshev oscillator concept is readily transferable to other platforms and wavelengths. Simulations indicate that the nonlinear Er:Si_3_N_4_ waveguide amplifier section could be operated individually for similariton pulse amplification (Supplementary Note 11).

The combination of high pulse energy, excellent coherence and stable mode-locking demonstrated with wafer-scale manufactured PICs opens new opportunities for nonlinear integrated photonics, including mid-infrared supercontinuum sources for spectroscopy^46^, handheld low-cost terahertz systems for non-destructive testing^47^ and chip-scale frequency combs for optical atomic clocks^48^. Beyond this, our work highlights the emerging frontier of integrated photonics and ultrafast laser science, which so far has been explored using fibres and bulk laser cavities, with erbium- or other rare-earth-ion-doped silicon nitride nonlinear PICs.

## Methods

### Sample fabrication process

The erbium-doped wafers used in this study (D20602) were fabricated using a subtractive process^50,51^. The process started with 100-mm silicon wafers (SIEGERT WAFER GmbH). A 10-μm thermal SiO_2_ layer was grown by wet oxidation. A 362-nm stoichiometric Si_3_N_4_ film was deposited by low-pressure chemical vapour deposition. The thickness compensated for shrinkage during subsequent annealing, yielding a final thickness close to 350 nm. Waveguide patterns were defined by deep-ultraviolet lithography (ASML PAS5500/350C, JSR M108Y, Brewer Science DUV-42P) and fluorine-based reactive-ion etching using CHF_3_/SF_6_/Ar/O_2_ gases (SPTS APS). For isolated features, the critical dimension shrunk by approximately 55 nm on each side; this bias was compensated for in the edge coupler design and in the calibration of the WBG parameters. After stripping the resist, the backside Si_3_N_4_ was removed by reactive-ion etching while the front side was protected with a photoresist. The wafers were megasonically cleaned to remove particle contamination and then annealed at 1,200 °C for 12 h in N_2_. Removing the backside Si_3_N_4_ before annealing was crucial, as the 350-nm film has sufficient internal stress to cause plastic deformation and permanent wafer bowing, which would impede subsequent fabrication steps. Ultraviolet direct-write lithography (Heidelberg MLA150, 6 μm AZ 15nXT) then defined the implantation mask, which protected the WBGs and edge couplers.

Erbium ions were implanted using a 500-kV air-insulated implanter. Singly charged ions were extracted from an indirectly heated cathode Bernas-type ion source with a 40-kV extraction system and post-accelerated to energies up to 500 keV, then mass-separated by an analysing magnet and transported using quadrupoles and an Einzel lens. A neutral trap prevented neutral particles from reaching the target. The ion beam was scanned in the *x* and *y* directions at approximately 1 kHz with a slight frequency offset. To tailor a dopant profile across the film thickness, implantation was performed sequentially at 125 keV, 253 keV and 500 keV with doses of 8.40 × 10^14^ cm^−2^, 1.41 × 10^15^ cm^−2^ and 2.84 × 10^15^ cm^−2^, respectively. All implantation was conducted at room temperature with a 0° incidence angle.

After ion implantation, the photoresist was removed by oxygen plasma. The surface was cleaned in concentrated hydrochloric acid to eliminate erbium oxide residues and in dilute hydrofluoric acid to remove resputtered material. The wafers were annealed at 900 °C for 1 h in N_2_ to repair radiation damage to the Si_3_N_4_ waveguide. Approximately 6 μm of SiO_2_ cladding was deposited by inductively coupled plasma chemical vapour deposition using a SiCl_4_ precursor^52^, followed by an anneal at 600 °C for 1 h in O_2_ to reduce the optical loss. Metallic microheaters were fabricated by sputtering approximately 25 nm of Ti and approximately 500 nm of Pt, followed by ultraviolet lithography (Heidelberg MLA150, 2 μm AZ 10nXT) and ion-beam etching (Veeco IBE350). The photoresist was reflown at 135 °C to reduce etch fencing. Dies were separated by ultraviolet lithography (Heidelberg MLA150, 10 μm AZ 15nXT), followed by deep reactive-ion etching through the SiO_2_ and approximately 250 μm of Si. The backside was ground to give a final chip thickness of approximately 250 μm. Finished dies were baked on a 310 °C hotplate overnight to heal Si_3_N_4_ damage incurred due to ultraviolet exposure from intense plasmas during fabrication, which can increase the waveguide optical loss^50^.

The current process has two known issues that are readily addressed. First, the highly stressed 350-nm Si_3_N_4_ film can crack within days if not promptly patterned. Such cracks can destroy the waveguides and reduce the yield, especially after annealing-induced shrinkage. Patterning deep trenches (in a 2-μm-wide square grid, approximately 3 μm deep, like what was used in ref. ^50^) into the oxide in a ring along the wafer edge effectively prevented cracking. Second, high-dose implantation can swell the Si_3_N_4_ surface and form overhanging edges that lead to the formation of voids during the deposition of SiO_2_ cladding with inductively coupled plasma chemical vapour deposition, which increases the scattering loss. This can be mitigated by introducing lateral cladding before implantation, for example, with the photonic damascene process^7,53^ or with low-pressure chemical vapour deposition followed by chemical-mechanical polishing and controlled hydrofluoric acid etching.

The dispersion-engineered Si_3_N_4_ waveguides used for the supercontinuum demonstration (D7802) were fabricated using the photonic damascene process, as described in ref. ^53^.

### Waveguide loss and erbium absorption

We characterized the total waveguide loss of D20602 samples after ion implantation using optical frequency-domain reflectometry with a home-built optical vector network analyser^54^. Extended Data Fig. 1 shows the measured small-signal loss spectrum, which reveals broadband erbium absorption between approximately 1,400 nm and 1,600 nm, with a peak loss of approximately 165 dB m^−1^ at 1,533 nm, about twice that of a commercial highly erbium-doped fibre (Liekki ER80-8/125). Outside the erbium absorption band, we estimated the background waveguide loss to be 9.5 dB m^−1^, as inferred from the loss between 1,300 nm and 1,370 nm.

### Heterodyne beat note across a wide wavelength range

In the integrated Mamyshev oscillator MLL, the repetition rate of 175.5 MHz is lower than the minimal resolution bandwidth of available grating-based optical spectrum analysers (0.02 nm), such that the individual comb lines cannot be resolved. To visualize the comb-like spectrum, we characterized the heterodyne beat note of the MLL output with a tunable single-frequency laser (Toptica CTL) and a balanced photodetector at nine different wavelengths. Because of the wide wavelength range to cover, the fibre Bragg grating preselector can no longer be used, as in Fig. 3e. The SNR may appear lower and vary from line to line owing to the more pronounced saturation in the balanced photodetector (and, thus, distortion in its time-domain response) caused by the higher pulse energy. As shown in Extended Data Fig. 2, we observed discrete beat notes at all nine wavelengths in the emission bandwidth, indicating discrete comb lines within ±1 GHz of each observation wavelength.

## Online content

Any methods, additional references, Nature Portfolio reporting summaries, source data, extended data, supplementary information, acknowledgements, peer review information; details of author contributions and competing interests; and statements of data and code availability are available at https://doi.org/10.1038/s41586-026-10517-4.

## Supplementary information

Supplementary Information This file includes Supplementary Notes 1–11 covering performance benchmarks; simulations and modelling of the laser and supercontinuum generation; detailed descriptions of the experimental set-up; notes on fibre-to-chip coupling, pulse reconstruction and simulations of single-pass pulse amplification.

Peer Review File

## Data Availability

All experimental datasets used to produce the results are available at Zenodo (https://doi.org/10.5281/zenodo.18732610)^55^.

## Code availability

The laser simulation code is available at Zenodo (https://doi.org/10.5281/zenodo.18732610)^55^.
