# Supplementary material for: High-pulse-energy integrated mode-locked laser using a Mamyshev oscillator
Source: Nature. 2026 Jun 3;654(8117):57–63. doi: 10.1038/s41586-026-10517-4 (PMC13661250; doi:10.1038/s41586-026-10517-4)
Supplement: Supplementary file 1 [file 41586_2026_10517_MOESM1_ESM.pdf]

nature portfolio

https://doi.org/10.1038/s41586-026-10517-4

Supplementary information

High-pulse-energy integrated mode-locked laser using a Mamyshev oscillator

In the format provided by the authors and unedited

Supplementary Notes for:
High-pulse-energy integrated mode-locked laser using a Mamyshev oscillator

Zheru Qiu†,[1,2] Xuan Yang†,[1,2] Xurong Li†,[1,2] Jianqi Hu†,[1,2] Zhongshu Liu,[1,2] Yichi Zhang,[1,2] Xinru Ji,[1,2] Jiale Sun,[1,2] Grigory Lihachev,[1,2,3] Zihan Li,[1,2] Ulrich Kentsch,[4] and Tobias J. Kippenberg*[1,2]

[1] *Institute of Physics, Swiss Federal Institute of Technology Lausanne (EPFL), CH-1015 Lausanne, Switzerland*
[2] *Institute of Electrical and Microengineering, EPFL, CH-1015 Lausanne, Switzerland*
[3] *Current address: EDWATEC SA, EPFL Innovation Park, CH-1015 Lausanne, Switzerland*
[4] *Helmholtz-Zentrum Dresden-Rossendorf (HZDR), D-01328 Dresden, Germany*

Contents

S1. Comparison with state-of-the-art photonic integrated circuit-based ultrafast sources SP2

S2. Simulation of integrated Mamyshev oscillator and design guidelines SP4
- A. Method SP4
- B. Parameters SP6
- C. Simulation results SP8
- D. Design analysis guidelines SP11

S3. Detailed description of the experiment setup SP13
- A. Basic mode-locking operation SP13
- B. Self-seeding by pump modulation SP14
- C. Autocorrelation characterization SP14
- D. Coherence characterization SP15
- E. External seeding SP15
- F. Alternative starting method by extended cavity Q-switching SP16
- G. Supercontinuum generation SP16
- H. THz time-domain spectroscopy SP17

S4. Details on the waveguide Bragg gratings SP18

S5. Dynamics of pulse build-up SP18

S6. Time to start for self-seeding SP21

S7. Fiber-to-chip coupling and output beam quality SP22

S8. Time domain pulse reconstruction from dispersion sweep data SP23

S9. Simulation and additional experimental results of supercontinuum generation SP25

S10. Comparison with state-of-the-art terahertz TDS systems driven by integrated ultrafast sources SP27

S11. Single-pass pulse propagation in the gain section SP28

References SP29

†:These authors contributed equally.
*: tobias.kippenberg@epfl.ch

S1. Comparison with state-of-the-art photonic integrated circuit-based ultrafast sources

Here, we compare the key performance metrics of our work with state-of-the-art integrated ultrafast sources reported in the literature (Table S1). For completeness, we present a comprehensive list of integrated ultrafast sources, including some semiconductor and waveguide lasers that may not strictly be considered photonic integrated circuits (PICs) by today's standards due to fabrication process compatibility and mode confinement. In addition, a selection of small commercially available mode-locked lasers (MLLs) and notable recent laboratory demonstrations of fiber-based MLLs are also listed for reference. Figure S1 compares the pulse energy E_p and the pulse interval ($1/f_\mathrm{rep}$) of the sources listed in Table S1.

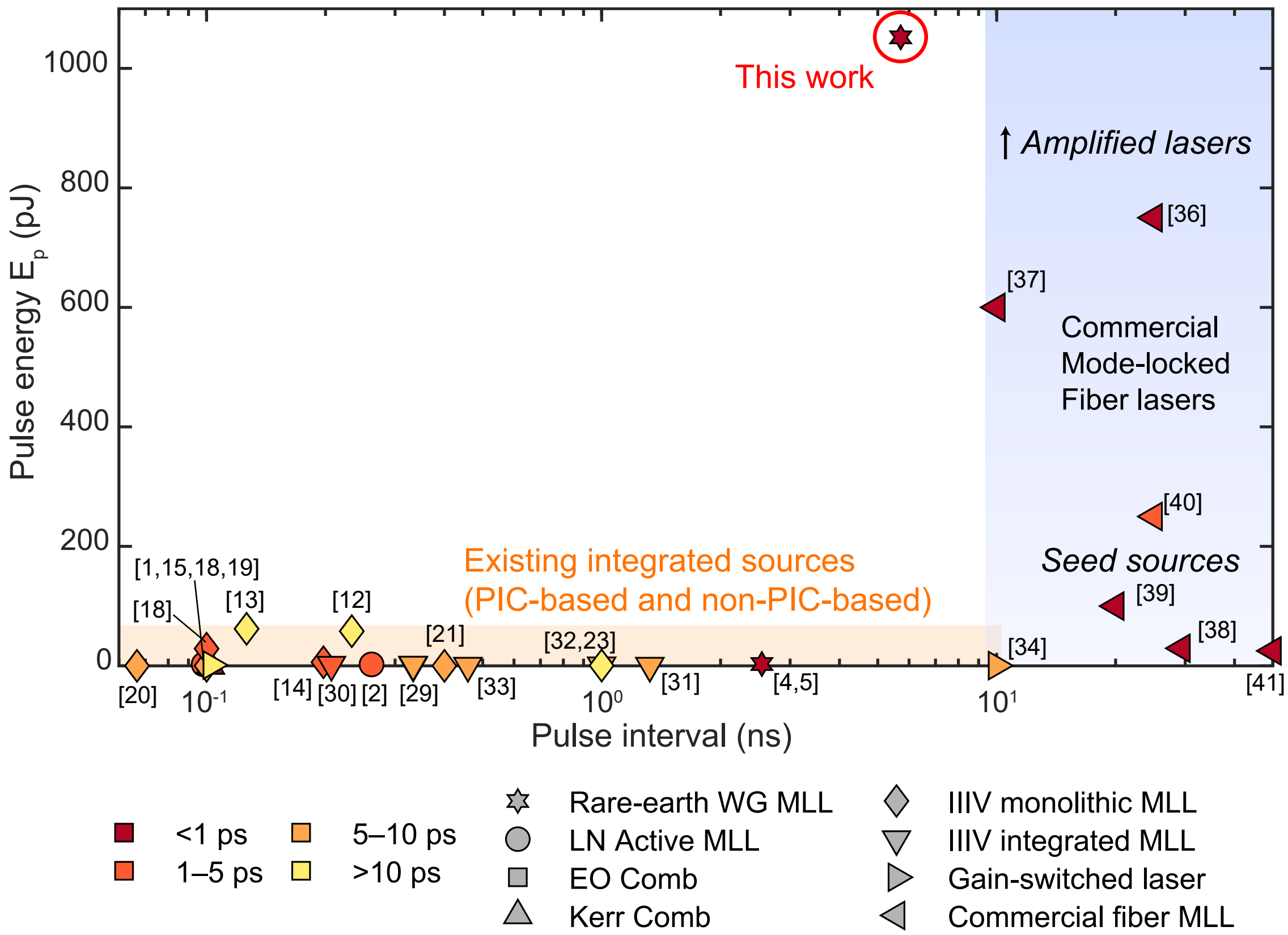

Figure S1. Comparison with state-of-the-art integrated ultrafast pulse sources (and a few commercial lasers) in terms of pulse energy E_p and pulse interval ($1/f_\mathrm{rep}$). The color of the markers represents the pulse width.

Table S1: Comparison with state-of-the-art integrated ultrafast pulse sources and a sample of state-of-the-art lasers.

Year	λ_c (μm)	f_{rep} (GHz)	Pulse width (ps)	(On-chip) average power (mW)	E_p (pJ)	f_rep RF linewidth (Hz)	Comb line linewidth (kHz)	Spectrum width (nm)	Type	Reference
2026	1.56	0.175	0.147[a]	184	1052	1.2×10^{-5}	31.4	64 (20 dB)	Er-doped-Si_3N_4 Mamyshev	**This work**
Integrated ultrafast pulse sources										
2023	1.06	10.17	4.81	53	2.6	x	3.91×10^3	0.35 (3 dB)	LN active MLL	[1]
1995	1.60	3.823	3.8	0.9	2.4	x	–	0.95 (3 dB)	LN active MLL	[2]
2024	1.55	39.58	∼20	11	0.28	x	0.6[v]	20	LN active MLL	[3]
2009	1.56	0.393	0.44	1.2[o]	3.05	+	-	8.4 (3 dB)	Er-glass WG MLL (SESAM)	[4, 5]
2019	1.90	1.2	-	9	-	-	-	17 (3 dB)	Tm:Al_2O_3 passive MLL	[6]
2022	1.56	30.10	0.52	16.25	0.54	x	x	6 (10 dB)	EO comb (LN, time-lens)	[7]
2022	1.55	30.925	0.336	20	0.65	x	x	132 (−70 dBm)	EO comb (LN, resonant)	[8]

Table S1 (continued)

Year	λ_c (μm)	f_{rep} (GHz)	Pulse width (ps)	(On-chip) average power (mW)	E_p (pJ)	f_{rep} RF linewidth (Hz)	Comb line linewidth (kHz)	Spectrum width (nm)	Type	Reference
2020	1.56	9.78	0.098	0.11^{c}	0.011^{c}	x	x	25.8 (3 dB)	Kerr comb (SiN)	[9]
2023	1.55	99.72	++	∼6	∼0.06	x	x	–	Kerr comb (SiN)	[10]
2022	1.55	143.6	++	20	0.14	x	0.5	200 (60 dB)	Kerr comb (hybrid SiN)	[11]
2006	1.55	4.29	10	250	58	-	-	5.7 (3 dB)	III–V monolithic (SCOWL)	[12]
2007	0.98	7.92	16	489	62	-	-	0.96 (3 dB)	III–V monolithic (SCOWL)	[13]
2005	1.26	5.06	3.2	<30	<6	-	-	7.1 (3 dB)	III–V monolithic (QD)	[14]
2011	1.51	10.0	6	0.8^{o}	0.08^{o}	1×10^{2e}	-	1.5	III–V monolithic (QD)	[15]
2005	1.26	21.0	0.39	25	0.95	-	-	20	III–V monolithic (QD)	[16]
2008	1.54	92.0	0.312	8.6	0.09	-	-	11.62 (3 dB)	III–V monolithic (QD)	[17]
2012	1.26	10.0	2.2	288	28.7	-	-	5.6 (3 dB)	III–V monolithic (Tapered)	[18]
2025	1.53	10.6	0.726	-	-	-	-	19.22 (20 dB)	III–V monolithic (QW)	[19]
2006	1.53	15.0	5	∼0.15	0.01	4×10^5	6×10^5	4.5 (3 dB)	III–V monolithic (Ring)	[20]
2015	1.58	2.5	9.8	0.08	0.032	6.13×10^3	-	3 (3 dB)	III–V monolithic (InP PIC)	[21]
2017	1.53	21.5	0.35	1	0.047	4.5×10^5	-	14 (3 dB)	III–V monolithic (InP PIC)	[22]
2010	1.58	1.0	36	>0.59	0.59	-	7×10^4	5 (10 dB)	III–V monolithic (QW)	[23]
2021	1.55	0.99	272	-	-	7×10^4	-	-	III–V monolithic	[24]
2019	1.31	20.0	1.7	0.48	0.024	400	-	5.5 (3 dB)	III–V monolithic (on Si)	[25]
2019	1.27	20.0	5	<18	<0.9	1.8×10^{3i}	10.6×10^{3v}	8.4 (10 dB)	III–V monolithic (on Si)	[26]
2024	1.55	3.0	-	2	0.67	-	-	23 (10 dB)	III–V integrated (het. SiN)	[27]
2018	1.58	20.0	0.9	1.83	0.09	1.1×10^3	-	∼20 (20 dB)	III–V integrated (het. Si)	[28]
2021	1.61	3.0	8	5.6	2	4×10^2	$< 10^3$	4 (10 dB)	III–V integrated (het. SiN)	[29]
2015	1.56	4.83	3	9	1.86	1.7×10^3	$< 2 \times 10^3$	3.5 (10 dB)	III–V integrated (het. Si)	[30]
2021	1.58	0.755	7.46	0.125	0.17	1	146	3.27 (10 dB)	III–V integrated (het. SiN)	[31]
2017	1.60	1.0	7	<0.8	<0.8	9×10^2	2.5×10^2	17 (10 dB)	III–V integrated (het. Si)	[32]
2021	1.56	2.18	6.31	0.4^{o}	0.18	31	-	8.3 (10 dB)	III–V integrated (hyb. SiN)	[33]
2025	1.53	3.03	4.5	12.5	4.2	1.4×10^3	-	10 (10 dB)	III–V integrated (het. SiN)	[34]
2023	1.27	0.1	5.3	0.042	0.42	-	-	0.4 (3 dB)	Gain-switched DFB laser	[35]
2021	1.55	9.72	-	13	1.34	-	-	-	Gain-switched laser	[36]
Commercial mode-locked lasers										
2025	1.55	0.04	<0.3	30	750	-	-	20	Fiber-based MLL (w/ amp.)	[37]
2025	1.56	0.1	0.1	60	600	3×10^{-8p}	$< 0.12^{p}$	30	Fiber-based MLL (w/ amp.)	[38]
2025	1.50	0.034	0.38	1	29.4	-	-	20	Fiber-based MLL (w/ amp.)	[39]
2025	1.95	0.05	0.35	5	100	-	-	10	Fiber-based MLL (w/o amp.)	[40]
2025	1.03	0.04	3	10	250	3×10^{-10e}	-	14	Fiber-based MLL (w/o amp.)	[41]
2025	1.55	0.02	0.5	0.5	25	-	-	-	Fiber-based MLL (w/o amp.)	[42]
Lab demonstration of fiber-based mode-locked lasers										
2017	1.03	0.017	0.04	850	5×10^4	-	-	110	Fiber Mamyshev oscillator	[43]
2019	1.05	0.008	0.041	9000	1.1×10^6	-	-	127 (20 dB)	Fiber Mamyshev oscillator	[44]
2022	1.05	0.014	0.058	17000	1.2×10^6	30^{i}	-	76 (10 dB)	Fiber Mamyshev oscillator	[45]
2017	1.03	0.1	0.098	10	100	-	-	43 (3 dB)	figure-9 fiber laser	[46]
2017	1.55	0.25	0.072	3	12	-	-	23 (3 dB)	figure-9 fiber laser	[46]
2017	2.05	0.01	0.24	4.6	460	-	-	24 (3 dB)	figure-9 fiber laser	[46]

Notes:

QD: Quantum dot; QW: Quantum well; SCOWL: Slab-coupled optical waveguide laser; SESAM: Semiconductor saturable absorber mirrors; LN:$LiNbO_3$; het.: heterogeneous; hyb.: hybrid.

x Not applicable (for example, when it directly depends on the driving source).

- No data provided.

+ No data provided, but likely good as the integrated jitter (26 fs from 10 kHz to 10 MHz) is low.

++ No data provided, but likely very short due to the solitonic nature and wide spectrum.

[a] Autocorrelation function width after linear compression in fiber.

[c] Estimated from the conversion efficiency.

[p] Experimentally measured at EPFL for an older unit produced in 2016.

[e] Estimated from figures on datasheet.

[o] Off-chip coupled power or unspecified. No coupling efficiency reported.

[v] Average optical linewidth of each mode from delayed self-heterodyne method.

[i] Integrated full width at half maximum linewidth.

S2. Simulation of integrated Mamyshev oscillator and design guidelines

A. Method

Pulse propagation in the Mamyshev oscillator cavity is modeled using the generalized nonlinear Schrödinger equation (GNLSE):

$$\frac{\partial A}{\partial z} = -\frac{i\beta_2}{2}\frac{\partial^2 A}{\partial t^2} + \frac{g-\alpha}{2}A + i\gamma|A|^2 A, \tag{1}$$

where $A(z,t)$ is the complex envelope of the intracavity optical field, β_2 is the group velocity dispersion for the waveguide, γ is the waveguide Kerr nonlinear coefficient detailed later, $g(z)$ is the position-dependent optical gain, and α is the linear optical loss. Pulse propagation is performed sequentially in the forward and backward directions using the split-step Fourier method [47] or higher-order algorithms, assuming negligible nonlinear interaction between counter-propagating pulses. Due to the short physical length of the gratings, the reflections from the gratings are modeled as an instantaneous linear process. Accurate modeling of $g(z)$ requires additional effort and we adopt a first-principles static model instead of the gain saturation model commonly used in the literature. The latter usually assumes a fixed small-signal gain g_0 and a constant saturation power P_{sat}, with the gain given by $g = \frac{g_0}{1+P/P_{\mathrm{sat}}}$. In erbium-doped waveguides with sub-meter-scale lengths and high doping levels, pump power can decay significantly along the propagation direction, which makes the assumption of a constant P_{sat} inaccurate.[1] To address this, we aim to model the non-constant population inversion $n(z)$ and gain $g(z)$ for each point during the pulse and pump propagation, using the effective two-level system rate equations [48, 49]:

$$\begin{aligned}\frac{\mathrm{d}N_2}{\mathrm{d}t} &= -\frac{N_2}{\tau} + \left(N_1\sigma_{12\mathrm{s}} - N_2\sigma_{21\mathrm{s}}\right)\phi_{\mathrm{s}} - \left(N_2\sigma_{21\mathrm{p}} - N_1\sigma_{12\mathrm{p}}\right)\phi_{\mathrm{p}} \\ \frac{\mathrm{d}N_1}{\mathrm{d}t} &= \frac{N_2}{\tau} + \left(N_2\sigma_{21\mathrm{s}} - N_1\sigma_{12\mathrm{s}}\right)\phi_{\mathrm{s}} - \left(N_1\sigma_{12\mathrm{p}} - N_2\sigma_{21\mathrm{p}}\right)\phi_{\mathrm{p}}.\end{aligned} \tag{2}$$

Under the assumption that the population inversion has reached equilibrium ($\mathrm{d}N_{1,2}/\mathrm{d}t = 0$) and the inversion is uniform across the waveguide cross-section, the normalized population inversion $n(z) = N_2/N_0'$ becomes

$$n = N_2/N_0' = \frac{\tau\frac{\sigma_{12\mathrm{s}}}{h\nu_{\mathrm{s}}}I_{\mathrm{s}} + \tau\frac{\sigma_{12\mathrm{p}}}{h\nu_{\mathrm{p}}}I_{\mathrm{p}}}{\tau\frac{\sigma_{12\mathrm{s}}+\sigma_{21\mathrm{s}}}{h\nu_{\mathrm{s}}}I_{\mathrm{s}} + \tau\frac{\sigma_{12\mathrm{p}}+\sigma_{21\mathrm{p}}}{h\nu_{\mathrm{p}}}I_{\mathrm{p}} + 1}. \tag{3}$$

The resulting gain coefficient $g(z)$ and pump power evolution are:

$$\begin{aligned} g(z) &= \left[n(z)\big(1-n_{\mathrm{cluster}}\big)\sigma_{21\mathrm{s}} - \Big(\big(1-n(z)\big)\big(1-n_{\mathrm{cluster}}\big) + n_{\mathrm{cluster}}\Big)\sigma_{12\mathrm{s}}\right] N_0\Gamma\frac{n_g}{n_A}, \\ \frac{\mathrm{d}I_{\mathrm{p,prop}}(z)}{\mathrm{d}z} &= \left[n(z)\big(1-n_{\mathrm{cluster}}\big)\sigma_{21\mathrm{p}} - \Big(\big(1-n(z)\big)\big(1-n_{\mathrm{cluster}}\big) + n_{\mathrm{cluster}}\Big)\sigma_{12\mathrm{p}}\right] N_0\Gamma\frac{n_g}{n_A} I_{\mathrm{p,prop}}(z) - \alpha I_{\mathrm{p,prop}}(z). \end{aligned} \tag{4}$$

where the definitions of the parameters in Eqs. (2)-(4) can be found below:

- $\sigma_{12\mathrm{s}}$ and $\sigma_{12\mathrm{p}}$: absorption cross sections for signal and pump.
- $\sigma_{21\mathrm{s}}$ and $\sigma_{21\mathrm{p}}$: emission cross sections for signal and pump.
- ϕ_{s}, ϕ_{p}: photon fluxes for signal and pump (sum of both forward and backward propagating light).
- I_{s} and I_{p}: optical intensities of signal and pump (sum of both forward and backward propagating light).
- α: power loss coefficient in either forward or backward propagation, assumed to be the same for pump and signal.

[1] In some literature on erbium-doped fibers or waveguides, P_{sat} is presented as a constant independent of pump power, and typically expressed as inversely proportional to the upper-level lifetime τ and the emission (or absorption) cross-section σ. However, this is incorrect for systems where the optical pumping rate greatly exceeds the spontaneous decay rate, as can be seen by analyzing the rate equations Eq. (2). For erbium-doped waveguides, while the spontaneous decay rate is on the order of 10^2 s^{-1} due to the millisecond-scale lifetime, the optical pumping rate can reach 10^5 s^{-1} for hundreds of milliwatts of pump power. When the waveguide functions as a laser cavity with signal average power exceeding several milliwatts (as in continuous-wave or high-repetition-rate pulsed systems), the stimulated emission rate can similarly exceed the spontaneous decay rate by orders of magnitude, making τ irrelevant.

- g: power gain coefficient for signal in either forward or backward propagation.
- $I_{\mathrm{p,prop}}$: optical intensity of the pump in either forward ($I_{\mathrm{p,fwd}}$) or backward ($I_{\mathrm{p,bwd}}$) propagation.
- τ: spontaneous emission lifetime.
- $h\nu_{\mathrm{s}}$, $h\nu_{\mathrm{p}}$: photon energies of signal and pump.
- N_0: peak erbium ion concentration.
- Γ: overlap factor between the erbium ion distribution and optical mode intensity, normalized to the peak concentration.
- $N_1(z)$, $N_2(z)$: ground- and excited-state population densities (peak of the profile).
- $N_0' = N_1(z) + N_2(z)$: total active erbium concentration (peak of the profile).
- $n_{\mathrm{cluster}} = 1 - N_0'/N_0$: fraction of erbium ions in ion clusters that cannot be excited, while still absorbing.
- n_g/n_A: correction factor of the gain in high-index-contrast waveguides [50].

In the gain simulation, we assumed a uniform excitation ratio in the cross-section of the waveguide, which is a good approximation for high pump and signal intensity. We considered a simplified phenomenological quenching model similar to the pair-induced quenching in [51] due to the relatively high erbium doping concentration (up to 0.5 atomic percent), where some ions are considered to be in "clusters" that absorb while having negligible radiative emission efficiency. We did not include the uniform up-conversion processes [52], as these effects are found to be negligible in our strongly pumped system. This conclusion is based on parameters extracted from separate photoluminescence decay measurements in implanted short waveguide samples. The emission and absorption cross-sections, as well as the fraction of clustered ions, are empirically determined by fitting the model to measured amplifier gain data.

Algorithm 1: Steady state Mamyshev oscillator simulation

```
Input: Guess of the intracavity field: A(ν), pump power at the boundaries: P_{0,fwd}, P_{0,bwd}, complex reflectivity of the
       two filters: R_1(ν) and R_2(ν), and other waveguide properties used in propagation.
Create initial guesses as functions of spatial coordinate z for average power of pulse propagating backward P_{avg,bwd,1}(z),
 and pump power propagating backward P_{p,bwd,1}(z) with piecewise linear functions;
for j ← 1 to M do
    // Pseudo-"time stepping" for convergence of spectrum
    for k ← 1 to N do
        // Self-consistent iteration for all the optical powers
        Reset A(ν,0) to the A'(ν,0) obtained from the last "time-stepping";
        for m ← 1 to l/dz do
            // Forward propagation.
            Current position z ← m × dz;
            Compute average signal power at the past position P_{avg,fwd,k}(z − dz) using the electric field A(ν, z − dz);
            Compute population inversion n(z) using P_{avg,fwd,k}(z − dz) + P_{avg,bwd,k−1}(z) and
             P_{p,fwd,k}(z − dz) + P_{p,bwd,k−1}(z) with Eq. (3);
            Compute the gain g(z) using n(z) with Eq. (4);
            Compute the signal electric field A(ν, z) from A(ν, z − dz) and g(z) using split-step Fourier method;
            Compute the pump power P_{p,fwd,k}(z) from P_{p,fwd,k}(z − dz) using n(z) and propagation equation Eq. (4);
        // Apply filter 1.
        A(ν, l) ← A(ν, l) × R_2(ν);
        for m ← 1 to l/dz do
            // Backward propagation.
            Current position z ← l − m × dz;
            Compute average signal power at the past position P_{avg,bwd,k}(z + dz) using the electric field A(ν, z + dz);
            Compute population inversion n(z) using P_{avg,bwd,k}(z + dz) + P_{avg,fwd,k}(z + dz) and
             P_{p,fwd,k}(z + dz) + P_{p,bwd,k}(z + dz) with Eq. (3);
            Compute the gain g(z) using n(z) with Eq. (4);
            Compute the signal electric field A(ν, z) from A(ν, z + dz) and g(z) using split-step Fourier method;
            Compute the pump power P_{p,bwd,k}(z) from P_{p,fwd,k}(z − dz) using n(z) and propagation equation Eq. (4);
        if |P_{avg,bwd,k}(0) − P_{avg,bwd,k−1}(0)| < tol or k = N then
            // Self-consistent iterations converged or exhausted.
            // Apply filter 2 and prepare for the next "time-stepping" from z = 0.
            A'(ν, 0) ← A(ν, 0) × R_2(ν);
            Break;
Postprocess the computed intra-cavity field A'(ν, 0) to obtain output field and performance estimations;
```

Due to the linear cavity design and bidirectional pumping, both signal and pump propagate in forward and backward

directions. Their intensities modify the local population inversion, which in turn affects the gain and absorption experienced during pulse and pump propagation. Thus, forward and backward propagation and the propagation of pulse and pump are interdependent and must be solved in a self-consistent manner.

In this work, we searched for steady-state solutions of the system using Algorithm 1, where the average powers are assumed to be time-independent and the average powers obtained from pulse propagation (accounting for gain and loss) are consistent with those used in the gain calculation. This avoids simulating the slow population dynamics (microsecond timescale) over many cavity round-trips (nanosecond timescale), which would otherwise be computationally expensive. For each propagation step for position $z \to z + dz$, we compute the steady-state population inversion $n = N_2/N_0'$ using Eq. (3) and the intensities I_s and I_p as detailed in the Algorithm 1. Then we compute $g(z)$ using Eq. (4) and proceed with the split-step method for the signal and the scalar propagation of the pump. The average signal and pump powers in both directions are stored as guesses for the following reversed propagation step. This bidirectional propagation loop is repeated iteratively until the forward and backward power profiles converge. Once convergence is achieved, the updated intracavity field A is used as the input for the next outer iteration ("pseudo-time step") to refine the pulse shape and spectrum. The overall process continues until a given number of outer loops is reached.

The algorithm typically converges within tens of outer iterations across most of the parameter space, either settling into a stable mode-locked state or collapsing to a trivial solution dominated by continuous-wave lasing from parasitic reflections. In some cases, the algorithm fails to converge, which we attribute to chaotic pulse dynamics, which have also been occasionally observed experimentally as unstable or chaotic pulsing behavior under specific pump powers and grating separations.

The `MATLAB`-based simulation code is provided in the supplementary Zenodo repository accompanying the manuscript (https://doi.org/10.5281/zenodo.18732611).

B. Parameters

To estimate the Kerr nonlinear coefficient γ of our Si_3N_4 waveguide, we use the definition:

$$\gamma = \frac{2\pi}{\lambda}\frac{n_2}{A_\mathrm{eff}}, \tag{5}$$

where $\lambda = 1.55\ \mu$m and $n_2 = 2.2 \times 10^{-19}\,\mathrm{m^2/W}$ is the Kerr nonlinear index of Si_3N_4 [53]. We assume that the nonlinear index modification caused by the ion implantation is negligible. The nonlinear contribution from the SiO_2 cladding is neglected due to the significantly lower n_2 in SiO_2 and the confinement of the optical mode. A_eff is the effective mode area of the waveguide, which can be calculated using a fully vectorial model [54]:

$$A_\mathrm{eff} = \frac{\mu_0}{\varepsilon_0}\frac{3\left|\int_\infty (\mathbf{E}\times\mathbf{H}^*)\cdot \mathrm{d}\boldsymbol{S}\right|^2}{n^2_{\mathrm{Si_3N_4}}\int_{\mathrm{Si_3N_4}}\left[2|\mathbf{E}|^4 + |\mathbf{E}^2|^2\right]\mathrm{d}S}, \tag{6}$$

where $\mathbf{E}$ and $\mathbf{H}$ are the modal electric and magnetic fields derived from finite element simulation with `COMSOL`, $n_{\mathrm{Si_3N_4}}$ is the refractive index of Si_3N_4 and $\mathrm{d}\boldsymbol{S}$ is the vectorial surface element of the cross-section. Figure S2 shows the calculated values of the nonlinear coefficient γ for the fundamental transverse electric (TE) mode across different waveguide geometries, as this mode typically exhibits the strongest confinement and highest nonlinearity. In this work, we selected a Si_3N_4 waveguide thickness of 350 nm as a compromise between achieving high nonlinearity and ensuring compatibility with cost-effective ion implantation using air-insulated electrostatic accelerators.

The waveguide used here has simulated dispersion coefficients of $\beta_2 = 715\ \mathrm{ps^2/km}$ (normal group velocity dispersion (GVD)) and $\beta_3 = -1.81\ \mathrm{ps^3/km}$. For the cavity geometry used in the experiments, the corresponding total round-trip group-delay dispersion is 0.601 $\mathrm{ps^2}$. Dispersion in the Si_3N_4 waveguides is primarily determined by the waveguide geometry. In our fabrication process, the Si_3N_4 layer thickness is monitored by optical interferometry and the waveguide width is defined lithographically and verified by scanning electron microscopy. Typical dimensional tolerances are approximately ± 5 nm in thickness and ± 20 nm in width, which correspond to an estimated GVD uncertainty bound at the level of approximately 6%. In future developments, commercial foundry fabrication with statistical process control is expected to further improve dispersion reproducibility across wafers.

The complex reflectivity $R_1(\nu)$ and $R_2(\nu)$, as well as the transmissivity of the waveguide Bragg gratings (WBGs), are simulated using the transfer matrix method (TMM) [55]. The effective index modulation Δn and parasitic chirping $\Delta l/l$ caused by the bandgap center shift due to apodization [56], are extracted from experimentally measured grating spectra fabricated during calibration runs. We note that the experimentally extracted Δn in our gratings can be lower than the numerical predictions from either cross-section effective index simulations or photonic-bandgap

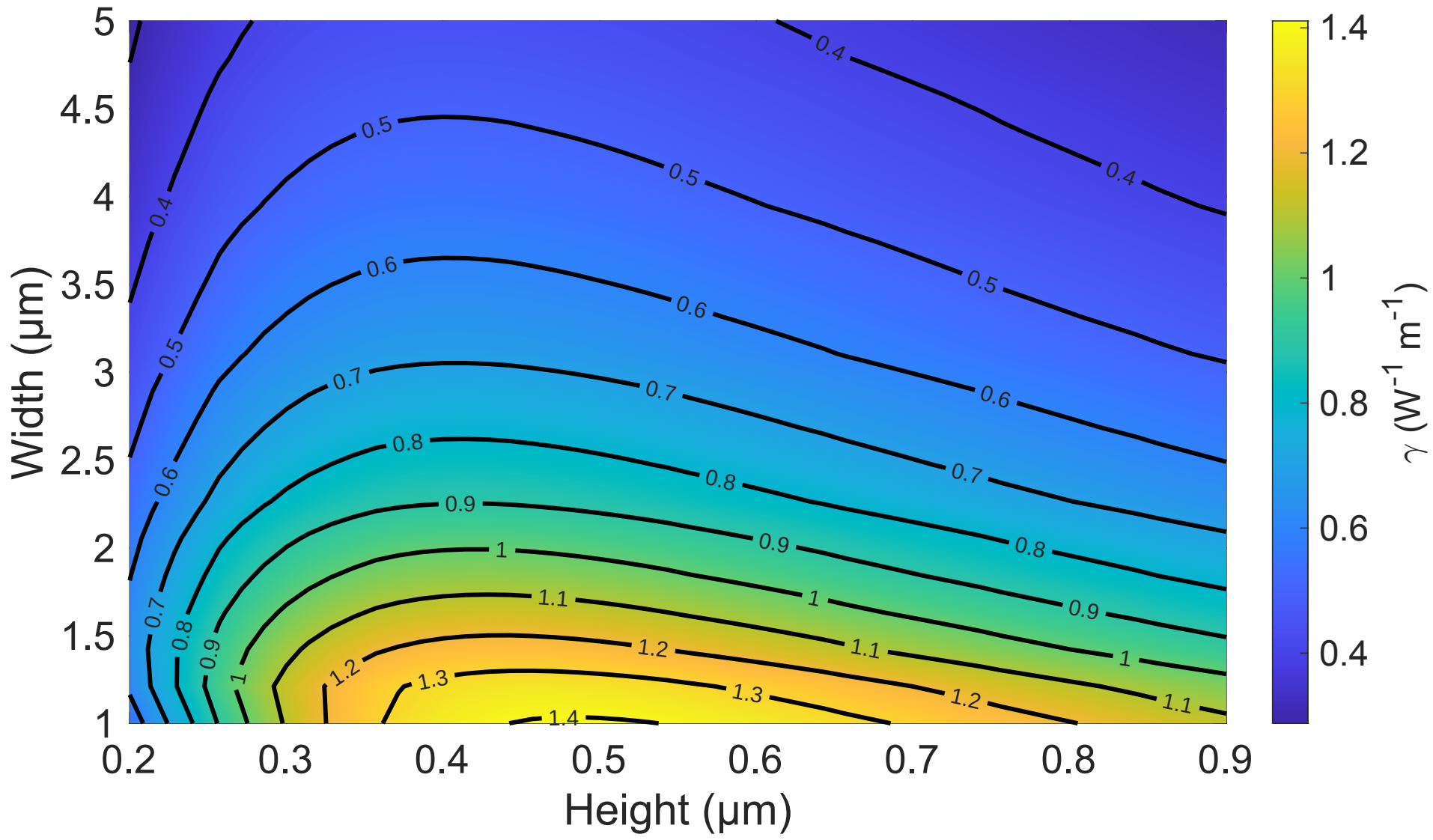

Figure S2. Computed Kerr nonlinear coefficient γ as a function of the width and height of a Si_3N_4 waveguide at 1.55 µm wavelength.

simulations [56], even after compensating for the finite lithographic resolution. The reason for this discrepancy is currently unknown, and may arise from the breakdown of the perturbative approximation for the strongly modulated gratings used here. Nevertheless, we find that TMM provides a good approximation of the grating spectrum after using the experimentally calibrated Δn.

Mamyshev oscillator MLLs are particularly sensitive to parasitic backreflections, which can lead to undesirable continuous-wave lasing in the cavity, reduced round-trip gain, and eventually failure to reach stable mode-locking. To model wideband Fresnel backreflections at the chip facets, which represent the dominant source of reflections, we introduce a correction term to the grating reflectivity: $R_i' = R_i + \sqrt{R_{\text{parasitic}}}(1-|R_i|^2)\exp(\text{i}\varphi)/[1+R_i\sqrt{R_{\text{parasitic}}}\exp(\text{i}\varphi)]$, where $R_{\text{parasitic}}$ is the measured power backreflection ratio, and φ is the frequency-dependent, round-trip propagation phase between the grating and facet.

The typical parameters for the integrated Mamyshev MLL are listed in Table S2. These parameters are used to produce the pulse propagation simulation shown in Fig. 1(c) of the main text. The silicon nitride waveguide is assumed to have a refractive index of 1.98 at the wavelengths of interest.

Table S2: Parameters used in the integrated Mamyshev MLL simulation

Symbol	Variable name	Description	Value
λ_s		Simulation center wavelength	1550 nm
λ_p		Pump wavelength	1480 nm
l	`l`	Length of doped waveguide	0.42 m
n_g	`ng`	Waveguide group refractive index	2.0023
β_2	`beta2`	Group-velocity dispersion	$0.715\ \text{ps}^2/\text{m}$
β_3	`beta3`	Third-order dispersion	$-1.81\times10^{-3}\ \text{ps}^3/\text{m}$
γ	`gamma_nl`	Kerr nonlinear coefficient	$1.145\ \text{W}^{-1}\,\text{m}^{-1}$
α	`alpha_loss`	Propagation loss	$10\ \text{dB/m} = 2.3\ \text{m}^{-1}$
n_{cluster}	`clusteringFrac`	Fraction of clustered ions	0.06
Γ	`overlap`	Mode-gain overlap	0.22
N_0	`N0`	Peak erbium ion concentration	$3.68\times10^{26}\ \text{m}^{-3}$
A_s, A_p	`As`, `Ap`	Effective mode area (signal, pump)	$1.5\times10^{-12}\ \text{m}^2$
τ	`tau`	Upper-state lifetime	$3.0\times10^{-3}\ \text{s}$
$\sigma_{12\text{p}}$, $\sigma_{12\text{s}}$	`s12p`, `s12s`	Absorption cross-sections (pump, signal)	$3.43\times10^{-25}\ \text{m}^2$; $2.53\times10^{-25}\ \text{m}^2$
$\sigma_{21\text{p}}$, $\sigma_{21\text{s}}$	`s21p`, `s21s`	Emission cross-sections (pump, signal)	$5.9\times10^{-26}\ \text{m}^2$; $4.48\times10^{-25}\ \text{m}^2$
$\Delta\lambda_g$	`delta_lambda_g`	Gain bandwidth	40 nm
$P_{\text{p,fwd}}$	`pump_power_fwd`	On-chip forward pump power	450 mW
$P_{\text{p,bwd}}$	`pump_power_bwd`	On-chip backward pump power	390 mW
$\Delta\lambda_{\text{f}}$	`filter_gap`	Offset between filters	8.9 nm
$R_{\text{parasitic}}$	`br`	Power back-reflection ratio	1.0×10^{-3}

Table S2 (continued)

Symbol	Variable name	Description	Value
N	`NN`	Number of grating periods	950
Δn	`dn`	Peak-to-peak effective index modulation	8.6×10^{-3}
$\Delta l/l$	`parasitic_chirping`	Parasitic chirping due to index change	1.2×10^{-3}
σ_{apo}	`apodization_sigma`	Standard deviation of the Gaussian apodization	0.15

C. Simulation results

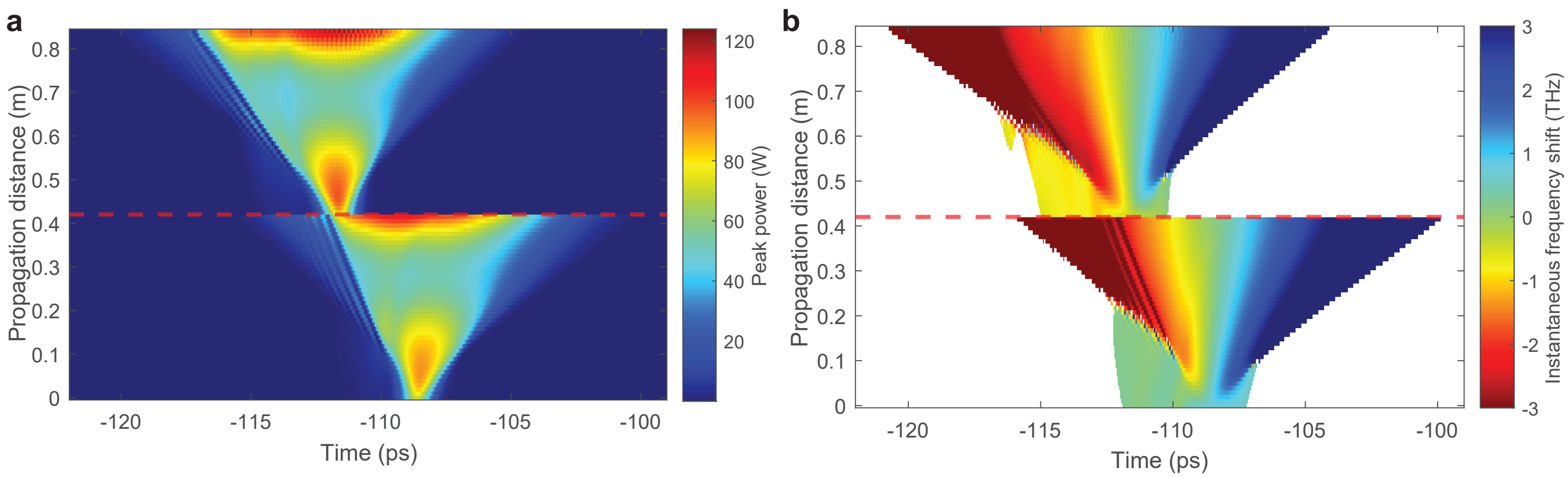

Figure S3. **Pulse propagation in the time domain. a,** Time-domain intensity of the pulse as a function of propagation length within the cavity. The red dashed line indicates the transition from forward to backward propagation after filtering. **b,** Instantaneous frequency of the pulse as a function of propagation distance. The white area indicates where the pulse intensity is less than 0.1% of the peak and the chirping is irrelevant.

For reference, we define Grating 1 as the red-shifted WBG and Grating 2 as the blue-shifted one. "Forward propagation" refers to the direction from Grating 1 to Grating 2, while "backward propagation" is from Grating 2 to Grating 1. All reported powers in the simulation correspond to on-chip values.

Figure S3 shows the simulated time-domain evolution of the pulse as it propagates through the cavity. The pulse acquires a linear chirp and broadens significantly to several picoseconds due to the strong normal GVD of the waveguide, which disperses the newly generated frequency components. The intracavity peak power remains within 50 W to 125 W throughout the round-trip, allowing for substantial self-phase modulation (SPM) without inducing pulse instability or damage to the waveguide.

Figure S4 shows the time-domain envelope and instantaneous frequency at various points in the cavity, including the reflected and transmitted pulses at the WBGs. The simulated output pulses from both gratings feature pulse widths of a few picoseconds and clearly exhibit a linear frequency chirp. This indicates that the output pulses can be significantly compressed by group delay dispersion compensation. Figure S5 presents the simulated optical spectra of the laser outputs along with the modeled WBG reflectance. While the details of the spectra differ from the measured ones, the simulations reproduced key features such as spectral valleys and fine oscillations on one side of the output.

Figure S6 presents the pulse after optimal group delay dispersion compensation. Sub-150 fs compressed pulses are achieved from both output directions, with peak powers at the kilowatt level.

The accumulated nonlinear phase shift during a round-trip is semi-quantitatively characterized using the B-integral $B = \int_0^{2l} \gamma I_{\text{s,prop}}(z)\,\mathrm{d}z$ [57]. Figure S7 shows the B-integral as a function of propagation length. The maximum B-integral reaches approximately 20π per round-trip, which is well beyond the regime accessible in soliton-based mode-locking [43, 57].

Figure S8 shows the evolution of pulse energy during propagation. The forward pass and the backward pass are plotted separately, and the abrupt drop in energy represents output coupling from the cavity. One notes that the pulse energy increases at both the beginning and the end of the cavity, while slightly decreasing in the middle section. We believe this is due to the rapid depletion of the pump power at both ends of the waveguide, leading to insufficient population inversion at the center. In future developments, a more uniform intra-cavity gain profile may be achieved by decreasing the erbium doping concentration or engineered selective doping. The fractions of incident power reflected by the WBGs are 8.3% and 9.6% for the WBG before the forward pass and that after the forward pass, respectively.

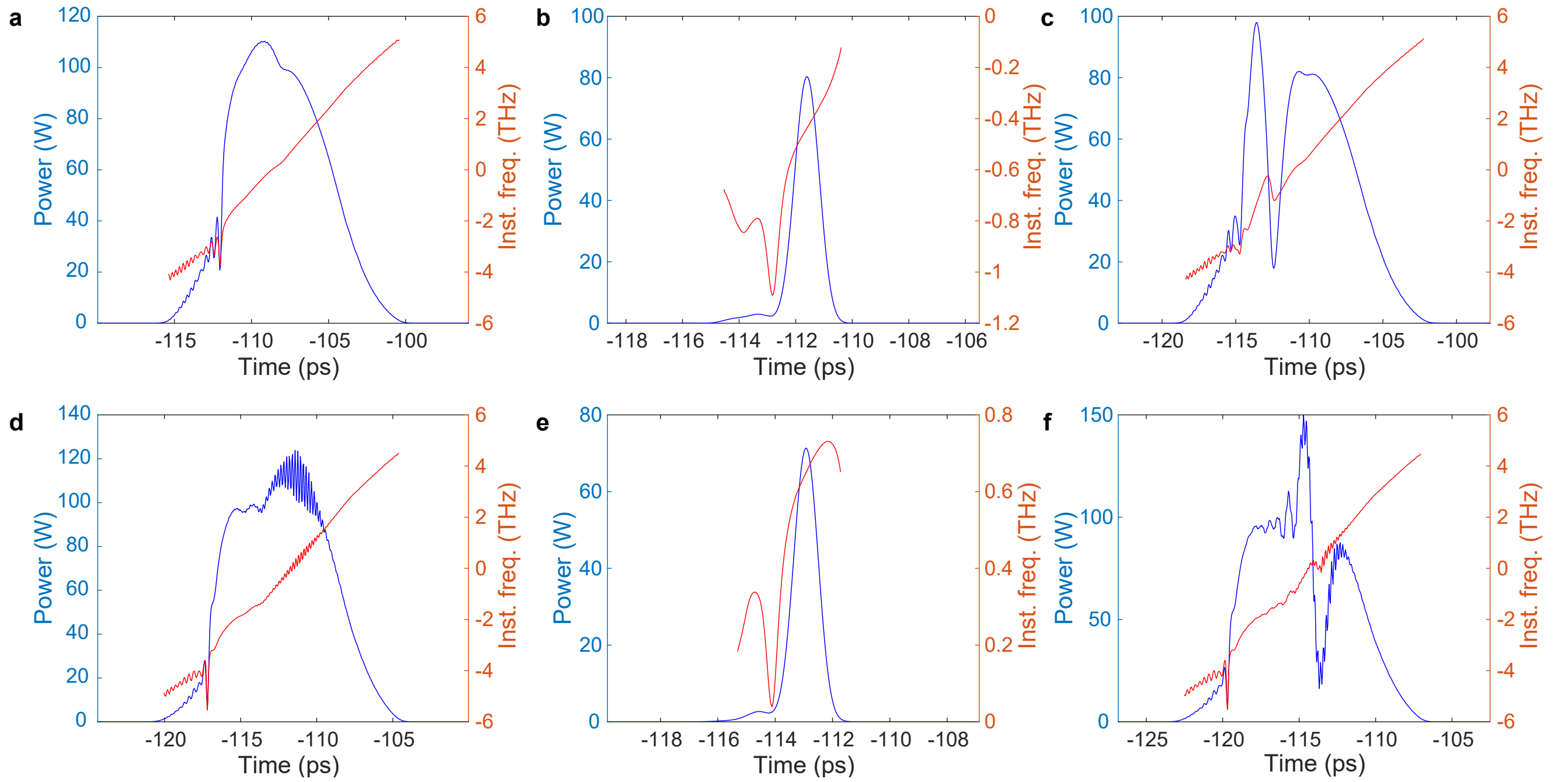

Figure S4. **Pulse waveforms and chirp at key locations.** **a,** Pulse after forward propagation, before WBG reflection. **b,** Pulse after forward propagation, immediately after reflection. **c,** Output pulse transmitted through WBG after forward propagation. **d-f,** Same as **a-c**, but for backward propagation.

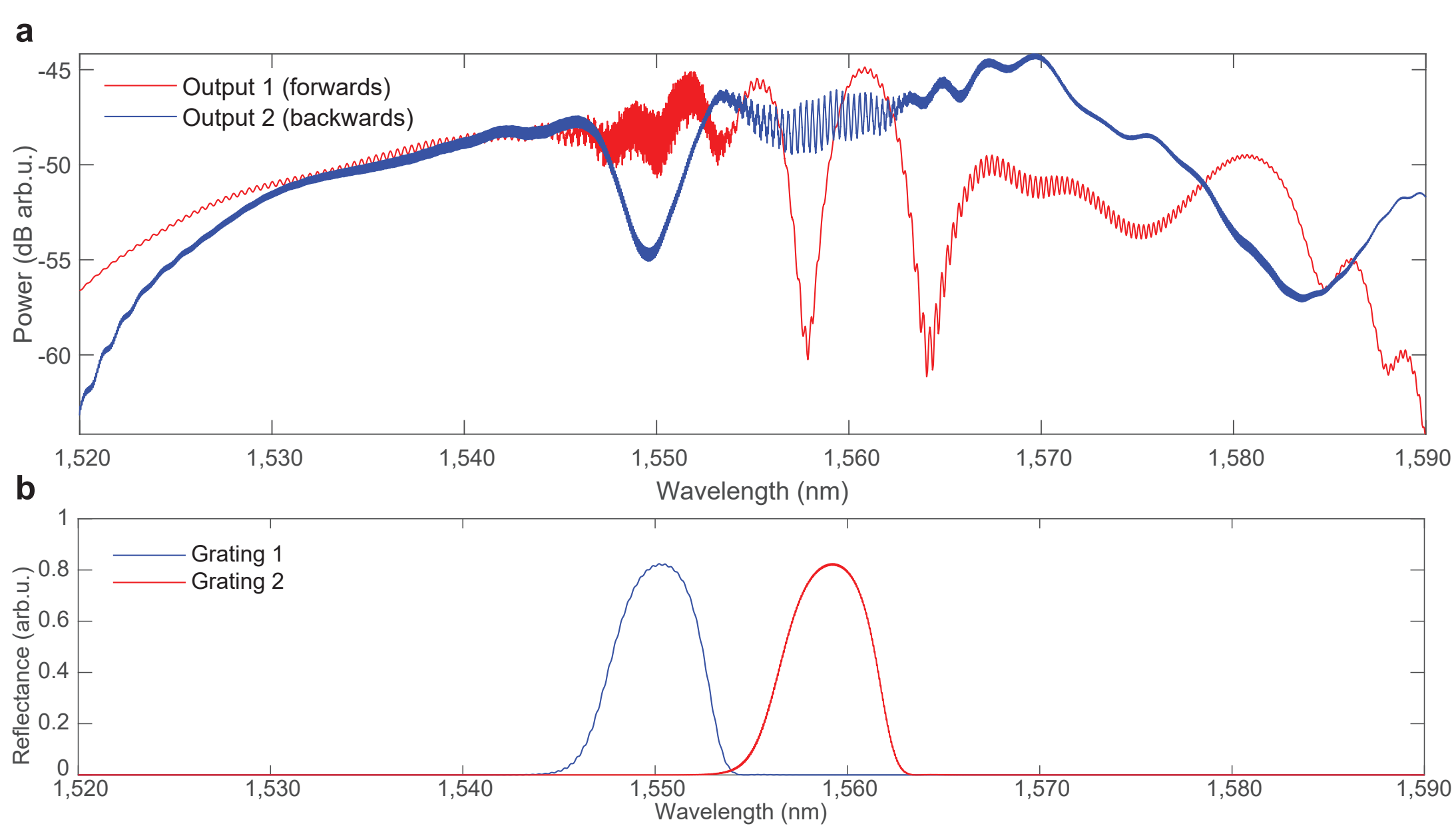

Figure S5. **Simulated frequency-domain results.** **a,** Output spectra of the Mamyshev oscillator. **b,** Reflectance spectra of the WBGs including modeled facet parasitic reflections.

Figure S9 shows the simulated intracavity and dispersion compensated output peak power of the stable solution as a function of two key design parameters: the grating center wavelength offset $\Delta\lambda_f$ and the cavity length l. A wide parameter range supports stable mode-locking, with WBG separations ranging from 7 nm to 20 nm and cavity lengths from 15 cm to 75 cm. However, for cavity lengths exceeding 57 cm combined with relatively large grating separations, the algorithm often fails to converge to a stable solution. These regions are suspected to correspond to chaotic states where the pulse becomes unstable, possibly due to excessive nonlinear phase accumulation within

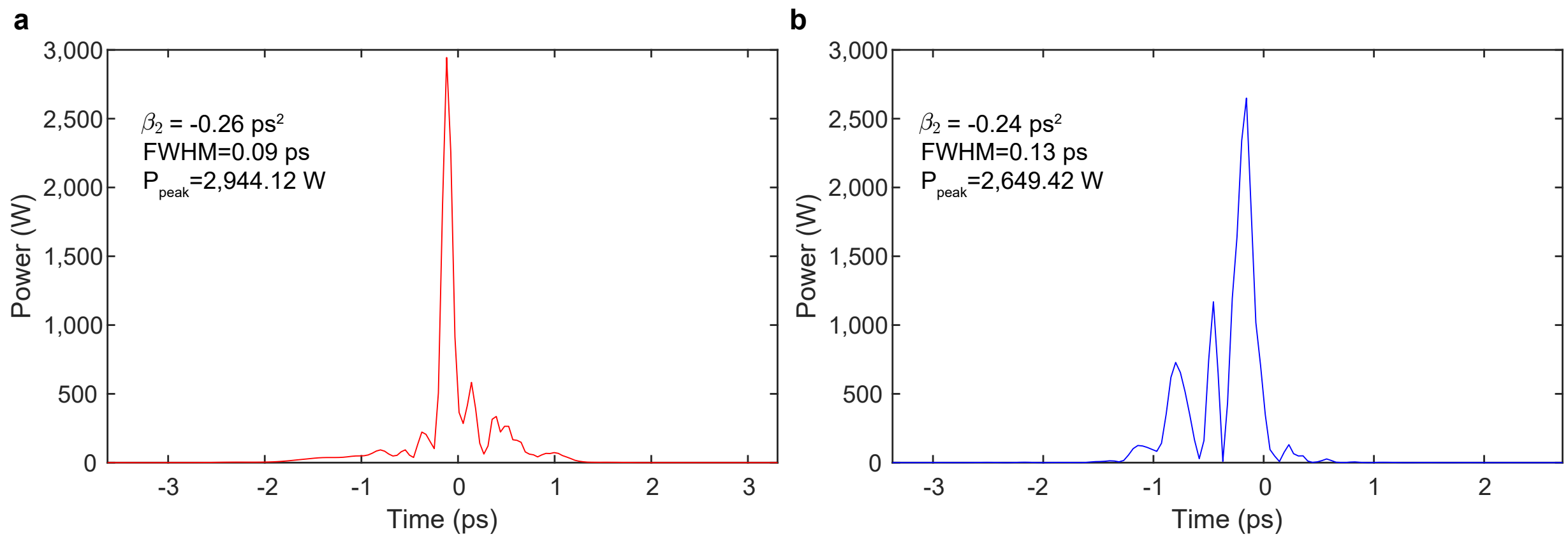

Figure S6. **Compressed output pulses. a,** Forward-propagated and **b,** Backward-propagated output pulse after optimal group delay dispersion compensation. β_2: group delay dispersion applied. FWHM: full width at half maximum of the compressed pulse. P_{peak}: peak power after compression.

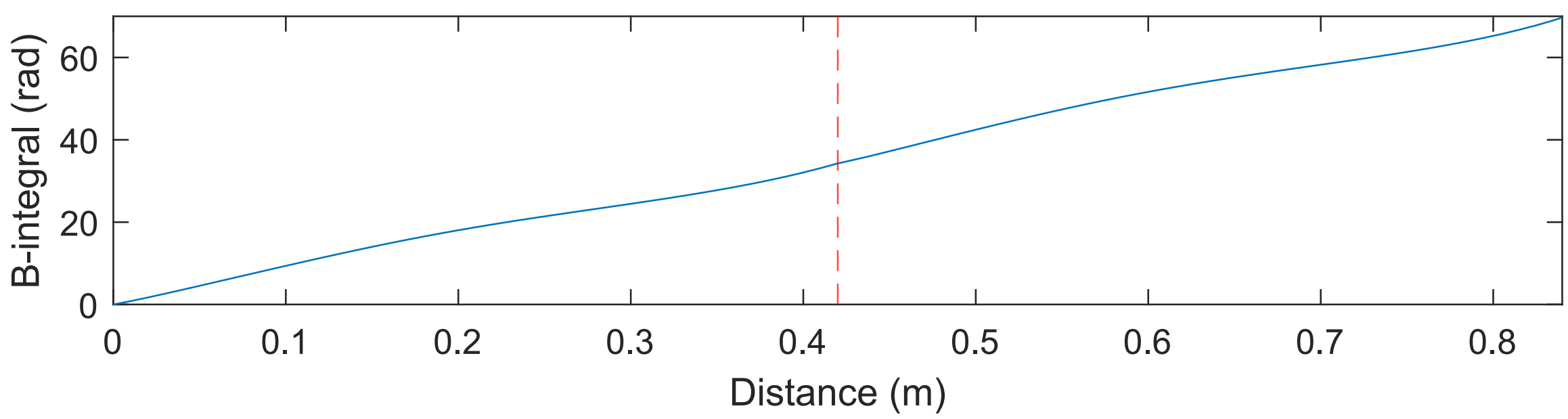

Figure S7. Accumulated nonlinear phase shift (B-integral) during pulse propagation in the linear cavity. The vertical line separates forward and backward propagation segments.

a round-trip. The cavity lengths where stable mode-locking is observed correspond to a repetition rate range of 500 MHz to 131 MHz. Further extension of the repetition range may be achieved either by using a higher doping concentration, by including additional sections of dispersion engineered passive (undoped) waveguides in the cavity, or by using a pulse interleaver at the output [58]. Our current design is made with a moderate cavity length and relatively small grating separation, to enable reuse of calibrated grating designs. It lies well within the stable regime, leaving a margin for unforeseen fabrication variations. We believe that in future developments, different repetition rates and potentially higher dechirped peak power can be achieved with an optimized design.

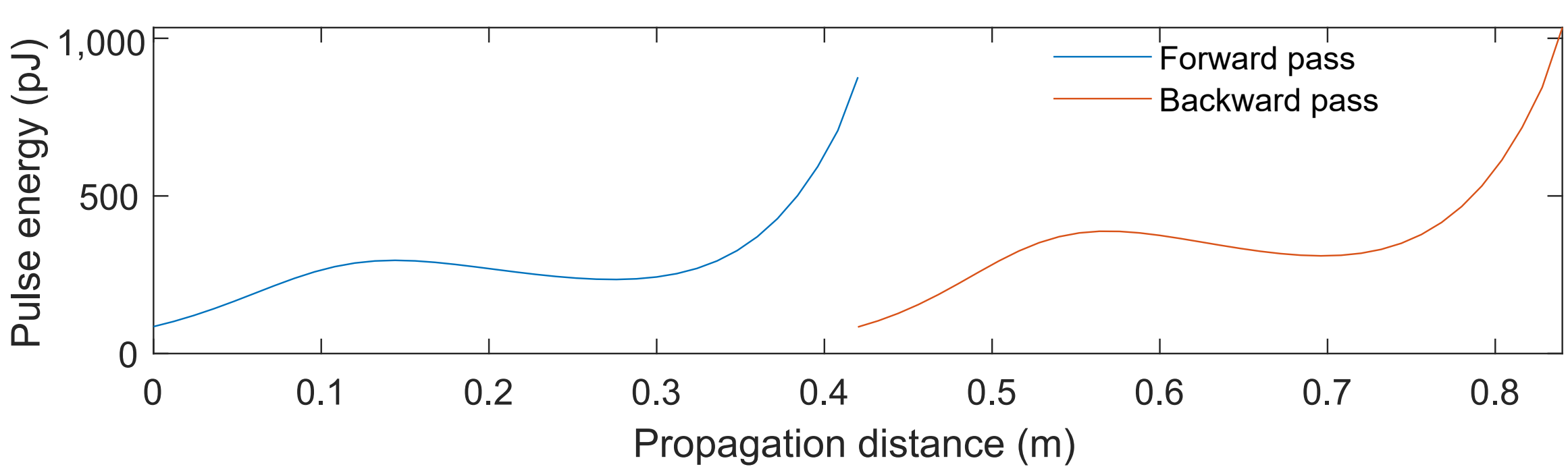

Figure S8. Pulse energy as a function of the propagation distance. The forward propagation pass and backward propagation pass are plotted separately and the drop in energy represents out-coupling from the cavity.

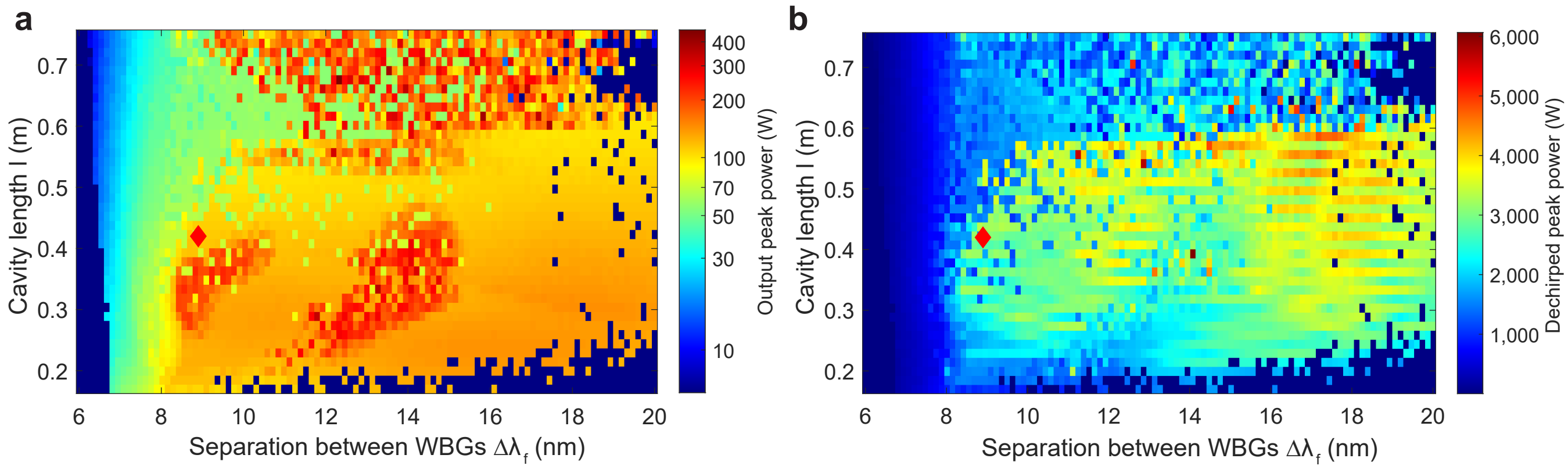

Figure S9. **Parameter map of peak power. a,** Intracavity peak power after backward propagation as a function of WBG center wavelength offset $\Delta\lambda_\mathrm{f}$ and cavity length l. Color map is in logarithmic scale. **b,** Output peak power after optimal group delay dispersion compensation. The red diamond marker indicates the location of the current design.

D. Design analysis guidelines

One way to understand the pulse propagation behavior in the waveguides is to rewrite the GNLSE (Eq. (1)) in a normalized, dimensionless form following [47]. We define a characteristic pulse duration T_0 and a characteristic peak power P_0 for the pulse reflecting off the grating. This allows us to define two fundamental length scales: the dispersion length $L_\mathrm{D} = \frac{T_0^2}{|\beta_2|}$ and the nonlinear length $L_\mathrm{NL} = \frac{1}{\gamma P_0}$. We then introduce dimensionless variables for distance (ξ), time (τ), and the optical field (U):

$$\xi = \frac{z}{L_\mathrm{D}}, \quad \tau = \frac{t}{T_0}, \quad U = \frac{A}{\sqrt{P_0}} \tag{7}$$

Substituting these into the original equation and multiplying by L_D yields the normalized form. We define the soliton number $N = \sqrt{\gamma P_0 L_D} = \sqrt{\frac{L_\mathrm{D}}{L_\mathrm{NL}}}$ and the normalized net gain $\hat{g} = (g - \alpha)L_D$. The term $\hat{g}$ represents the net power growth scaled to the dispersion length; in lasers, this is subject to gain saturation and clamping, and is determined by intracavity loss and the out-coupling ratio. The propagation equation thus simplifies to:

$$\frac{\partial U}{\partial \xi} = -\frac{i}{2}\mathrm{sgn}(\beta_2)\frac{\partial^2 U}{\partial \tau^2} + \frac{\hat{g}}{2}U + iN^2|U|^2U \tag{8}$$

This implies that in (8), if N^2 and $\hat{g}$ are comparable for two systems, the pulse propagation dynamics will also be similar. Table S3 compares the key parameters of two different integrated Mamyshev oscillator designs to those in optical fiber [43]. While the integrated Mamyshev oscillator is governed by the same underlying physics (Kerr nonlinearity, dispersion, and rare-earth doped gain) as its fiber-based counterparts, the unnormalized physical parameters of the integrated platform (γ, β_2, l, g, α) are in a vastly different regime due to the tighter mode-confinement in waveguides. However, both integrated designs feature a comparable soliton number ($N \sim 10$) to the fiber-based laser, though the normalized gain factor $\hat{g}$ differs significantly. Our intuition is that, to prevent wave-breaking and ensure gradual self-phase modulation during propagation, it is preferable to maintain $N \sim \mathcal{O}(10)$ while utilizing normal GVD ($\mathrm{sgn}(\beta_2) > 0$). Consequently, stronger normal dispersion and a wider grating bandwidth (which decreases T_0) permit a higher peak power P_0. A wider grating also increases the total reflectance of the circulating power and the cavity finesse, potentially decreasing threshold power. Thus, the strong normal dispersion of the 350 nm thick waveguides and the high 3 dB grating bandwidth in our current design directly contribute to the high peak power and pulse energy achieved experimentally.

These design principles can be adapted to other waveguide platforms selected for availability or co-integration requirements. Alternative platforms may present different material refractive indices, dispersion profiles, cross-sectional geometries, and gain properties, alongside cavity length (l) limitations dictated by chip area and defect rates. The first step in the design is extracting parameters for gain, passive loss, and maximum achievable non-chirped grating bandwidth (often limited by the fabrication process via Δn). Given a specific application constraint on pump power, the numerical analysis described above can determine suitable operational parameters.

As an example, an alternative design could target a lower-power integrated Mamyshev oscillator featuring a reduced mode-locking threshold, trading off output pulse energy. For this goal, one can reduce the waveguide dispersion β_2

Table S3. **Comparison of physical and dimensionless parameters between a typical fiber-based Mamyshev oscillator design and the integrated Si_3N_4 oscillator.** Some parameters are order-of-magnitude estimates extracted from [43]. T_0 and P_0 correspond to the pulse passing through/reflected by the filter and entering the nonlinear section.

**Parameter**	**Symbol**	**Fiber laser [43]** Yb-doped SMF	**Integrated laser** 350 nm thick Er:Si_3N_4	**Integrated laser** 600 nm thick Er:Si_3N_4
Physical parameters				
Center wavelength	λ_c	1030 nm	1555 nm	1555 nm
Nonlinear coefficient	γ	$\mathbf{0.005\,W^{-1}m^{-1}}$	$\mathbf{1.145\,W^{-1}m^{-1}}$	$\mathbf{0.987\,W^{-1}m^{-1}}$
Group velocity dispersion	β_2	$\mathbf{\sim 0.025\,ps^2/m}$	$\mathbf{0.715\,ps^2/m}$	$\mathbf{0.069\,ps^2/m}$
Saturated gain	g_{sat}	$\mathcal{O}$(4 dB/m)	$\mathcal{O}$(25 dB/m)	$\mathcal{O}$(21 dB/m)
Cavity half-length	l	4.1 m	0.42 m	0.6 m
Filter bandwidth		4 nm	5 nm	5 nm
Parameters from numerical simulation, $P_{\mathrm{p}} = 75$ mW for 600 nm integrated laser.				
Filtered pulse width	T_0	$\sim$ 0.5 ps	$\sim$ 1 ps	$\sim$ 1 ps
Peak power	P_0	$\sim$ 2 kW	$\sim$ 80 W	$\sim$ 7 W
Dimensionless parameters for pulse propagation				
Dispersion length	$L_{\mathrm{D}} = T_0^2/\lvert\beta_2\rvert$	$\sim$ 10 m	$\sim$ 1.4 m	$\sim$ 15 m
Normalized cavity length	l/L_{D}	$\sim$ 0.4	$\sim$ 0.3	$\sim$ 0.04
Nonlinear length	$L_{\mathrm{NL}} = 1/(\gamma P_0)$	$\sim$ 0.1 m	$\sim$ 0.01 m	$\sim$ 0.14 m
Soliton number	$N = \sqrt{L_{\mathrm{D}}/L_{\mathrm{NL}}}$	**$\sim$ 10**	**$\sim$ 9**	**$\sim$ 10**
Norm. saturated gain	$\hat{g} = g_{\mathrm{sat}} L_{\mathrm{D}}$	**$\mathcal{O}$(35)**	**$\mathcal{O}$(40)**	**$\mathcal{O}$(315)**

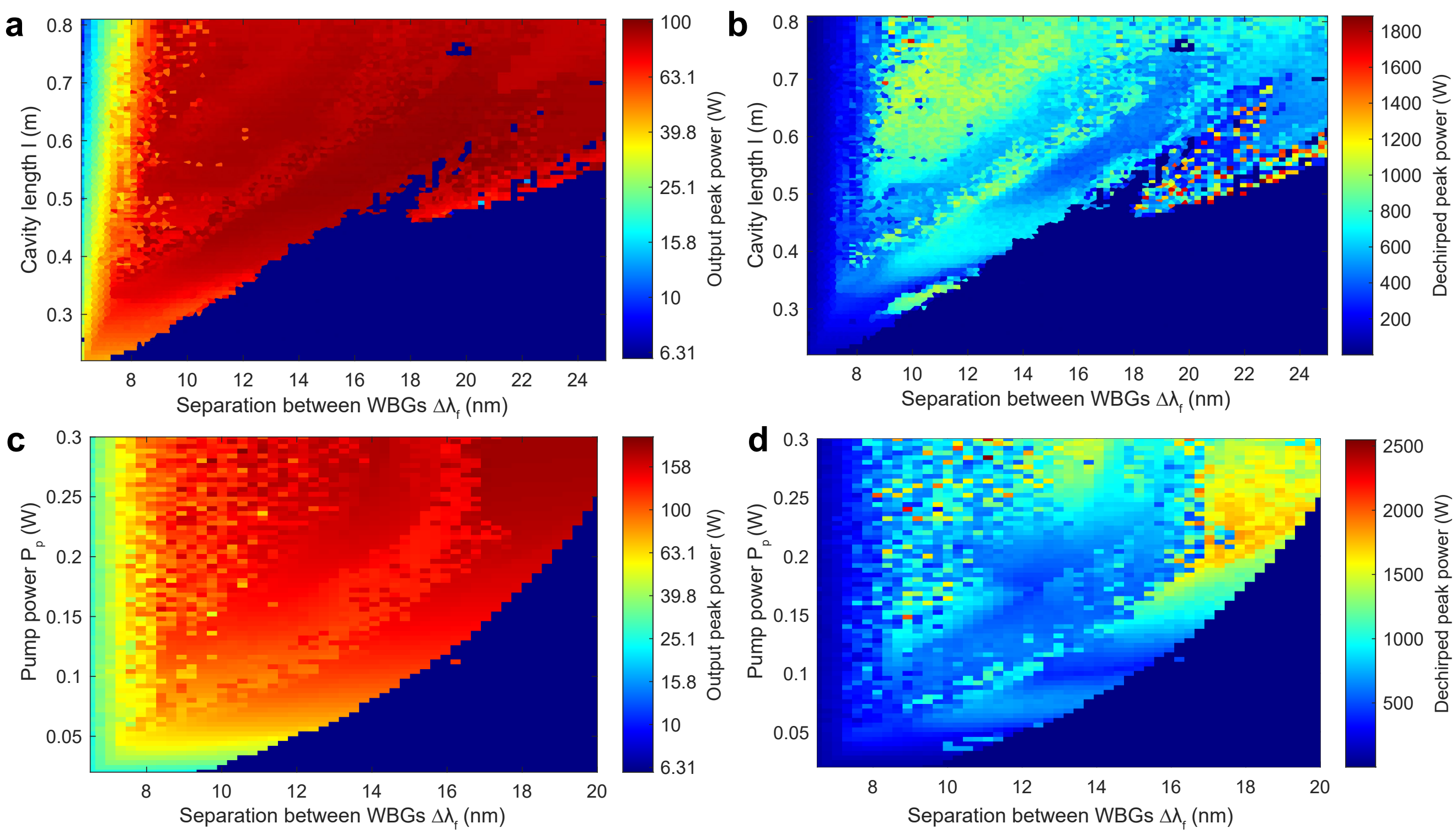

Figure S10. **Parameter maps of peak power for a 2 µm × 600 nm waveguide.a,** Intracavity peak power after backward propagation versus WBG center-wavelength offset $\Delta\lambda_{\mathrm{f}}$ and cavity length l (logarithmic color scale). Pump power $P_{\mathrm{p,fwd}} = P_{\mathrm{p,bwd}} = P_{\mathrm{p}} = 75$ mW. **b,** Output peak power after optimal group-delay-dispersion compensation versus WBG center-wavelength offset $\Delta\lambda_{\mathrm{f}}$ and cavity length l. **c,** Intracavity peak power after backward propagation versus pump power P_{p} and WBG center-wavelength offset $\Delta\lambda_{\mathrm{f}}$. **d,** Output peak power after optimal group-delay-dispersion compensation versus pump power P_{p} and WBG center-wavelength offset $\Delta\lambda_{\mathrm{f}}$.

to allow a similar $N \sim 10$ to be reached for a reduced P_0. We can select Er:Si_3N_4 waveguides with a 2 µm × 600 nm cross-section. Compared to the 350 nm devices used in the main text, these exhibit significantly weaker normal dispersion ($\beta_2 = 0.069\,\mathrm{ps^2/m}$) but a comparable nonlinear coefficient ($\gamma = 0.987\,\mathrm{W^{-1}m^{-1}}$, Figure S2). If ion implantation is similarly restricted to a maximum energy of 500 keV (a common practical limit for air-insulated implanters), the overlap factor Γ drops significantly to ~ 0.09. This can be partially countered by increasing the peak erbium ion concentration N_0 to $4.76 \times 10^{26}\,\mathrm{m^{-3}}$; if higher-energy implantation is available, the ion distribution and dose should be adjusted accordingly [59].

Assuming the same grating response as the 350 nm lasers, numerical simulations indicate a wide range of feasible mode-locking parameters. The acceptable WBG separation $\Delta\lambda_f$ scales with cavity length l (Figure S10 a,b), though an unstable pulsing regime appears at $\Delta\lambda_f > 18$ nm and $l > 0.45$ m. This aligns with the physical intuition that longer cavities produce more self-phase modulation, allowing for greater filter separation, but risk destabilizing the pulse due to extreme nonlinear phase shifts. Simulations demonstrate this laser can operate at on-chip pump powers as low as $P_p = 25$ mW per side. Parameter sweeps of $\Delta\lambda_f$ and P_p (Figure S10 c,d) map the regions supporting stable mode-locking. Higher pump powers increase intracavity power and spectral broadening, accommodating larger grating separations. However, increasing P_p beyond 100 mW at narrow grating separations (< 10 nm) degrades pulse stability (where N increases to above ~ 20), indicating a regime that should be avoided. Final design parameters should be selected within these established stable boundaries.

S3. Detailed description of the experiment setup

A. Basic mode-locking operation

Pump power for the integrated MLL engine is currently provided by single-mode-fiber-coupled 1480 nm lasers. In the highest power demonstration, we use a Connet VLSS-1480-B-800-1 (based on two polarization combined laser diodes in a butterfly package, pump 1) and a Lumibird CRFL-05-1480-OM1-8230-FA (a Raman fiber laser) for the 959 mW pump 2. For the demonstrations with lower power, we use only the Connet laser. When the 959 mW pump 2 is used, the Lumibird laser is operated at 2 W output power and attenuated before sending to the wavelength division multiplexer (WDM).

In the demonstration of self-seeding by pump modulation, the pump lasers are polarization combined, butterfly packaged diode lasers. Two diodes from QPhotonics are used for pump 1 and one diode from QPhotonics and one from Aerodiode are used for pump 2. We note that for the self-seeding at lower power (approximately < 1000 mW total power), we have deliberately disabled one of the two diodes on each end to reach the lower total power. For the basic operation of the laser, no polarization beam combiners are required.

For future integration, we note that the currently used powers are compatible with high power InP laser diodes in butterfly packages after polarization combining, commercially available from vendors including Lumentum [60] (S35/S36 series, rated at 600 mW) and Anritsu (AF4B SERIES type E, rated at 650 mW). These laser diodes can be either hybrid integrated or integrated in systems as external pump modules. The use of the Lumibird Raman laser is purely for experimental convenience rather than a limitation of the architecture.

Two thin-film-filter-based WDMs (Ascentta FWDM-45-L-10-FA) are used to inject the 1480 nm pump light while simultaneously extracting the laser output in the 1550 nm band. To minimize back-reflection from the pump lasers, we use thin-film filter-based WDMs of the “1480nm pass / 1550nm reflect” configuration, which offer better 1550 nm band isolation at the 1480 nm port compared to typical fused-fiber WDMs or the “1550nm pass / 1480nm reflect” variants. These WDMs also feature sufficiently wide passbands in the 1550 nm range to accommodate the MLL output spectrum. The pump power after the WDM is calibrated using an optical power sensor (Thorlabs S145C). We note that the WDMs can be readily implemented on chip using either a directional coupler or an unbalanced Mach-Zehnder interferometer design for future hybrid integrated devices. In this tapeout, we used 1480 nm pumping for a lower coupling loss with our current facet couplers designed for 1550 nm, while pumping at 980 nm is likely possible as well. A higher gain coefficient is achievable with 980 nm pumping due to the more complete population inversion, which may be helpful for extending this laser concept to higher repetition rates, although the quantum defect is larger and can lead to lower on-chip power efficiency, even though GaAs-based pump laser diodes are generally more power-efficient.

Isolators or circulators are placed along the 1550 nm signal path to suppress back-reflections from photodetectors and other diagnostic instruments for the characterizations shown in the main text. Meanwhile, we have also successfully demonstrated mode-locked operation without isolators or circulators (when self-seeded); no significant impact on performance has been observed, except that parasitic continuous-wave lasing may appear for the supercontinuum-generation experiment described below. If isolators are still desirable for downstream applications, passive and broadband hybrid-integrated isolators [61] have been recently shown. Such parasitic lasing does not prevent the

laser from continuing to generate mode-locked pulses capable of driving an octave-spanning supercontinuum. This can be prevented by using butt coupling with index matching for the second thick silicon nitride chip, or, in future integration, by placing both waveguides on the same chip. The integrated microheaters are powered by a DC power supply (Rigol DP832A) or a source measure unit (Keithley 2450). For many samples, stable mode-locking can be reached without using the microheaters, although some samples required tuning to prevent parasitic continuous-wave lasing. Light is coupled into and out of the chip by butt-coupling with cleaved Coherent UHNA7 fibers. To reduce facet reflections, the fiber-chip interface is covered with index-matching gel (Thorlabs G608N3), ensuring no air gap remains at the contact point. The sample is mounted on a metal block equipped with a thermoelectric cooler, which is feedback-controlled via a thermistor for temperature stabilization. Thorlabs NanoMax flexure stages are used to position the fibers.

Average output powers from both outputs are monitored using Thorlabs S144C power sensors, placed after several broadband fiber directional couplers. During calibration, the wavelength-dependent insertion losses between the common port of the WDMs and the power sensors are characterized using a swept tunable laser and taken into account to ensure accurate measurement of the laser output power. The optical spectra from both outputs are simultaneously monitored using two optical spectrum analyzers (OSAs): a Yokogawa AQ6370D with a 0.2 nm resolution (Output 1), and a HP 71451B with 1 nm resolution (Output 2). The power spectral density (PSD) plots shown in the main text are corrected for insertion loss and normalized to a resolution bandwidth of 0.2 nm for both outputs. However, there remains significant uncertainty in the absolute power level in the optical spectrum due to the varying physical resolution bandwidths/instrument bandpass of these grating-based OSAs. The time-domain pulse traces are recorded using a DC-coupled fast photodiode (Thorlabs DET08CFC/M) and a digital oscilloscope.

B. Self-seeding by pump modulation

The integrated Mamyshev oscillator can be self-seeded when the pump power is modulated. In that case, we use two Koheron CTL300 laser drivers for two laser diodes (Aerodiode) providing the pump from one side, and two SRS LDC502 laser drivers for two other diode lasers (QPhotonics and Aerodiode) providing pump from the other side. The powers are combined with a fiber-coupled polarization beam splitter and are directly applied to the WDMs. The current modulation is applied to the Koheron drivers via the digital bias switching input (`BSEL`), programmed to switch the current setpoint between zero and a fixed level. This allows fast modulation with a higher depth than the analog input despite a significant distortion in the waveform. For each pump power, we select a modulation frequency of the Koheron drivers between 1.5 MHz and 2.8 MHz to achieve the fastest startup, and the modulation frequency of the SRS drivers is fixed to 1.2 MHz due to the lower bandwidth of its analog modulation input.

The procedure to start the Mamyshev oscillator can be readily automated as follows:

1. Turn on the pump power.
2. Set the microheaters, wait up to 0.5 s for stabilization.
3. Enable pump modulation.
4. Wait for the time described in Section S6;
   Optional: detect whether the laser has already entered the mode-locking state with a photodiode. If so, skip waiting.
5. Disable pump modulation. *Now the laser should be mode-locked.*
6. *Optional:* Fine-tune the pump power and heater setpoint to target a desired pulse energy and output spectrum.

We note that programmable generation of the modulation signal can be straightforwardly implemented with low-cost electronics, such as using the pulse width modulation (PWM) outputs from modern microcontrollers. Applying the modulation to the laser diode driver is also possible with a transistor shunt switch.

C. Autocorrelation characterization

To characterize the pulse duration after applying dispersion compensation, we perform intensity autocorrelation measurements using the output from Port 1 of the laser (path A in the main text). The signal is first sent through an optical isolator to prevent backreflections, then through a programmable optical filter (Finisar Waveshaper 1000s) for dispersion compensation, and is subsequently amplified using a home-built erbium-doped fiber amplifier (EDFA) to compensate for the ~ 6 dB loss in the filter. The total fiber path length from the chip facet to the autocorrelator (APE pulseCheck USB 50) is approximately 11 m. We avoid the use of commercial EDFAs, which typically employ several meters of erbium-doped fiber and are known to introduce distortions to the pulse due to uncontrolled dispersion and nonlinear effects at high peak powers. Instead, we construct an EDFA with minimal fiber length to preserve the pulse

fidelity. The custom EDFA consists of two 980/1550 nm WDMs (Thorlabs), a 980 nm pump laser diode (Aerodiode), and a short (∼50 cm) section of highly doped erbium fiber (LIEKKI ER80-4/125-HD-PM). To further minimize unwanted nonlinear broadening, we keep the fiber connection between the EDFA output and the autocorrelator as short as possible (approximately 1 m).

In an alternative approach, we employ the group delay dispersion of the SMF to compress the MLL output pulse. The signal from output 2 of the laser (path B) is routed through a SMF delay line to the autocorrelator. In this case, no EDFA is used and the autocorrelation measurement is performed directly after the fiber. The total length of the fiber is approximately 10 m, corresponding to a dispersion of $-0.22\ \mathrm{ps}^2$.

D. Coherence characterization

To characterize the radio-frequency (RF) repetition-rate beatnote via direct photodetection, we use a high-power-handling p-i-n photodiode (Discovery Semiconductor DSC40S). The RF spectrum is measured using an electronic signal analyzer (ESA, Keysight N9020A), while phase noise is characterized using a signal-source analyzer (Rohde & Schwarz FSUP26), which offers sensitivity beyond the phase noise floor of standard ESAs. The timing jitter T_j is calculated by integrating the measured phase noise spectrum (after removing spurs), using $T_\mathrm{j} = \frac{1}{2\pi f_\mathrm{rep}}\sqrt{2 \times \int 10^{\mathrm{SSBPN}(f)/10}\,\mathrm{d}f}$, where SSBPN($f$) is the single-sideband phase noise at offset frequency f in units of dBc/Hz. An optical attenuator is used to adjust the optical power to the photodetector, as the photodetected RF signal phase noise can be affected by photodetector saturation and amplitude-noise-to-phase-noise (AM-to-PM) conversion. For narrowband measurements of the RF beatnote and its phase noise, we use bandpass filters (Mini-Circuits ZX75BP-1062-S+ for the 6th harmonic, ZX75BP-188-S+ and SLP-250+ for the fundamental harmonic) and a low-noise RF preamplifier (Mini-Circuits ZFL-1000LN+) to optimize the signal-to-noise ratio. For wideband spectral analysis of the beatnote, no preamplifier is used to avoid saturation and nonlinear harmonic generation within the amplifier.

To evaluate the linewidth of individual comb lines by heterodyne detection, we first preselect a narrow spectral portion of the MLL output using a tunable fiber Bragg grating (AOS GmbH), aligned to match the wavelength (∼ 1552 nm) of a single-frequency erbium-doped fiber laser (Koheras Adjustik). After optimizing polarization, the filtered comb signal and the reference laser are combined using a 50:50 directional coupler and detected with a balanced photodetector (Discovery Semiconductor DSC720-39). The resulting heterodyne signal is analyzed using an ESA (Rohde & Schwarz FSW). For frequency noise extraction, a 100 ms segment of the in-phase and quadrature baseband signal is recorded from the ESA, converted to phase and post-processed using Welch's method.

E. External seeding

To initiate mode-locking in the integrated Mamyshev oscillator by external seeding, we use the experimental setup shown in Fig. S11. A commercial fiber MLL (Menlo ELMO High Power) serves as the seed source. Because the pulse width from the laser (51 fs full-width-half-maximum) is considerably shorter than required to start the integrated MLL, we employed a programmable optical filter (Finisar Waveshaper) to degrade the pulse to emulate a lower-performance laser. The spectrum of the seed pulse is reduced using a Gaussian-shaped filter, typically with a bandwidth of 0.15 THz to 0.6 THz. This also avoids excessive nonlinear broadening and pulse breaking of the seed pulse in subsequent fiber propagation in the test system. Meanwhile, a group delay dispersion of 0.4 ps/nm is also programmed to the filter to pre-compensate for dispersion in the fiber-based experiment setup. The pulse is subsequently amplified by another home-built low-nonlinearity erbium-doped fiber amplifier (EDFA) to boost the power to approximately 20 mW. To select single pulses from the pulse train, we use an electro-optic Mach-Zehnder intensity modulator (EOSpace) driven by a pulse picker board (Aerodiode). The pulse picker is synchronized with the seed MLL using the trigger output from the MLL. We note that the repetition rate of the seed MLL is unrelated to that of the integrated MLL when the mode-locked state has been initiated by the gated pulse. We also note that the Mamyshev oscillator is relatively tolerant to variations in seed pulse parameters, particularly when parasitic backreflections from the fiber coupling or experimental setup are minimized. During experiments, stable mode-locking can be established across a broad parameter space of the seed, even when the energy, filter bandwidth, or polarization state are varied substantially. We have observed that once started with a single pulse, the laser tends to stabilize at the same state as dictated by the pump power and grating configuration after tens of round-trips, like a stable attractor in the nonlinear dynamical system, regardless of the seed parameters. A single pulse is sufficient to start the mode-locked operation when the pump power is sufficient and fiber coupling alignment is optimized, corresponding to a starting time of tens of round trips (on the order of hundreds of nanoseconds).

The minimum bandwidth of the Gaussian-filtered pulse used for seeding is lower than 0.15 THz, corresponding to a transform-limited pulse width of ∼3 ps. The seed pulse energy delivered on chip ranged from 15 pJ to 45 pJ. We have achieved repeatable seeding with pulses of 15 pJ on-chip energy and 0.15 THz bandwidth, which is within reach of integrated III-V mode-locked diodes combined with on-chip erbium-doped waveguide amplifiers.

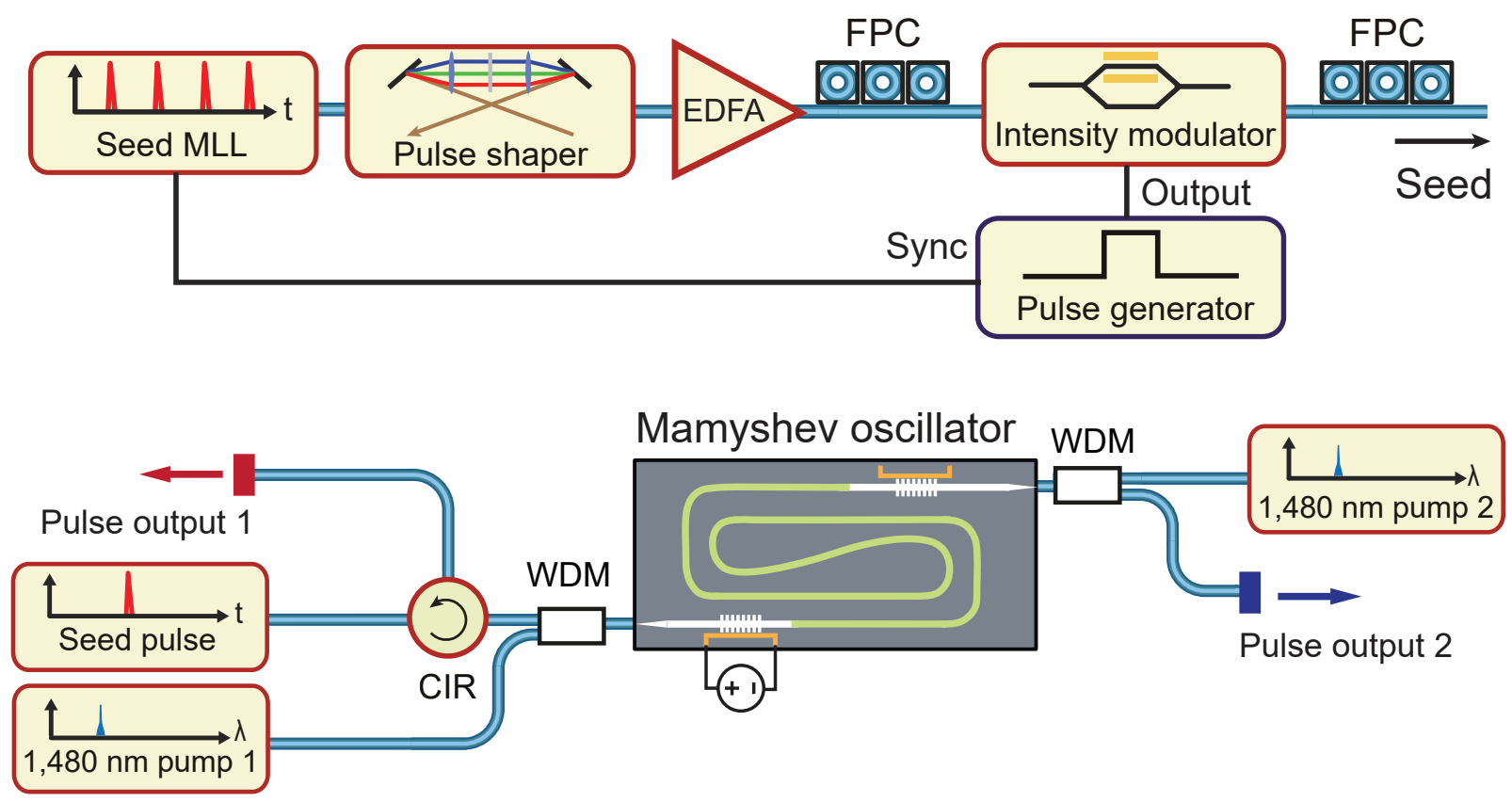

Figure S11. Schematic of the seed source used to initiate Mamyshev oscillator operation and the setup for external seeded initiation of the mode-locked state.

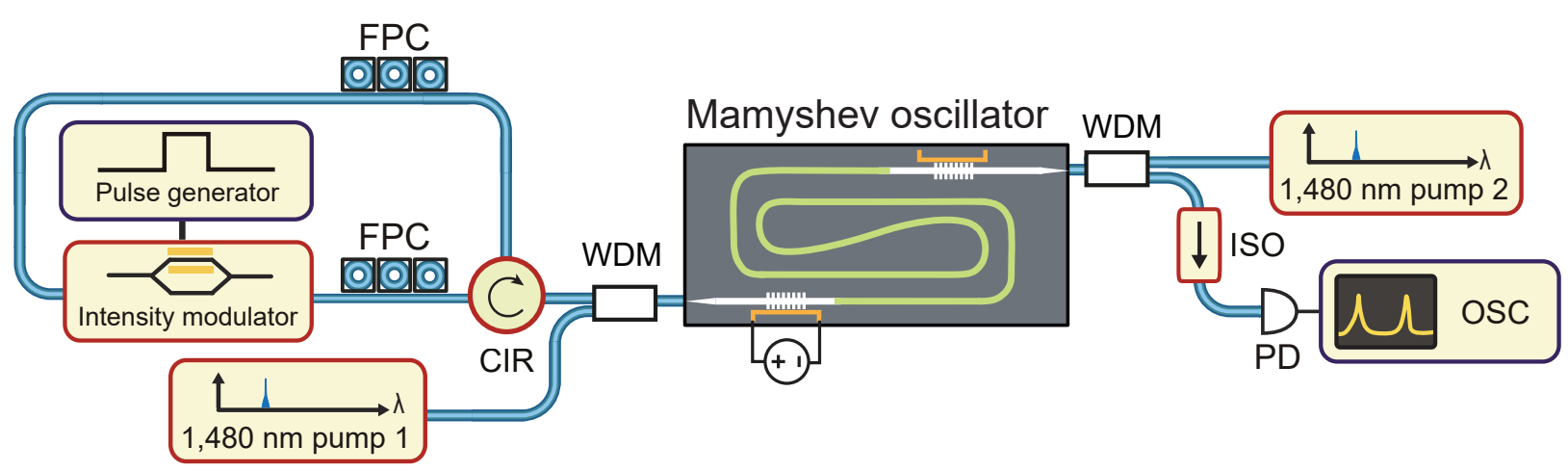

Figure S12. Schematic of the experimental setup for initiating the Mamyshev oscillator using an extended cavity Q-switching method.

F. Alternative starting method by extended cavity Q-switching

A high-energy, nanosecond-duration intracavity pulse can also initiate mode-locking. To demonstrate this, we construct an extended cavity by routing one output port of the device back into the waveguide and implementing active Q-switching using an external modulator. We use a higher off-chip pump power of 2000 mW from pump 1 and 820 mW from pump 2 during the starting, although these powers can be significantly lowered after mode-locking is started. As illustrated in Fig. S12, a controllable extended cavity is created using an electro-optic intensity modulator (EOSpace) in combination with a circulator (Thorlabs). The modulator is driven by a function generator at a repetition rate of 1 kHz, providing high transmission for approximately 200 ns per period in the extended arm of the cavity. A Q-switch pulse can build up in the main cavity and the extended arm after the gate is switched on, providing a pulse with sufficient energy to initiate the mode-locking (see Section S5). Since the intensity modulator operates on a single polarization with the orthogonal polarization being blocked, we empirically optimized the fiber polarization controller settings by monitoring the Q-switching behavior on an oscilloscope.

G. Supercontinuum generation

For the supercontinuum generation demonstration, the output from the WDM (including approximately 2.5 m of fiber) is routed through a series of fiber-connected components. The signal first passes through an optical isolator with a 2.18 m pigtail, followed by a polarization controller (3.43 m of fiber), and is then directed into a 1550 nm lensed fiber (approximately 2 m in length), which couples the light into a second Si_3N_4 chip. We estimate a coupling loss

of 2.5 dB from fiber to chip near 1550 nm. The optical isolator is included to prevent back-reflection from the lensed fiber and the input facet of the Si_3N_4 supercontinuum chip (potentially up to a few percent) which can disrupt stable mode-locking. For future monolithic implementations, the optical isolator can be omitted, and the approximately 3.8 dB power penalty from coupling loss and fiber components can also be eliminated. The output from the second chip is collected using a similar 1550 nm lensed fiber (not optimized for short-wavelength coupling efficiency) and routed to two optical spectrum analyzers (Yokogawa AQ6375 and AQ6373) for spectral characterization. To eliminate potential second-order diffraction artifacts from the grating-based optical spectrum analyzers in the long-wavelength region (1800 nm to 2400 nm), two Thorlabs FELH1250 long-pass filters are used in a free-space fiber bench (Thorlabs) to block shorter wavelengths during acquisition in this region. The long end of the transmission band of the FELH1250 filters is 2150 nm and their transmission at >2200 nm is not documented, so it is possible that the output power at the longer wavelengths is underestimated.

H. THz time-domain spectroscopy

We use two commercial fiber-coupled photoconductive antennas (PCAs; Menlo Systems TERA15-TX-FC and TERA15-RX-FC) for the THz field emission and sampling. Each PCA is equipped with a 1 m fiber pigtail and an integrated high-resistivity silicon hyperhemispherical lens to reduce beam divergence. Because the emitted THz beam remains weakly divergent, we use metallic off-axis parabolic mirrors (Thorlabs) for collimation and re-focusing. The total THz beam path length is approximately 25 cm.

After the WDMs, the output of the integrated Mamyshev oscillator is directly sent through a length of single-mode fiber for partial chirp compensation, followed by a polarization controller, a fiber-coupled polarization beam splitter (PBS), and a 50:50 splitter. No optical isolator is used. The polarization controller and the PBS are used for power monitoring and adjustable attenuation, since the $> 100\,\mathrm{mW}$ laser output can easily exceed the optical power rating of the current PCAs used (PCAs rated for higher optical power are available on the market). After the final splitter, one arm is sent to the receiver PCA and the other to the transmitter PCA via a free-space optical delay line.

Before THz measurements, we optimize the pulse duration at the PCAs by selecting the dispersion-compensation fiber length while monitoring the pulse with an autocorrelator. To ensure that the autocorrelator measurement faithfully represents the pulses delivered to the PCAs, we insert an additional 1 m fiber section before the autocorrelator input, matching the fixed pigtail length of the PCAs. With this procedure, we routinely achieve autocorrelation function FWHM of approximately 200 fs at the PCA inputs. The time delay is provided by a motorized free-space delay line (Thorlabs ODL100) operated at $20\,\mathrm{mm\,s^{-1}}$ (double pass). In future systems aiming for a compact solution or fast scanning, asynchronous optical sampling techniques [62] could also be applied to eliminate the mechanical delay line.

To suppress $1/f$ noise from the PCAs and electronics, we employ lock-in detection and modulate the transmitted THz signal by applying a square-wave modulation to the transmitter PCA bias. A high-voltage amplifier (Falco WMA-300), driven by a function generator (Keysight 33622A) at approximately 23 kHz, provides a 0 V to 100 V modulation. The receiver PCA output is treated as a current signal from a high-impedance source; with a clear beam path, the peak photocurrent is typically on the order of 100 nA to 500 nA. We demodulate the signal using a lock-in amplifier (SRS SR630) with built-in transimpedance amplifier (10^6 V/A gain) and digitize the two-channel demodulated output with a NI USB-6216 acquisition device. We average multiple scans over approximately 5 min. The main THz electric field peaks from each scan are temporally aligned prior to averaging. Windowed Fourier transformation of the THz time-domain waveform yields the corresponding frequency-domain spectrum.

To demonstrate thickness metrology, we insert a double-side-polished, high-resistivity float-zone silicon wafer into the THz beam path. The time delay between the main pulse and the reflected pulse is $\Delta t_{\mathrm{rt}} = 11.93\,\mathrm{ps}$. This delay corresponds to the round-trip propagation time of the terahertz pulse inside the silicon wafer. Accordingly, the single-pass delay is $\Delta t_{\mathrm{sp}} = \Delta t_{\mathrm{rt}}/2 = 5.965\,\mathrm{ps}$. Using the refractive index of $n_{\mathrm{Si}} = 3.4173$ [63], the wafer thickness d is obtained from $d = \frac{c\,\Delta t_{\mathrm{rt}}}{2n_{\mathrm{Si}}} = 523.3\,\mu\mathrm{m}$, where c is the speed of light. The minimum measurable thickness is set by the minimum resolvable separation between the main pulse and its internal-reflection replica. In our THz-TDS system, the 3 dB width of the main time-domain peak is $\Delta t_{\mathrm{rt,3dB}} = 0.35\,\mathrm{ps}$, and we take this value as an estimate of the minimum resolvable delay without using deconvolution. The corresponding thickness resolution is therefore $d_{\mathrm{min}} = \frac{c\,\Delta t_{\mathrm{rt,3dB}}}{2n_{\mathrm{Si}}} = 15.4\,\mu\mathrm{m}$.

To demonstrate material identification, we measure lactose ($C_{12}H_{22}O_{11}$), which exhibits well-known absorption features in the THz band and is of practical interest as lactose content in dairy products such as milk powder is relevant to individuals with lactose intolerance. A sample of lactose powder (Adipogen SA) is sealed in a thin plastic bag and placed in the beam path; the bag material is effectively transparent at THz frequencies. For comparison, we place wheat flour (from a local supermarket) packaged in an identical bag, which is visually similar to lactose, in the beam

path. We compute the absorbance as $A(f) = -\ln[T(f)]$, where the transmittance is $T(f) = P_{\text{sample}}(f)/P_{\text{reference}}(f)$ and $P(f)$ denotes the measured THz power.

S4. Details on the waveguide Bragg gratings

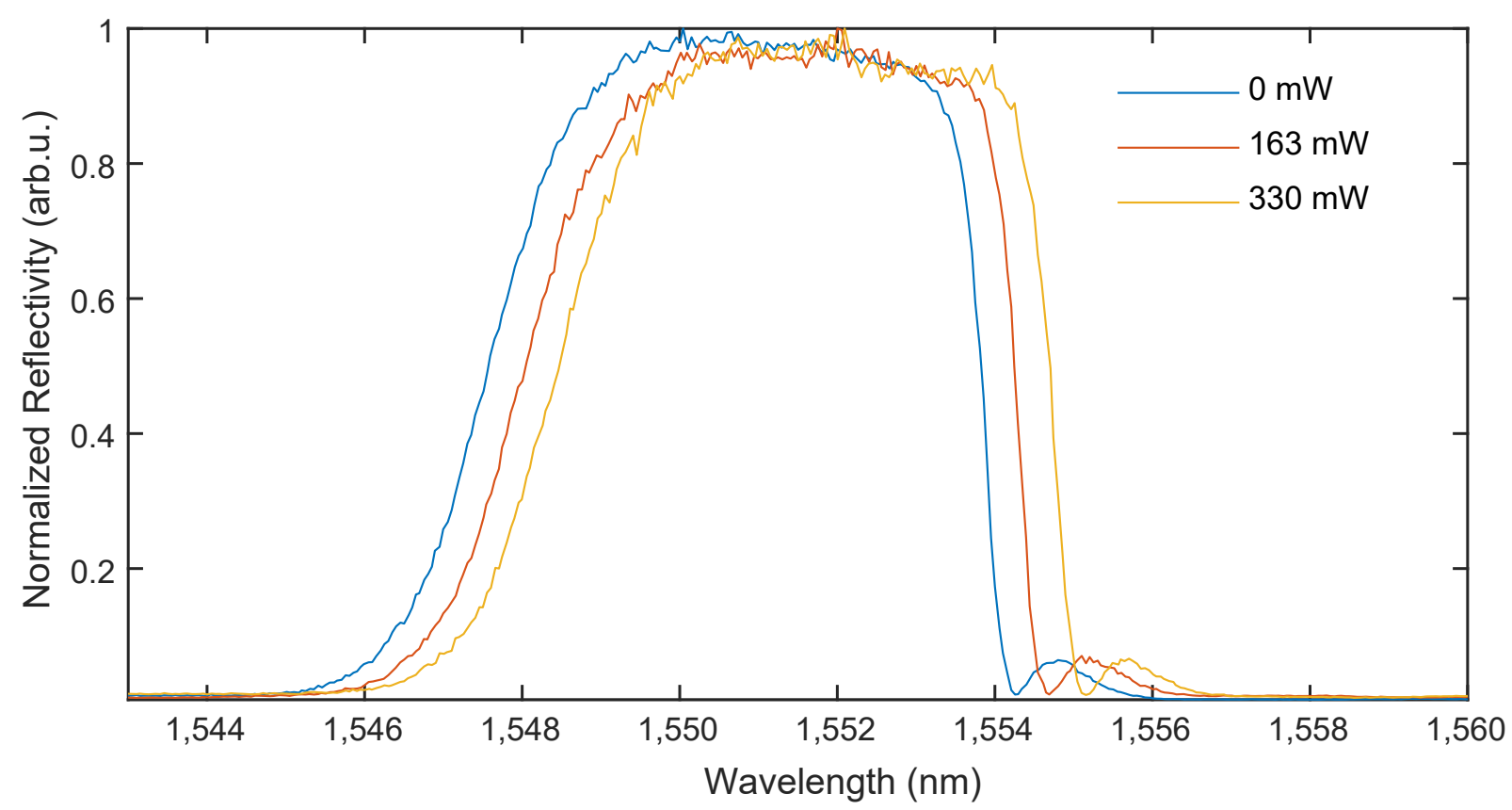

Figure S13. Normalized reflectance spectrum of a representative WBG for different electrical powers applied to the microheater (0 mW, 163 mW, and 330 mW). The reflection band red-shifts with increasing heater power.

For the WBGs, we adopt an apodized grating design [64], using only 950 grating periods to reduce both chromatic dispersion and cladding mode scattering loss at the pump wavelength [65], while maintaining sufficient reflectivity.

The unpowered grating reflection spectrum shown in Figure 1h of the main text is characterized using a swept single-frequency laser (Toptica CTL) serving as the probe source. The reflected signal is extracted using a 50:50 directional coupler (Fibermart) and measured with a power meter (Thorlabs) at the polarization maximizing reflection signal. Each of the two gratings is probed individually from the closest ends of the device. The probe power is kept sufficiently low to avoid reaching the transparency of the erbium-doped waveguides, ensuring that nearly all transmitted light is attenuated between the gratings. The same UHNA fiber butt-coupling setup as in the main experiments is used to minimize backreflections from the chip facets during measurement.

To quantify the thermal tunability of the WBGs, we separately characterize the WBG reflection spectrum at different heater powers. The measurement is performed with lensed fibers for chip coupling. The characterized grating has a slightly higher corrugation strength than the WBGs used in the laser experiments; however, the thermal tuning behavior is expected to be comparable because it is primarily determined by the cross-section geometry and thermal conductivity. As shown in Figure S13, increasing heater power produces a clear red shift of the reflection band. To extract the band center, we identify the two wavelengths at which the reflectivity reaches 50% on the rising and falling edges of the reflection band and take the average of the two. Using this definition, we obtain a tuning efficiency of approximately 2.66 pm/mW, corresponding to -0.332 GHz/mW near 1550 nm. We have operated the heaters continuously at ~ 500 mW electrical power for several hours without observing degradation. At this power level, the demonstrated tuning range is approximately 1.33 nm. In future fabrications, thermal isolation trenches can be created during the deep etch die separation process to reduce the power requirement for tuning.

S5. Dynamics of pulse build-up

In the experiments, mode-locking can be initiated either by (1) modulating the pump current, (2) using an external mode-locked laser to seed the laser or (3) generating an energetic pulse with an extended cavity and Q-switch.

The pump modulation method is preferable in most cases as no off-chip optical component is required and the pump current modulation can be implemented with commercial drivers (see Section S3 B) or simple external electronics. In that case, the pump can be modulated at a frequency close to the relaxation oscillation frequency of the parasitic continuous-wave lasing, thus leading to a significantly amplified response in the intracavity intensity or Q-switched pulsing. Such high-intensity, slow, quasi-continuous-wave (CW) pulsing can be observed in Figure S14, before the probabilistic initiation of a continuous train of fast mode-locked pulses. After the appearance of the mode-locked

pulse, the pump current modulation can be disabled at any moment (i.e. can be immediately disabled, or if limited by control electronics, at any later time) as the mode-locked pulse train is stable. This is verified by using the burst mode of the signal generator for the modulation. When the modulation is disabled, the laser will then reproducibly convert into a stable pulsing state as shown in Figure S15. Notably, the required modulation frequency for the integrated laser is significantly higher (MHz level) than that for fiber-based lasers in [66] (100 kHz level), which corresponds to the difference in relaxation oscillation frequency.

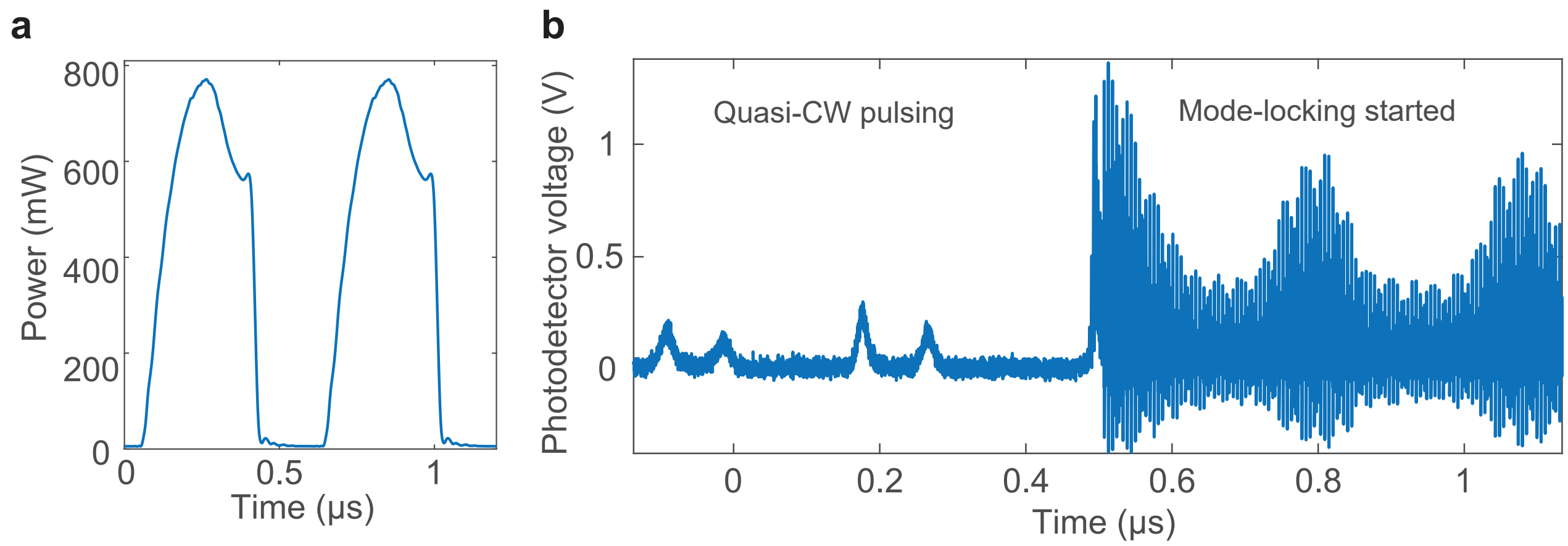

Figure S14. **The self-seeded pulse build-up process monitored with a photodetector and oscilloscope. a,** Instantaneous power of the diode laser when current modulated at 1.7 MHz. The vertical axis is calibrated against the average power measured with a thermal pile power meter. **b,** Pulse emergence from strong relaxation oscillation pulses. The captured trace only covers a segment before switching off the modulation. The amplitude modulation envelope of the mode-locked pulses will disappear when the modulation is switched off.

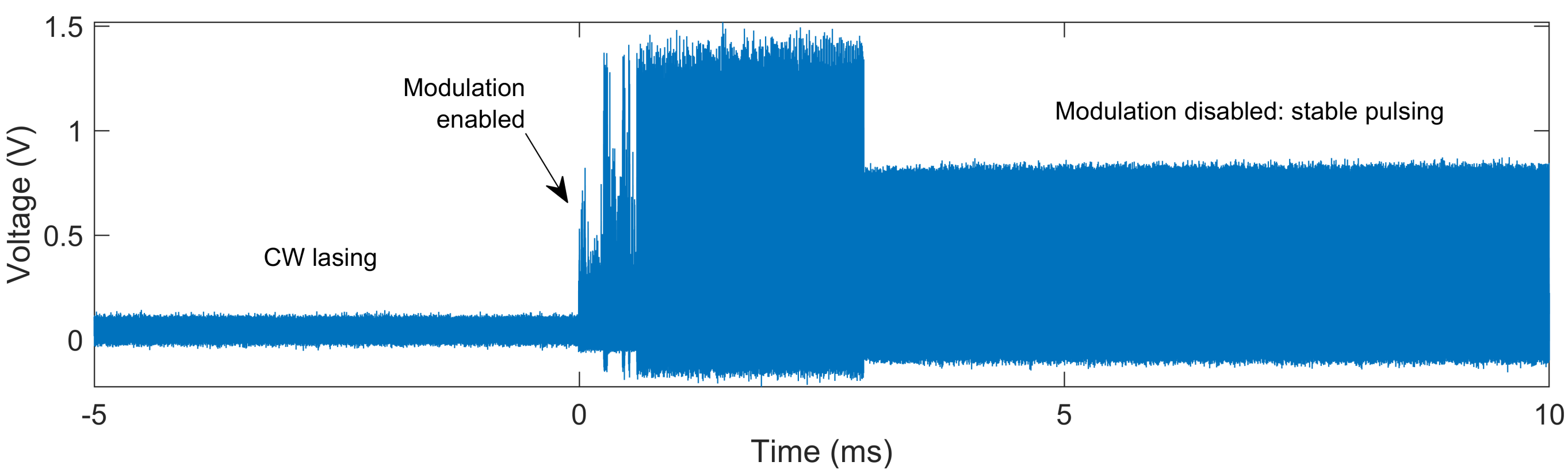

Figure S15. Pulse emergence dynamics from the relaxation oscillation pulsing, and transition into stable pulsing after switching off modulation.

Alternatively, pulse initiation can be achieved using an extended cavity arm and an electro-optic Q-switch, similar to the scheme in [67] with the effective saturable absorber replaced with a modulator. In this case, the dynamics differ significantly: mode-locked pulse trains emerge following a high-energy Q-switched pulse, as shown in Figure S16 b. The Q-switched pulse lasts approximately 24 ns and carries an on-chip energy on the order of 100 nJ, estimated roughly by integrating the pulse area (with possible large error due to photodiode saturation), comparable to the values reported in [68]. A train of fast pulses builds up after the slow Q-switched pulse and rapidly increases in amplitude, eventually splitting into two pulses after roughly 150 ns due to excess energy. This later evolves into a single-pulse state (Fig. S16 c) as the population inversion decreases. Significant amplitude fluctuations are observed immediately after initiation, but they damp out quickly and nearly vanish after around 3000 round-trips (Fig. S16 d), leading to a low noise mode-locked state. This behavior may correspond to the high-energy chaotic state during pulse build-up as discussed in [69].

When external seeding is used to start the mode-locked laser, Figure S16 a shows the temporal evolution when the laser is seeded by a short pulse from an external MLL, as described in Section S3 E. In this case, similar to the "coherence memory" regime reported in [69], the injected seed pulse directly evolves into the stable mode-locked pulse in the cavity. The pulse amplitude exhibits only a modest ∼20% overshoot before settling into a steady state within approximately 20 round-trips.

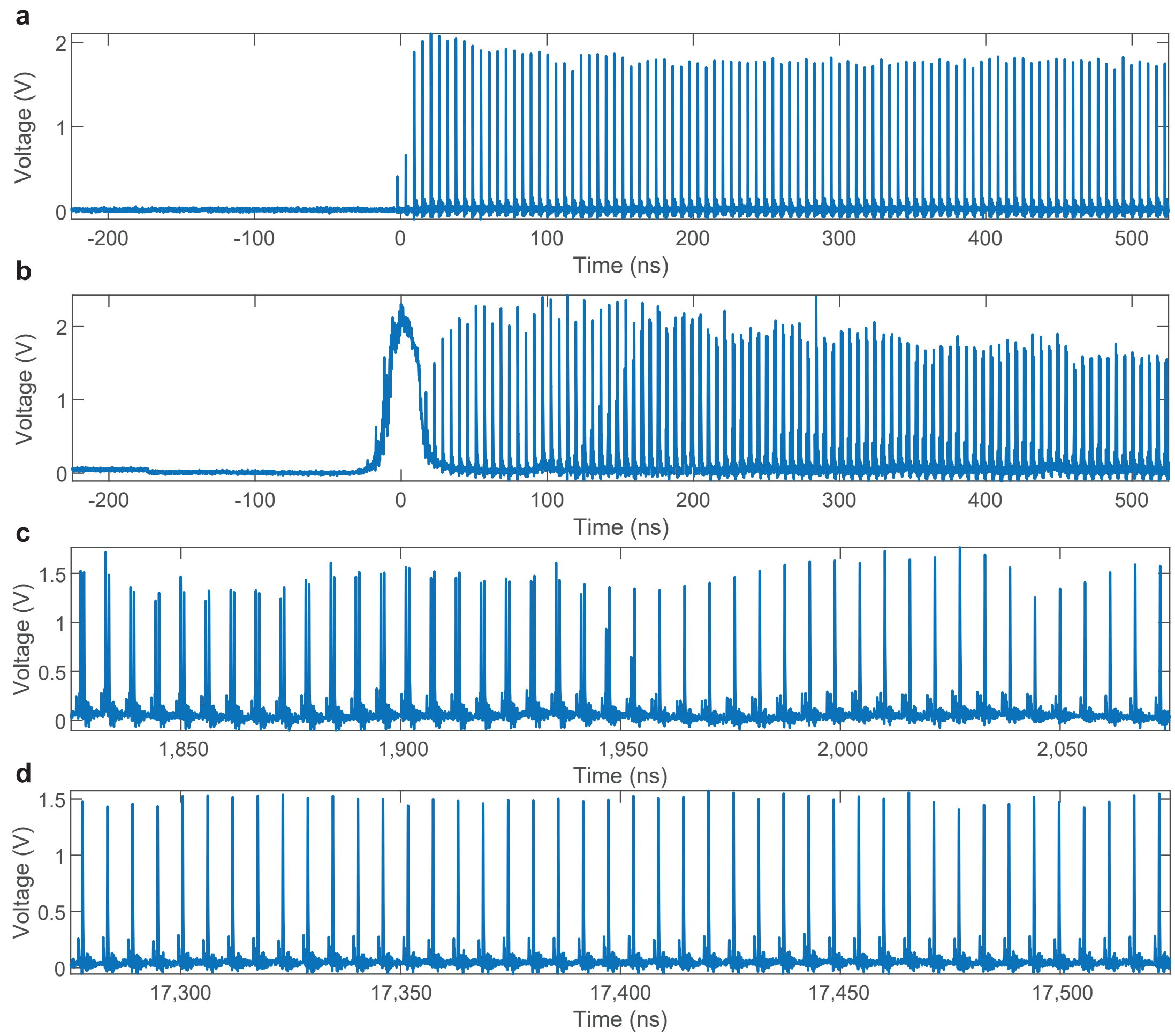

Figure S16. **Instantaneous output power during the pulse build-up process for external seeding or extended cavity Q-switching.** Monitored with a photodetector and oscilloscope. **a,** Pulse evolution after seeding by an external MLL pulse. **b,** Pulse evolution after initiation using an external cavity and Q-switch. **c,** Transition from a double-pulse state to a single-pulse state, occurring approximately 340 round-trips after **b**. **d,** Stable pulse train recorded approximately 3000 round-trips after **b**.

We note that the stable mode-locking state may be regarded as a stable attractor in the nonlinear system, and the system tends to reach that state given a certain set of grating and pump parameters, regardless of the route taken to reach it. In Figure S17, we compare the optical spectrum of the self-seeded mode-locked state and the state seeded by an external MLL, which is practically the same except for the expected fluctuations in the heater power and fiber coupling.

We would also like to note that we have never observed device damage during initialization of mode-locking using any of the three approaches for the currently presented devices fabricated with 350 nm thick silicon nitride. Despite the extreme intracavity pulse energy and peak power that may incur during the Q-switching or with pump modulation, we believe the damage threshold of the waveguides is still significantly higher than what is required for starting.

Several reports have demonstrated self-seeding or self-starting fiber-based Mamyshev oscillators using similar or different mechanisms [66, 70–72]. We believe the relative difficulty in starting the integrated Mamyshev oscillator arises from the large difference between the power of parasitic continuous-wave lasing and the peak power of the mode-locked pulses. To initiate mode-locking, a high intracavity gain is required to amplify small intensity fluctuations into a viable pulse seed. However, with a typical estimated back-reflection level of $10\log_{10} R_{\mathrm{parasitic}} \approx -27$ dB, parasitic

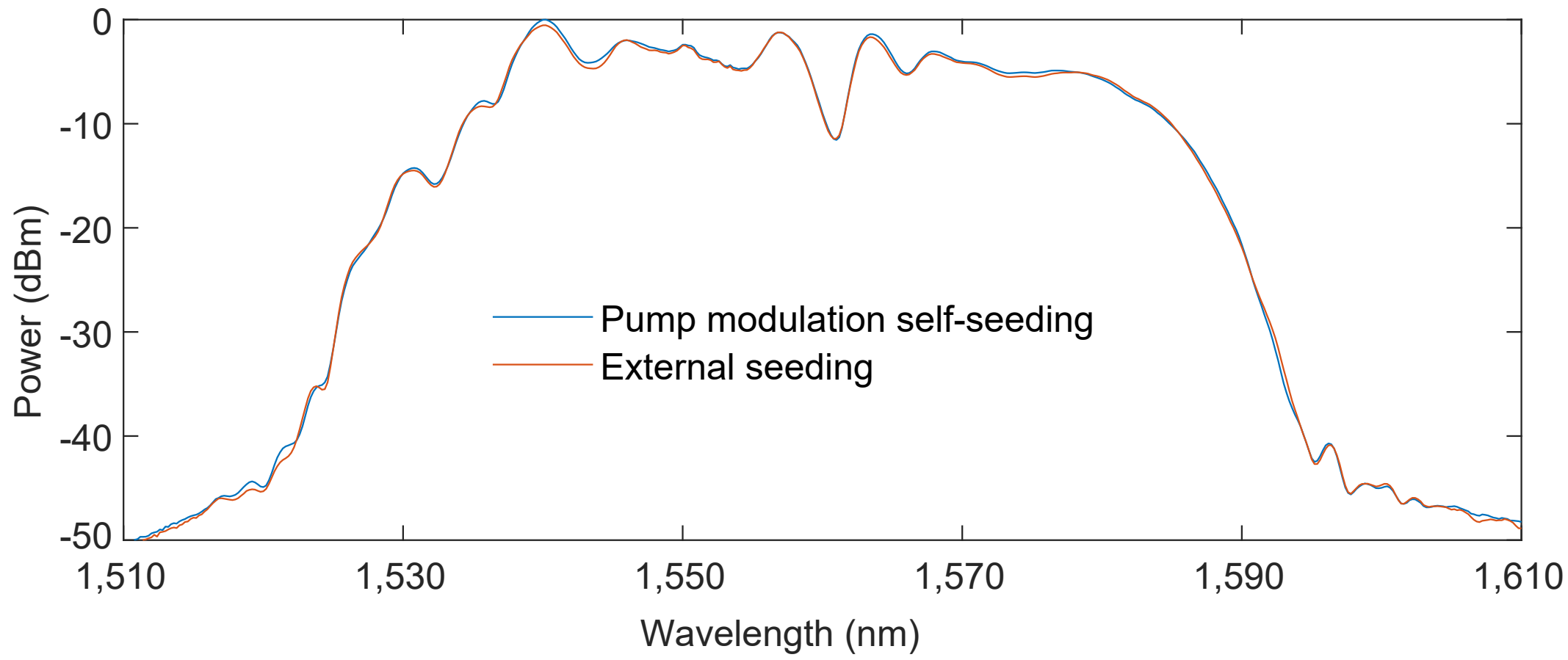

Figure S17. Comparison between the experimentally measured optical spectrum of a self-seeded mode-locked state and an external seeded state.

lasing between one of the waveguide Bragg gratings (WBGs) and the reflection point clamps the single-pass gain to only 13.5 dB, insufficient to amplify noise to a level capable of sustained pulsing. In numerical simulation, we find that approximately −42 dB of back-reflection are required for self-starting from intracavity noise without pump modulation. Nevertheless, using angled edge couplers and matched fiber array units can significantly reduce chip facet reflectivity, currently the dominant source of parasitic back-reflection. For reference, commercial angled end-face fiber connectors (e.g.: FC/APC) can routinely achieve a back reflection level of −60 dB, which would be sufficient with a significant margin.

S6. Time to start for self-seeding

For self-seeding of the integrated mode-locked laser by pump modulation, an important question is how long it takes from the start of pump modulation to the emergence of a stable mode-locked pulse train. Due to the probabilistic nature of self-seeding from slow relaxation-oscillation pulses with nanosecond duration (in contrast to external seeding), there is intrinsically a distribution of the time to start. This time is orders of magnitude longer than the cavity round-trip time. We statistically study the time to start at different pump-power levels by repeatedly applying pump modulation, waiting for the mode-locking to stabilize, and extinguishing mode-locking by either manually shifting the gratings or disabling the pump.

In the experiment, we generate a trigger signal indicating the initiation of mode-locked pulses by passing the laser output through an optical filter (Dicon, 0.8 nm bandwidth) centered at approximately 1538 nm. The quasi-continuous-wave parasitic lasing does not reach this wavelength; when mode-locked pulses with sufficient bandwidth are generated, significant power is detected by a photodiode (Thorlabs) after the filter. We feed the optical trigger signal to a digital oscilloscope to apply thresholding. We manually verified the oscilloscope traces to ensure that a continuous pulse train is generated after each triggering event. A National Instruments USB-6216 data acquisition device is used to measure the time difference between the first modulation edge sent to the laser driver and the trigger output from the oscilloscope.

As shown in Figure S18, reliable self-seeding is realized across a wide range of pump powers from 623 mW (467 mW on-chip) to 1012 mW (759 mW on-chip). Here, the pump-power level represents the total off-chip pump power at 1480 nm applied to both inputs of the integrated laser. For each pump-power level, more than 20 self-seeding events are recorded for statistical analysis. The self-seeding always occurs within 10 s, while in most cases it takes more than 100 µs. The rate of success is virtually 100%. Somewhat surprisingly, the time to start does not decrease monotonically with increasing pump power. We note that it is generally possible to initialize mode-locking at an advantageous pump-power level and then either increase or decrease the pump power to reach the desired operating state (either for higher lasing performance or for lower power consumption), while maintaining continuous mode-locked operation.

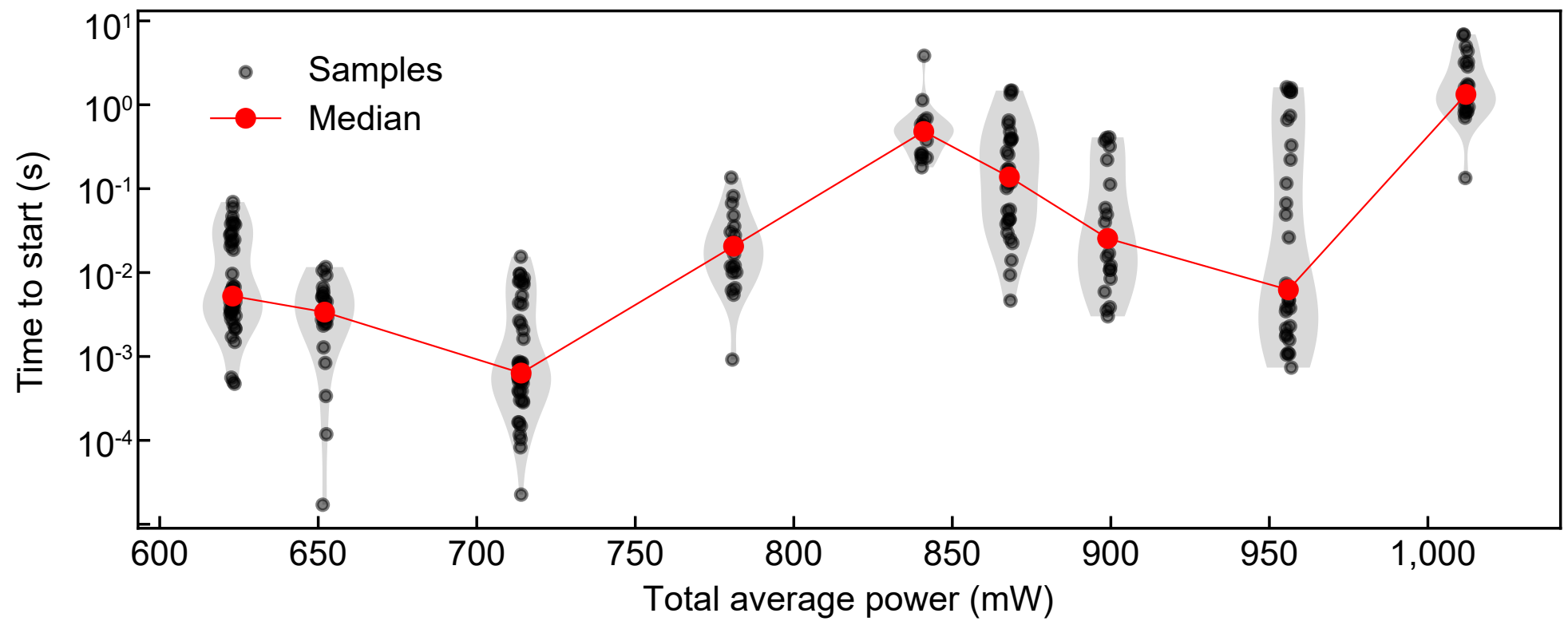

Figure S18. **Time to start:** Violin plot of the time to start stable mode-locking from the first modulation edge applied to the pump laser diodes, overlaid with the median time to start. The data points are dithered along the x-axis to allow visualization of each point with minimal overlap.

S7. Fiber-to-chip coupling and output beam quality

To efficiently couple light from fiber to photonic integrated circuits, we use edge couplers with inverse tapers that transform the guided mode to better match the mode field in the optical fiber. We use Coherent UHNA7 fiber, which has a mode-field diameter (MFD) of approximately 3 µm near a wavelength of 1550 nm, spliced to conventional SMF-28 fiber. The edge coupling efficiency η is calculated from the vectorial field overlap [73]:

$$\eta = \frac{\int \boldsymbol{E}_\mathrm{f} \times \boldsymbol{H}_\mathrm{w} \cdot \mathrm{d}\boldsymbol{S} \int \boldsymbol{E}_\mathrm{w} \times \boldsymbol{H}_\mathrm{f} \cdot \mathrm{d}\boldsymbol{S}}{\int \boldsymbol{E}_\mathrm{f} \times \boldsymbol{H}_\mathrm{f} \cdot \mathrm{d}\boldsymbol{S} \int \boldsymbol{E}_\mathrm{w} \times \boldsymbol{H}_\mathrm{w} \cdot \mathrm{d}\boldsymbol{S}}, \tag{9}$$

where $\boldsymbol{E}_\mathrm{f}, \boldsymbol{H}_\mathrm{f}, \boldsymbol{E}_\mathrm{w}, \boldsymbol{H}_\mathrm{w}$ are the electric and magnetic fields of the fiber and waveguide, respectively. The mode fields are simulated with commercial software (`COMSOL`) using the finite element method. The parameters of the UHNA7 fiber are extracted from the datasheet. The waveguide height is designed to be 350 nm as mentioned to leverage a high effective Kerr nonlinearity γ, while still maintaining sufficient overlap between the erbium ion distribution and the optical mode field. We conservatively designed the length of the tapering section to be 500 µm long to ensure adiabatic mode conversion. The theoretical coupling efficiency η as a function of taper width is shown in Fig. S19 a. The taper width is designed to be around 350 nm to maximize the mode-field overlap, which corresponds to a simulated fiber-to-fiber loss of 1.45 dB.

We characterized the coupling loss with a 2 cm dummy straight waveguide from the same wafer but without ion implantation, using a scanning single frequency laser and a power meter. As shown in Fig. S19 b, the average total fiber-to-fiber loss between 1540 nm and 1570 nm is approximately 2.52 dB, corresponding to a per-facet coupling loss of 1.26 dB. This value includes contributions from waveguide propagation loss ($<$ 0.2 dB) and splice losses between SMF and UHNA7 fibers at both ends. The splice loss, estimated at 0.62 dB, is characterized by measuring the total loss of three spliced segments (SMF-UHNA7-SMF).

We note that thermal drift and the resulting hysteresis in the coupling setup (despite the use of a TEC) make manual coupling significantly more difficult when the fiber is "hot" (carrying significant pump and output power), compared to coupling to dummy waveguides at low power. This can be resolved by improving the design of the chip mount, or properly designed packaging of the photonic integrated circuit. The coupling loss described above should therefore be considered as a lower bound for the actual loss during the experiment. Meanwhile, we note that once the system reaches thermal equilibrium (potentially after realigning the fiber), sustained stable operation with the TEC disabled is possible and demonstrated experimentally.

We would like to also note that the 26 parallel waveguides in the current layout design are not meant for simultaneous operation, due to the space constraints at the coupling edge. The 20 µm spacing between the waveguides is too small for accessing individual cavities with fiber arrays (commonly with pitch of 127 µm or 250 µm). If a high-density array of MLLs is desired, single MLL cavities can be fitted in a footprint as small as 0.35×0.15 cm^2 when the parallel spiral configuration is not used, using simulation-verified 70 µm radius bends and 9.5 µm waveguide spacing. This design requires a chip area less than 1/26 of that of the chip used in the current demonstration and can support higher integration density and better fabrication economy.

Concerning the beam quality of the emission, because we access the integrated mode-locked laser through facet couplers and butt-coupled single-mode fibers in near field, the concept of M^2 does not directly apply. In this case,

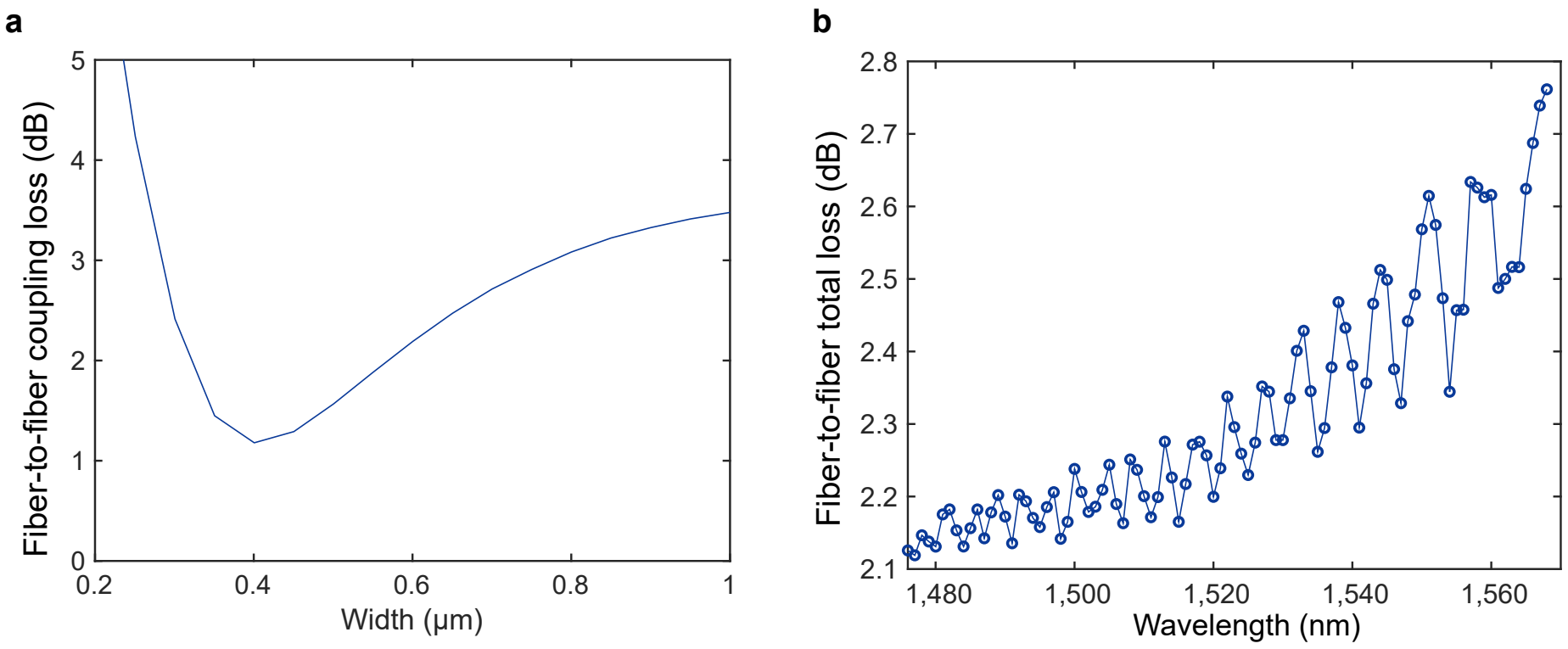

Figure S19. **Fiber to waveguide coupling loss. a,** Simulated fiber to chip to fiber coupling loss of TE mode of a cladded 350 nm thick Si_3N_4 waveguide to the UHNA7 fiber for different taper widths at 1.55 μm. **b,** Measured loss from fiber to chip to fiber for a 2 cm reference waveguide.

all the energy in the beam that is not matching the mode field is supposedly lost in the characterization. The facet couplers are also designed to feature a narrow taper that only supports the fundamental mode. For the above reason, we believe the M^2 factor of the fiber-coupled output beams should be very close to 1. These fiber-coupled beams are also what we refer all power and energy characterizations results to. The relatively high coupling efficiency of approximately 75% shown in Section S7 indicates the output has a very high spatial coherence.

To explicitly confirm this, we characterize the beam diameter from a pre-aligned fiber collimator (Thorlabs F240APC-1550) as a function of the distance from the furthest surface of the collimator (Fig. S20), including the near field and the far field regime. A fit of the beam expansion to the scaled Gaussian beam expansion law (10) in logarithmic scale yields an estimation of $M^2 = 1.08$, which is very close to 1, confirming the single-mode output.

$$D(z) = D(0)\sqrt{1 + \left(\frac{M^2\,\lambda}{\pi w_0^2}\,(z - z_0)\right)^2}, \tag{10}$$

where z_0 is the axial position of the waist, λ is the center wavelength, and z is the axial propagation coordinate at which the diameter $D(z)$ is measured.

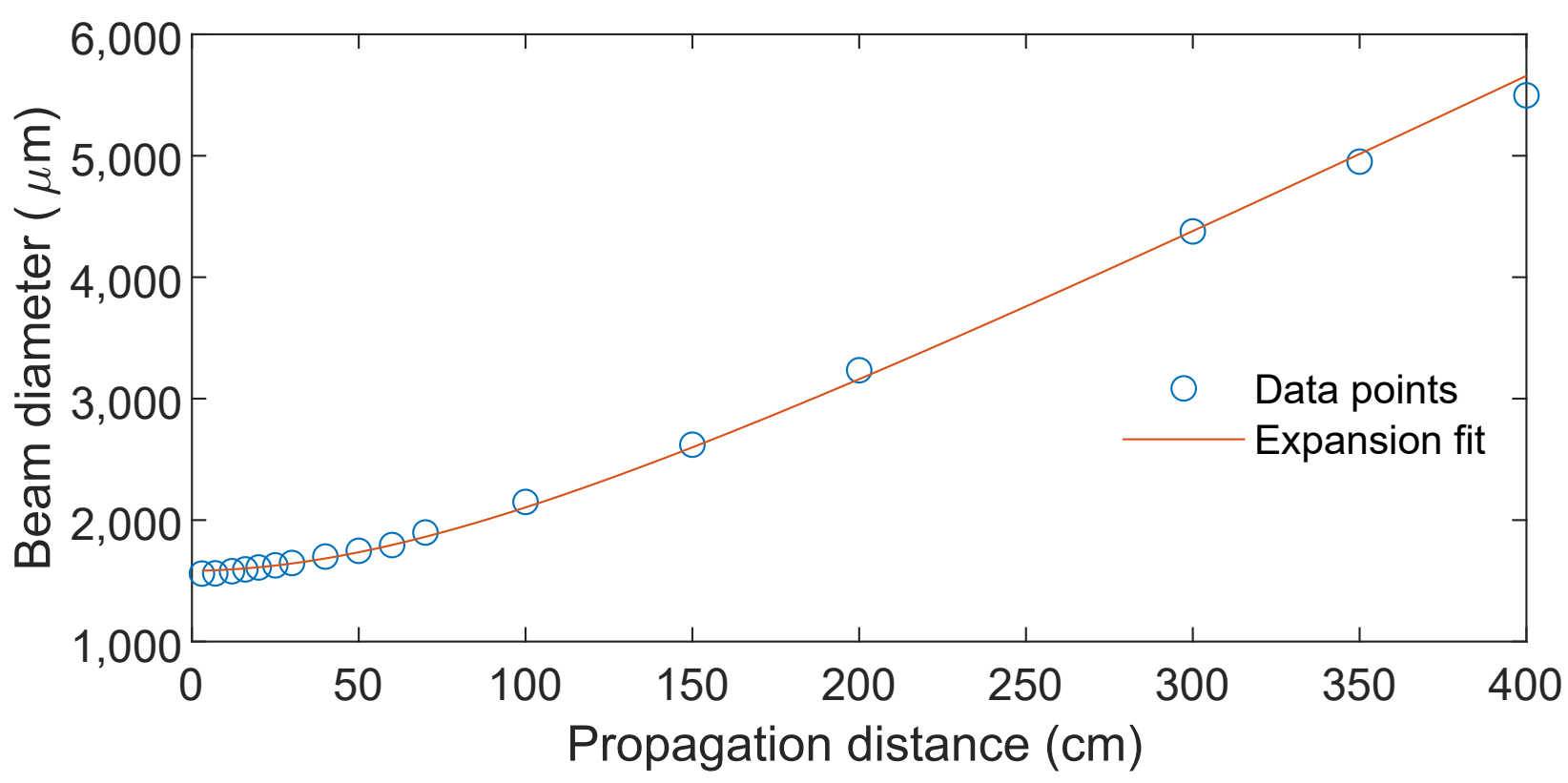

Figure S20. Beam diameter as a function of propagation distance after emerging from the fiber collimator.

S8. Time domain pulse reconstruction from dispersion sweep data

It is well known that one cannot retrieve the optical pulse profile unambiguously from the intensity autocorrelation measurement alone. In our experiments, since we have acquired a series of intensity autocorrelation functions $\mathrm{IAC}(t)$ measured under different group-delay-dispersion values β_2, together with the measured spectrum intensity $|E(\omega)|^2$,

we can computationally reconstruct the amplitude and phase of the pulse. The phase to retrieve $\phi(\omega)$ is defined by $E(\omega) = |E(\omega)|e^{i\phi(\omega)}$ and its relation to the intensity autocorrelation $\mathrm{IAC}(t)$ can be established by the following equations. First, the time-domain intensity of the pulse $I(t)$ with quadratic spectral phase assignment (β_2) is given by:

$$I(t) = \left| \mathcal{F}^{-1}\left\{ |E(\omega)|\ e^{i\phi(\omega)}\ e^{i\frac{\beta_2}{2}\omega^2} \right\} \right|^2, \tag{11}$$

where $\mathcal{F}^{-1}$ denotes the inverse Fourier transform, and ω is the offset angular frequency with respect to the carrier frequency of light. The autocorrelation of intensity can be computed in the spectral domain using the Wiener-Khinchin theorem:

$$\mathrm{IAC}(t) = \mathcal{F}^{-1}\left\{ |\mathcal{F}\{I(t)\}|^2 \right\}, \tag{12}$$

where $\mathcal{F}$ denotes the Fourier transform. Equations (11) and (12) describe the forward model of the phase retrieval problem.

We use the stochastic gradient descent optimizer in `PyTorch`, which implements automatic differentiation to calculate the gradient and perform backpropagation. The detailed algorithm is shown in Algorithm 2. Notably, we adopted the cosine similarity as the metric for the similarity between the measured and computed correlation functions, which is scale-invariant and thus robust to the amplitude mismatches between the computed and measured intensity autocorrelation function. We also applied a weighted time window to emphasize the region near the zero delay. In addition, we observed that subtracting the background at large time delays in both measured intensity autocorrelation and the forward model improves the retrieval quality. During optimization, the loss converges after 10000 iterations, and the reconstructed pulse reaches the shortest pulse width at $\beta_2 = 0.0189$ ps^2 (Fig. S21). The linear temporal phase in the main pulse peak indicates that the most of the pulse energy is well compressed. Minor ripples in the reconstructed pulse intensity away from the main peaks may be artifacts from the retrieval process. Figure S22 confirms the validity of the retrieved pulse, where the measured intensity autocorrelation is in good agreement with the computed intensity autocorrelation based on the retrieved pulse.

Algorithm 2: Spectral phase retrieval from measured intensity autocorrelation

---

Input: Measured optical spectrum $|E(\omega)|^2$, measured autocorrelation map $\mathrm{IAC_{meas}}(t, \beta_2)$ as a function of time delay t and dispersion values β_2

A. Data preprocessing

Define uniform time and frequency grids;

Interpolate $\mathrm{IAC_{meas}}(t, \beta_2)$ and $|E(\omega)|$ onto the uniform grids;

Normalize, subtract the background, and apply weighting of $\mathrm{IAC_{meas}}(t, \beta_2)$ for each β_2:

  $\mathrm{IAC_{meas'}}(t, \beta_2) \leftarrow w(t)\left(\mathrm{IAC_{meas}}(t, \beta_2) - \min_t(\mathrm{IAC_{meas}}(t, \beta_2))\right)$;

B. Parameter initialization

Set an initial guess for spectral phase $\phi(\omega)$;

Create a weighting function $w(t)$ centered at zero delay;

C. Forward model

Function `ForwardModel(`$\phi(\omega)$, β_2`)`

  `// Compute the autocorrelation function for a given spectral phase and dispersion.`

  $E(\omega) \leftarrow |E(\omega)|\, e^{i\phi(\omega)}$;

  $E(t, \beta_2) \leftarrow \mathcal{F}^{-1}\{E(\omega) e^{i\frac{\beta_2}{2}\omega^2}\}$;

  $I(t, \beta_2) \leftarrow |E(t, \beta_2)|^2$;

  Compute $\mathrm{IAC_{fwd}}(t, \beta_2)$ of $I(t, \beta_2)$ via the Wiener–Khinchin theorem;

D. Spectral phase retrieval

for $k \leftarrow 1$ **to** n_{iter} **do**

  foreach *dispersion value* β_2 **do**

    Compute the autocorrelation function: $\mathrm{IAC_{fwd}}(t, \beta_2) \leftarrow$ ForwardModel($\phi(\omega)$, β_2);

    Subtract background and apply weighting: $\mathrm{IAC_{est}}(t, \beta_2) \leftarrow w(t)\left(\mathrm{IAC_{fwd}}(t, \beta_2) - \min_t(\mathrm{IAC_{fwd}}(t, \beta_2))\right)$;

    Compute cosine similarity loss: $L(\beta_2) \leftarrow 1 - \mathrm{cos\_sim}\left(\mathrm{IAC_{est}}(t, \beta_2), \mathrm{IAC_{meas'}}(t, \beta_2)\right)$;

  Compute average loss over all β_2: $L \leftarrow \mathrm{mean}\left(L(\beta_2)\right)$;

  Update $\phi(\omega)$ with the stochastic gradient descent algorithm;

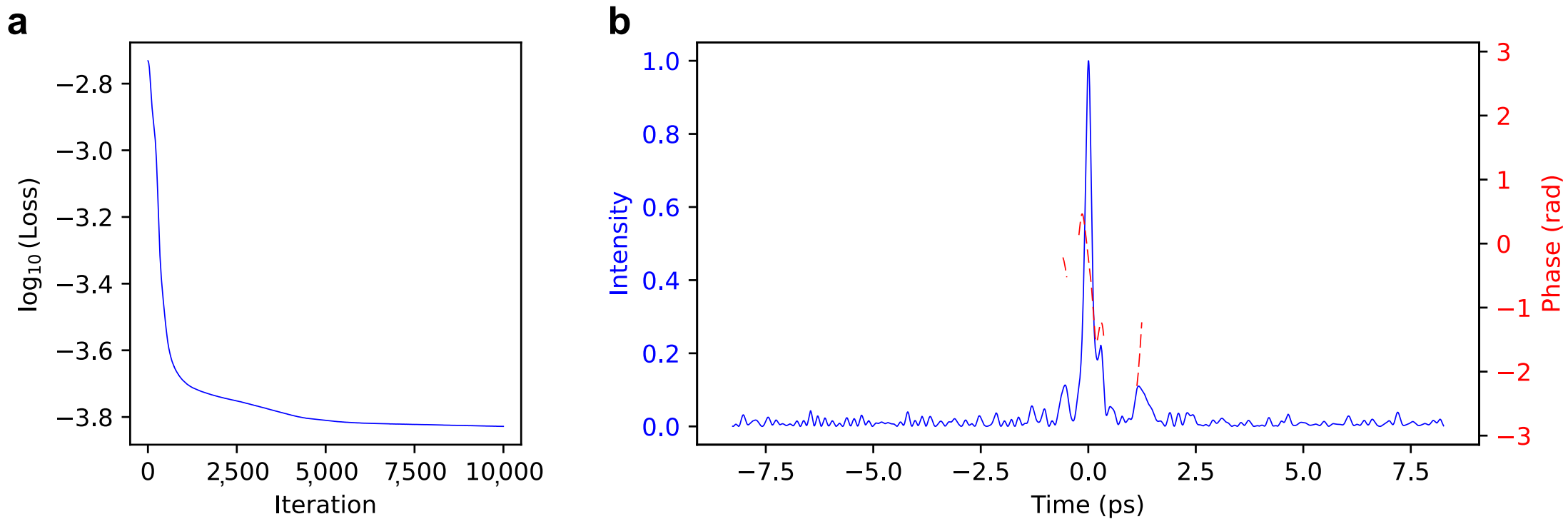

Figure S21. **a,** Evolution of the retrieval error over the course of the optimization iterations. **b,** Reconstructed pulse intensity and phase at the point of minimum pulse duration, corresponding to a dispersion value of $\beta_2 = 0.0189$ ps^2. The phase is shown only in regions where the pulse intensity exceeds a threshold for clarity.

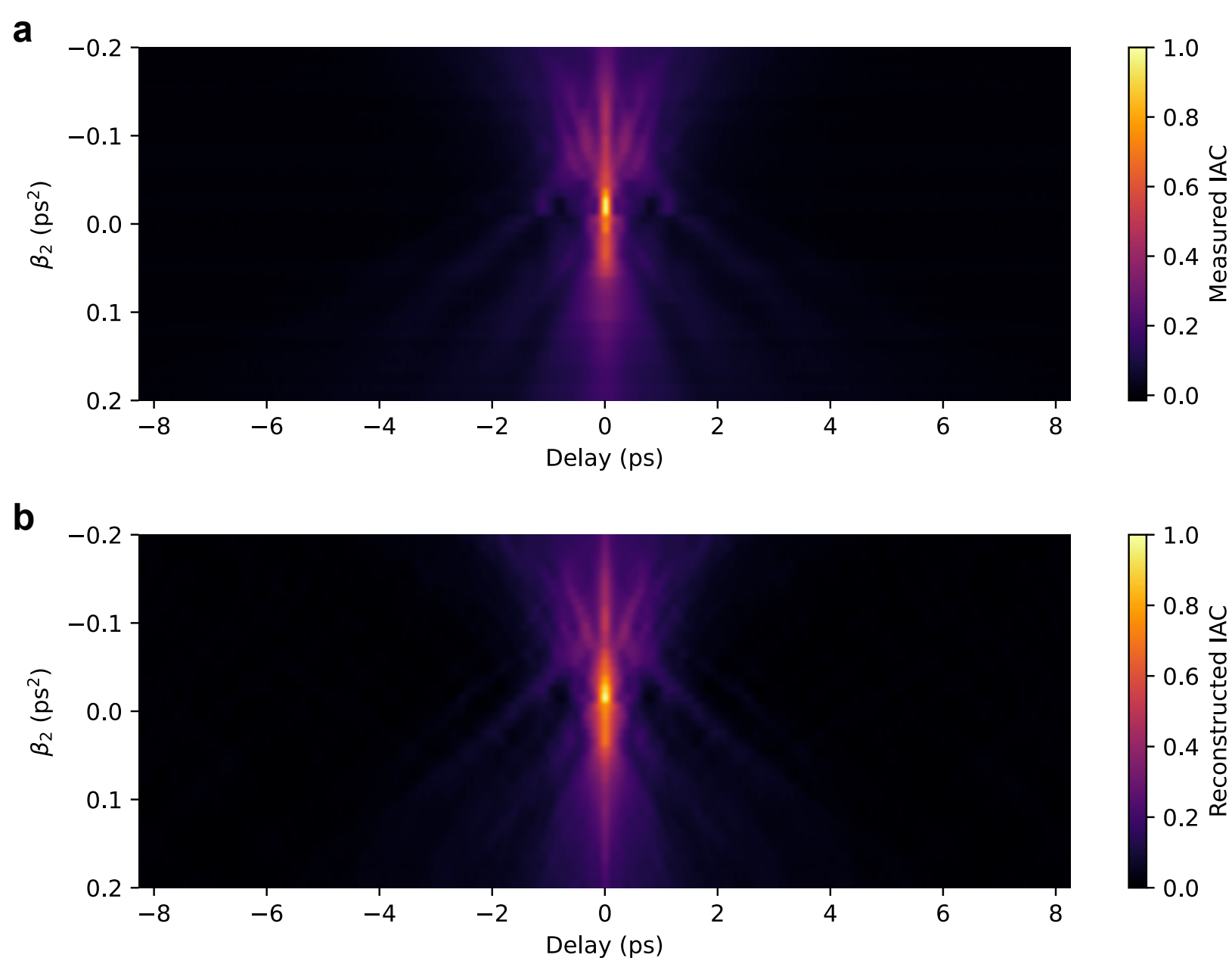

Figure S22. **a,** Measured and **b,** reconstructed intensity autocorrelation function, shown as a function of dispersion β_2 and delay.

S9. Simulation and additional experimental results of supercontinuum generation

In this section, we present simulations of supercontinuum generation based on the nonlinear Schrödinger equation (NLSE) [74, 75]. The input pulse is taken from the simulated output of the Mamyshev oscillator after chirp compensation (Fig. S6 a). While the simulated pulse in Fig. S6 a is compressed to its minimal width, in practice, the connecting optical fiber may slightly under- or over-compensate the chirp, since pulse compression is experimentally optimized by adjusting the fiber length in ∼1 m steps. To account for this, we also include a small residual chirp in the simulated input pulse. The on-chip average power is set to approximately 18 mW (corresponding to a peak power of ∼450 W), based on experimental estimates that take into account the input coupling loss. The nonlinear coefficient is calculated as a function of wavelength using the simulated effective mode area, and a propagation loss of 5 dB/m is assumed for the waveguide. The dispersion of the Si_3N_4 waveguide (Fig. 4e in the main text) is extracted from the broadband mode effective index simulation, obtained in COMSOL Multiphysics with the finite element method. The simulated waveguide cross-section is set to 2.07 µm × 0.70 µm, based on SEM measurements (Fig. 4 c in the main text). The generated visible light components below 600 nm measured on the OSA and observed with the naked eye during the

experiment may result from modal-phase-matched third-harmonic generation, which is not modeled here.

Figure S23 shows the simulated supercontinuum spectrum in a 43.7 mm dispersion-engineered Si_3N_4 waveguide, exhibiting good agreement with the measured spectrum (Fig. 4d in the main text). Minor discrepancies may arise from uncertainties in the Si_3N_4 refractive index or variations in waveguide height across different samples. We also experimentally investigated supercontinuum generation in a longer Si_3N_4 waveguide (175 mm) with the same cross-section. The corresponding measured and simulated spectra are presented in Fig. S24 a and b, respectively. Compared to the 43.7 mm device, the 175 mm waveguide shows enhanced spectral broadening toward both shorter and longer wavelengths, though likely at the expense of reduced coherence. Figure S24 c illustrates the simulated spectral evolution along the propagation length. The results indicate that the 43.7 mm waveguide length approximately corresponds to the onset of octave-spanning supercontinuum generation, with the initial section contributing to compensating for the residual chirp of the input pulse.

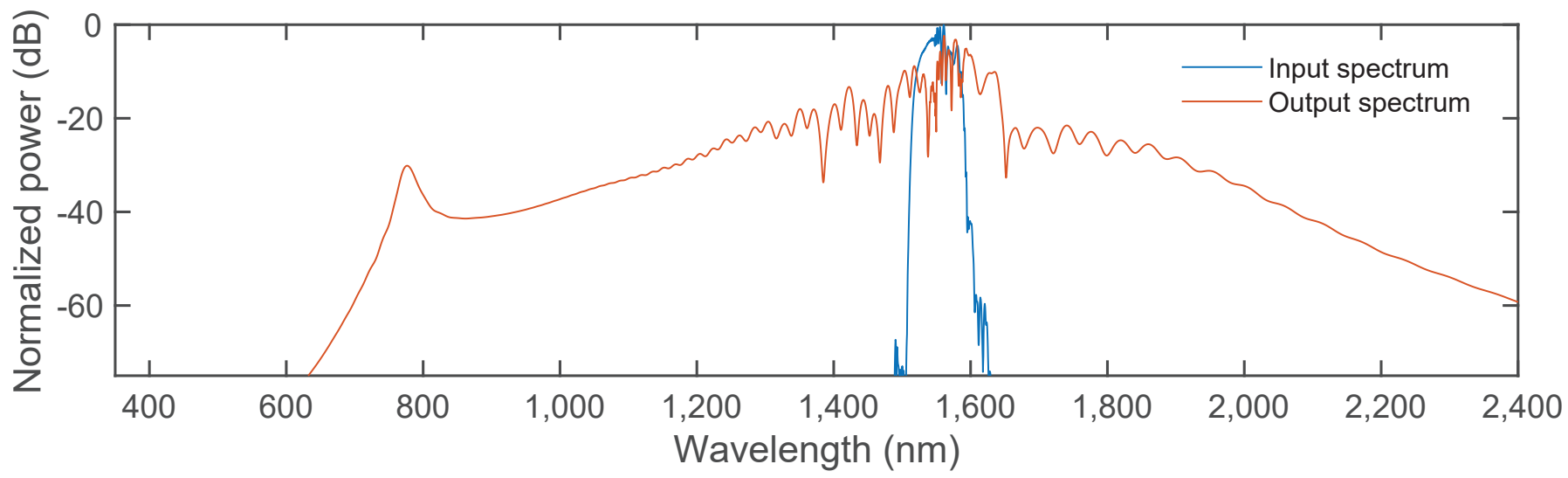

Figure S23. Simulated supercontinuum generated in a 43.7 mm Si_3N_4 waveguide driven by the Mamyshev oscillator.

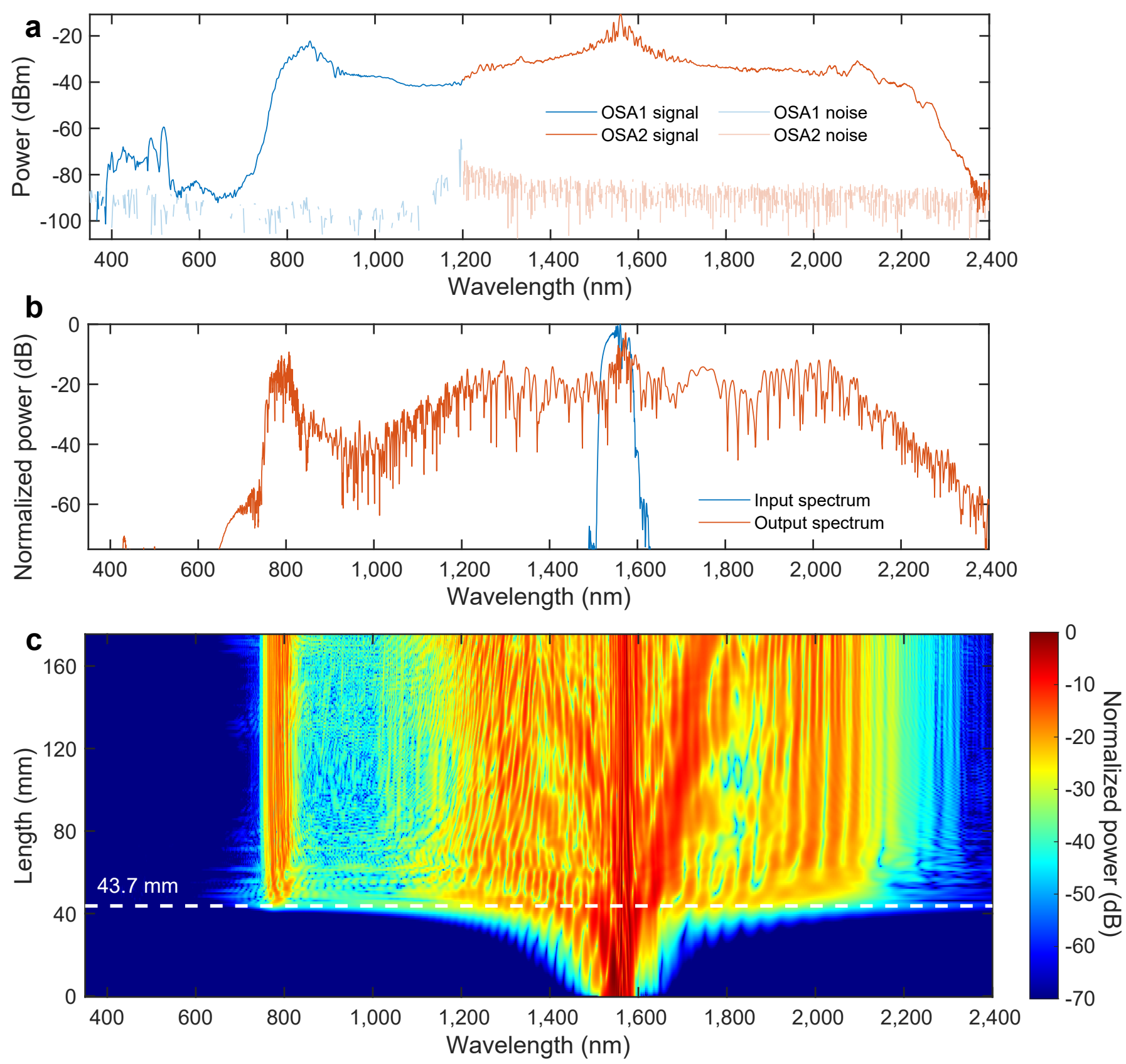

Figure S24. **a,** Experimentally measured supercontinuum generated in a 175 mm Si_3N_4 waveguide. **b,** Simulated supercontinuum generated in a 175 mm Si_3N_4 waveguide driven by the Mamyshev oscillator. **c,** Simulated evolution of spectra over a propagation distance of 175 mm. The dashed line marks the propagation distance of 43.7 mm, corresponding to the spectrum in Fig. S23.

S10. Comparison with state-of-the-art terahertz TDS systems driven by integrated ultrafast sources

We compare our terahertz TDS demonstration with other state-of-the-art lab-scale experiments driven by integrated ultrafast sources, and with commercially available systems, in terms of peak dynamic range and bandwidth (Fig. S25, Table S4). For completeness, in Table S4, we have also included the comparison with several lab-scale experiments using table-top laser systems. The performance of our integrated Mamyshev oscillator-driven terahertz TDS system is superior to all existing terahertz TDS systems driven by integrated ultrafast sources, and is on par with commercial systems.

Table S4: Comparison of state-of-the-art terahertz TDS demonstrations

**Reference**	**Ultrafast optical source**	**Peak dynamic range (dB)**	**Bandwidth (THz)**
**This work**	Integrated MLL	90	5
Integrated source demonstrations			
[76]	Semiconductor MLL	45	0.6
[77]	Semiconductor MLL	65	0.9
[78]	Semiconductor MLL	60	1
[79]	Semiconductor MLL	56	1.4
[80]	Semiconductor MLL	68	1.6
[81]	Semiconductor MLL	65	1.1
[82]	Semiconductor MLL	70	1.2
[83]	Semiconductor MLL	90 (6.5 h avg.)	1.6
[84]	Microcomb	40	0.6
Commercial systems			
Menlo Systems Tera K15	Fiber-based MLL	100	6
Menlo Systems Tera ASOPS	Fiber-based MLL	70	4.5
Toptica TeraFlash pro	Fiber-based MLL	100	6
Toptica TeraFlash smart	Fiber-based MLL	80	4.5
TeraView TeraPulse Lx Sample Chamber	Fiber-based MLL	95	6
TeraView TeraPulse Lx PolyScan Head	Fiber-based MLL	80	4
Advantest TAS7500SP	Fiber-based MLL	70	4
Advantest TAS7500SU	Fiber-based MLL	70	7
PNP Tera Prospector	Fiber-based MLL	40	4
BATOP TDS1008	Fiber-based MLL	85	4.5
BATOP TDS1015	Fiber-based MLL	65	1.5
Hamamatsu C12068-01	Fiber-based MLL	50	4
Hamamatsu C12068-02	Fiber-based MLL	50	7
Luna T-Ray 5000 Series HTS40n2	Fiber-based MLL	70	3.5
Luna T-Ray 5000 Series HTS40n3	Fiber-based MLL	55	3.5
Rainbow Photonics TeraSys-ULTRA	Fiber-based MLL	70	20
Rainbow Photonics TeraSys12	Fiber-based MLL	60	12
Laboratory demonstrations using table-top lasers			
[85]	External cavity semiconductor MLL	43	1.4
[86]	Fiber-based MLL	90	4.5
[87]	Fiber-based MLL	100	6.5
[88]	Fiber-based MLL	~ 95	10
[89]	Fiber-based MLL	137	6.5
[90]	Fiber-based MLL	50	6
[91]	Fiber-based MLL	80	20
[92]	Ti:Sapphire MLL	100	6
[93]	Mid-IR optical parametric amplifier	~ 45	~ 20
[94]	Ti:Sapphire MLL & amplifier	~ 55	200
[95]	Ti:Sapphire MLL & amplifier	~ 70	13

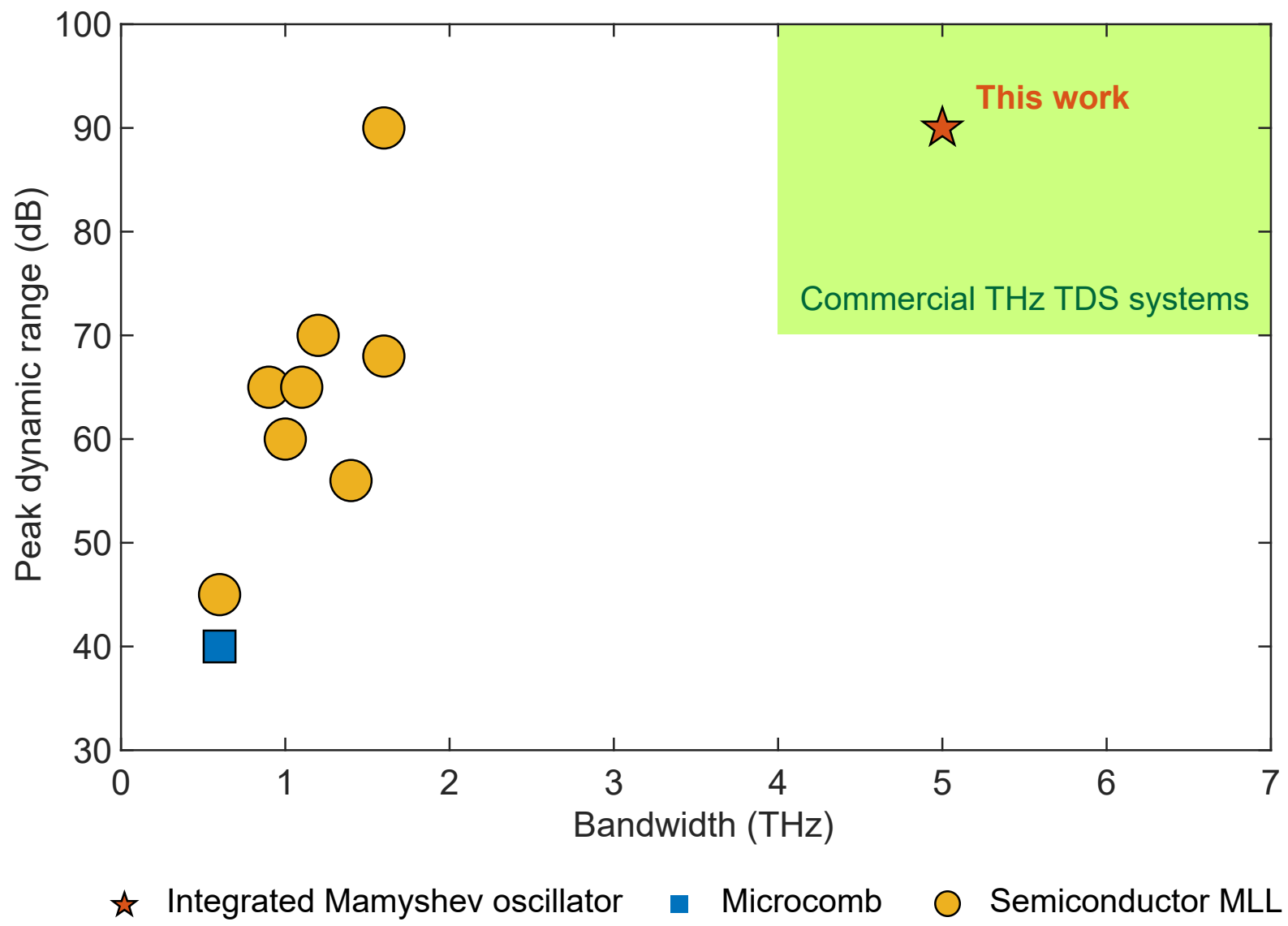

Figure S25. Performance comparison with state-of-the-art terahertz TDS systems driven by integrated ultrafast sources.

S11. Single-pass pulse propagation in the gain section

One interesting question is how the pulse propagates in the gain section if it is isolated from the laser cavity and operated as a standalone amplifier for external pulse inputs. We performed a numerical simulation using the same gain and pump parameters as in Table S2 for a sech-shaped transform-limited pulse input with 10 W on-chip peak power, 3 ps duration and 175.5 MHz repetition rate. The input pulse is injected from the side with 450 mW pump power. Such a pulse train has properties similar to the seed pulses used for initiating the mode-locking when pump-modulation is not employed.

As shown in Figure S27 b, the spectrum is significantly broadened during the propagation in the amplifier with strong nonlinearity. The resulting pulse, shown in Figure S26, has a 1.57 nJ on-chip energy, features a linear chirp and can be compressed to a FWHM of approximately 150 fs when applying the ideal group delay dispersion. Several features in the propagation process such as the highly linear chirp and wave-breaking free propagation are consistent with the self-similar propagation in fiber amplifiers described in [96]. We believe this result shows a complementary approach to on-chip high-energy pulse generation, if suitable external seed pulses are available from other sources such as semiconductor mode-locked lasers[2] or fiber-based lasers. However, pulse coherence, and whether such a hybrid system would require isolators between the source and the amplifier remain to be experimentally investigated.

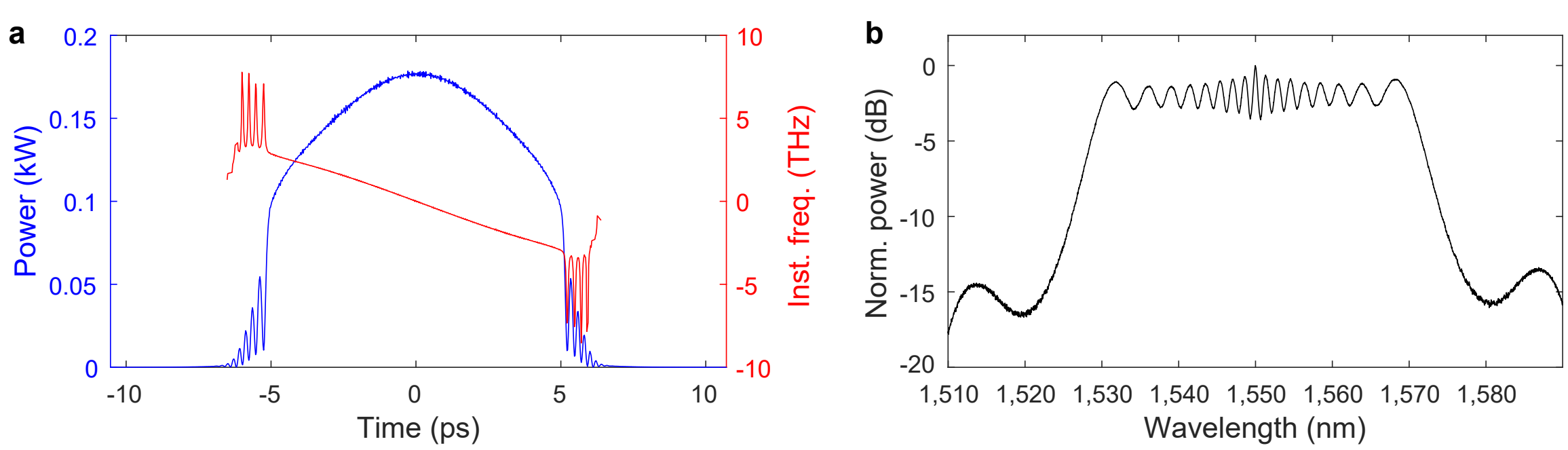

Figure S26. **Single-pass pulse output from the gain section: a,** The pulse output in time domain, showing the instantaneous power and the instantaneous frequency. **b,** The output power spectral density in frequency domain.

[2] We note that existing integrated ultrafast sources typically generate pulses at repetition rates of $\mathcal{O}(1\text{ GHz})$ to $\mathcal{O}(100\text{ GHz})$ featuring relatively low pulse energies (Section S1). Pulse picking and an additional pre-amplification stage would likely be required to achieve a sufficiently low repetition rate to produce a pulse train comparable to the input used in this simulation.

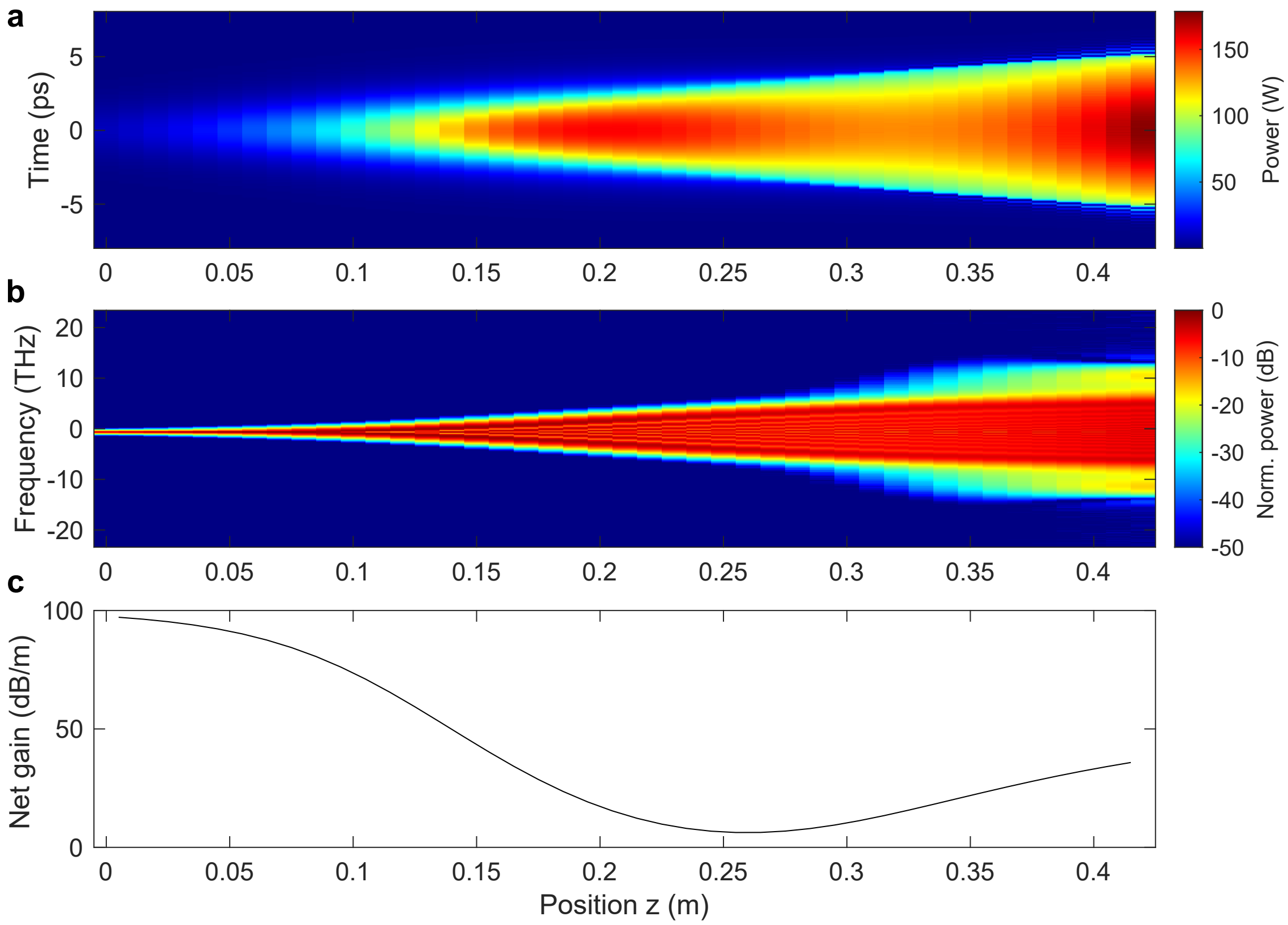

Figure S27. **Single-pass pulse evolution in the gain section: a,** Time domain evolution along the propagation. **b,** Frequency domain power spectral intensity of the pulse along the propagation. **c,** Net gain as a function of the propagation distance.

[1] Guo, Q. *et al.* Ultrafast mode-locked laser in nanophotonic lithium niobate. *Science* **382**, 708–713 (2023).
[2] Suche, H. *et al.* Harmonically mode-locked Ti:Er:LiNbO$_3$ waveguide laser. *Optics Letters* **20**, 596–598 (1995).
[3] Ling, J. *et al.* Electrically empowered microcomb laser. *Nature Communications* **15**, 4192 (2024).
[4] Byun, H. *et al.* Integrated low-jitter 400-MHz femtosecond waveguide laser. *IEEE Photonics Technology Letters* **21**, 763–765 (2009).
[5] Pudo, D. *et al.* Scaling of passively mode-locked soliton erbium waveguide lasers based on slow saturable absorbers. *Opt. Express* **16**, 19221–19231 (2008).
[6] Shtyrkova, K. *et al.* Integrated CMOS-compatible Q-switched mode-locked lasers at 1900 nm with an on-chip artificial saturable absorber. *Optics Express* **27**, 3542–3556 (2019).
[7] Yu, M. *et al.* Integrated femtosecond pulse generator on thin-film lithium niobate. *Nature* **612**, 252–258 (2022).
[8] Hu, Y. *et al.* High-efficiency and broadband on-chip electro-optic frequency comb generators. *Nature Photonics* **16**, 679–685 (2022).
[9] Liu, J. *et al.* Photonic microwave generation in the X- and K-band using integrated soliton microcombs. *Nature Photonics* **14**, 486–491 (2020).
[10] Helgason, Ó. B. *et al.* Surpassing the nonlinear conversion efficiency of soliton microcombs. *Nature Photonics* **17**, 992–999 (2023).
[11] Dmitriev, N. Y. *et al.* Hybrid integrated dual-microcomb source. *Physical Review Applied* **18**, 034068 (2022).
[12] Plant, J. J. *et al.* 250 mW, 1.5 μm monolithic passively mode-locked slab-coupled optical waveguide laser. *Optics Letters* **31**, 223–225 (2006).
[13] Gopinath, J. T. *et al.* 980-nm monolithic passively mode-locked diode lasers with 62 pJ of pulse energy. *IEEE Photonics Technology Letters* **19**, 937–939 (2007).
[14] Gubenko, A. *et al.* High-power monolithic passively modelocked quantum-dot laser. *Electronics Letters* **41**, 1124–1125 (2005).
[15] Carpintero, G., Thompson, M., Yvind, K., Penty, R. & White, I. Comparison of the noise performance of 10 GHz repetition rate quantum-dot and quantum well monolithic mode-locked semiconductor lasers. *IET optoelectronics* **5**, 195–201 (2011).

[16] Rafailov, E. U. *et al.* High-power picosecond and femtosecond pulse generation from a two-section mode-locked quantum-dot laser. *Applied Physics Letters* **87**, 081107 (2005).
[17] Lu, Z. *et al.* 312-fs pulse generation from a passive C-band InAs/InP quantum dot mode-locked laser. *Optics Express* **16**, 10835–10840 (2008).
[18] Nikitichev, D. I. *et al.* High peak power and sub-picosecond fourier-limited pulse generation from passively mode-locked monolithic two-section gain-guided tapered ingaas quantum-dot lasers. *Laser Physics* **22**, 715–724 (2012).
[19] Sun, D. *et al.* Generation of 10-GHz ultrashort pulse and flat optical comb using a semiconductor mode-locked laser. *Optics Communications* **583**, 131655 (2025).
[20] Barbarin, Y. *et al.* Characterization of a 15 GHz integrated bulk InGaAsP passively modelocked ring laser at 1.53 μm. *Optics Express* **14**, 9716–9727 (2006).
[21] Latkowski, S. *et al.* Monolithically integrated 2.5 GHz extended cavity mode-locked ring laser with intracavity phase modulators. *Opt. Lett.* **40**, 77–80 (2015).
[22] Lo, M.-C. *et al.* 1.8-THz-wide optical frequency comb emitted from monolithic passively mode-locked semiconductor quantum-well laser. *Optics Letters* **42**, 3872–3875 (2017).
[23] Cheung, S. *et al.* 1-GHz monolithically integrated hybrid mode-locked InP laser. *IEEE Photonics Technology Letters* **22**, 1793–1795 (2010).
[24] Alloush, M. A. *et al.* RF analysis of a sub-GHz InP-based 1550 nm monolithic mode-locked laser chip. *IEEE Photonics Technology Letters* **33**, 828–831 (2021).
[25] Auth, D., Liu, S., Norman, J., Bowers, J. E. & Breuer, S. Passively mode-locked semiconductor quantum dot on silicon laser with 400 Hz RF line width. *Opt. Express* **27**, 27256–27266 (2019).
[26] Liu, S. *et al.* High-channel-count 20 GHz passively mode-locked quantum dot laser directly grown on Si with 4.1 Tbit/s transmission capacity. *Optica* **6**, 128–134 (2019).
[27] Billet, M. *et al.* Heterogeneous tunable III-V-on-silicon-nitride mode-locked laser emitting wide optical spectra. *Photon. Res.* **12**, A21–A27 (2024).
[28] Davenport, M. L., Liu, S. & Bowers, J. E. Integrated heterogeneous silicon/III-V mode-locked lasers. *Photon. Res.* **6**, 468–478 (2018).
[29] Hermans, A. *et al.* High-pulse-energy III-V-on-silicon-nitride mode-locked laser. *APL Photonics* **6**, 096102 (2021).
[30] Keyvaninia, S. *et al.* III–V-on-silicon anti-colliding pulse-type mode-locked laser. *Optics Letters* **40**, 3057–3060 (2015).
[31] Cuyvers, S. *et al.* Low noise heterogeneous III-V-on-silicon-nitride mode-locked comb laser. *Laser & Photonics Reviews* **15**, 2000485 (2021).
[32] Wang, Z. *et al.* A III-V-on-Si ultra-dense comb laser. *Light: Science & Applications* **6**, e16260–e16260 (2017).
[33] Vissers, E., Poelman, S., de Beeck, C. O., Gasse, K. V. & Kuyken, B. Hybrid integrated mode-locked laser diodes with a silicon nitride extended cavity. *Opt. Express* **29**, 15013–15022 (2021).
[34] Poelman, S., Reep, T., Billet, M. & Kuyken, B. High-power heterogeneously integrated mode-locked laser enabled by a booster amplifier. *Opt. Express* **33**, 54747–54756 (2025).
[35] Kobayashi, M., Nakamura, T., Nakamae, H., Kim, C. & Akiyama, H. Gain-switched pulse generation of 5.3 ps from 30 GHz-modulation-bandwidth 1270 nm DFB laser diode. *Opt. Lett.* **48**, 6344–6347 (2023).
[36] Weng, W. *et al.* Gain-switched semiconductor laser driven soliton microcombs. *Nature communications* **12**, 1425 (2021).
[37] Optilab. Femtosecond Mode-Locked Laser Module – All-PM Fiber Option. https://www.optilab.com/products/femtosecond-mode-locked-laser-module-all-pm-fiber-option (2025). Accessed: 2025-06-22.
[38] Menlo Systems. ELMO Femtosecond Erbium Laser. https://www.menlosystems.com/products/femtosecond-lasers-and-amplifiers/elmo/ (2025). Accessed: 2025-06-22.
[39] Kokyo, Inc. (Symphotony). FL-MLEr-Kit-PM: PM-Type Mode-Locked Er Fiber Laser Kit (1.5 μm). https://en.symphotony.com/products/ultrafast-laser-kit/fl-mler-kit-pm/ (2025). Accessed: 2025-06-22.
[40] AdValue Photonics. 2 μm Mode-Locked Fiber Seed Laser (AP-ML). https://advaluephotonics.com/products/fiber-lasers-amplifiers/picosecond-pulsed/2-%C2%B5m-mode-locked-fiber-lasers-ps-fs/2-micron-mode-locked-fiber-seed-ap-ml/ (2025). Accessed: 2025-06-22.
[41] Cycle GmbH. SONATA – 1030 nm Yb Femtosecond Laser. https://www.cyclelasers.com/femtosecond-lasers/sonata-10-40/ (2025). Accessed: 2025-06-22.
[42] Calmar Laser. 1550 nm Low Jitter Fiber Femtosecond Laser Data Sheet. https://www.calmarlaser.com/docs/datasheets/benchtop/1550%20nm%20Low%20Jitter%20Fiber%20fs.pdf (2020). Accessed: 2025-06-22.
[43] Liu, Z., Ziegler, Z. M., Wright, L. G. & Wise, F. W. Megawatt peak power from a Mamyshev oscillator. *Optica* **4**, 649–654 (2017).
[44] Liu, W. *et al.* Femtosecond Mamyshev oscillator with 10-MW-level peak power. *Optica* **6**, 194–197 (2019).
[45] Lin, D. *et al.* The generation of 1.2 µj pulses from a Mamyshev oscillator based on a high concentration, large-mode-area Yb-doped fiber. *Journal of Lightwave Technology* **40**, 7175–7179 (2022).
[46] Hänsel, W. *et al.* All polarization-maintaining fiber laser architecture for robust femtosecond pulse generation. In *Exploring the World with the Laser: Dedicated to Theodor Hänsch on his 75th birthday*, 331–340 (Springer, 2018).
[47] Agrawal, G. (ed.) *Nonlinear Fiber Optics (Fifth Edition)*. Optics and Photonics (Academic Press, Boston, 2013), fifth edition edn.
[48] Becker, P. C., Anders Olsson, N. & Simpson, J. R. *Erbium-Doped Fiber Amplifiers*. Optics and Photonics (Academic Press, San Diego, 1999).
[49] Giles, C. R. & Desurvire, E. Modeling erbium-doped fiber amplifiers. *Journal of Lightwave Technology* **9**, 271–283 (2002).
[50] Robinson, J. T., Preston, K., Painter, O. & Lipson, M. First-principle derivation of gain in high-index-contrast waveguides.

Opt. Express **16**, 16659–16669 (2008).

[51] Delevaque, E., Georges, T., Monerie, M., Lamouler, P. & Bayon, J.-F. Modeling of pair-induced quenching in erbium-doped silicate fibers. *IEEE Photonics Technology Letters* **5**, 73–75 (1993).

[52] Snoeks, E. *et al.* Cooperative upconversion in erbium-implanted soda-lime silicate glass optical waveguides. *J. Opt. Soc. Am. B* **12**, 1468–1474 (1995).

[53] Gao, M. *et al.* Probing material absorption and optical nonlinearity of integrated photonic materials. *Nature communications* **13**, 3323 (2022).

[54] Afshar, V. S., Monro, T. M. & de Sterke, C. M. Understanding the contribution of mode area and slow light to the effective Kerr nonlinearity of waveguides. *Opt. Express* **21**, 18558–18571 (2013).

[55] Bjork, G. & Nilsson, O. A new exact and efficient numerical matrix theory of complicated laser structures: properties of asymmetric phase-shifted DFB lasers. *Journal of Lightwave Technology* **5**, 140–146 (1987).

[56] Brückerhoff-Plückelmann, F. *et al.* General design flow for waveguide Bragg gratings. *Nanophotonics* (2025).

[57] Fu, W., Wright, L. G., Sidorenko, P., Backus, S. & Wise, F. W. Several new directions for ultrafast fiber lasers. *Optics Express* **26**, 9432–9463 (2018).

[58] Qiu, Z. *et al.* Large-scale photonic chip based pulse interleaver for low-noise microwave generation. *Nature Communications* **16** (2025).

[59] Liu, Y. *et al.* A photonic integrated circuit–based erbium-doped amplifier. *Science* **376**, 1309–1313 (2022).

[60] Lumentum Operations LLC. 600 mW fiber Bragg grating stabilized 14xx nm pump modules. Accessed: 2025-06-17.

[61] Lapointe, J., Coia, C., Dupont, A. & Vallée, R. Passive broadband Faraday isolator for hybrid integration to photonic circuits without lens and external magnet. *Nature Photonics* **19**, 248–257 (2025).

[62] Mosley, C. D. W. *et al.* Asynchronous optical sampling of on-chip terahertz devices for real-time sensing and imaging applications. *Opt. Express* **32**, 27940–27949 (2024).

[63] Franta, D., Franta, P., Vohánka, J., Čermák, M. & Ohlídal, I. Determination of thicknesses and temperatures of crystalline silicon wafers from optical measurements in the far infrared region. *Journal of Applied Physics* **123**, 185707 (2018).

[64] Erdogan, T. Fiber grating spectra. *Journal of Lightwave Technology* **15**, 1277–1294 (2002).

[65] Zhan, J. *et al.* Investigation of backward cladding-mode coupling in bragg gratings implemented on a Si3N4 waveguide platform. *J. Opt. Soc. Am. B* **36**, 3442–3449 (2019).

[66] Chen, Y.-H., Sidorenko, P., Thorne, R. & Wise, F. Starting dynamics of a linear-cavity femtosecond Mamyshev oscillator. *J. Opt. Soc. Am. B* **38**, 743–748 (2021).

[67] Sidorenko, P., Fu, W., Wright, L. G., Olivier, M. & Wise, F. W. Self-seeded, multi-megawatt, Mamyshev oscillator. *Opt. Lett.* **43**, 2672–2675 (2018).

[68] Singh, N. *et al.* Silicon photonics-based high-energy passively Q-switched laser. *Nature Photonics* **18**, 485–491 (2024).

[69] Cao, B. *et al.* Coherence memory and amnesia in a mode-locked Mamyshev oscillator. *Optica* **11**, 1673–1681 (2024).

[70] Rochette, M., Chen, L. R., Sun, K. & Hernandez-Cordero, J. Multiwavelength and tunable self-pulsating fiber cavity based on regenerative SPM spectral broadening and filtering. *IEEE Photonics Technology Letters* **20**, 1497–1499 (2008).

[71] Liu, Z. *et al.* Mamyshev oscillation self-starting mode-locked fiber laser based on a self-feedback amplifying sub-cavity. *Opt. Lett.* **49**, 6397–6400 (2024).

[72] Wang, C., Li, X. & Zhang, S. Automated start-up and extinction dynamics of a Mamyshev oscillator based on a temperature-dependent filter. *Laser & Photonics Reviews* **17**, 2201016 (2023).

[73] Wu, Y. & Chiang, K. S. Mode-selective coupling between few-mode fibers and buried channel waveguides. *Optics Express* **24**, 30108–30123 (2016).

[74] Dudley, J. M., Genty, G. & Coen, S. Supercontinuum generation in photonic crystal fiber. *Reviews of modern physics* **78**, 1135–1184 (2006).

[75] Guo, H. *et al.* Mid-infrared frequency comb via coherent dispersive wave generation in silicon nitride nanophotonic waveguides. *Nature Photonics* **12**, 330–335 (2018).

[76] Merghem, K. *et al.* Terahertz time-domain spectroscopy system driven by a monolithic semiconductor laser. *Journal of Infrared, Millimeter, and Terahertz Waves* **38**, 958–962 (2017).

[77] Balzer, J. C., Tonder, S., Lehr, J. & Koch, M. THz TDS system driven by a commercially available laser diode. In *2019 44th International Conference on Infrared, Millimeter, and Terahertz Waves (IRMMW-THz)*, 1–2 (2019).

[78] Tonder, S. C. *et al.* A compact THz quasi TDS system for mobile scenarios. In *2019 Second International Workshop on Mobile Terahertz Systems (IWMTS)*, 1–5 (2019).

[79] Tybussek, K.-H., Kolpatzeck, K., Faridi, F., Preu, S. & Balzer, J. C. Terahertz time-domain spectroscopy based on commercially available 1550 nm Fabry–Perot laser diode and ErAs:In(Al)GaAs photoconductors. *Applied Sciences* **9**, 2704 (2019).

[80] Kolpatzeck, K. *et al.* System-theoretical modeling of terahertz time-domain spectroscopy with ultra-high repetition rate mode-locked lasers. *Optics Express* **28**, 16935–16950 (2020).

[81] Cherniak, V. *et al.* Direct time axis reconstruction for THz-TDS systems with ultra-high repetition rates. In *2020 45th International Conference on Infrared, Millimeter, and Terahertz Waves (IRMMW-THz)*, 1–2 (2020).

[82] Cherniak, V. *et al.* Compact and inexpensive terahertz system driven by monolithically integrated commercial light sources. In *2021 46th International Conference on Infrared, Millimeter and Terahertz Waves (IRMMW-THz)*, 1–2 (2021).

[83] Cherniak, V., Kubiczek, T., Kolpatzeck, K. & Balzer, J. C. Laser diode based THz-TDS system with 133 dB peak signal-to-noise ratio at 100 GHz. *Scientific Reports* **13**, 13476 (2023).

[84] Peters, L. *et al.* Millimetre-wave comb generated by an optical microcomb. *arXiv preprint* (2025). 2512.05005.

[85] Jördens, C. *et al.* All-semiconductor laser driven terahertz time-domain spectrometer. *Applied Physics B* **93**, 515–520

(2008).

[86] Vieweg, N. *et al.* Terahertz-time domain spectrometer with 90 dB peak dynamic range. *Journal of Infrared, Millimeter, and Terahertz Waves* **35**, 823–832 (2014).

[87] Kohlhaas, R. B. *et al.* Photoconductive terahertz detectors with 105 dB peak dynamic range made of rhodium doped InGaAs. *Applied Physics Letters* **114**, 221103 (2019).

[88] Kohlhaas, R. B. *et al.* Ultrabroadband terahertz time-domain spectroscopy using III-V photoconductive membranes on silicon. *Opt. Express* **30**, 23896–23908 (2022).

[89] Dohms, A. *et al.* Fiber-coupled THz TDS system with mW-level THz power and up to 137-dB dynamic range. *IEEE Transactions on Terahertz Science and Technology* **14**, 857–864 (2024).

[90] Couture, N. *et al.* Compact, low-cost, and broadband terahertz time-domain spectrometer. *Appl. Opt.* **62**, 4097–4101 (2023).

[91] Puc, U., Bach, T., Günter, P., Zgonik, M. & Jazbinsek, M. Ultra-broadband and high-dynamic-range THz time-domain spectroscopy system based on organic crystal emitter and detector in transmission and reflection geometry. *Advanced Photonics Research* **2**, 2000098 (2021).

[92] Yardimci, N. T., Turan, D. & Jarrahi, M. Efficient photoconductive terahertz detection through photon trapping in plasmonic nanocavities. *APL Photonics* **6**, 080802 (2021).

[93] Koulouklidis, A. D. *et al.* Observation of extremely efficient terahertz generation from mid-infrared two-color laser filaments. *Nature communications* **11**, 292 (2020).

[94] Matsubara, E., Nagai, M. & Ashida, M. Ultrabroadband coherent electric field from far infrared to 200 THz using air plasma induced by 10 fs pulses. *Applied Physics Letters* **101**, 011105 (2012).

[95] Singh, A., Pashkin, A., Winnerl, S., Helm, M. & Schneider, H. Gapless broadband terahertz emission from a germanium photoconductive emitter. *ACS Photonics* **5**, 2718–2723 (2018).

[96] Kruglov, V. I., Peacock, A. C., Dudley, J. M. & Harvey, J. D. Self-similar propagation of high-power parabolic pulses in optical fiber amplifiers. *Opt. Lett.* **25**, 1753–1755 (2000).
