# Supplementary material for: High-pulse-energy integrated mode-locked laser using a Mamyshev oscillator
Source: Nature. 2026 Jun 3;654(8117):57–63. doi: 10.1038/s41586-026-10517-4 (PMC13661250; doi:10.1038/s41586-026-10517-4)
Supplement: Supplementary file 2 [file 41586_2026_10517_MOESM2_ESM.pdf]

nature portfolio

Peer Review File

High-pulse-energy integrated mode-locked laser using a Mamyshev oscillator

Corresponding Author: Professor Tobias Kippenberg

Any redactions in this file are there to maintain patient confidentiality, the confidentiality of unpublished data, or to remove third-party material.

This file contains all reviewer reports in order by version, followed by all author rebuttals in order by version.

Version 0:

Reviewer comments:

Referee #1

(Remarks to the Author)
The manuscript by Dr. Qiu and coworkers reports the first photonic integrated circuit-based mode-locked laser employing erbium-doped silicon nitride waveguides. This device overcomes the pulse-energy limitations of previous mode-locked integrated photonic lasers by more than two orders of magnitude, achieving energies in the nanojoule range thanks to the adoption of the Mamyshev architecture. The authors provide a detailed account of the device fabrication, along with a thorough characterization of the oscillator's coherence, pulse compression, phase and frequency noise. Furthermore, the compressed pulses are shown to directly drive, without any further amplification, the generation of a1.5 octave-spanning spectrum in an integrated silicon nitride waveguide.
The results presented are original, technically sound and have the potential to pave the way for future developments in integrated photonic frequency combs, to cite a possible application. The quality of the presentation and the reported data is clear and appropriate, with prior work being adequately cited both in the main manuscript and in the supplementary materials.
Although the manuscript presents outstanding results in the field of integrated photonics, with significant impact on integrated photonic lasers, in my opinion it does not, in its current form, constitute a true breakthrough with relevance beyond the photonics community. For this reason, I do not recommend its publication in Nature. Instead, I believe it would be more appropriately suited for Nature Photonics. The demonstrated ability to directly drive a spectrum spanning more than an octave is indeed highly intriguing for self-referencing; however, this is not achieved here, as a frequency comb is not demonstrated. Such a demonstration could have substantially extended the impact of the present work on the fields of metrology and optical spectroscopy, although it would likely remain borderline for publication in Nature. To effectively address the broad scientific audience targeted by Nature, the manuscript should directly demonstrate a strong impact on technologies or scientific disciplines beyond photonics, introducing a paradigm shift across several fields.

Referee #2

(Remarks to the Author)
This manuscript presents the development of an integrated Mamyshev oscillator on a photonic chip, aiming to translate the powerful pulse-energy scaling capabilities of this cavity design from fiber platforms to a compact, on-chip format. The work addresses a significant challenge in integrated ultrafast photonics by attempting to implement the Mamyshev mechanism's nonlinear switching (acting as a saturable absorber equivalent) within a monolithic waveguide structure. This approach to achieving high-energy ultrashort pulses on-chip is highly relevant and timely, which can be published by high-profile technical journals. However, I do not recommend this manuscript to be published in Nature for the following concerns:

1. It's claimed to have "demonstrated a fully integrated Mamyshev oscillator architecture MLL based on a wafer-scale fabricated erbium-doped Si_3N_4 photonic chip", while two high-power, SMF-coupled 1480-nm pump lasers were still required in the experiment.
2. Furthermore, it's also required to initiate mode-locking in the integrated Mamyshev oscillator by feeding a commercial fiber MLL (Menlo ELMO High Power) as the seed source, delivering 51 fs FWHM pulses at a 100-MHz repetition rate and

approximately 100 mW average power, which is quite a high-quality fiber MLL by itself, comparing with the output 1 was merely compressed down to 187 fs FWHM with the optimized group delay dispersion.
3. How sensitive would the performance of that seed source affect the output of the integrated Mamyshev oscillator?
4. Why was 1480 nm selected instead of the conventional 980 nm pump for erbium gain? While their custom EDFA consisted of two 980/1550 nm WDMs (Thorlabs), a 980 nm pump laser diode (Aerodiode), and a short (~50 cm) section of highly doped erbium fiber (LIEKKI ER80-4/125-HD-PM).
5. In Fig.1(c), how ddid the intra-cavity pulse energy evolve?
6. What's the dispersion coefficient of the integrated erbium waveguide? What's the total dispersion of the cavity? Can dispersion be precisely engineered during fabrication? Is intra-cavity dispersion management feasible to generate sub-100 fs pulses directly without external compression?
7. What's the reflected ratio of the two WBGs in the experiment? How's the tunability of them by using the microheater? Is it possible to achieve self-starting by tuning the WBGs?
8. Based on the simulation results in Fig. S8, ~18 nm separation yields higher compressed peak power. Why were 42 cm cavity length and 9.8 nm WBG separation chosen experimentally? In addition, how about the influence of different parameters on the output pulse energy? How do other parameters (such as filter bandwidth and output ratio) impact the performance? In these terms, does the integrated Mamyshev oscillator follow the same theoretical principles as fiber-based systems?
9. Can WDM CIR ISO also be integrated? What are the possibilities and challenges of achieving full integration of the entire setup in the future?
10. What's the scalable repetition rate of this integrated Mamyshev oscillator? While the repetition rate frep was 175.5 MHz, and the seed source was at 100-MHz repetition rate. Please elaborate the difference between the two.
11. What factors limit the pulse energy so far?
12. How's the beam quality M2 of the output beam?
13. Is simultaneous operation of 26 cavities feasible on a 2 × 1.1 cm^2 chip? How significant are the thermal effects, and must they be actively controlled?
14. What are the primary challenges in realizing integrated directly passive mode-locked lasers?
15. (Minor) Formatting suggestions:
1) Fig.1(d), add the scale bar.
2) Fig.2(a) figure caption, define "WDM" (wavelength-division multiplexing).
3) Fig S1, label schematic elements with corresponding references.

Referee #3

(Remarks to the Author)
I co-reviewed this manuscript with one of the reviewers who provided the listed reports.

Referee #4

(Remarks to the Author)
This is a strong and carefully characterized system result: an integrated Mamyshev oscillator that reaches nanojoule-class energies at 175 MHz with sub-200 fs compressed pulses, narrow repetition-rate/optical linewidths, and direct supercontinuum generation. It is being somewhat oversold however.

Major concern: system complexity for start-up.

The reported device achieves impressive nJ-class pulses. However, realizing this performance requires a substantial off-chip seeding complexity (fiber MLL, programmable filter, EDFA, synchronized pulse picker) to inject a single 15–45 pJ pulse, or, alternatively, an extended-cavity Q-switching arrangement with higher pump power (external modulator + circulator). This is in tension with the paper's "reduced-complexity, fully integrated" frame. A stronger result (which would undoubtable be of worthy of broad dissemination, e.g. in Nature) would demonstrate robust self-start without external seed or extended cavity.

Comparison claims. The manuscript states ">2 orders of magnitude" improvement over prior PIC ultrafast sources; the authors' own table appears closer to 1 order (15x). Please reconcile the headline with the table (define exactly which prior class is being compared).

Summary of the key results

- A Mamyshev oscillator is implemetned on Er doped SiN PIC, which uses a complex off-chip seeding setup.
- Dual 1480 pumps provide ~1 W each. A third mode locked laser seed starts with a single pulse. (alternative Q switch is also shown)
- ~20 dB BW of ~50-60nm
- 20 RT for build-up
- ~1 nJ pulse energy is an order of magntiude larger than previous PIC approaches.

Suggested improvements:
- Language should be softened? "seed-assisted integrated mamyshev oscillator achieving nJ-class pulses"?

- Self starting is essential. I don't think lasers need to be integrated, they can be off-chip, but if the thing needs very complex off-chip optics (MLL or circulators, modulators etc.), then the reduced complexity argument can't be made.
- I would be interested in knowing what the single-pass characteristics are.

Questions that I believe need to be answered in the text before proceeding further:
- Does pump-to-output sum the two ports?
- What is the start probability vs pump power, and the distribution of time-to-lock?
- What minimum seed energy (on-chip) reliably starts ML?
- If you route the shaped seed through the doped section without feedback/filters, then compress: what are the output energy, spectrum, etc.?
- Back-reflections: specify the target return-loss needed for self-start, with supporting sim/measurement (today's parasitics appear ~-30 dB; what level enables noise-start?). Outline a realistic path (angled/AR edge couplers, etc.) and expected margin.

--

I believe that as as written, the reliance on complex off-chip optics for start-up undercuts the "fully integrated, reduced-complexity" claim expected for Nature. Demonstrating robust self-start (or tempering the claims and tightening quantitative comparisons) would lift the work to that bar. If it turns out that self starting of these oscillators is simply not possible (e.g. by the detailed analysis I've requested above), then the impact of this work would be significantly lessened and so this needs to be understood (via demonstrating self-starting oscillator) before publication.

Version 1:

Reviewer comments:

Referee #1

(Remarks to the Author)
The revised version represents a substantial improvement over the original submission, both in terms of technical completeness and system-level relevance. Such an extensive and careful revision is really appreciated.
As I noted in my previous review (Reviewer #1), the work is of excellent quality both scientifically and technologically. The demonstration of a self-starting, turn-key Mamyshev oscillator fully integrated on chip (except for the pump sources), delivering nJ-level femtosecond pulses at a repetition rate of 175 MHz, constitutes a clear and significant advance over the current state of the art in integrated ultrafast photonics. The added experimental evidence addressing self-starting operation convincingly resolves one of the major concerns raised in the first review process.
I also acknowledge the effort made to strengthen the breadth of impact by demonstrating an application in THz-TDS. This addition shows that the source can directly enable demanding system-level applications without external amplification, and the reported THz performance (bandwidth and dynamic range) is indeed competitive with established tabletop commercial systems. That said, my original concern regarding the scope of interdisciplinarity is not fully removed. While the THz-TDS experiment is a meaningful demonstration, in my view it showcases the capabilities of the laser source, rather than a fundamentally new result in THz science or in a discipline outside photonics. The core innovation and scientific depth of the manuscript are still strongly in ultrafast optics and integrated photonics. Nevertheless, I recognize that Nature often values the introduction of genuinely new technological capabilities that have the potential to propagate across multiple disciplines. Some of the references provided in the authors' response and published on Nature support this view. In this sense, the revised manuscript now makes a stronger and more credible case that the demonstrated platform can truly act as an enabling technology for applications in spectroscopy, sensing, and materials characterization beyond the photonics community.
Overall, I consider the manuscript scientifically sound, technically mature, and of high impact. While its suitability for Nature remains borderline in terms of interdisciplinarity, an aspect which the Editors are best positioned to evaluate, I believe that the combination of the unprecedented on-chip pulse energy, turn-key operation, and the demonstrated system-level application makes the manuscript worthy of consideration by Nature if the other reviewers share this positive assessment.

Referee #2

(Remarks to the Author)
We appreciate the authors for their detailed response to the previous reviewers' comments. The revised manuscript shows improvements compared to the previous version, particularly in the self-starting scheme and the demonstration of the terahertz measurement application. The further simplification of the laser start-up process and the further verification of the laser performance all contribute to a more solid foundation for this work. However, we still have the following questions/comments:

1) Regarding “New Tool”: This study is another implementation mode for generating ultrafast laser with comparable performance, i.e., on-chip integration, while the integrated photonics platform itself is not novel and has been widely reported. Regarding the implementation principle, the authors acknowledge that “We believe that this integrated Mamyshev oscillator should follow the same theoretical principles as the fiber counterparts,” although they add, “However, an integrated Mamyshev oscillator operates in a substantially different parameter regime,” without further in-depth research to reveal new physical insights or general design principles.

2) Regarding “New Capability”: The direct cavity-output pulse energy does represent a significant improve (2 order) over prior integrated sources, while this performance advantage may not be unique. Other integrated source schemes could potentially achieve similar or superior performance for application through subsequent on-chip amplification, which may not introduce additional complexity.

3) Regarding “New Application”: The authors list a series of applications for mode-locked ultrafast lasers (such as ranging, imaging, and spectroscopy), and add a new application on terahertz spectroscopy using their laser source, demonstrating its interdisciplinary practicality. The terahertz spectroscopy experiment remains, in essence, a demonstration of a new source's functionality, and does not move beyond the established paradigm. It does not demonstrate a unique, transformative impact on new applications.

Other questions:
1. We thank the authors for their further explanation of the performance design, including parameters such as cavity length, dispersion, filters, and output coupling ratio, etc. In the rebuttal letter, the authors emphasize that their research focuses on high-energy integrated on-chip laser sources, and claimed that the integrated Mamyshev oscillator operates in a “new parameter regime” compare to the fiber Mamyshev oscillator. In this regard, for the first on-chip integrated Mamyshev study, I suggest that the authors provide more general design analysis or simulation guidelines which would be crucial for advancing this research direction, rather than choosing one method for demonstration due to limitations in experimental conditions.

2. In S1, “selection of commercially available low-power table-top mode-locked lasers (MLLs) is also listed for reference”. Comparing only to low-power commercial table-top lasers is of limited value. We suggest a direct and honest comparison with the best-reported performance of fiber mode-locked lasers (including fiber Mamyshev oscillators) on key metrics like pulse energy and duration to objectively contextualize the advantages and limitations of this work. A similar issue exists in S14 for the terahertz TDS system performance. Comparisons with the best-reported systems should be included.

3. At line 213, please explicitly state the method for calculating thickness from the time delay. What’s the minimum measurable thickness of the system?

4. Formatting suggestions:
1) In the SI, the page number “S1” and the section label “S1” are confusing. Please adjust.
2) Please ensure all SI sections are cited in the main text and consider reordering them to follow the citation sequence.
3) Please unify the reference formatting between the main text and the SI.

Referee #3

(Remarks to the Author)
I co-reviewed this manuscript with one of the reviewers who provided the listed reports.

Referee #4

(Remarks to the Author)
The authors have carried out a substantial revision that addresses my principal concerns. In particular:

The most critical issue, the reliance on an external ML laser for initiation has been resolved by demonstrating a pump-current-modulation self-seeding approach that eliminates all off-chip optical components for start-up. The supporting data seem to be fairly convincing. The misleading full integrated language has been appropriately corrected.

The addition of the THz TDS demonstration provides a concrete application that strengthens the case.

The remaining technical characterizations have all been provided and are satisfactory.

I have two minor remaining comments:

1. The authors use "self-starting" and "self-seeded", is this the right terminology for an electrically initiated process?

2. The headline compressed pulse duration of 147 fs is achieved using ~10 m of external single-mode fiber. The authors discuss on-chip compression via chirped Bragg gratings as feasible but do not demonstrate it. I would appreciate a brief explicit statement in the main text noting this... (if i understood it correctly)

These are minor points that do not affect the core conclusions. I am now fairly supportive of publication in Nature.

Open Access This Peer Review File is licensed under a Creative Commons Attribution 4.0 International License, which permits use, sharing, adaptation, distribution and reproduction in any medium or format, as long as you give appropriate credit to the original author(s) and the source, provide a link to the Creative Commons license, and indicate if changes were made.
In cases where reviewers are anonymous, credit should be given to 'Anonymous Referee' and the source.
The images or other third party material in this Peer Review File are included in the article's Creative Commons license, unless indicated otherwise in a credit line to the material. If material is not included in the article's Creative Commons license and your intended use is not permitted by statutory regulation or exceeds the permitted use, you will need to obtain permission directly from the copyright holder.
To view a copy of this license, visit https://creativecommons.org/licenses/by/4.0/

Executive summary of changes

We sincerely thank the reviewers for their thorough and thoughtful evaluation of our manuscript. We greatly appreciate the constructive feedback, which has helped us improve the clarity, rigor, and overall quality of the work. In response to the reviewer's comments, we have carefully revised the manuscript.

In summary, since the original submission, we have generated substantial new experimental evidence that we hope can address the two concerns brought up: system-level practicality and breadth of impact), while preserving the core technical advance (nJ-class pulses from a wafer-fabricated, integrated Mamyshev oscillator):

- **Turn-key self-seeded initiation**: In our revised manuscript we now demonstrate that without any external ultrafast seed source or off-chip optics we can self-seed the Mamyshev oscillator. Reliable self-starting is achieved by a readily automized procedure: momentary application of a pump-current modulation. After initiation, modulation is then disabled and the oscillator runs passively and autonomously in steady-state.
  The robustness of this method is quantified: we provide start probability and time-to-lock statistics versus pump power, characterized repeatability across many independent start events and convergence to the same steady-state spectra for identical cavity settings.
- **Realization of a concrete cross-disciplinary application demonstration:** It was requested to demonstrate the use of our Mamyshev oscillator in an application beyond photonics. Towards this end we demonstrate that our integatrated Mamyshev oscillator enables the construction of a **terahertz time-domain spectroscopy (THz-TDS)** experiment, achieving 90 dB dynamic range and 5 THz bandwidth – on par with legacy fs laser systems - and we demonstrate representative sensing tasks (thickness measurements and material identification via chemical THz absorption spectrum).

Moreover, we have:

- Clarified claims and tightened benchmarking. We remove ambiguous language (e.g., "fully integrated" in a way that could imply on-chip pumps), explicitly define the comparison class underlying the ">2 orders of magnitude" claim (PIC-based ultrafast sources), and reconcile all headline statements with the updated comparison table.
- Added missing practical characterizations requested by reviewers. We add or clarify: output beam quality (M2), dispersion coefficients and cavity dispersion, WBG reflectivity and heater tunability, intra-cavity energy evolution, and single-pass pulse propagation in the gain-section.

In the following, we provide a detailed, point-by-point response to the reviewer's comments. For clarity and ease of reading, the reviewer's original comments are shown in blue, our responses are provided in black, and the corresponding actions taken for the manuscript are highlighted in red.

Referee #1 (Remarks to the Author):

"The manuscript by Dr. Qiu and coworkers reports the first photonic integrated circuit-based mode-locked laser employing erbium-doped silicon nitride waveguides. This device overcomes the pulse-energy limitations of previous mode-locked integrated photonic lasers by more than two orders of magnitude, achieving energies in the nanojoule range thanks to the adoption of the Mamyshev architecture. The authors provide a detailed account of the device fabrication, along with a thorough characterization of the oscillator's coherence, pulse compression, phase and frequency noise. Furthermore, the compressed pulses are shown to directly drive, without any further amplification, the generation of a 1.5 octave-spanning spectrum in an integrated silicon nitride waveguide.

The results presented are original, technically sound and have the potential to pave the way for future developments in integrated photonic frequency combs, to cite a possible application. The quality of the presentation and the reported data is clear and appropriate, with prior work being adequately cited both in the main manuscript and in the supplementary materials."

Answer: We sincerely thank Referee #1 for the thorough evaluation of our manuscript and for recognizing the originality, technical soundness, and the order-of-magnitude improvement in pulse energy achieved by our device.

Referee #1: "Although the manuscript presents outstanding results in the field of integrated photonics, with significant impact on integrated photonic lasers, in my opinion it does not, in its current form, constitute a true breakthrough with relevance beyond the photonics community. For this reason, I do not recommend its publication in Nature. Instead, I believe it would be more appropriately suited for Nature Photonics."

Answer: We thank the reviewer for recognizing that our work presents "outstanding results in the field of integrated photonics, with significant impact on integrated photonic lasers." However, we respectfully disagree with that it does not yet constitute a breakthrough with relevance beyond the photonics community and would therefore be better suited for Nature Photonics.

Since their invention in the 1980s, mode-locked lasers have consistently enabled transformative advances far outside photonics. Importantly, the first Nobel Prize related to mode-locked lasers was awarded in Chemistry rather than Physics, because ultrashort pulses gained insights into ultrafast chemical and biological dynamics [R1.1]. Mode-locked lasers now underpin a broad range of disciplines and technologies, including time-of-flight metrology [R1.2], terahertz non-destructive testing [R1.3], photonic radar [R1.4], corneal surgery [R1.5], precision material processing [R1.6], and deep-tissue imaging [R1.7], and thus have had pervasive impact well beyond photonics. Frequency-stabilized mode-locked lasers have further expanded these impacts through optical frequency combs, enabling precision optical metrology [R1.8], high-resolution spectroscopy [R1.9], dual-comb ranging [R1.10] and spectroscopy [R1.11], and high-accuracy time transfer [R1.12]. **One new experimental demonstration and more explicit examples will be detailed in the following reply.**

Precisely because of these far-reaching applications, there has been a long-standing global effort to miniaturize mode-locked lasers to chip-scale devices. Yet, despite intense research, including major international initiatives such as DARPA's DODOS program in 2014 and the EIC Pathfinder FEMTOCHIP project in 2021, photonic integrated ultrafast sources generally fall significantly short of bulk or fiber oscillators in the combination of pulse energy, pulse duration, and noise performance required for demanding applications (see Supplementary Information S1). We view the central contribution of this work as removing this long-standing practical bottleneck by demonstrating a wafer-fabricated, photonic-integrated mode-locked laser delivering nanojoule-class pulse energies with sub-150 fs compressibility and low-noise operation.

We therefore believe that the breakthrough demonstrated here is not only of significance to integrated photonics, but also unlocks cross-disciplinary opportunities with broad scientific and technological relevance. As one specific example we have in our revised manuscript demonstrated the use of our integrated Mamyshev oscillator for a Terahertz Time domain spectrometer, accessing the spectral region between optical and RF frequencies, which is used for numerous non-contact chemical detection and inspection techniques. For these reasons, we feel that the work, after revision, shall meets the criteria for publication in *Nature*.

Referee #1: “The demonstrated ability to directly drive a spectrum spanning more than an octave is indeed highly intriguing for self-referencing; however, this is not achieved here, as a frequency comb is not demonstrated. Such a demonstration could have substantially extended the impact of the present work on the fields of metrology and optical spectroscopy, although it would likely remain borderline for publication in Nature.”

Answer: We thank the reviewer for highlighting that octave-spanning spectra that we have attained is ‘intriguing for self-referencing’. Indeed, our work is the first to realize an integrated mode locked laser that can generate an octave spanning spectrum via supercontinuum generation. It can, in principle, enable self-referencing and thereby broaden impact in optical metrology and spectroscopy. We agree with this broader vision. However, we respectfully submit that demonstrating a fully self-referenced comb is not commensurate with the scope of the present manuscript, whose central contribution is the first wafer-fabricated, photonic-integrated Mamyshev-oscillator engine delivering nanojoule-class pulses that can be dechirped to sub-150 fs. The realization of a self-referenced photonic integrated frequency comb is in itself, a decades prevailing challenge, that has to date not been met (apart from using Kerr combs that do not employ supercontinuum generation).

In practice, self-referencing is a demanding system-level experiment that typically requires a dedicated setup (e.g., a free-space f-2f interferometer) and stringent suppression of technical noise, in our case particularly pump intensity noise.

Even in the microcomb community that have a more than 15-year history, self-referencing demonstrations have been rare. A representative example is the work by Papp et al. (*Nature*, "An optical-frequency synthesizer using integrated photonics"), which is widely regarded as a heroic experiment: it relied on substantial external infrastructure (including external lasers and amplification) and multiple integrated components. The self-referencing itself constituted a major technical achievement rather than an incremental add-on. **We therefore view a comparable self-referencing experiment as a substantial standalone effort that would shift the focus of the present manuscript away from establishing the integrated high-pulse-energy ultrafast oscillator and toward a specialized optical-frequency-metrology implementation.**

We would also like to emphasize that prior PIC-based mode-locked sources have generally lacked the pulse energy needed to realistically support octave broadening and self-referencing, whereas our results provide the missing high-pulse-energy ultrafast engine required for such a system.

In addition, we would like to note that we experimentally demonstrated the frequency-comb structure of the laser output across its emission bandwidth, by heterodyne detection with a continuous-wave local oscillator. Clear RF beat notes at the comb-line spacing are observed, even though we do not close the self-referencing loop. This is described in the main text and expanded in the added Supplementary Information S13. In fact, our work has established that the Mamyshev oscillator produces an optical frequency comb and we have measured the individual comb teeth.

Action taken:

We revised the main text and added Supplementary Information S13, to expand on the heterodyne characterization of the frequency-comb structure of the laser output on frequency domain.

Referee #1: “To effectively address the broad scientific audience targeted by Nature, the manuscript should directly demonstrate a strong impact on technologies or scientific disciplines beyond photonics, introducing a paradigm shift across several fields.”

Answer: We thank the referee for this thoughtful perspective and for clearly articulating what is needed for a broad *Nature* audience. Mode-locked lasers underpin a wide range of technologies and scientific disciplines beyond photonics, including femtochemistry, precision ranging, optical clocks, micromachining, and biomedical imaging. We agree that, in the original submission, this cross-disciplinary relevance was primarily motivated rather than directly demonstrated.

In the revised manuscript, we therefore add a concrete application beyond photonics: terahertz time-domain spectroscopy (THz-TDS), directly driven by our integrated Mamyshev oscillator. Terahertz radiation offers a unique combination of properties: it can penetrate many optically opaque materials, is non-ionizing (and therefore safer than X-rays), provides higher spatial resolution than millimeter waves, and exhibits rich spectral fingerprints of molecules. These features make THz waves attractive for **non-destructive testing, security screening, biomedical and pharmaceutical inspection, chemistry and materials research** [R1.3, R1.13-1.17]. Specifically, terahertz can detect defects in Lithium-ion battery electrodes for industrial non-destructive testing [R1.17]; it can detect concealed weapons and identify explosives for security screening [R1.18]; it can assist dental caries diagnosis for biomedical imaging [R1.19]; it can characterize crystalline properties of drugs for pharmaceutical inspection [R1.20]; it can detect foreign objects and adulteration in food products [R1.21]; it can locate hidden defects in ancient paintings for art conservation [R1.22]; it can identify damage in conductive traces within electronic chip packages [R1.23].

The most widespread terahertz instruments and sensors nowadays are terahertz TDS system, where an ultrafast light source enables the generation and optical sampling of terahertz wave through photoconductive antennas or nonlinear optical process ([R1.13, R1.24]). However, existing terahertz TDS systems typically rely on bulky and costly solid-state or fiber MLLs ([R1.13, R1.17]) to provide the ultrafast high-power pulses, making the development of small, portable and cost-effective terahertz TDS system challenging. **The mode-locked laser is well known to limit the deployment of THz technology**, quoting a few words from review papers on professional journals:

“The **heart of any THz-TDS system** is an ultrafast laser oscillator or amplifier.”

— Jens Neu, and Charles A. Schmuttenmaer. "Tutorial: An introduction to terahertz time domain spectroscopy (THz-TDS)." Journal of Applied Physics 124.23 (2018).

“Laser-based THz generation **depends critically on available pump laser sources. A current challenge for THz generation based on lasers is to either provide a low-cost source** or provide a unique application justifying the cost. **This is of particular importance to industrial applications.**”

— Alfred Leitenstorfer, et al. "The 2023 terahertz science and technology roadmap." Journal of Physics D: Applied Physics 56.22 (2023): 223001.

The performance of a THz-TDS system depends directly on the driving laser, and in particular on sufficiently short optical pulses to for broad THz generation bandwidth and fast photocarrier gating, and sufficiently high power (and corresponding pulse energy) to achieve high signal-to-noise ratio and dynamic range. Our photonic integrated Mamyshev oscillator excels at all these aspects and can be the high-performance yet low-cost drop-in replacement for expensive and massive traditional lasers.

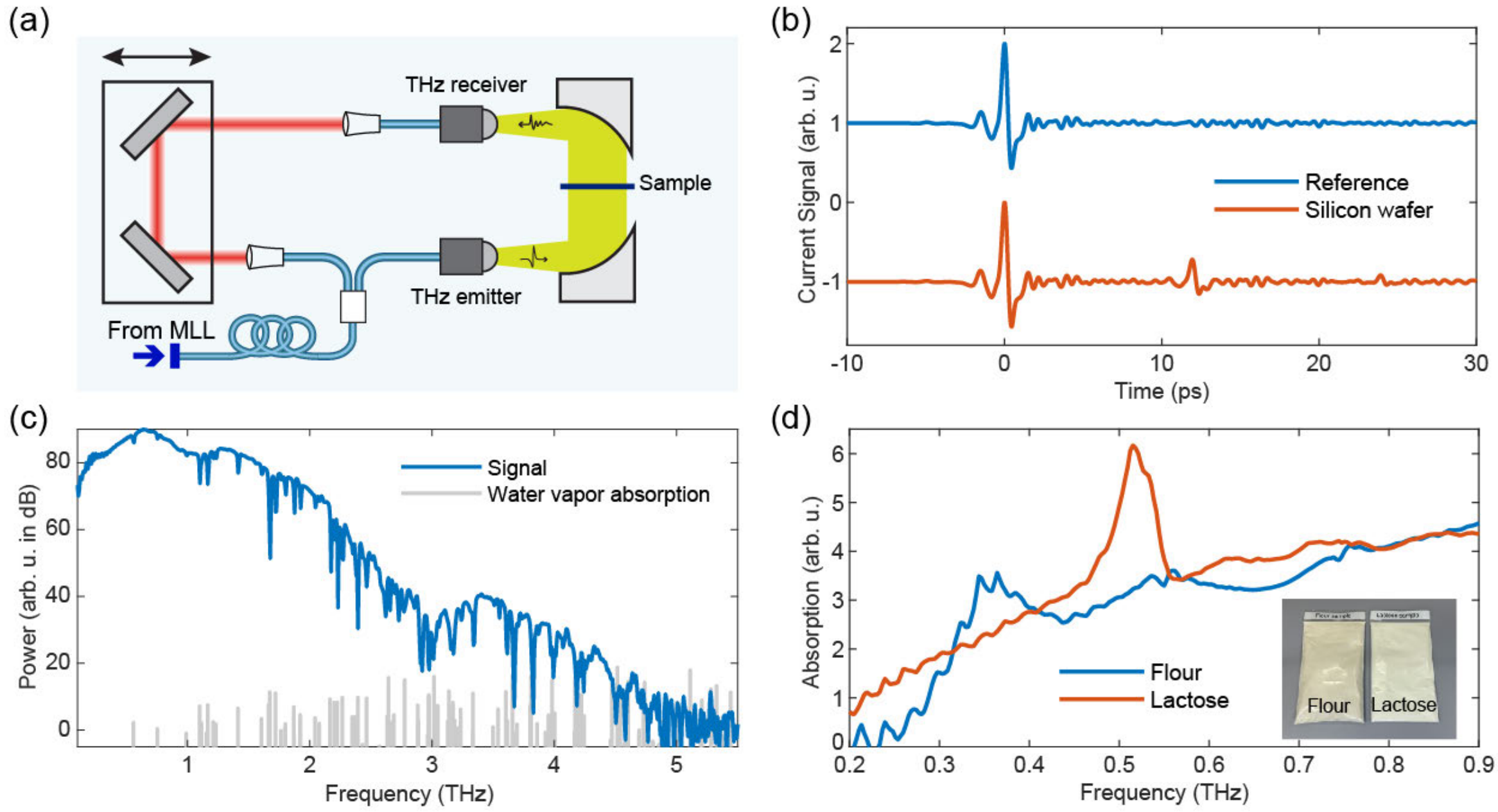

New Figure 5: Demonstration of a THz-TDS system directly driven by the integrated Mamyshev oscillator

In our demonstration, the integrated Mamyshev oscillator provides sub-150 fs pulses and >100 mW average power, directly driving a THz-TDS system without any pulse amplification. This enables a peak dynamic range of 90 dB and a 5 THz detection bandwidth (Fig. 5), with a 5-minute averaging time. Atmospheric water-vapor absorption lines are clearly resolved in the spectrum. **These results are not merely a proof-of-concept: they already rival the performance of commercial terahertz sensors or spectrometers on the market** [R1.25-1.29], and they substantially exceed prior THz-TDS demonstrations driven by integrated ultrafast sources, which report markedly lower peak dynamic range and bandwidth (microcomb: 40 dB, 0.6 THz [R1.30]; semiconductor MLL: 45 dB, 0.6 THz [R1.31]; 90 dB, 1.6 THz (*6.5-hour averaging*) [R1.32]). Figure R1 summarizes this comparison and added Supplementary Information S14 provides full references.

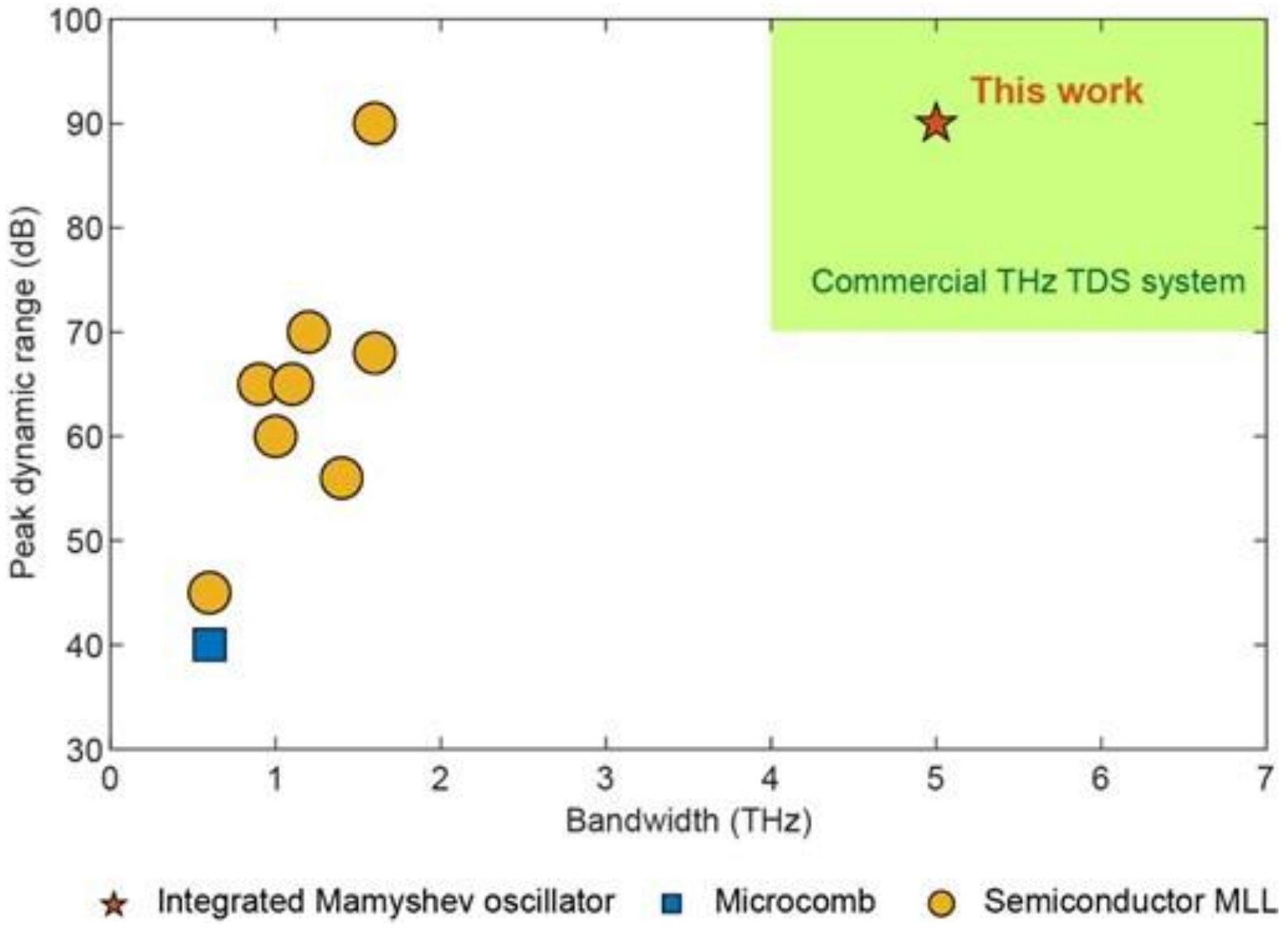

Figure R1 Graphical comparison of terahertz time-domain spectroscopy (THz-TDS) demonstrations. Included in Supplementary Information S14 with full references.

We further performed two representative measurements enabled by this THz-TDS system. First, thickness gauging of a silicon wafer: from the time-of-flight trace we measured an 11.93 ps pulse separation, corresponding to a thickness of 523.9 micrometers, in close agreement with the nominal 525 micrometers. Second, material identification: we distinguish lactose powder ($C_{12}H_{22}O_{11}$) from wheat flour by resolving the characteristic absorption feature near 0.53 THz [R1.33].

Together, these new results show that our wafer-scale-manufactured laser oscillator removed the ultra-fast laser bottleneck in THz instrumentation, and provides a realistic path toward handheld and lower-cost time-domain THz sensors, particularly when combined with established approaches such as asynchronous optical sampling (*moving-parts-free*) and, in future work, further integration of THz emitters (bonded semiconductor or ferroelectrics). We believe this can have significant impacts, **from academic research in material science, chemical dynamics, and semiconductor physics, to industrial employments for nondestructive inspection.**

We would like to emphasize that THz-TDS is only one concrete example. Numerous applications, within or beyond photonics, are waiting to be explored by our photonic integrated Mamyshev oscillator. For example, the combination of high pulse energy, ultrashort pulses, and low-noise operation directly enables:

- **Integrated optical spectroscopy.** Recent advances in high-resolution on-chip spectrometers offer compact, low-cost tools for chemical, environmental, and material sensing. However, most of them works *only with single spatial mode and suffer from very low optical throughput/etendue* (Ref. [R1.34]). The octave-spanning supercontinuum generated by our laser provides the high-brightness broadband illumination necessary to make these spectrometers viable for portable or field-deployable infrared spectroscopy applications such as plastics identification or food adulteration detection.
- **Biomedical imaging and diagnostics.** The demonstrated nanojoule pulse energy is already sufficient for second-harmonic generation microscopy. In addition, the generated broadband supercontinuum is well suited for high-resolution spectral-domain optical coherence tomography (OCT), widely used in ophthalmic diagnostics (Ref. [R1.35]). An integrated femtosecond source has the potential to enable point-of-care imaging and portable clinical instruments.
- **Precision ranging and industrial metrology**. Ultrafast pulses can enable time-of-flight ranging with significantly higher dynamic range than nanosecond pulses (Ref. [R1.36]). A compact, low-cost femtosecond source can open new opportunities for precision length metrology in industrial environments.

We also note that the demonstrated pulse energy makes the laser an ideal seed source for integrated optical amplifiers, which typically provide a limited gain up to approximately 30 dB, if an even higher pulse energy is required.

Finally, we respectfully submit that the central contribution of our work is the removal of a long-standing barrier to chip-scale ultrafast science. Pulse energy and the (compressed) pulse duration is the key practical bottleneck that has prevented integrated ultrafast sources from being deployed in cross-disciplinary applications. By achieving nanojoule-level energies and sub-150 fs pulses, *our result is less about a single application and more about unlocking an entire class of miniaturized systems* that previously required bulky Ti:sapphire or fiber lasers. In this sense, the result is less about a single application and more about introducing a fundamentally new capability that can be propagated across multiple scientific and technological domains, which we believe is aligned with the criteria for a broad *Nature* audience.

Action taken: In response to your comments, we have added a THz-TDS demonstration to explicitly showcase relevance to sensing, chemistry, and materials science (Fig. 5 in main text, and corresponding experimental details in the Supplementary Information S3.H). We have revised the introduction and the

abstract as well to emphasize the broad, cross-disciplinary impact of the work beyond photonics. The performance comparison to state-of-the-art commercial solutions and ones using integrated sources, with full references are included in Supplementary Information S14.

References:

[R1.1] Zewail, A. H. Femtochemistry: Atomic-scale dynamics of the chemical bond. The Journal of Physical Chemistry A 104, 5660–5694 (2000).

[R1.2] Lee, J., Kim, Y.-J., Lee, K., Lee, S. & Kim, S.-W. Time-of-flight measurement with femtosecond light pulses. Nature Photon 4, 716–720 (2010).

[R1.3] Li, X., Li, J., Li, Y., Ozcan, A. & Jarrahi, M. High-throughput terahertz imaging: progress and challenges. Light Sci Appl 12, 233 (2023).

[R1.4] Ghelfi, P. et al. A fully photonics-based coherent radar system. Nature 507, 341–345 (2014).

[R1.5] Soong, H. K. & Malta, J. B. Femtosecond Lasers in Ophthalmology. American Journal of Ophthalmology 147, 189–197.e2 (2009).

[R1.6] Kannatey-Asibu, E. Principles of Laser Materials Processing: Developments and Applications (John Wiley & Sons, Inc., Hoboken, NJ, 2023).

[R1.7] Helmchen, F. & Denk, W. Deep tissue two-photon microscopy. Nat Methods 2, 932–940 (2005).

[R1.8] Beloy, K. et al. Frequency ratio measurements at 18-digit accuracy using an optical clock network. Nature 591, 564–569 (2021).

[R1.9] Diddams, S. A., Hollberg, L. & Mbele, V. Molecular fingerprinting with the resolved modes of a femtosecond laser frequency comb. Nature 445, 627–630 (2007).

[R1.10] Coddington, I., Swann, W. C., Nenadovic, L. & Newbury, N. R. Rapid and precise absolute distance measurements at long range. Nature Photon 3, 351–356 (2009).

[R1.11] Picque, N. & Hansch, T. W. Frequency comb spectroscopy. Nature Photonics 13, 146–157 (2019).

[R1.12] Xin, M. et al. Attosecond precision multi-kilometer laser-microwave network. Light: Science & Applications 6, e16187–e16187 (2017).

[R1.13] Koch, M., Mittleman, D. M., Ornik, J. & Castro-Camus, E. Terahertz time-domain spectroscopy. Nat Rev Methods Primers 3, 48 (2023).

[R1.14]: Baxter, Jason B., and Glenn W. Guglietta. "Terahertz spectroscopy." Analytical chemistry 83.12 (2011): 4342-4368. https://doi.org/10.1021/ac200907z

[R1.15]: McIntosh, Alexander I., et al. "Terahertz spectroscopy: a powerful new tool for the chemical sciences?." Chemical Society Reviews 41.6 (2012): 2072-2082. https://doi.org/10.1039/C1CS15277G

[R1.16]: Nsengiyumva, Walter, et al. "Sensing and nondestructive testing applications of terahertz spectroscopy and imaging systems: State-of-the-art and state-of-the-practice." IEEE Transactions on Instrumentation and Measurement 72 (2023): 1-83.

[R1.17] Li, X. et al. Plasmonic photoconductive terahertz focal-plane array with pixel super-resolution. Nat. Photon. 18, 139–148 (2024).

[R1.18]: Liu, H.-B., et al. Terahertz Spectroscopy and Imaging for Defense and Security Applications. Proc. IEEE 95, 1514–1527 (2007). https://doi.org/10.1109/JPROC.2007.898903

[R1.19]: Cai, J. et al. Dental caries diagnosis using terahertz spectroscopy and birefringence. Opt. Express, OE 30, 13134–13147 (2022).

[R1.20]: Zeitler, J. A. et al. Terahertz pulsed spectroscopy and imaging in the pharmaceutical setting - a review. Journal of Pharmacy and Pharmacology 59, 209–223 (2010).

[R1.21]: Afsah-Hejri, et.al. A Comprehensive Review on Food Applications of Terahertz Spectroscopy and Imaging. Comprehensive Reviews in Food Science and Food Safety 18, 1563–1621 (2019). https://doi.org/10.1111%2F1541-4337.12490

[R1.22]: Guillet, J.-P. et al. Art Painting Diagnostic Before Restoration with Terahertz and Millimeter Waves. J Infrared Milli Terahz Waves 38, 369–379 (2017).

[R1.23]: E. Kato, et.al. "Development and Application of Terahertz Optical Sampling Systems for the Semiconductor Industry," 2020 IEEE International Symposium on Radio-Frequency Integration Technology (RFIT), Hiroshima, Japan, 2020, pp. 1-3, https://doi.org/10.1109/RFIT49453.2020.9226197

[R1.24] Hochrein, Thomas. "Markets, availability, notice, and technical performance of terahertz systems: Historic development, present, and trends." Journal of Infrared, Millimeter, and Terahertz Waves 36.3 (2015): 235-254.

[R1.25]: TeraPulse Lx System with Lx PolyScan Head: https://teraview.com/terapulselx/

[R1.26]: Hamamatsu C12068-01: https://www.hamamatsu.com/eu/en/product/photometry-systems/terahertz-spectrometer/C12068-01.html

[R1.27]: LUNA HTS40n2 Transmitter Type 2 with the T-Ray® 5000: https://lunainc.com/product/hts40n2-transmitter-type-2

[R1.28] Menlo Systems Tera K15: https://www.menlosystems.com/products/thz-time-domain-solutions/tera-k15/

[R1.29] Vieweg, N., et al. Terahertz-time domain spectrometer with 90 dB peak dynamic range. J Infrared Milli Terahz Waves 35, 823–832 (2014). https://doi.org/10.1007/s10762-014-0085-9

[R1.30] Peters, L. et al. Millimetre-Wave Comb Generated by an Optical Microcomb. Preprint at https://doi.org/10.48550/arXiv.2512.05005 (2025).

[R1.31]: Merghem, K., Busch, S.F., Lelarge, F. et al. Terahertz Time-Domain Spectroscopy System Driven by a Monolithic Semiconductor Laser. J Infrared Milli Terahz Waves 38, 958–962 (2017). https://doi.org/10.1007/s10762-017-0401-2

[R1.32] Cherniak, V., Kubiczek, T., Kolpatzeck, K. et al. Laser diode based THz-TDS system with 133 dB peak signal-to-noise ratio at 100 GHz. Sci Rep 13, 13476 (2023). https://doi.org/10.1038/s41598-023-40634-3 (Performance data taken from the comparison in Figure 1, claim for 133 dB peak SNR is questionable when considering the noise floor in Figure 8).

[R1.33] Datta, S. et al. Terahertz Spectroscopic Analysis of Lactose in Infant Formula: Implications for Detection and Quantification. Molecules 27, 5040 (2022).

[R1.34]: Peters, Maarten RA, et al. "Integrated photonic spectrometers: a critical review." Photonics Insights 4.4 (2025): R10-R10.

[R1.35]: Barrick, Jessica, et al. "High-speed and high-sensitivity parallel spectral-domain optical coherence tomography using a supercontinuum light source." Optics letters 41.24 (2016): 5620-5623.

[R1.36]: Lee, J., Kim, YJ., Lee, K. et al. Time-of-flight measurement with femtosecond light pulses. Nature Photon 4, 716–720 (2010). https://doi.org/10.1038/nphoton.2010.175

Referee #2 (Remarks to the Author):

"This manuscript presents the development of an integrated Mamyshev oscillator on a photonic chip, aiming to translate the powerful pulse-energy scaling capabilities of this cavity design from fiber platforms to a compact, on-chip format. The work addresses a significant challenge in integrated ultrafast photonics by attempting to implement the Mamyshev mechanism's nonlinear switching (acting as a saturable absorber equivalent) within a monolithic waveguide structure. This approach to achieving high-energy ultrashort pulses on-chip is highly relevant and timely, which can be published by high-profile technical journals. However, I do not recommend this manuscript to be published in Nature for the following concerns:"

Answer: We thank the reviewer for their careful assessment of our manuscript and for recognizing that our work timely addresses a significant challenge in integrated ultrafast photonics. We appreciate the opportunity to clarify several aspects of the architecture, experimental implementation, and future integration pathway.

Referee #2: "1. It's claimed to have "demonstrated a fully integrated Mamyshev oscillator architecture MLL based on a wafer-scale fabricated erbium-doped Si3N4 photonic chip", while two high-power, SMF-coupled 1480-nm pump lasers were still required in the experiment."

Answer: We thank the reviewer for raising this point. We agree that our original phrasing could be read as implying monolithic integration of the pump sources. In the revised manuscript, we therefore rewrote the claim to avoid any ambiguity: we have removed all references to "fully integrated" and clarified that we demonstrate the wafer-fabricated integrated Mamyshev oscillator engine, while the optical pump power is provided externally in this prototype.

We emphasize that we strongly believe that using off-chip pump lasers in the current experiment does not diminish the scientific and practical significance of the result. *The remarks from Referee #4 appear consistent with our view, which we quote: "I don't think lasers need to be integrated, they can be off-chip".* Stating that off-chip pump sources are acceptable provided the integrated oscillator operates autonomously (which we address in the answer to Question 2).

We have the following four reasons:

- **Consistency with standard practice in the integrated photonics community (including in recent *Nature* publications).**

We would like to clarify that, in integrated photonics, it is routine to use external pump or drive lasers to validate a new on-chip photonic functionality at the *component-prototyping stage.*

Numerous (>8) works published in *Nature* in the last three years that explicitly claim "integrated" or "on-chip" in the title still rely on external lasers for the demonstration experiment. In many cases, the laser used are even fiber-amplified or external cavity lasers, with less obvious path for future integration than direct diode lasers.

- Zhao, Y., et al. All-optical frequency division **on-chip** using a single laser. *Nature* 627, 546–552 (2024). https://doi.org/10.1038/s41586-024-07136-2 :
  EDFA amplified lasers are used to pump the ring resonator for on-chip comb generation and OPO. (Fig.4(d) is only proposed but not yet realized in the publication).
- Sun, S., et al. **Integrated** optical frequency division for microwave and mmWave generation. *Nature* 627, 540–545 (2024). https://doi.org/10.1038/s41586-024-07057-0 :
  EDFA amplified table-top external cavity diode lasers (ECDLs) (NewFocus Velocity) are used for pumping the microcomb.

- Feng, H., et al. **Integrated** lithium niobate microwave photonic processing engine. *Nature* 627, 80–87 (2024). https://doi.org/10.1038/s41586-024-07078-9 :
  EDFA amplified table-top tunable lasers (Santec TSL-510) are used for the signal processing experiments.
- Zhao, P., et al. Ultra-broadband optical amplification using nonlinear **integrated** waveguides. Nature 640, 918–923 (2025). https://doi.org/10.1038/s41586-025-08824-3 :
  An external, high power (2.5 W on-chip), single frequency laser (unspecified, but likely only possible with EDFAs) is used for pumping the integrated amplifier.
- Kuznetsov, N., et al. An ultra-broadband **photonic-chip-based** parametric amplifier. *Nature* 639, 928–934 (2025). https://doi.org/10.1038/s41586-025-08666-z :
  EDFA (Keopsys) amplified table-top tunable lasers (Toptica CTL) are used for pumping the integrated amplifier.
- Yang, J., et al. Titanium:sapphire-on-insulator **integrated lasers** and amplifiers. *Nature* 630, 853–859 (2024). https://doi.org/10.1038/s41586-024-07457-2 :
  Table-top frequency doubled diode pumped solid state laser (Coherent Verdi) are used for the demonstration of the best performance of the integrated Ti:Sa amplifier and laser, diode laser pumps are only shown with significant performance trade-offs.
- Stokowski, H.S., et al. **Integrated** frequency-modulated optical parametric oscillator. *Nature* 627, 95–100 (2024). https://doi.org/10.1038/s41586-024-07071-2 :
  Broadband tunable lasers (presumably table-top, after EDFA amplification in one case) are used for the integrated OPO characterization, chip integration is only proposed but not yet demonstrated in the publication.
- Li, B., et al. Frequency–angular resolving LiDAR using **chip-scale** acousto-optic beam steering. *Nature* 620, 316–322 (2023). https://doi.org/10.1038/s41586-023-06201-6 :
  External narrow linewidth lasers and modulators (Thorlabs ULN15PC, Thorlabs LNP6118) are used for generating the optical signal sent out for LiDAR ranging.

Likewise, recent works explicitly claiming *integrated lasers/OPOs* published on other leading journals frequently employ external table-top pump lasers for the proof-of-concept demonstration.

Here we sample a few:

- Singh, N., et al. **Silicon photonics-based** high-energy passively Q-switched **laser**. *Nat. Photon.* 18, 485–491 (2024). https://doi.org/10.1038/s41566-024-01388-0
  A table-top laser (TLG 220; Alnair Labs). amplified with a high-power polarization-maintaining L-band amplifier (IPG EAR-10-1610-LP-SF) were used as the 1.6 um pump.
- Wang, Y., et al. **Photonic-circuit-integrated** titanium:sapphire **laser**. *Nat. Photon.* 17, 338–345 (2023). https://doi.org/10.1038/s41566-022-01144-2
  Table-top 532 nm diode pumped solid-state lasers are used for pumping the PIC integrated Ti:Sa laser.
- Sun, Y., et al. Advancing **on-chip** Kerr **optical parametric oscillation** towards coherent applications covering the green gap. *Light Sci Appl* 13, 201 (2024). https://doi.org/10.1038/s41377-024-01534-x
  A table-top ECDL amplified with a tapered amplifier is used to pump the on-chip OPO.
- Perez, E.F., et al. High-performance Kerr microresonator **optical parametric oscillator on a silicon chip.** *Nat Commun.*14, 242 (2023). https://doi.org/10.1038/s41467-022-35746-9
  A table-top laser (Toptica 980 CTL) amplified by a tapered SOA is used to pump the OPO.
- Liu, K., et al. Large mode volume **integrated** Brillouin **lasers** for scalable ultra-low linewidth and high power. *Nat Commun* 16, 6419 (2025). https://doi.org/10.1038/s41467-025-61637-w
  A table-top tunable ECDL amplified by a EDFA is used as the pump for the Brillouin laser.

- Luis Ledezma, et al. Octave-spanning tunable infrared **parametric oscillators** in **nanophotonics**.*Sci. Adv.* 9, eadf9711 (2023). https://doi.org/10.1126/sciadv.adf9711
  A table-top ECDL, gated by a SOA and amplified with a YDFA is used as the pump of the integrated OPO.
- Sekine, R., et al. Multi-octave frequency comb from an ultra-low-threshold **nanophotonic parametric oscillator.** *Nat. Photon.* 19, 1189–1195 (2025). https://doi.org/10.1038/s41566-025-01753-7
  A table-top Yb-doped-fiber-based mode-locked laser (Menlo Orange HP10 Yb) is used as the pump for the integrated OPO.

Our use of off-chip 1480 nm diode pumps therefore follows established practice at this development stage and does not limit the feasibility of subsequent system-level integration.

We therefore respectfully submit that requiring integration of the pump diodes in the present study would shift the evaluation from the core scientific and technological advance to packaging and system-integration engineering. While important, these engineering steps are separable from the central contribution of this manuscript and are more appropriately addressed in follow-on work focused on a commercially viable product.

- **The pump powers are compatible with butterfly diodes and hybrid integration.**

In the revised manuscript and Supplementary Information, we now explicitly show that the laser can be self-seeded and then operated with two compact, *butterfly-packaged* diode pumps, and all the self-seeded experiments are performed with these butterfly-packaged laser diodes.

We emphasis that the required pump power even to reach the full nanojoule level performance can be supplied by such 1480 nm diode modules in butterfly packages as well, if polarization combining (either on-chip, co-packaged or within the laser-diode package) is utilized. In particular, we clarify that *the tabletop Connet pump laser units used in our experiment are actually itself built-upon these butterfly-packaged diodes*. *The Lumibird Raman laser used in some early measurements were selected for convenience and availability during prototyping, rather than because the integrated oscillator requires unrealistically high power or a special pump technology.*

We note that the relatively high pump power requirement does not imply poor optical conversion efficiency, but is necessary for reaching the high pulse energy. The total on-chip optical-to-optical energy conversion efficiency reaches 27.5% (comparable with other works in solid-state mode-locked lasers [R2.1]) and the total output power reaches 360 mW (summing two output ports).

Recently, there are also reports by Furukawa/FITEL for two co-packaged, highly reliable 14xx nm laser diodes in a single package, designed for polarization combining, which provide total output power of 1.3 W. This can support the starting and operation of the MLL at reduced power *with a single 14-pin butterfly packaged module*, and support full nanojoule level operation with only two modules [R2.2].

In future developments, the diodes can be co-packaged and coupled by butt coupling (with readily implemented on-chip WDMs, see reply to Question 9) or micro-optics benches (using on-chip WDMs or thin-film filter WDMs), yielding a compact, self-contained optical subsystem. Both approaches are widely used in commercial laser products and in pluggable transceiver modules. In prior work of our group, we have demonstrated hybrid integration of 1480 nm pump diodes for erbium-based integrated lasers and amplifiers (Refs. [R2.3] and [R2.4]).

In these hybrid setups, thermal management is a solvable issue, for example, in Ref. [R2.5], comb lasers driven by butt coupled high power lasers using 4.2 W drive power are shown (further discussed in reply to Question 13). Silicon photonics optical engines in telecom industry also routinely need to manage few watts of thermal dissipation.

We would also note that the pump power requirement can be further relaxed to ***lower than 140 mW off-chip power*** *by redesigning the device using less dispersive waveguides of a 2 um x 600 nm cross-section* (for example, and keep other design parameters the same; even lower threshold is possible with higher implantation energy), per numerical simulation. Although there will be obvious trade-off in pulse energy (66 pJ) due to energy conservation and pulse duration (dechirp to 180 fs FWHM), such lowered pump power shall further reduce the burden on the thermal management for a hybrid device.

We stress that hybrid integration of pumps is certainly attractive, but it is not necessary to validate the integrated Mamyshev oscillator concept and the key advancement of on-chip generation of high energy pulses, and it is not the focus of this manuscript.

- **External optical pumping is the norm for mode-locked lasers and does not contradict an "photonic integrated oscillator engine."**

External optical pumping is standard for essentially all high-performance mode-locked lasers, including solid-state and fiber platforms. Table-top Ti:sapphire, Cr:LiSAF, and Yb:KGW systems, as well as erbium/ytterbium-fiber mode-locked lasers, universally rely on external pump diodes (or frequency-doubled solid-state pump lasers). This separation between (i) the mode-locked oscillator/pulse-formation mechanism and (ii) an external electrical-to-optical power supply, is standard in the community. Our integrated Mamyshev oscillator should be understood in the same way: the cavity, nonlinear switching mechanism, and pulse evolution are integrated on-chip as a mass-manufactured core; the pump is an external module that supplies optical power.

More fundamentally, one key strength of a PIC-based ultrafast laser is not that the pump diode must sit on the same substrate, but that the ultrafast pulse engine is implemented in a scalable photonic platform that can directly integrate additional optical functions on chip.

With external pumping, a PIC oscillator still uniquely enables straightforward co-integration (monolithically or via heterogeneous integration) of other functionalities including MHz piezoelectric tuning, pulse picking and gating, interleaving, chirp compensation/adjustment, nonlinear wavelength conversion, optical sampling, and supercontinuum generation, regardless whether the pump diode sit on the same substrate.

- **Remote (fiber-delivered) pumping is not only acceptable, but is an established and increasingly preferred system architecture in the industry.**

This is particularly true in silicon photonics, which is arguably the largest commercial ecosystem for integrated photonic technology. In remote-pumped architectures, the pump (or light source) is intentionally separated from the photonic integrated circuit and delivered via standard single-mode fiber. This design choice is motivated by several concrete and well-understood advantages:

- *Thermal management and reliability*. The pump diode is typically the dominant heat source. Moving it off the photonic chip reduces local temperature rise, relaxes thermal design constraints, and improves stability of on-chip components.
- *Modularity and serviceability ("hot swapping").* Fiber-connected pump modules can be replaced without disturbing the photonic chip package. This modularity is highly valued in industrial settings, where field serviceability is critical.
- *Leveraging highly optimized pump modules.* Telecom pump diodes are mature products with optimized electrical efficiency, reliability and lifetime qualification (Telcordia), and standardized packaging. Utilizing them as external modules allows the PIC system to inherit this maturity without re-qualifying the pump at the chip-package level.

Such considerations are not hypothetical, but widely recognized by the optical communications industry, which has made sustained efforts to standardize external light sources for silicon photonics-based systems. Co-packaged optics (CPO) architectures, being pursued for next-generation interconnects *rely on external fiber-connected lasers,* as shown by the recent press releases from NVIDIA (Ref. [R2.6,

R2.7]). External-laser small form factors (ELSFP) laser modules (Ref. [R2.8, R2.9]) have also recently been standardized by OIF (Ref. [R2.10]) for remotely powering the silicon photonics engines.

[Redacted text and figure]

In our case, remote pumping is particularly attractive: it removes the major heat source from the mode-locked laser PIC while the cavity and pulse-formation mechanism are fully on wafer-scale mass-manufacturable chips. From an end-user standpoint, the cost, footprint and complexity reduction are still substantial because the bulky free-space cavity optics or long fiber cavities that needs expensive manual assembly are eliminated; what remains external are the compact pump module that is already a commodity component.

Action taken: We have also correspondingly amended the claims in the manuscript to show that the device developed is the optical engine for an integrated mode-locked laser system and removed wording that may cause misinterpretation ("fully-integrated"), while we believe still have significant transformative potential for significantly reducing the footprint and cost of a mode-locked laser system. In the revised manuscript and the supplementary information, we have emphasized that the pump power can be provided with semiconductor diode lasers in standard butterfly packages, which have small footprint and straightforward path for further integrations. the use of laboratory table-top fiber-coupled pump lasers including the Lumibird Raman laser was purely for experimental convenience rather than a limitation of the architecture.

Referee #2: "2. Furthermore, it's also required to initiate mode-locking in the integrated Mamyshev oscillator by feeding a commercial fiber MLL (Menlo ELMO High Power) as the seed source, delivering 51 fs FWHM pulses at a 100-MHz repetition rate and approximately 100 mW average power, which is quite a high-quality fiber MLL by itself, comparing with the output 1 was merely compressed down to 187 fs FWHM with the optimized group delay dispersion."

Answer: We thank you for highlighting this important limitation of the original submission. We agree that relying on an external fiber-based mode-locked laser for initialization would of course negate the entire advantage of a high pulse energy mode locked laser on chip and undermine the motivation for and impact of a chip-scale mode-locked source. In our revised manuscript, we have in fact addressed this crucial point. Below the changes are detailed:

- **Self-seeding without any external MLL**

In the revised manuscript we demonstrate that the integrated Mamyshev oscillator can be **reliably self-started without any external seed source**. Specifically, mode-locking is initiated by temporarily modulating the 1480 nm pump intensity after setting the microheater to an appropriate operating point, i.e., establishing a suitable relative offset between the two on-chip WBG passbands. In practice, this is implemented by directly applying current modulation to the pump diode(s) using generic commercially available diode-laser drivers.

Importantly, this start-up procedure requires **no external optical components**: no seed laser, no circulator, no seed shaping and picking. After mode-locking is established, the pump modulation is then

electronically disabled and the oscillator continues to operate autonomously in the steady-state mode-locked regime. Once mode-locked, the operating point remains tunable by adjusting the pump power and heater settings to target a desired pulse energy and spectrum within the stability region.

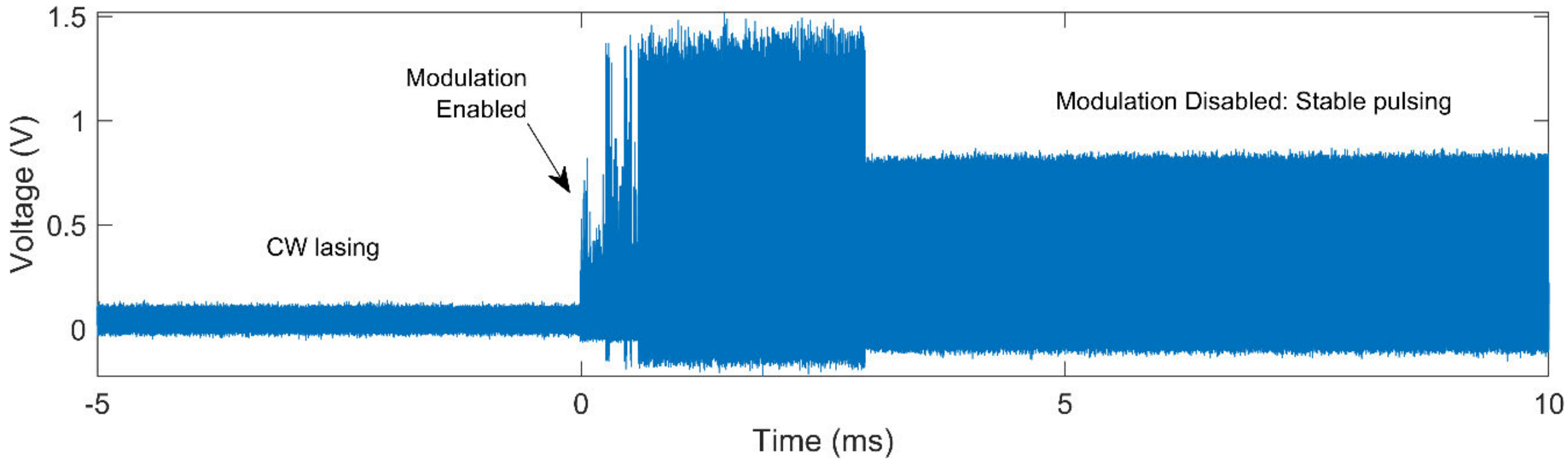

New Figure S15: Pulse emergence dynamics from the enhanced noise during modulation, and transition into stable pulsing after switching off modulation. The entire process is completed in approximately 3 ms.

In our new experiments, the pump modulation is a simple square-wave current modulation at 1.2-2.8 MHz. **The required electronics are also straightforward**, generating a square-wave modulation signal at a few MHz is **trivial with modern electronics**. For example, one can directly use the PWM output from a programmable microcontroller like RP2350 (cost <$1, [R2.11]), ESP32 (cost from $1 to $2, [R2.12]), or STM32F3 (cost $2, [R2.13]), which can also simultaneously carry out other control functionalities (shown on Figure R3). Moreover, applying such modulation to the low-voltage 1-2 A-class pump-diode currents is likewise simple in practice, either via the built-in modulation/switching capabilities of commercial diode driver modules (as used here) or, if desired, via an external switching stage (for example, a MOSFET-based shunt switch, cost <$0.3) and a constant current driver. Therefore, the required pump modulation does not represent a barrier to practical system integration and the additional Bill of Material cost is neglectable.

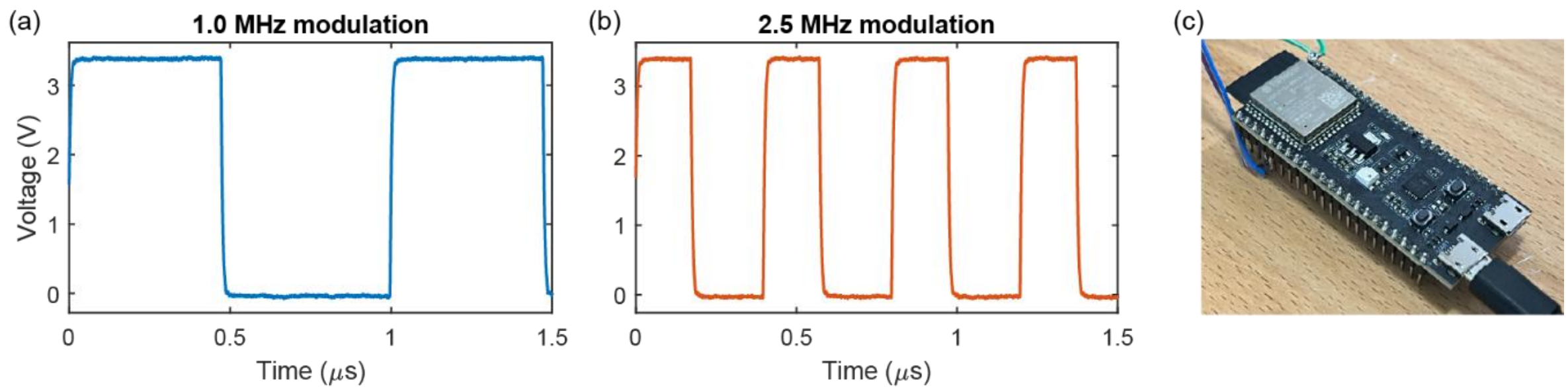

Figure R3 (a,b) Measured modulation waveform simutanously generated at (a) 1.0 MHz and (b) 2.5 MHz from a single low-cost ESP32-S3 microcontroller. (c) Photograph of the development board used for the waveform generation demonstration. The entire controller development board (ESP32-S3-DevKitC-1) cost ~$5 on AliExpress and is capable of simutanously generating up to 4 independent square wave modulation signals and carry out the starting control procedure autonomously.

The procedure to start the Mamyshev oscillator is readily automatized as following:

- Turn on the pump power.
- Set the microheaters.
- Enable pump current modulation.
- Wait for a time less than 10 s, usually sub-1 s.
- Disable pump current modulation.
  Now the laser shall be mode-locked, ready for use.
- Optional: Ramp up or ramp down the pump power and change heater setpoint to target a desired pulse energy and output spectrum.

The self-starting success rate is virtually 100% over a wide total pump power range of 623 mW to 1012 mW launched, corresponding to 467 mW to 759 mW on-chip power. Across this range, the oscillator always reaches the mode-locked state within 10 s after enabling modulation; in many cases, initiation occurs on timescales of tens of milliseconds.

We include representative experimental data in the revised manuscript and Supplementary Information, including:

(i) the transient dynamics during the self-start process (Supplementary Fig. S14 and S15),

(ii) repeatability across multiple independent start events (Fig. 2(e)),

(iii) spectra confirming that self-starting converges to the same mode-locked state as external seeding for identical cavity settings (Supplementary Fig. S17), and

(iv) statistics of the initialization time versus average pump power (Supplementary Fig. S18).

We note that we use "self-seeded" and "self-starting" interchangeably, as consistent with terminology in literature (e.g.: Ref. [R2.14, R2.15]) about fiber-based Mamyshev oscillators that uses the same pump modulation technique, as well as Ref. [R2.16] using filter tuning to initiate mode-locking. Despite this admittedly does not fit a very strict definition of "self-starting": being able to mode-lock immediately when the constant pump power is applied, we believe our approach is equivalent in practice as it does not compromise on the performance in any way or add significant complexity, everything is electronically controlled and readily automatized. We hope these additions directly address your concern by showing that the oscillator can be self-seeded without external seeding.

- **Clarification: the Menlo ELMO's performance is not required for seeding**

We also would like to clarify the role of the Menlo ELMO in the original submission. While the ELMO is indeed a high-performance 51 fs fiber MLL, its performance was *not required* for the seeding approach. We used it because of experimental convenience. As described in the revised supplementary information, we intentionally inserted a programmable spectral filter to narrow the seed spectrum and *deliberately degrade the seed pulse* to emulate a few hundred femtoseconds to picosecond-class source. The purpose was to demonstrate that the integrated oscillator does not require a high-quality ultrashort seed; rather, it only requires a transient perturbation sufficient to bring the system into the basin of attraction of the stable mode-locked state.

In practice, substantially lower-performance sources (for example, an amplified semiconductor mode-locked laser or other picosecond pulsed sources) would be sufficient for such initialization. The steady-state mode-locked output is determined primarily by the integrated cavity parameters (pump power and filter detuning), rather than the quality of the initial seed pulse (detailed in the answer to the next question).

Action Taken: We have edited the manuscript extensively to put emphasis on the new self-starting method based on temporary pump-current modulation, demonstrating mode-locking initiation without any external seed laser:

- The abstract, introduction and manuscript narrative are updated to emphasize self-seeding/self-starting capability and to remove any implication that external MLL seeding is a required for operation.
- We revised Figure 2 in the main text and the experimental setup description in the Supplementary Information S3 to highlight the repeatability and reflect the self-starting configuration (no need for seed injection path). We also commented on the generation of the modulation waveform with low-cost electronics.

- We have also clarified in the Supplementary Information S3 that in the externally seeded configuration, the Menlo ELMO was used for availability in the lab and was deliberately degraded, and that lower-performance pulsed sources are sufficient for initialization when external seeding is used.

Referee #2: "3. How sensitive would the performance of that seed source affect the output of the integrated Mamyshev oscillator?"

Answer: We thank the reviewer for raising this point, as it goes to the important question whether the measured steady-state output could be limited or "imprinted" by the properties of the source used to initiate mode-locking.

In the revised manuscript, we demonstrate a turn-key self-starting (self-seeding) procedure based on temporary pump-current modulation, which removes the requirement for an external ultrafast seed source for routine operation. In this case, the steady-state mode-locked operating point is set by the on-chip cavity parameters, in particular the pump powers and the relative detuning of the two on-chip spectral filters (WBG passbands). As a result, the output characteristics that we report correspond to the oscillator settling into a stable mode-locked state determined by these cavity settings rather than by the characteristics of the initial perturbation.

Experimentally, we support this conclusion in the revision by comparing the spectrum of the laser seeded by an external pulse and by pump modulation in Fig. S17 in the Supplementary Information S5, the resulting spectrum is similar despite of the very different pathway taken to reach the stable mode-locking state. This is further supported by the repeated independent self-start events converge reproducibly to the same mode-locked output for identical pump and heater settings, despite the stochastic nature of the self-seeded starting process (see the revised Fig. 2(e)).

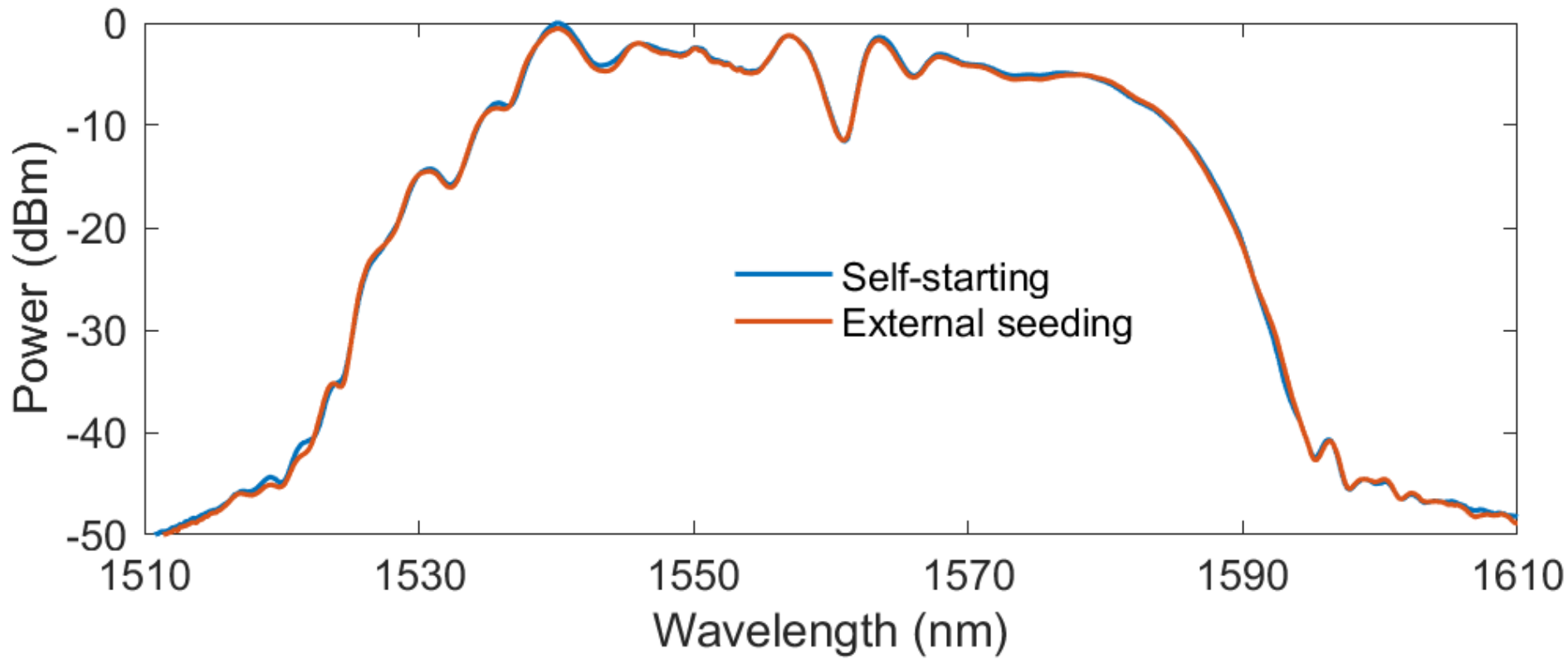

New Figure S17 Comparison between the experimentally measured optical spectrum of a self-started mode-locked state and an external seeded state, showing nearly identical optical spectum despite of very different routes and dynamics to reach the mode-locked state.

The same independence of seeding source has also been observed numerically and experimentally for the external seeding case as well. Shown in Figure R4, the simulated shapes of the intracavity pulse are practically identical regardless which of the three seed parameters are used. This is expected for the laser as once pulsing is initialized in the cavity; the subsequent evolution will quickly erase the properties of the original pulse. In this sense, the steady-state can be viewed as a stable attractor of the nonlinear dynamical system, consistent with observations in fiber-based Mamyshev oscillators (e.g., Ref. [R2.17]).

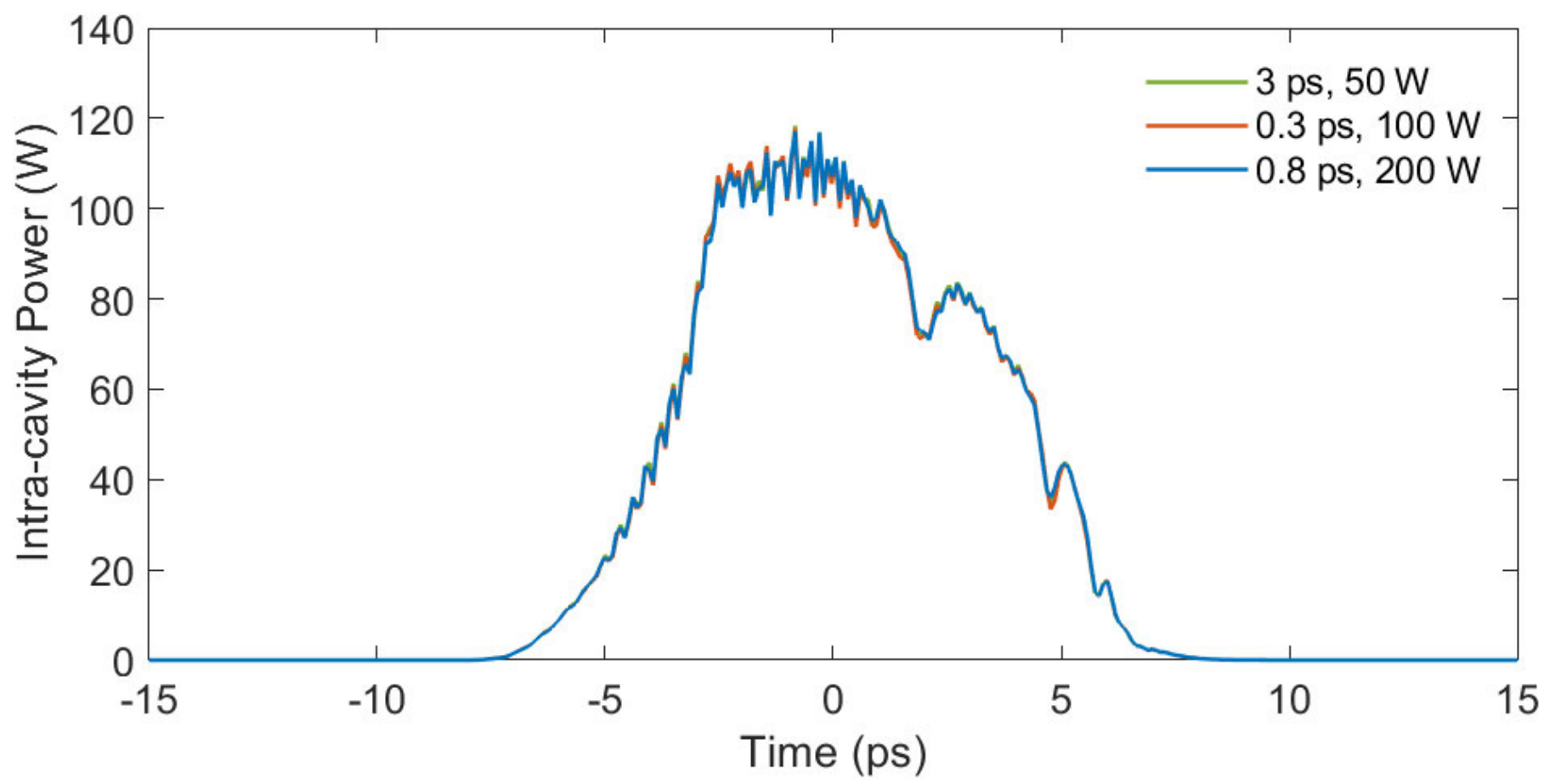

Figure R4 Comparison of simulated intra-cavity pulse after 20 round-trips, after correcting for a global time-shift, when seeded by a single sech shaped pulse of 3 ps duration, 50 W peak power; 300 fs duration, 100 W peak power; or 800 fs duration, 200 W peak power.

Action taken: In the revised manuscript and Supplementary Information, we add the pump-modulation self-starting method and include experimental evidence (new Fig. 2(e) and Fig. S17) demonstrating reproducible convergence to the same steady-state mode-locked output for fixed cavity settings, clarifying that the reported performance reflects the intrinsic steady-state of the integrated oscillator rather than the quality of an external seed source.

Referee #2: "4. Why was 1480 nm selected instead of the conventional 980 nm pump for erbium gain? While their custom EDFA consisted of two 980/1550 nm WDMs (Thorlabs), a 980 nm pump laser diode (Aerodiode), and a short (~50 cm) section of highly doped erbium fiber (LIEKKI ER80-4/125-HD-PM)."

Answer: We thank the reviewer for this question. Both 980 nm and 1480 nm pumping are widely used for erbium gain, and we agree that 980 nm pumping is often considered the "default" choice for EDFAs. Our choice of 1480 nm pumping in this prototype integrated oscillator was primarily driven by practical considerations, rather than by any fundamental limitation of the Mamyshev-oscillator concept or the doped silicon nitride gain medium.

In this work, the on-chip cavity is accessed through edge coupling via inverse tapers that serve as the mode-field converters for both the pump and the 1550 nm laser light. Using a 1480 nm pump, which is close in wavelength to the 1550 nm signal, allows a single edge-coupler / mode-field-converter design to provide high coupling efficiency at both wavelengths simultaneously. In contrast, introducing 980 nm pump light through the same couplers would require either a separate set of couplers optimized for 980 nm (with different mode sizes and matching different UHNA or lensed fibers) and optionally wavelength multiplexers. For a proof-of-concept demonstration focused on validating the integrated Mamyshev oscillator architecture, 1480 nm pumping offered a lower-risk and more direct path to high on-chip pump power with minimal additional photonic components.

We emphasize that our device principles are fully compatible with 980 nm pumping. In future iterations, 980 nm pump delivery can be implemented using integrated wavelength-division multiplexers (e.g., directional couplers or Mach-Zehnder interferometer-based WDMs) and dedicated facet couplers optimized for 980 nm. Both types of building blocks are readily available on chip. From a gain-physics standpoint, 980 nm pumping can provide higher inversion and higher small-signal gain, which may enable shorter gain sections and shorter cavities (thus higher repetition rates), and potentially improved noise performance in certain regimes. At the same time, the higher quantum defect at 980 nm implies

increased heat load in the amplifier cavity while the laser diodes are more power efficient, which may requires more engineering focus on the thermal management of on-chip cavity.

We also note that 1480 nm pumping is not unusual in applications: it is widely deployed in the telecommunications industry for both EDFAs and fiber Raman amplification, and mature high-power single-mode 1480 nm InP pump diode modules are commodity items available from multiple vendors (e.g. Lumentum, Anritsu, Fitel, QPhotonics). Therefore, the use of 1480 nm pumping in our prototype does not represent an exotic requirement.

Finally, we agree with the reviewer's observation that our custom fiber EDFA used 980 nm pumping. That choice was made to maximize gain per unit fiber length so as to keep the fiber as short as possible, thereby reducing dispersion and nonlinear pulse distortion. This optimization criterion is specific to the fiber amplifier and does not directly translate to the on-chip oscillator cavity.

Action taken: In the revised manuscript and Supplementary Information S3.A, we clarify that 1480 nm pumping was chosen for coupling/interface simplicity in this prototype, and we add a short discussion of using 980 nm pumping is also possible for future designs and what performance trade-offs it may offer.

Referee #2: "5. In Fig.1(c), how ddid the intra-cavity pulse energy evolve?"

Answer: We thank the reviewer for this request. We agree that explicitly showing the intra-cavity energy evolution helps clarify how the pulse propagate in the cavity.

In the revised Supplementary Information (Section S2), we now include the simulated evolution of the intra-cavity pulse energy along the cavity as a function of propagation distance. The results show that within a single pass through the gain section, the energy growth is not uniform: it increases more rapidly near the ends of the gain waveguide where the local pump intensity is higher and the erbium population inversion is correspondingly larger, leading to a higher local gain coefficient. While in the middle of the gain waveguide, slight decrease of the intracavity pulse energy is observed. This is due to the depletion of the pump power at both ends of the waveguide, where the pump power is efficiently converted into the circulating pulses. We believe that in the future developments, the doping concentration may be lowered to remove the middle drop in the intracavity pulse energy.

Action taken: We added a dedicated plot (Figure. S8) and accompanying explanation in Supplementary Information Section S2 showing the intra-cavity pulse-energy evolution versus propagation distance, and we explain the observed nonuniform energy growth in terms of the longitudinal pump distribution and inversion profile.

Referee #2: "6. What's the dispersion coefficient of the integrated erbium waveguide? What's the total dispersion of the cavity? Can dispersion be precisely engineered during fabrication? Is intra-cavity dispersion management feasible to generate sub-100 fs pulses directly without external compression?"

We thank the reviewer for these questions and we would like to answer them individually.

- **Dispersion parameters in the present device.**

In this design we use silicon nitride waveguides with a 350 nm x 1600 nm cross section, which feature normal group-velocity dispersion (GVD) of $\beta_2 = 715\mathrm{ps}^2/\mathrm{km}$ and third-order dispersion $\beta_3 = -1.81\ \mathrm{ps}^3/\mathrm{km}$ at 1550 nm. For the present cavity layout, the resulting total round-trip group-delay dispersion is $0.601\ \mathrm{ps}^2$. We have added these values explicitly in the revised Supplementary Information and clarify how the total dispersion is obtained from the waveguide lengths in the cavity.

- **Dispersion engineering and fabrication control.**

We believe the dispersion can be engineered with high precision in silicon nitride platforms because it is primarily defined by geometric parameters (core thickness and width). We fabricated the devices at EPFL in a shared academic cleanroom, where the Si_3N_4 layer thickness is controlled by optical interferometry, while the waveguide width is defined lithographically and verified by SEM. In practice, we typically achieve better than ±5 nm control of thickness and ±20 nm control of width. These tolerances correspond to an uncertainty limit of GVD of approximately 6% by simulation. In future developments, fabrication in commercial foundries with statistical process control of deposition, lithography, and etching would be expected to further improve dispersion reproducibility across wafers.

- **Feasibility and trade-offs of intracavity dispersion management for direct sub-100 fs output.**

We believe that implementing lower-dispersion Mamyshev oscillators on-chip is technically plausible using thicker Si_3N_4 waveguides with near-zero or tailored dispersion, potentially combined with intracavity dispersive elements such as chirped Bragg gratings.

Direct few-cycle pulse generation from a Mamyshev oscillator is reported in Ref. [R2.18], where a cavity incorporating a grating pair compressor (can be replaced with chirped WBGs) is utilized. Both the doping of close to zero dispersion waveguides (Ref. [7] in main text) and fabrication of WBGs with them (unpublished) have been demonstrated in our group. However, achieving this on-chip would require a dedicated optimization of the cavity design (ion overlap and gain, filtering, dispersion, and nonlinearity). It can be an important topic for a follow-up study but is not the central focus of the present proof-of-concept demonstration.

At the same time, in this work we chose a strongly net normal-dispersion cavity that produces chirped intracavity and output pulses, both for the consideration of chirping the intracavity pulse to reduce peak power and avoid potential pulse break, and also for a higher overlap factor between optical mode and erbium doping and thus higher modal gain. In this sense, optimizing for maximal pulse energy and optimizing for minimum intracavity pulse duration are not necessarily the in-line and some trade-offs may need to be considered.

Finally, we would like to highlight that chirped output pulses can be desirable in practice because due to the lower peak power right after the cavity, further amplification, time delay or spectral processing on-chip can be simplified without causing pulse breaking or waveguide damages. These linearly chirped output pulses can be straightforwardly dechirped back either externally with fiber or on-chip with dispersion engineered waveguides, chirped waveguide Bragg gratings or Echelle grating pairs. Commercial fiber-based lasers (such as Menlo ELMO, Toptica FemtoFErb 1560 FD6.5 and optilab FML-15-B) are also commonly designed to reach the specified minimal pulse width after a certain fiber pigtail length, which provides additional flexibility for system integration that uses the output remotely.

Action taken: In the revised Supplementary Information S2, we explicitly report the dispersion coefficients in the waveguides and the total round-trip dispersion, and we add discussion clarifying dispersion engineering tolerances and the design trade-offs between energy scaling and direct generation of shorter pulses.

Referee #2: "7. What's the reflected ratio of the two WBGs in the experiment? How's the tunability of them by using the microheater? Is it possible to achieve self-starting by tuning the WBGs?"

Answer: We thank the reviewer for these questions and we would like to answer them individually.

- **WBG reflected ratio in the experiment.**

The peak reflectivity of the waveguide Bragg gratings (WBGs) used as the on-chip spectral filters is simulated to be close to unity, and we characterize them to exhibit >80% peak reflection at the center of the stopband (limited by calibration precision). In the cavity model, the effective fraction of

circulating power that is reflected by each grating and participates in the linear-cavity feedback is set by the overlap between the spectrum of the circulating pulse and the grating reflection band(s). In our simulations using the measured grating spectra and cavity settings detailed in the Supplementary Information S2, we find that the effective reflected ratio to be 8.3% and 9.6% of the circulating light per reflection. The exact value would depend on the pump power and on the relative wavelength offset between the two grating passbands, both affecting the circulating pulse spectrum.

- **Thermal tunability using the microheaters.**

We have added an explicit independent characterization of heater tuning in the revised Supplementary Information (Fig. S13). Heating produces a clear red shift of the reflection band. Using the band-center definition based on the two 50% reflectivity points (averaged), we measure a tuning efficiency of approximately 2.66 pm/mW, corresponding to about 0.332 GHz/mW. We have also operated the heaters continuously at ~500 mW for several hours without observing degradation, which we consider a reasonable operating level. At this power, the demonstrated tuning range is ~1.33 nm. This tuning efficiency and range can be extended in the future, if necessary, by etching deep slots into the silicon substrate for heat insulation, which can be performed together with our current die separation process with no additional cost.

- **Can self-starting be achieved by tuning the WBGs?**

In our experiments, we do not observe reliable self-starting by heater tuning alone. We attribute this to two practical considerations. First, with the present chip facets, residual back-reflections always lead to parasitic CW/Q-switched lasing, which can clamp the gain and suppress the growth of the transient pulse dynamics needed to reach the mode-locked state. Second, the microheater response is intrinsically too slow compared to the gain dynamics of the parasitic cavity: the heater thermal response rolls off on the order of ~10 kHz, whereas the relaxation oscillation occurs at MHz-rate. The successful pump-current modulation method instead can reach a significantly higher effective modulation frequency.

Action taken: In the revised Supplementary Information S4, we have explicitly report the characterization of the WBG thermal tuning coefficient and tuning range. In the revised Supplementary Information S2, we reported the ratio of the reflected power over total power incident on the WBGs.

Referee #2: “8. Based on the simulation results in Fig. S8, ~18 nm separation yields higher compressed peak power. Why were 42 cm cavity length and 9.8 nm WBG separation chosen experimentally? In addition, how about the influence of different parameters on the output pulse energy? How do other parameters (such as filter bandwidth and output ratio) impact the performance? In these terms, does the integrated Mamyshev oscillator follow the same theoretical principles as fiber-based systems?”

Answer: We thank the reviewer for these questions, which we answer individually.

- **Why 42 cm cavity length and 9.8 nm WBG separation were chosen.**

As we have fabricated the devices in a shared academic cleanroom facility without a process design kit, we reused the calibrated grating designs in earlier tapeout iterations to reduce cost and risk in this proof-of-concept demonstration. Particularly, we were not sure about whether there can be impact of grating center wavelength from pattern density (caused by lithography and chemical RIE process), while it turned out to be neglectable retrospectively. A larger WBG separation may necessities a higher nonlinear phase shift, and thus higher threshold power. At the design stage, we were also not confident in our estimation of the nonlinear coefficient of the silicon nitride waveguide after ion implantation, caused by initial lack of confidence in the effective area computation and the uncertainty of the material

nonlinear index after implantation. We chose a smaller WBG separation also to be conservative for this. Similarly, the 42 cm cavity length was selected considering several trade-offs. In practice, longer cavities reduce the required small-signal gain per round trip but make the device more susceptible to "killer" defects caused in the fabrication. We have silicon-proven designs of waveguides of 36-45 cm fabricated in earlier tapeouts, and that is why we preferred such a length instead of longer ones.

We agree with the reviewer that our simulations indicate that other filter separations (e.g., around 18 nm for the specific simulated parameter set) can yield higher dechirped peak power, and we view this as a clear indication that the present design is not yet globally optimized. In the present work, we selected parameters that are conservative and sit in the middle of the stability region, with relatively low pump threshold, instead of pursuing the ultimate performance. We believe a further optimized design can be investigated in future works.

- **Influence of key parameters (filter separation, bandwidth, output coupling) on pulse energy and peak power.**

Our simulations and experiments support the qualitative trends similar to fiber-based Mamyshev oscillators: the filter separation (effective detuning between the two spectral filters) sets the threshold power of the effective "saturable absorber" and hence strongly affects the stability region, pulse energy, and achievable spectral broadening. Increasing the separation effectively increases the threshold power which can potentially support a higher peak power after dechirping, but it also raises the threshold for pulse formation and can reduce robustness in the presence of loss and parasitic CW background. In an integrated cavity with high gain and significant facet back-reflection, the practical optimum may therefore differ from the optimum in an idealized simulation, because larger separations demand higher intracavity peak powers and stricter suppression of parasitic lasing.

The filter bandwidth also introduces an important trade-off. In the present design, the WBG passbands were chosen to be as wide as feasible with our non-chirped grating implementation, because overly narrow passbands tend to reduce the effective finesse of the cavity and increase the lasing threshold. In our simulations, we empirically observe that a larger effective filter bandwidth can also improve tolerance to residual facet back-reflections, because it reduces the tendency for narrowband parasitic lasing to clamp the gain at a wavelength that competes with the intended mode-locked state. At the same time, narrower filtering can, in some parameter regimes, improve the linearity of the output chirp and thereby improve compressibility, but at the cost of reducing the effective cavity transmission (increasing round-trip loss) and thus lowering the attainable intracavity pulse energy.

Similarly, the effective output coupling ratio (or more generally the extraction and cavity loss distribution) affects pulse energy through the standard energy-balance condition: higher extraction can increase usable output power but lowers intracavity energy and may reduce nonlinear broadening, while lower extraction increases intracavity energy but reduces the extracted power and can make the operating point more sensitive to parasitic processes.

- **Does the integrated Mamyshev oscillator follow the same principles as fiber-based systems?**

We believe that this integrated Mamyshev oscillator should follow the same theoretical principles as the fiber counterparts, as the underlaying physics for the rare-earth doped gain and the nonlinear pulse propagation in Kerr nonlinear medium is always the same.

However, an integrated Mamyshev oscillator operates in a **substantially different parameter regime**. Integrated waveguides can have more flexibility in dispersion engineering than fibers. Waveguides can be designed to have normal or anomalous GVD by changing the cross-section geometry, and can reach a much larger absolute value (>40x higher) than possible in fibers. Waveguides also have orders-of-magnitude higher nonlinearity coefficient due to tighter confinement (1000x higher than SMF28), and higher loss due to sidewall scattering. In addition, the small signal gain per unit length in rare-earth-

doped waveguides can be much higher (>110 dB/m compared to few dB/m), while the absolute cavity length is usually much shorter. These differences, together with the intrinsic complexity of the nonlinear dynamics, suggest that although the qualitative understanding of the behavior may partially carry over from fiber, the sensitivity to parameters may differ significantly for the practical design of an integrated MLL.

Action Taken: In the revised Supplementary Information S2.C, we have described that our current design is conservative and shown that we expect to achieve higher dechirped output power with an optimized design.

Referee #2: "9. Can WDM CIR ISO also be integrated? What are the possibilities and challenges of achieving full integration of the entire setup in the future?"

Answer: We thank the reviewer for this important question. We agree that system-level integration matters. We would like to emphasize that among WDM, CIR and ISO, only WDM is really necessary for the laser operation, and can be readily integrated on-chip.

- **WDM: monolithic integration is proven on silicon nitride.**

Wavelength-division multiplexing for pump/signal combination is a standard function in integrated photonics and can be implemented on silicon nitride using well-established building blocks such as directional couplers and unbalanced Mach-Zehnder interferometers. These approaches have been extensively demonstrated with low loss and good thermal stability in the literature (Ref. [R2.19], [R2.20]), and also been demonstrated by us and others for use with erbium-doped waveguide amplifiers (Ref. [R2.3], [R2.4], [R2.21], [R2.22], Fig. R5).

[Redacted text and figure]

In the present work, we did not include on-chip WDMs because the goal was to validate the integrated Mamyshev-oscillator architecture and its pulse-energy scaling in a first wafer-scale fabricated prototype, rather than to deliver a fully packaged commercial module. Adding WDMs is therefore better viewed as a packaging/system-integration step that is enabled by existing PIC technology rather than a fundamental requirement or limitation of the laser architecture. We would like to also note, as described in the reply to the Question 1, it is common for the works in the integrated photonics community to utilize external components for a demonstrative experiment, at the current device proof-of-concept stage.

- **Circulator: no longer used in the revised "self-seeded" operation mode.**

In the original submission, the circulator was used purely to enable external seed injection and convenient separation of input/output paths in the laboratory. In the revised manuscript, we demonstrate turn-key initiation by temporary pump-current modulation, which eliminates the seed injection path entirely. As a result, a circulator is not required for start-up or steady-state operation in the revised operating mode.

In the future with integrated WDMs, the output can be collected directly from the chip facets/couplers as in any integrated laser system, and standard on-chip routing (including couplers/splitters and WDMs) can be used to direct the pump and signal as desired.

- **Isolator: not required; used to protect against back-reflections from diagnostics; integration solutions exist.**

First, we would like to clarify that the isolator in our laboratory setup is not required for the oscillator to operate properly. Its role is to prevent accidental back-reflections from downstream *diagnostic instruments* from perturbing the cavity, particularly the autocorrelator and unoptimized Thorlabs photodetectors (optimized ones from Finisar/II-VI have sufficient return loss). When the return loss is appropriately managed in the packaging and application (e.g., angled facets/edge couplers, AR-coated micro-optics), an isolator is often unnecessary for stable operation. We have explicitly verified this in experiment after self-seeding the laser by pump modulation and use the output for the terahertz spectroscopy demonstration without any ISO/CIR in the optical path, and obtained comparable performance in that downstream experiment (5 THz usable bandwidth and 90 dB peak dynamic range), to the same experiment performed with ISO/CIR and external seeding.

In case that isolation is still desired at the system level, several works have demonstrated isolators available for integration. Particularly, Jerome Lapointe, et. al. has demonstrated a passive broadband Faraday isolator that allows direct hybrid integration to PICs by butt coupling (Ref. [R2.23]). This isolator requires no lens, no magnet, have wide bandwidth and excellent insertion loss of <1.5 dB. Monolithically integrated options also exist as shown in (Ref. [R2.24]) by direct deposition of the magneto-optic materials.

Furthermore, bulk subminiature Faraday isolators is mature technology and have already been widely deployed in the telecom industries for semiconductor lasers for decades: isolators *with 2.5 mm diameter and 2 mm length* are commercially available since earlier than 2010 and can be co-packaged within laser modules with negligible footprint penalty relative to the overall package size. This is shown in mass produced semiconductor laser modules from Furukawa/Fitel (Ref. [R2.25]), and Anritsu (Ref. [R2.26]).

Overall, we believe system level integration is feasible (as detailed in the reply to Question 14), and we do not foresee road blocking challenges.

Action Taken: We revise the narrative in the Supplementary Information to clearly separate (a) components required by the integrated oscillator from (b) laboratory components used for diagnostics and, in the original submission, seed injection. We clarify that, with pump-modulation turn-key initiation, the circulator is not required and that the laser can be operated without ISO/CIR for application demonstrations. We add discussion in Supplementary Information Section S2 showing that on-chip WDM functionality is readily available on silicon nitride platforms, and ISO can be hybrid integrated.

Referee #2: "10. What's the scalable repetition rate of this integrated Mamyshev oscillator? While the repetition rate frep was 175.5 MHz, and the seed source was at 100-MHz repetition rate. Please elaborate the difference between the two."

Answer: We thank the reviewer for this question. The repetition rate of the integrated Mamyshev oscillator is set solely by the physical round-trip time of its cavity, whereas the repetition rate of the external seed laser (used only in the original seed-assisted start-up experiments) is not a property of the integrated oscillator and does not determine its steady-state operation.

- **Scalable repetition-rate range**

In the present device, the repetition rate 175.5 MHz is determined by the cavity length of the integrated oscillator. Based on our cavity design and simulations using the current gain-section and grating/filter designs, we find that stable mode-locking remains feasible over a substantial range of cavity lengths (see Supplementary Information (Section S3.E)). Specifically, we identify a practical range of approximately 15 cm to 57 cm (for the present gain and filter designs), corresponding to repetition rates of roughly 500 MHz down to 131 MHz. When cavity length is beyond 57 cm, the mode-locking may not be stable, which we attribute to chaotic dynamics when the nonlinear phase shift is excessive. We expect these chaotic pulses in these regions may still have limited use for some applications: imaging, spectroscopy, but will have low temporal coherence. At cavity lengths less than 15 cm, continuous wave lasing emerges and can coexist with mode-locked pulses. This range reflects the balance between round-trip loss, available gain, and the nonlinear switching condition set by the Mamyshev filtering.

We also note that the repetition-rate range can possibly be further extended with design modifications. For example, higher erbium concentration and/or 980 nm pumping can increase the available gain per unit length, which can support shorter cavities (higher repetition rates) without sacrificing threshold margin. Lower waveguide dispersion can act similarly by reducing intracavity chirp. However, the possible cooperative up-conversion effect in the erbium ions may cause excessive unsaturable absorption at higher doping concentrations. This requests future experimental study. Conversely, if a lower repetition rate is desired for higher pulse energy per pulse at fixed average power, additional undoped (masked) waveguide with larger mode area (thus less nonlinearity) can be included to extend the cavity while keeping the gain section unchanged.

- **The seed source repetition rate can differ as it is irrelevant after start-up.**

The 100 MHz repetition rate of the external seed source in the original experiments simply reflects the repetition rate of the commercial fiber MLL that was available in the laboratory. It does not need to match the integrated cavity repetition rate. In the seed-assisted configuration, we gate out *a single* (or a small number of) seed pulse(s) to provide a transient perturbation that brings the oscillator into the basin of attraction of the stable mode-locked state. Once mode-locking is initiated, the integrated oscillator runs autonomously at its own cavity-determined repetition rate (175.5 MHz here), and no further seed pulses are required. In other words, the seed laser serves only as an initiation trigger; *it is not injection-locking the repetition rate or otherwise synchronizing the oscillator.*

Finally, in the revised manuscript, we demonstrate self-seeded initiation by pump-current modulation, which removes the need for any external seed laser altogether. This further underscores that the oscillator repetition rate is an intrinsic property of the integrated cavity and is independent of any external source.

Action taken: In the revised Supplementary Information (Section S3.E), we explicitly clarify (i) the range of repetition-rate achievable with our current gain parameters and grating design, and (ii) that the seed laser repetition rate is unrelated to the oscillator's steady-state repetition rate because only a transient gated pulse is used for initiation.

Referee #2: "11. What factors limit the pulse energy so far?"

Answer: We thank the reviewer for this important question, which is directly related to our core claim of pulse-energy scaling in a photonic-integrated Mamyshev oscillator.

In the present prototype devices, the practical factor that prevents us from pushing to higher energies is the emergence of continuous-wave (CW) lasing components when the pump power is further increased (shown in Fig. R6). Interestingly, we found that the mode-locked pulses still remain stable in this regime: the mode-locked pulses do not disappear or become noisy. Rather, the CW component coexists with the pulse train, which makes it difficult to quantify the pulse energy with our diagnostics as there is a continuous wave background. Such lasing may come from the parasitic lasing between the gratings and the chip facet, or from the slight overlap between the reflection spectrum of the gratings due to sidelobes introduced by fabrication imperfections (jitter in periods).

As a side note, we have made sure that we report pulse energies only from operating conditions where no measurable CW background is present, ensuring that the stated pulse energies are not inflated by a CW contribution.

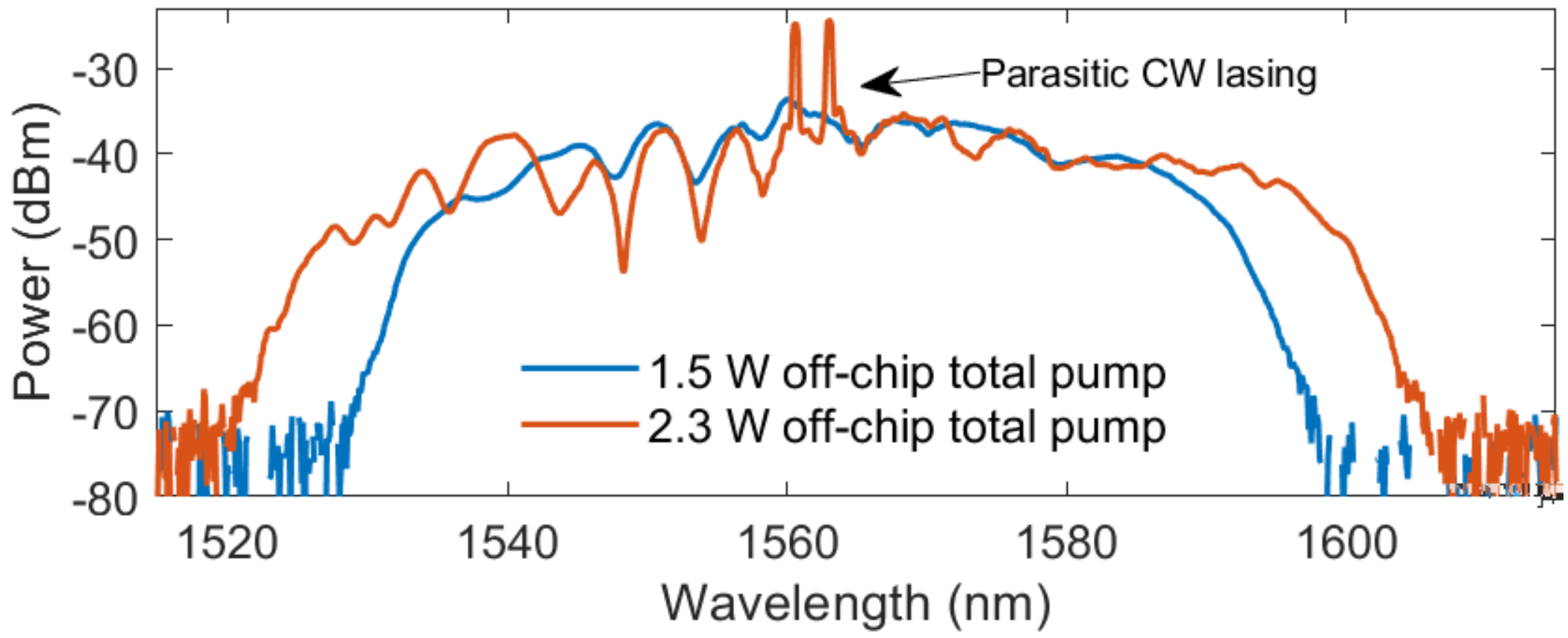

Figure R6 Appearance of parasitic CW lasing when pump power is further increased.

In idealized simulations, where the parasitic facet reflections and reflector artifacts are removed, we observe a second limitation that is expected to become relevant once the experimental CW background is suppressed. At higher pump powers, the single-pulse solution eventually loses stability and the pulse undergoes temporal breakup (pulse breaking), after which stable operation persists as multiple pulses in a single round-trip. In our current design, this transition occurs at pulse energies on the order of approximately 4 nJ.

This behavior is consistent with known scaling limits of high-energy mode-locked oscillators: beyond a certain nonlinear phase shift and gain saturation power, the single-pulse attractor becomes unfavorable and the system accommodates additional intracavity energy by forming multiple pulses. We emphasize that this simulated threshold is not universal and further design optimization (for example, using waveguides with larger mode area and thus less nonlinear coefficient) can shift the stability boundary.

We envisage that to scale pulse energy beyond the single-oscillator stability region, a standard strategy in ultrafast systems, master-oscillator power-amplifier (MOPA) architecture combined with pulse picking, can be utilized. In this approach, the integrated Mamyshev oscillator provides a seed; a pulse picker lowers the repetition rate to raise the energy per pulse; and an integrated amplifier boosts pulse energy further. This is particularly relevant for integrated amplifiers, where available gain is limited and higher seed energy is advantageous. In preliminary experiments (unpublished), we have obtained pulse energies exceeding 10 nJ from a waveguide amplifier. The modulator for pulse picking and the amplifier can both be integrated on the same chip via wafer bonding or transfer printing.

Referee #2: “12. How’s the beam quality M2 of the output beam?”

Answer: We thank the reviewer for raising this point.

In our experiments, the chip output is collected in the *near field* by butt-coupling into a single-mode fiber (Coherent UHNA7). In this configuration, the notion of a free-space M2 factor is not directly applicable at the chip facet because the field is immediately projected into the guided mode in fiber. Instead, what matters experimentally is (i) whether the chip emits into a well-defined spatial mode that can be efficiently coupled into a single-mode fiber, and (ii) whether the field emerging from that fiber is close to an ideal Gaussian beam when collimated.

First, we obtain a fiber-coupling efficiency of approximately 80% from the chip facet into UHNA7 fibers (excluding the splicing loss of UHNA7 to SMF28 fibers). While the coupling efficiency also depends on mode-field matching, achieving this level of coupling into a single-mode fiber is consistent with very high spatial coherence and good overlap with the fiber fundamental mode.

Second, to provide an explicit free-space beam-quality metric, we directly measured the M2 of the collimated output. As described in Supplementary Information Section S11, we send the output from the WDM directly (*of note, no isolator used*) into a cemented pre-aligned fiber collimator (Thorlabs F240APC-1550) and measure the beam diameter as a function of propagation distance using a knife-edge profiler. Fitting the data to the Gaussian-beam propagation model yields an extracted beam quality factor of M2 = 1.08, confirming that the output beam is very close to diffraction-limited.

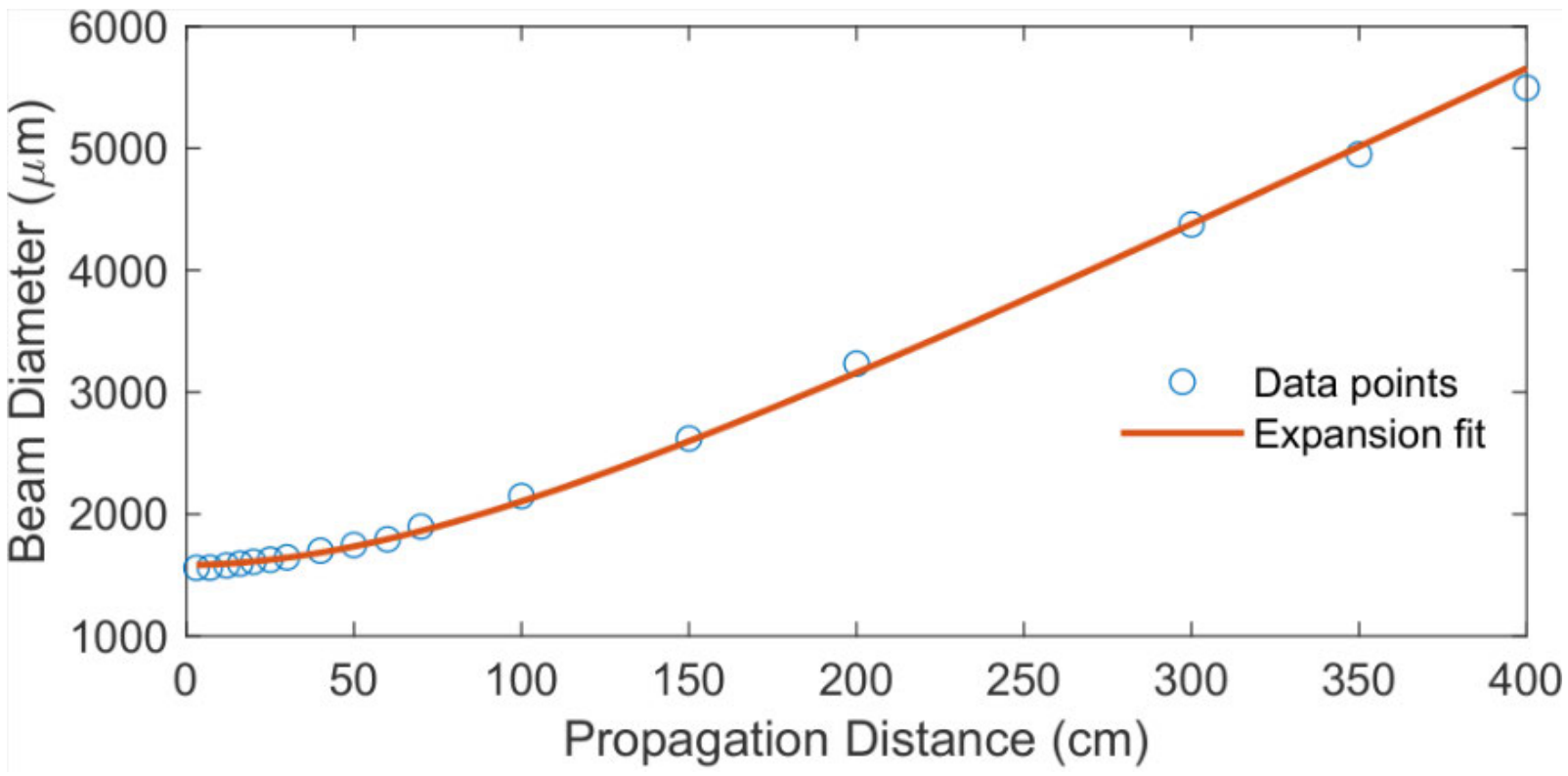

New Figure S24 Characterized beam expansion after emerging from the fiber collimator.

We would like to note that all the power characterization are also performed in the all single-mode fiber coupled system, and the higher order-mode components that can degrade beam quality, if any, are stripped out before reaching the power meters and thus excluded from the evaluation.

Action Taken: We have added a direct free-space beam-quality measurement of the beam emitted from the fiber collimator output and report M2 = 1.08 (Supplementary Information Section S11).

Referee #2: "13. Is simultaneous operation of 26 cavities feasible on a 2 × 1.1 cm² chip? How significant are the thermal effects, and must they be actively controlled?"

Answer: We thank the reviewer for this practical question.

- **Purpose of 26-cavity design: prototyping flexibility, not simultaneous operation**

First, we would like to clarify that in the present work, as described in the manuscript, the inclusion of 26 laser cavities on a single 2 × 1.1 cm² chip is intended for prototyping and efficient parameter screening, not for simultaneous parallel operation. Each cavity implements a different grating configuration, enabling us to rapidly evaluate design parameters and reduce chip area and fabrication cost during development.

Simultaneous operation of all 26 cavities was not attempted or required for any of the experiments reported. In fact, the physical pitch of facet couplers (20 μm) in this design is too small to allow for

practical fiber-array coupling or simultaneous independent access to each cavity; typical fiber array pitches are 127 µm or 250 µm. Thus, our current design is focused on single-cavity operation and exploration of parameter regimes, not high-density parallel lasing.

- **Path to scalable, parallel arrays: layout redesign for access and density**

For future applications requiring economical fabrication or massively parallel operation, the chip layout can be redesigned to ensure sufficient spacing for independent input/output coupling to each cavity. In the Fig. R7, we propose a compact layout in which a single mode-locked laser cavity, using simulation verified bends (70 um) and required spacings (9.5 um), can fit within 0.35 × 0.15 cm^2, **which allows for fabricating more than 1200 laser cores on a single 4-inch wafer.** Individual fiber access can be provided to such a chip, and a massive array of such packaged chip can be created, or alternatively the same design can be repeated on a single chip to create an array while shoreline space for accessing each cavity can be reserved.

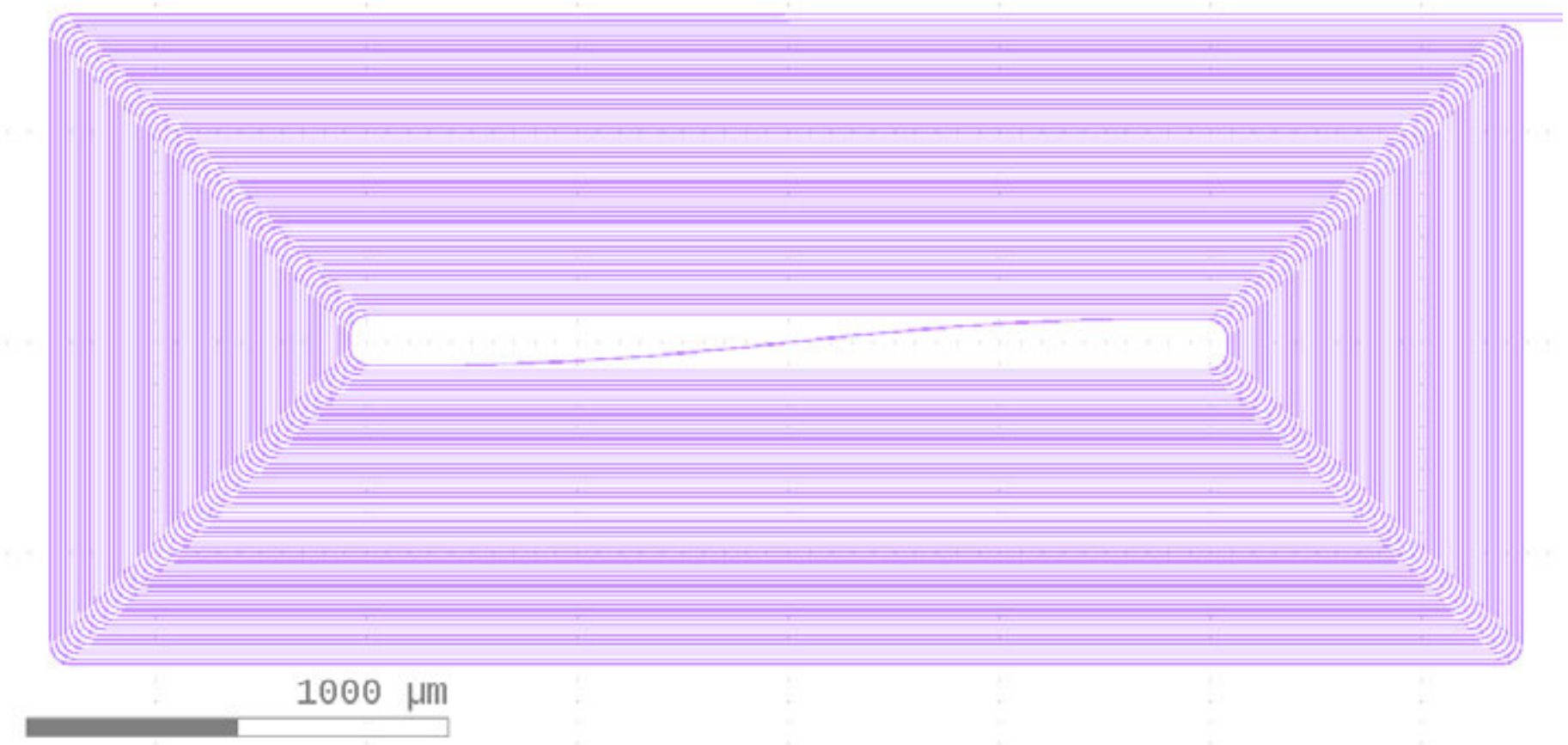

Figure R7 An proposed high density (0.35x0.15 cm^2) layout for a single integrated mode-locked laser.

- **Thermal effects: experimental tests**

Meanwhile in experiments, single Mamyshev MLLs operate stably even without active thermal management (TEC disabled): after reaching thermal equilibrium, lasing is stable without degradation in pulse characteristics or output power, even as the chip mount temperature rises by approximately 8°C. Notably, the THz spectroscopy demonstration in the revised manuscript was performed with thermal control disabled and showed no adverse effects on laser performance. The temperature rise in the chip mount is contributed by the unoptimized thermal conductance of our aluminum mount with a disabled TEC between it and the support metal, and can be further reduced with proper thermal design. Admittedly, the disabled active thermal control makes the initial manual alignment of the optical fibers more difficult before the chip and the mount reached thermal equilibrium, but this is temporary and would not be an issue when the chip is properly packaged. If the laser is used for applications requiring the stability of the beat notes, as like all other lasers, active thermal stabilization is certainly still desirable for stabilizing the intracavity phase and path length.

In addition, our previous work (Ref. [R2.27]) had also demonstrated that erbium-doped silicon nitride continuous-wave lasers remain fully operational at temperatures up to 125°C, a range exceeding the tolerance of many III–V semiconductor lasers. We believe our MLLs should similarly have high tolerance to elevated temperature due to the same gain section.

- **Feasibility of large-scale parallel operation from thermal management perspective**

We estimate the total power dissipation per mode-locked laser to be approximately 1.5 W for full nanojoule performance. For a hypothetical scenario where 26 lasers are operated simultaneously, the total power dissipation would reach ~39 W.

Such high power does pose a nontrivial engineering challenge, however, is within the thermal management capabilities of modern photonic packaging. Commercial quantum cascade laser modules, as well as multimode infrared laser diodes for fiber laser pumping operate in the same power range with established cooling solutions. For example, 30 W 980 nm single-emitter (which means a single III-V chip being the heat source in the package) lasers with 48 W rated maximum heat dissipation in ~12 cm² metal package are commodity items available from multiple vendors including AeroDIODE in France and BWT lasers in China.

In summary, the erbium gain is intrinsically tolerant to elevated temperature and we believe the thermal effects in our integrated lasers are manageable with careful photonic packaging design even for large-scale parallel operation, and do not present a fundamental limitation for the technology.

Action Taken: In the revised Supplementary Information, we clarified that the 26 lasers on the same die are not meant for simultaneous operation, while the individual lasers can be redesigned to achieve similar (even higher) integration density. We have mentioned that we did not observe any degradation in the lasing performance after disabling the active thermal control. In the newly added demonstration experiment for THz-TDS, we also mentioned we performed the experiment with the active thermal control turned off.

Referee #2: “14. What are the primary challenges in realizing integrated directly passive mode-locked lasers?”

Answer: We thank the reviewer for this question, which goes directly to the practicality of turning an integrated mode-locked laser from our current laboratory prototype into a turn-key source.

Because the phrase "directly passive mode-locked" can be interpreted in different ways, we first state our interpretation and then outline the remaining challenges in a manner that is specific to this platform and to the revised results.

Clarification

Here we interpret "integrated directly passive mode-locked laser" as a system that

(i) is passively mode-locked in steady state (no active electro-optic modulation is required to sustain mode-locking),
(ii) can be started without an external ultrafast seed source (self-starting/self-seeded)
(iii) can be implemented as a compact, electrically powered system (with pump delivery and packaging compatible with scalable integration).

Under this definition, (i) is not a problem for the laser architecture we proposed. The main challenges now fall into three categories: (A) how to start it reliably, (B) how to supply pump power, and (C) how to make a robust integrated system.

A. How to start it reliably and repeatably

For Mamyshev oscillators (for both in fiber and on-chip implementations), a key practical challenge is how to reliably reach the mode-locked state on demand. The very strong effective saturable absorption that stabilizes high-energy pulses also suppresses low-intensity noise, making spontaneous startup unlikely without a deliberate perturbation.

In the original submission we used an external seed pulse from a fiber-based MLL as a convenient laboratory method to access the mode-locked attractor. Which is indeed, incompatible with the goal for

a self-contained system. **In the revised manuscript we address this directly: the integrated Mamyshev oscillator can be self-started using only electrical control** by temporarily modulating the pump-diode current after setting the microheater to a suitable operating point (relative WBG detuning). **Importantly, this start-up procedure requires no additional external optical components (no seed laser, no circulator, and no seed injection path)**, and once the mode-locked state is reached the modulation is removed and the oscillator continues to operate passively in steady state.

To make the robustness concrete: we observe a virtually 100% self-starting success rate over a wide pump-power range (623 to 1012 mW launched pump power, corresponding to 467 to 759 mW on-chip), with initiation always occurring within 10 s and often within tens of milliseconds. This is now documented in the revised manuscript via repeated start events and startup-time statistics.

From the standpoint of "directly passive" operation, we view this as the most consequential step because it removes the main practical barrier specific to Mamyshev architectures: reliable, turn-key access to the stable mode-locked state.

B. Pump delivery in a compact, scalable implementation

A second challenge is delivering pump power in a way that is compatible with compact modules and scalable manufacturing. It is worth noting that external pump diodes are the norm for essentially all high-performance ultrafast lasers (solid-state and fiber-based), excluding the semiconductor-based ones that are far-less common on the market. For integrated rare-earth lasers, we believe that the practical question is therefore not "is a pump external?" but rather "is the pump compatible with compact diode technology and realistic system integration?"

We see two well-established routes for this:

i. **Hybrid integration / co-packaging of pump diodes.**

Co-packaging pump diodes with photonic integrated circuits using butt coupling or micro-optics is a mature approach in commercial laser and telecom transmitter modules. WDMs will be required for butt-coupled pump lasers, but they are well established in our and others' works for generic silicon nitride platforms and for amplifiers (Ref. [R2.3], [R2.4], [R2.19], [R2.20]). We have already demonstrated hybrid integration of 1480 nm pump diodes for erbium-based integrated lasers and amplifiers in our prior work (Refs. [R2.21], [R2.22]).

Implementing such packaging requires dedicated mechanical design and assembly process development, but it is an engineering step rather than a physics constraint of the integrated Mamyshev oscillator. We note that such photonic packaging can be readily automatized in the industry.

ii. **Remote pumping via fiber-coupled pump diode modules.**

We also emphasize that remote pump delivery is not merely a laboratory convenience. It is a preferred, widely used system architecture in silicon photonics industry, where external laser/pump modules are intentionally coupled via fiber for reasons of thermal management, qualification, modularity, and maintainability (as detailed in our response to Question 1). In our case, this approach is especially attractive because the pump diode is typically the dominant heat source; keeping it off-chip improves thermal stability while preserving the core benefit of integration: the cavity and pulse-formation mechanism are fully on-chip and are mass-manufacturable.

Thus, compact electrical powering is realistic and can follow either co-packaged pumps or remote pump-module architectures, both of which are established in practice and can be implemented.

C. System integration

A third set of challenges relates to how to design a self-contained optical subsystem. Here it is important to separate what was included for laboratory convenience from what is fundamentally required by the oscillator:

i. Back-reflections and isolators.

In the laboratory setup, an isolator was primarily used to protect against uncontrolled back-reflections from diagnostic instruments. In the revised self-starting configuration, the circulator is removed entirely, and we demonstrate operation in application experiments without ISO/CIR in the optical path. In the scenario where isolation is nevertheless needed, monolithic solutions exist (Ref. [R2.23]), high performance hybrid integrated isolators are recently shown (Ref. [R2.24]) and subminiature co-packaged bulk isolator is a proven technology used in telecom laser modules (Ref. [R2.25, R2.26]).

ii. Thermal control.

The Er:Si_3N_4 gain is highly tolerant to elevated temperatures (supported by our prior high-temperature CW laser results). We also observe stable operation without active temperature control after thermal equilibrium is reached. For product-level integration, thermal engineering remains important mainly because of the pump source, packaging and electronics, not because the oscillator principle is unusually temperature fragile.

The pump power requirement can be further relaxed to lower than 140 mW off-chip power by redesigning the device using less dispersive waveguides of 2 um x 600 nm cross-section (and keep other design parameters, including implantation energy, the same). Although there will be obvious trade-off in pulse energy (66 pJ simulated) due to energy conservation and pulse duration (Compressible to 180 fs FWHM), such further lowered pump power shall further reduce the burden on the thermal management for a hybrid device.

Overall, we believe future system level integration is possible, and we do not foresee road blocking challenges.

Referee #2: “15. (Minor) Formatting suggestions:

1) Fig.1(d), add the scale bar.

2) Fig.2(a) figure caption, define "WDM" (wavelength-division multiplexing).

3) Fig S1, label schematic elements with corresponding references.”

Answer: We thank the reviewer for these helpful formatting suggestions. We have implemented all requested changes to improve clarity and readability.

Action Taken: We added a scale bar to Fig. 1(d), defined WDM as wavelength-division multiplexing in the Fig. 2(a) caption and updated Fig. S1 by labeling the markers with the numbers of the corresponding references.

References:

[R2.1] : K. Gürel, et.al., "Green-diode-pumped femtosecond Ti:Sapphire laser with up to 450 mW average power," Opt. Express 23, 30043-30048 (2015)

[R2.2]: Yoshida, J., et al. "1.3 W fiber-output of highly reliable 14xx-dual-port Raman pump using asymmetric-waveguide laser by GaInAsP/InP electric-field-control-layer." High-Power Diode Laser Technology XXIII. Vol. 13345. SPIE, 2025.

[R2.3]: Z. Qiu et al., "Hybrid Integrated Multi-lane Erbium-doped Si3N4 Waveguide Amplifiers," ECOC 2024; 50th European Conference on Optical Communication, Frankfurt, Germany, 2024, pp. 1721-1724

[R2.4]: Liu, Y., Qiu, Z., Ji, X. et al. A fully hybrid integrated erbium-based laser. *Nat. Photon.* 18, 829–835 (2024). https://doi.org/10.1038/s41566-024-01454-7

[R2.5]: Gil-Molina, A., Antman, Y., Westreich, O. et al. High-power electrically pumped microcombs. Nat. Photon. 19, 1270–1274 (2025). https://doi.org/10.1038/s41566-025-01769-z

[R2.6]: How Industry Collaboration Fosters NVIDIA Co-Packaged Optics https://developer.nvidia.com/blog/how-industry-collaboration-fosters-nvidia-co-packaged-optics/

[R2.7]: Scaling AI Factories with Co-Packaged Optics for Better Power Efficiency https://developer.nvidia.com/blog/scaling-ai-factories-with-co-packaged-optics-for-better-power-efficiency/

[R2.8]: Lumentum External Laser Source (ELS) Module with Ultra-High-Power Laser https://www.lumentum.com/en/products/external-laser-source-els-module-ultra-high-power-laser

[R2.9]: The world's first 100 mW optical power x 16-channel blind mate ELS for co-packaged optics https://www.furukawaelectric.com/en/release/2024/comm_20240327.html

[R2.10]: OIF Announces External Laser Small Form-Factor Pluggable (ELSFP) Implementation Agreement, Paving the Way for Advancements in Co-Packaged Optics Applications: https://www.oiforum.com/oif-announces-external-laser-small-form-factor-pluggable-elsfp-implementation-agreement-paving-the-way-for-advancements-in-co-packaged-optics-applications/

[R2.11]: RP2350 microcontroller, Raspberry Pi Ltd.: https://www.raspberrypi.com/products/rp2350/

[R2.12]: ESP32-S3 dual-core XTensa MCU, Espressif Systems: https://www.espressif.com/en/products/socs/esp32-s3

[R2.13]: STM32F3 Series of mixed-signal MCUs, STMicroelectronics NV: https://www.st.com/en/microcontrollers-microprocessors/stm32f3-series.html

[R2.14] Samartsev, Igor, Andrey Bordenyuk, and Valentin Gapontsev. "Environmentally stable seed source for high power ultrafast laser." Components and Packaging for Laser Systems III. Vol. 10085. SPIE, 2017.

("The laser is self-starting." is claimed in the abstract, while the exactly same method to start is employed: "For the purpose of pulse initiation we modulate the pump source with a train of pulse of microsecond duration at tens of kilohertz repetition rate.")

[R2.15] Haig, Henry, et al. "Megawatt pulses from an all-fiber and self-starting femtosecond oscillator." Optics Letters 47.4 (2022): 762-765.

("self-starting." is claimed in the title, while the exactly same method to start is employed: "Specifically, we use a simple function generator to create a 70 kHz square wave signal that drives the pump power between 0 and 8 W.")

[R2.16] Wang, Chaoran, Xingliang Li, and Shumin Zhang. "Automated start-up and extinction dynamics of a Mamyshev oscillator based on a temperature-dependent filter." Laser & Photonics Reviews 17.7 (2023): 2201016.

("the narrow filter interval could produce self-starting" is claimed in the conclusion, and heater tuning is performed at each start)

[R2.17]: Liu, Zhanwei, et al. "Megawatt peak power from a Mamyshev oscillator." Optica 4.6 (2017): 649-654.

[R2.18]: Ma, Chunyang, et al. "Ultrabroadband, few-cycle pulses directly from a Mamyshev fiber oscillator." Photonics Research 8.1 (2019): 65-69.

[R2.19]: Gao, Ge, et al. "Silicon nitride O-band (de) multiplexers with low thermal sensitivity." *Optics Express* 25.11 (2017): 12260-12267.

[R2.20]: Cheung, Stanley S., and Michael RT Tan. "Silicon Nitride (Si3N4) (De-)Multiplexers for 1-μm CWDM Optical Interconnects." *Journal of Lightwave Technology* **38.13** (2020): 3404-3413.

[R2.21]: Osornio-Martinez, C. E., et al. "Monolithically integrated erbium-doped polycrystalline Al2O3 waveguide amplifier on silicon photonics platform." Optics express 33.11 (2025): 23491-23502.

[R2.22]: Mu, Jinfeng, et al. "High-gain waveguide amplifiers in Si3N4 technology via double-layer monolithic integration." Photonics Research 8.10 (2020): 1634-1641.

[R2.23]: Wei Yan, Yucong Yang, Shuyuan Liu, Yan Zhang, Shuang Xia, Tongtong Kang, Weihao Yang, Jun Qin, Longjiang Deng, and Lei Bi, "Waveguide-integrated high-performance magneto-optical isolators and circulators on silicon nitride platforms," Optica 7, 1555-1562 (2020)

[R2.24]: Lapointe, J., Coia, C., Dupont, A. et al. Passive broadband Faraday isolator for hybrid integration to photonic circuits without lens and external magnet. Nat. Photon. 19, 248–257 (2025). https://doi.org/10.1038/s41566-024-01601-0

[R2.25]: Hiroyuki Ishii, et al., "Narrow Spectral Linewidth Integrable Tunable Laser Assemblies for Digital Coherent Optical Communications", *Furukawa Electric Review*, No. 56, 04, 2025, Available at: https://www.furukawaelectric.com/en/rd/review/fr056/fr56_04.pdf

[R2.26]: Anritsu AF4B series, datasheet available at: https://dl.cdn-anritsu.com/en-us/test-measurement/files/Brochures-Datasheets-Catalogs/Brochure/AF4B_SERIES_type_D_E_C_3.pdf

[R2.27]: Ji, Xinru, et al. "Full C-and L-band tunable erbium-doped integrated lasers via scalable manufacturing." arXiv preprint arXiv:2501.06936 (2025).

Referee #3 (Remarks to the Author):

I co-reviewed this manuscript with one of the reviewers who provided the listed reports.

Referee #4 (Remarks to the Author):

This is a strong and carefully characterized system result: an integrated Mamyshev oscillator that reaches nanojoule-class energies at 175 MHz with sub-200 fs compressed pulses, narrow repetition-rate/optical linewidths, and direct supercontinuum generation. It is being somewhat oversold however.

Answer: We thank you for the careful reading and recognizing the technical strength of the result. We also sincerely appreciate your concern that some of the original framing could be read as overselling.

In the revision, we now demonstrate turn-key initiation of the mode-locked state without reliance on any external ultrafast seed source or the complex off-chip optical arrangements used previously. We have also clarified the scope in the performance-comparison statement to reduced ambiguity.

Referee #4: "Major concern: system complexity for start-up.

The reported device achieves impressive nJ-class pulses. However, realizing this performance requires a substantial off-chip seeding complexity (fiber MLL, programmable filter, EDFA, synchronized pulse picker) to inject a single 15–45 pJ pulse, or, alternatively, an extended-cavity Q-switching arrangement with higher pump power (external modulator + circulator). This is in tension with the paper's "reduced-complexity, fully integrated" frame. A stronger result (which would undoubtable be of worthy of broad dissemination, e.g. in Nature) would demonstrate robust self-start without external seed or extended cavity."

Answer: We appreciate your recognition of one key advancement in this work, namely the nJ-class pulse output, and your assessment that a self-starting implementation would be worthy of broad dissemination.

We also thank you for raising the important issue of start-up complexity and agree with the underlying concern: in the original submission, the off-chip seed-injection chain (fiber MLL, programmable filter, EDFA, pulse picker, circulator) and the components for the alternative extended-cavity Q-switching approach could reasonably be viewed as inconsistent with the goal of a miniaturized and scalable system and negate the entire advantage of a mode-locked laser on chip.

To address this, the revised manuscript experimentally demonstrates a new, turn-key self-starting (self-seeding) procedure that **eliminates the need for any external ultrafast seed laser and off-chip optical components for start-up (no external seed or extended cavity, exactly as you commented).** Mode-locking is initiated solely by temporarily modulating the pump power via the drive current of the 1480 nm pump diodes, after setting the on-chip microheater to an appropriate operating point (i.e., establishing a suitable relative offset between the two on-chip WBG passbands). After the mode-locked state is reached, the modulation is then disabled and the oscillator continues to operate autonomously in steady-state mode locking. The operating point remaining tunable after starting via the pump power and heater settings to reach desirable pulse energy and spectrum.

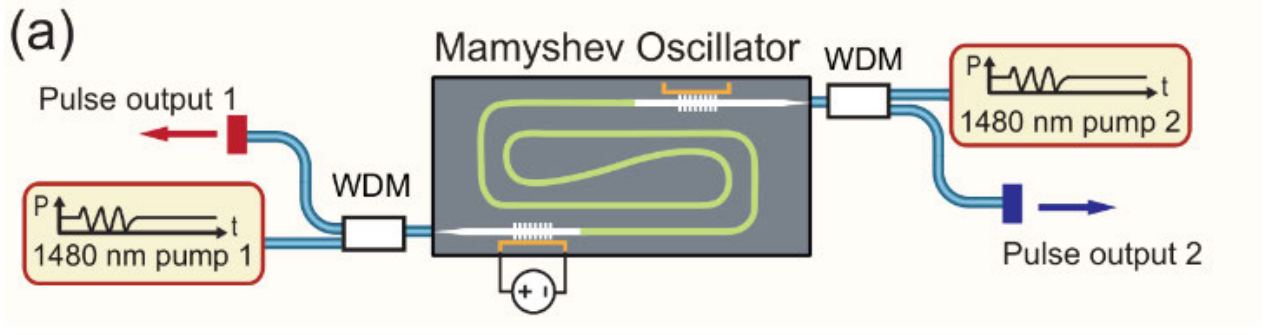

Revised Figure 2 (a): Experiment setup for operating the intergrated Mamyshev oscillator with no external MLL for seeding.

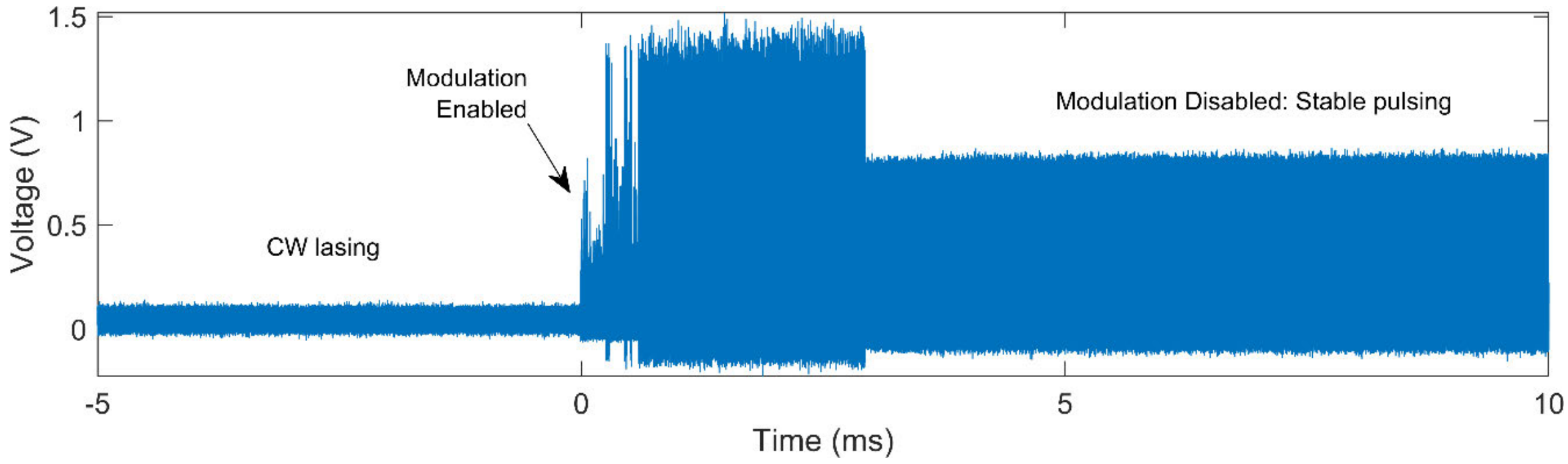

New Figure S15: Pulse emergence dynamics from enhanced noise after enabling modulation, and the transition into stable pulsing immediately after switching off modulation.The entire process is completed in approximately 3 ms.

Crucially, **the required electronics are also straightforward** and are already implemented using generic, commercially available diode-laser drivers in our experiments. The pump modulation is a simple square-wave current modulation at 1.2-2.8 MHz. Generating such a few-MHz square-wave signal is **trivial for modern microelectronics**. For example, one can directly use the PWM output from a programmable microcontroller like RP2350 (cost <$1, [R3.1]), ESP32 (cost $1 to $2, [R3.2]), STM32F3 (cost $2, [R3.3]) which can also simultaneously carry out other control functionalities (Shown on Figure R8). Applying it to the low-voltage, 1-2 A-class diode pump is likewise straightforward, either through built-in modulation/enable functionality of commercial drivers (as used here) or, if desired, via a simple external switching stage (for example a MOSFET-based shunt switch, cost <$0.3) and a constant current driver. Therefore, the start-up procedure adds neglectable cost and complexity to the system and is compatible with compact, embedded implementations.

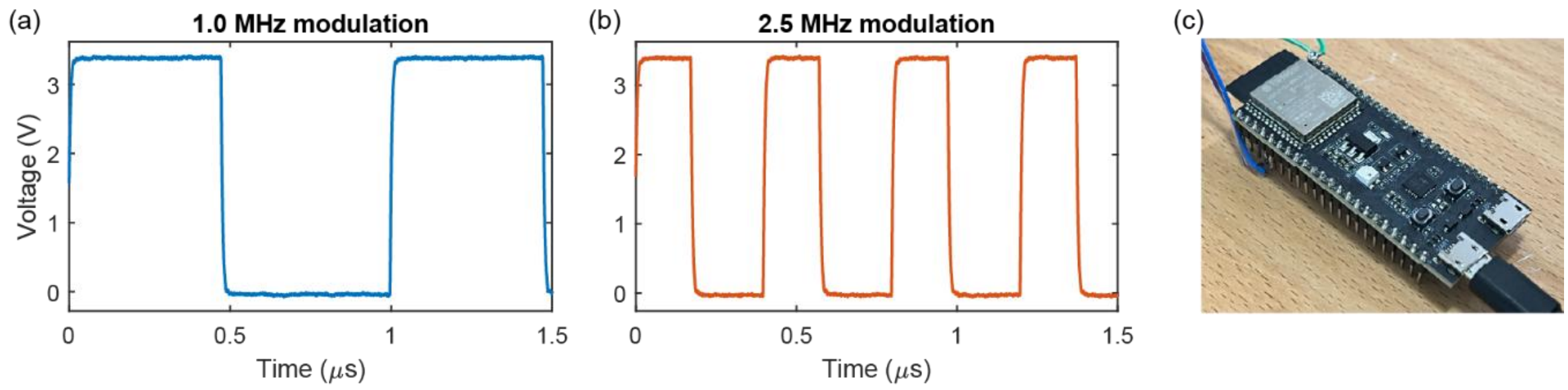

Figure R8 (a,b) Measured modulation waveform simutanously generated at (a) 1.0 MHz and (b) 2.5 MHz from a single low-cost ESP32-S3 microcontroller. (c) Photograph of the development board used for the waveform generation demonstration. The entire controller development board (ESP32-S3-DevKitC-1) cost ~$5 on AliExpress and is capable of simutanously generating up to 4 independent square wave modulation signals and carry out the starting control procedure autonomously.

Experimentally, this approach yields **a virtually 100% success rate**. We demonstrate reliable self-starting across a wide pump-power range, from 623 mW to 1012 mW launched pump power (467 mW to 759 mW on-chip pump power). Across this range, the oscillator always reaches the mode-locked state within 10 s after enabling modulation, and in many cases initiates on timescales of tens of milliseconds. **Across hundreds of starting demonstrations, we have not observed any sign of device damage or degradation associated with the transient during initiation, underscoring the robustness of the procedure.**

We include in the revised manuscript and Supplementary Information:

(i) Representative start-up transient dynamics (Supplementary Information S5),
(ii) Repeatability over multiple independent start events (Fig. 2(e)),
(iii) Spectra confirming convergence to the same mode-locked state as the seed-assisted approach for identical cavity settings (Supplementary Information S5, Fig. S17),
(iv) Statistics of the time-to-lock versus pump power (Supplementary Information S6, Fig. S18).

We have also employed this self-seeding approach for the new demonstration experiment in THz spectroscopy with expected result consistent with external seeded operation.

The procedure to start the Mamyshev oscillator is readily automatized simply as following:

- Turn on the pump power.
- Set the microheaters.
- Enable pump modulation.
- Wait for a time less than 10 s, commonly <1 s.
- Disable pump modulation.
  Now the laser shall be mode-locked and ready for use.
- Optional: Ramp up or ramp down the pump power and change heater setpoint to target a desired pulse energy and output spectrum.

We note that we use "self-seeded" and "self-starting" interchangeably, as consistent with terminology in literature (Ref. [R3.4], [R3.5]) about fiber-based Mamyshev oscillators that uses the same pump modulation technique as well as Ref. [R3.6] using filter tuning to initiate mode-locking. Despite this admittedly do not satisfy a very strict definition of "self-starting", that is being able to mode-lock immediately when the constant pump power is applied, we believe it is equivalent in practice as it does not compromise on the performance in any way or add significant complexity. In these schemes, everything is electronically controlled and readily automatized. We hope these additions can directly address your concern.

For completeness, we would like to clarify that a significant part of the complexity in the original seed chain was intentionally included to degrade the seed pulse (via spectral filtering) to emulate a more realistic, lower-performance seed source rather than to rely on a high-performance fiber-based femtosecond laser. Nevertheless, we agree that any dependence on an external ultrafast seed laser or a switched extended cavity is undesirable from an application perspective and should not be a prerequisite for operating the integrated oscillator, which is why we made self-starting (turn-key initiation) a central element of the revision.

Action Taken: We have edited the manuscript extensively to put emphasis on the new self-starting method based on temporary pump-current modulation, demonstrating mode-locking initiation without any external seed laser:

i. The abstract, introduction and manuscript narrative are updated to emphasize self-seeding/self-starting capability and to remove any implication that external MLL seeding is a required for operation.
ii. We revised Figure 2 in the main text and the experimental setup description in the Supplementary Information S3 to highlight the repeatability and reflect the self-starting configuration (no need for seed injection path). We also commented on the generation of the modulation waveform with low-cost electronics.
iii. We have also clarified in the Supplementary Information S3 that in the externally seeded configuration, the Menlo ELMO was used for availability in the lab and was deliberately degraded, and that lower-performance pulsed sources are sufficient for initialization when external seeding is used.

Referee #4: "Comparison claims. The manuscript states ">2 orders of magnitude" improvement over prior PIC ultrafast sources; the authors' own table appears closer to 1 order (15x). Please reconcile the headline with the table (define exactly which prior class is being compared)."

Answer: We thank you for pointing out this inconsistency. We agree that, as written in the original submission, the headline claim and the Supplementary comparison table can create confusion.

In the revised manuscript, we therefore explicitly define the comparison underlying the ">2 orders of magnitude" statement as **photonic integrated circuit (PIC)-based ultrafast mode-locked sources**, i.e., lasers whose cavity and pulse-formation mechanism are realized in a PIC platform intended to be compatible with integration of passive routing and filtering, as well as a broader suite of further on-chip functions (for example, monolithic MHz piezoelectrical tuning, pulse compression, nonlinear wavelength conversion, optical sampling, and supercontinuum generation).

In the Supplementary Information S1 table, we also listed several monolithic semiconductor mode-locked lasers based on specialized large-mode-area geometries (e.g., SCOWL-type or tapered designs; Refs. [9,10,25] in the SI) for reference. While these devices can deliver higher pulse energies than typical PIC-based demonstrations (still more than an order of magnitude lower than our work), they are not representative of PIC-based integrated photonics platforms as they rely on specialized waveguide and cavity geometries (e.g., thick guiding slabs and/or strongly asymmetric mode fields) that are not compatible with standard foundry PIC process flows and difficult to develop additional on-chip functionality. They may require specialized coupling optics (e.g., cylindrical-lens) rather than straightforward fiber-to-PIC, PIC-to-PIC butt coupling. Developing further functionalities with them can be significantly more challenging than the PIC-based lasers.

When restricting the comparison to PIC-based sources, the highest reported on-chip pulse energy in our table is 2.6 pJ (Ref. [1] in the SI). Compared with our demonstrated >1 nJ pulse energy, this corresponds to a >300x improvement, i.e., well beyond two orders of magnitude. We have revised the statement accordingly so that it explicitly refers to this PIC-based comparison class.

This comparison category is also consistent with how prior literature has framed performance benchmarks for PIC-based lasers. For example, the pioneering work by Qiushi Guo et al. (*Science* 382, 708-713, 2023; Ref. [1] in the SI) reported an integrated actively mode-locked laser on lithium niobate PIC with on-chip pulse energies exceeding 2.6 pJ and peak power exceeding 0.5 W, which were the top performance among the PIC-based lasers. It's stated that it "*offers the highest output pulse energy and peak power of any integrated MLLs in nanophotonic platforms*" at the time of publication (2023).

We also note that several of the specialized monolithic semiconductor devices listed previously can exhibit other severe limitations in pulse coherence, peak power and compressibility due to high cavity loss and strong nonlinear chirp accumulation (Refs. [9,25] in the SI), which can constrain their utility in other ways. However, to avoid over-extending the argument, our revised headline comparison is based strictly on the clearly defined PIC-based class and the single metric of on-chip pulse energy.

Action taken: In the revised manuscript and Supplementary Information, we now state explicitly that the ">2 orders of magnitude" comparison is made among PIC-based integrated ultrafast sources.

In the Supplementary Information S1, we have also stated: "For completeness, we present a comprehensive list of integrated ultrafast sources, including some that may not strictly be considered photonic integrated circuits (PICs) by today's standards due to the compatibility of fabrication processes and mode confinement."

Referee #4: "Summary of the key results

- A Mamyshev oscillator is implemetned on Er doped SiN PIC, which uses a complex off-chip seeding setup.

- Dual 1480 pumps provide ~1 W each. A third mode locked laser seed starts with a single pulse. (alternative Q switch is also shown)

- ~20 dB BW of ~50-60nm

- 20 RT for build-up

- ~1 nJ pulse energy is an order of magntiude larger than previous PIC approaches."

Answer: We thank you for providing this concise overview of the main results and for clearly identifying the aspects that, in the original submission, most affected the perceived system-level practicality. We agree that the initial manuscript could be read as relying on a complex off-chip seeding setup, and we have therefore revised the work to directly address this point.

In the revised manuscript, we now demonstrate turn-key initiation of mode-locking **without any external ultrafast seed laser and without additional off-chip optical components** for start-up (i.e., no seed-injection chain and no extended-cavity/Q-switched arrangement). Mode-locking is initiated solely through a temporary electrical modulation of the pump-diode drive current using a commercial laser driver, after which the oscillator operates autonomously in the steady-state mode-locked regime. We also emphasize that this turn-key initiation is realized over a wide operating range and **is not restricted to operation at the highest pump powers.** For applications that do not require the highest pulse energy, we show self-starting and stable mode-locked operation with total launched pump powers of 623 mW (with correspondingly lower on-chip pump power), while maintaining repeatable initiation and stable output.

Finally, we have clarified the comparison framing. In the revised version, the headline scaling statement is explicitly benchmarked against PIC-based ultrafast mode-locked sources, for which the highest reported on-chip pulse energies are in the few-pJ range, consistent with prior publications (SI Ref. [1]). Relative to these, our demonstrated >1 nJ pulse energy represents an improvement well beyond two orders of magnitude. The earlier impression of an approximately one-order-of-magnitude improvement arose from mixing this PIC-based class with specialized monolithic semiconductor large-mode-area devices in the same table; we now provide a clear rationale for the comparison class used.

Referee #4: "Suggested improvements:

- Language should be softened? "seed-assisted integrated mamyshev oscillator achieving nJ-class pulses"?"

Answer: We thank you for the suggestion to soften the language by describing the system as "seed-assisted." We agree that this phrasing would have been appropriate for the original submission, where an external seed laser was used as the primary start-up method.

However, for the revised manuscript, we now demonstrate turn-key self-seeding of the integrated Mamyshev oscillator without any external ultrafast seed source, achieved solely through a temporary electrical modulation of the pump-diode drive current. Since the oscillator can be initiated without seed pulses and then operates autonomously in steady-state, we believe it is more precise to describe the device as a self-seeded (turn-key) integrated Mamyshev oscillator, rather than "seed-assisted."

Referee #4: "- Self starting is essential. I don't think lasers need to be integrated, they can be off-chip, but if the thing needs very complex off-chip optics (MLL or circulators, modulators etc.), then the reduced complexity argument can't be made."

Answer: We thank you for emphasizing this point, and we agree with the underlying logic: if achieving mode-locking requires a complex off-chip optical system (external MLL seeding, circulators, modulators, extended cavities), then a reduced-complexity argument would not be justified. We also appreciate your clarification that the pump lasers themselves do not need to be integrated on the same chip at this stage.

In the revised manuscript, we directly address the critical part of your concern: start-up complexity, by demonstrating turn-key initiation of mode-locking with only electrical control, **without any external**

ultrafast seed source and **without additional off-chip optics** for start-up. Specifically, we initiate the mode-locked state solely via temporary modulation of the pump-diode drive current using a commercial laser driver, after which the oscillator runs autonomously in steady-state mode locking.

We also clarify the role of isolators and circulators in the original experimental setup. In the seed-assisted configuration, a circulator was required to inject the seed pulse and separate the output, and isolators were used primarily to protect the oscillator from back-reflections originating from diagnostic instruments. In the revised self-seeded operating mode, these components are not required for start-up or steady-state operation. More generally, in a future system integration, back-reflection can be mitigated through standard measures (e.g., angled/AR-coated interfaces and return-loss management in the downstream optical path), removing the need for isolators in many applications.

Referee #4: "- I would be interested in knowing what the single-pass characteristics are."

Answer: We thank you for raising this interesting question. We agree that understanding the single-pass behavior of the erbium-doped waveguide is valuable, both for insight into the oscillator dynamics and for assessing the potential of the gain section as a discrete amplifier element.

To address this, we added a new section in the revised Supplementary Information (Section S12) that explicitly analyzes the gain waveguide operated in single pass. In this analysis, we simulate the propagation of a train of input pulses through the erbium-doped waveguide with parameters chosen to match the experimental and modeling conditions of the oscillator. Specifically, we consider pulses with 3 ps duration and 10 W peak power, which are representative of the seed pulses used in the original seed-assisted initialization experiments. The gain section is pumped under the same conditions as in the oscillator simulations, and the input pulse train is assumed to have a repetition rate of 175.5 MHz to match the cavity free spectral range. (More details in the answer to the next questions.)

Action Taken: We have added a new section in the revised Supplementary Information (Section S12: "Single-pass pulse propagation in the gain section"), dedicated to the simulation of pulse propagation in the doped section in a "single-pass" manner, where the following figures showing the simulation results of the "single-pass" propagation are included.

Referee #4:

"Questions that I believe need to be answered in the text before proceeding further:

- Does pump-to-output sum the two ports?"

Answer: We thank you for requesting clarification on this point, as we agree the definitions should be explicit.

For the pump-to-output power conversion efficiency, we sum the optical output powers from both output ports, since both ports represent useful extracted laser power from the same cavity. In contrast, when reporting average output power and pulse energy, we report the values for a specified output port (the one used for the corresponding measurement), and we do not sum both ports unless explicitly stated.

We also note that having two simultaneous, inherently synchronized outputs from the same cavity can be advantageous for certain applications (for example, pump-probe measurements or THz time-domain spectroscopy). In future designs, if a strongly asymmetric output distribution is desired, the relative power in the two ports can be engineered by tailoring the spectral response of the filtering elements and output coupling conditions so that one port is preferentially extracted, or alternatively by interleaving the two outputs using a Mach-Zehnder modulator.

Action taken: In the revised manuscript, we now explicitly state that the pump-to-output efficiency is computed "when the powers are summed for two outputs."

Referee #4: “- What is the start probability vs pump power, and the distribution of time-to-lock?”

Answer: We thank you for requesting these quantitative start-up metrics. We agree that both the start probability and the time-to-lock distribution are important for assessing practical operation, and we have added explicit measurements and definitions in the revised manuscript and Supplementary Information.

- **Self-starting by pump-current modulation (revised manuscript).**

In the revised manuscript, we focus on the electrically initiated self-starting method based on temporary pump-current modulation, which removes the need for any external seed source. As shown in the revised Supplementary Information (Section S6 and Fig. S18), we characterize the distribution of time-to-lock as a function of pump power. We define the time-to-lock as the elapsed time from the first modulation edge to the generation of the mode-locked pulse, detected with an optical bandpass filter away from the parasitic lasing band. We have manually confirmed on oscilloscope that each recorded event corresponds to a successful landing in the mode-locked state when the modulation is switched off (with a 100% success rate).

In this way, we observe robust turn-key initiation over a wide total pump-power range, from 623 mW to 1012 mW launched pump power (corresponding to 467 mW to 759 mW on-chip). Across the accessible power range, the time-to-lock is always below 10 s, and in many cases occurs on timescales of tens of milliseconds. Notably, the time to lock is much longer than the cavity round-trip time, due to the stochastic nature of the seeding from noise.

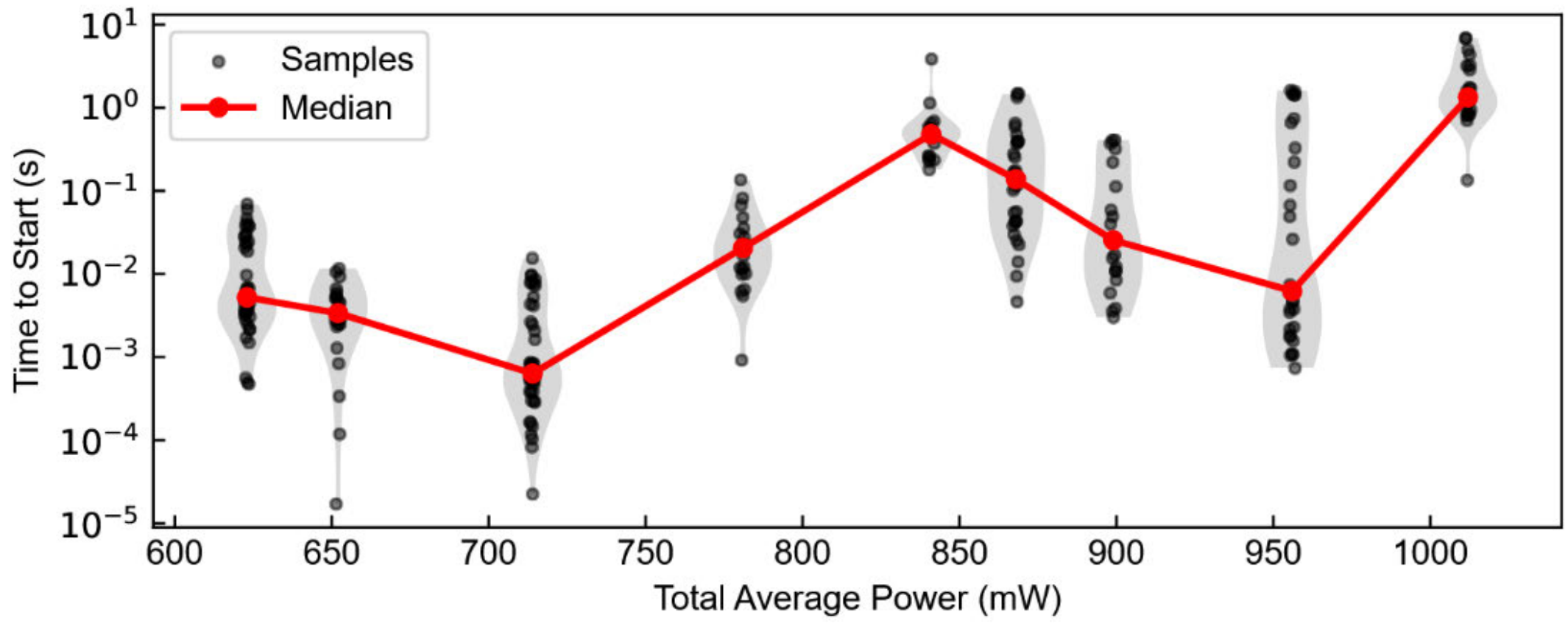

New Figure S18 Violin plot of the time to start mode-locking from the first modulation edge applied to the pump laser diodes, overlaid with the median time to start.

The start probability is effectively binary: within this power range the success rate is virtually 100%, whereas outside the range showed the start probability drops sharply, and we do not observe self-starting even for extended observation times (>1 min).

- **External seeded initialization (original manuscript).**

For completeness, we also summarize the behavior observed in the original seed-assisted configuration. When the total on-chip pump power exceeds a threshold (empirically in the range of approximately 0.6 W to 1.0 W, depending on the grating-separation setting), start-up is highly reliable: virtually every injected seed pulse triggers the transition into the mode-locked state. In this regime, the injected seed pulse effectively directly evolves into the first intracavity pulse and the subsequent pulse train builds up within a few round trips, consistent with the "coherence memory" behavior reported in Ref. [61] of the SI. The effective time-to-lock is therefore on the order of a few times of cavity round-trip time (~100 ns). Below this threshold, the start probability drops significantly and becomes sensitive to parasitic

continuous-wave/Q-switched lasing (often no start after thousands of seed pulses is observed unless alignment improved), which depends on coupling and back-reflection conditions. This binary behavior may be attributed to competition between different modes for the CW parasitic lasing.

Action taken: In the revised Supplementary Information, we have added Section S6: "Time to start for self-seeding", we added a clear definition of time-to-lock, stated the virtually 100% starting probability in the power range and visualized time-to-lock distributions versus pump power for pump-modulation initiation (Figure S18). We have also mentioned in Section S3 that hundreds of nanoseconds are needed to start when using external seeding.

Referee #4: "- What minimum seed energy (on-chip) reliably starts ML?"

Answer: We thank you for asking this question. In the revised manuscript we shift the emphasis to the pump-current-modulation initiation method, which removes the need for any external seed pulse.

Nevertheless, for completeness, we clarify here (and highlight more explicitly in the revised text) the seed-energy requirement observed in the original seed-assisted configuration. As described in Supplementary Information Section S3.E, the minimum on-chip seed energy required to reliably initiate mode-locking is estimated to be approximately 15 pJ in experiment. This range depends on the injected pulse width (and therefore peak power) and on the cavity condition at the time of injection, in particular the level of parasitic continuous-wave/Q-switched background, which is influenced by coupling and back-reflection conditions.

Action taken: In the revised manuscript and Supplementary Information S3.E, we make this value easier to locate, while emphasizing that external seeding is no longer required for routine turn-key operation.

Referee #4: "- If you route the shaped seed through the doped section without feedback/filters, then compress: what are the output energy, spectrum, etc.?"

Answer: We thank you for this very insightful question. We agree that the single-pass, amplifier-like behavior of the erbium-doped waveguide is important both for interpreting the oscillator operation and for assessing alternative architectures (e.g., seeded amplification without a cavity).

We would also like to note that in the new revision of the manuscript, we have shifted focus to the self-seeded operation of the laser. In this case the seed from outside of the cavity is no longer required.

Nevertheless, to answer your question directly, we added a new section in the revised Supplementary Information (Section S12) where we simulate propagation of a pulse train through the erbium-doped gain waveguide in a purely single-pass configuration (i.e., without cavity feedback or the Bragg grating filtering). We use input pulses of 3 ps duration and 10 W peak power, chosen to be representative of the shaped seed pulses employed in the original seed-assisted initialization experiments. The gain waveguide is pumped under the same conditions as in the oscillator simulations, and the repetition rate is set to 175.5 MHz to match the laser's operating point.

In the simulation, we observed the pulses undergo simultaneous amplification and strong self-phase-modulation broadening. Owing to the interplay between gain and dispersion, the pulse evolution remains stable (no pulse breaking observed) and exhibits behavior comparable to self-similar propagation in gain media, as described in fiber systems (Ref. [R3.7]). Under these conditions, the single-pass output reaches 1.57 nJ pulse energy, with an optical spectrum broadened to spanning approximately 50 nm and a predominantly linear chirp (and thus is, in principle, compatible with simple dechirping). The output pulses are compressible to approximately 150 fs.

We note that this observation also suggests an interesting *complementary* pathway to high-energy ultrafast pulse generation on-chip, based on a master-oscillator/power-amplifier concept. A suitable seed source with low performance can be amplified in an erbium-doped waveguide with sufficient

nonlinearity and normal GVD. Thanks to the high output power of the waveguide amplifier, significant pulse energy and narrow dechirped pulse can be achieved, however, the coherence and noise performance in such a configuration would directly depend on the quality of the seed and coherence in the pulse propagation dynamics. We believe this can be the subject of a follow-on work.

Action Taken: We have added a new section in the revised Supplementary Information (Section S12: "Single-pass pulse propagation in the gain section"), dedicated to the simulation of pulse propagation in the doped section in a "single-pass" manner, where the following figures showing the simulation results of the "single-pass" propagation are included.

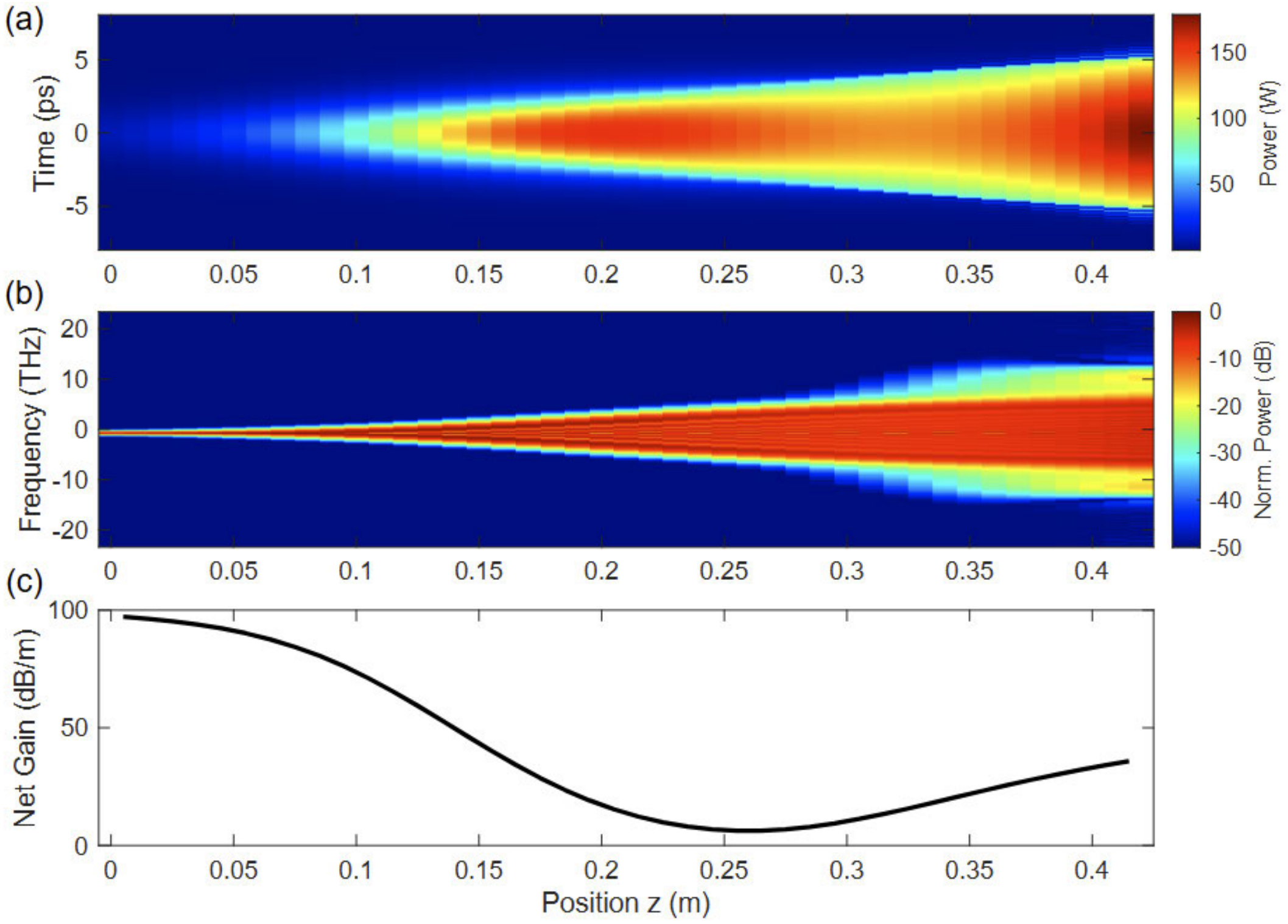

New Figure S25. Single-pass pulse evolution in the gain section: (a): Time domain evolution along the propagation. (b): Frequency domain power spectral intensity of the pulse along the propagation. (c): Net gain as a function of the propagation distance

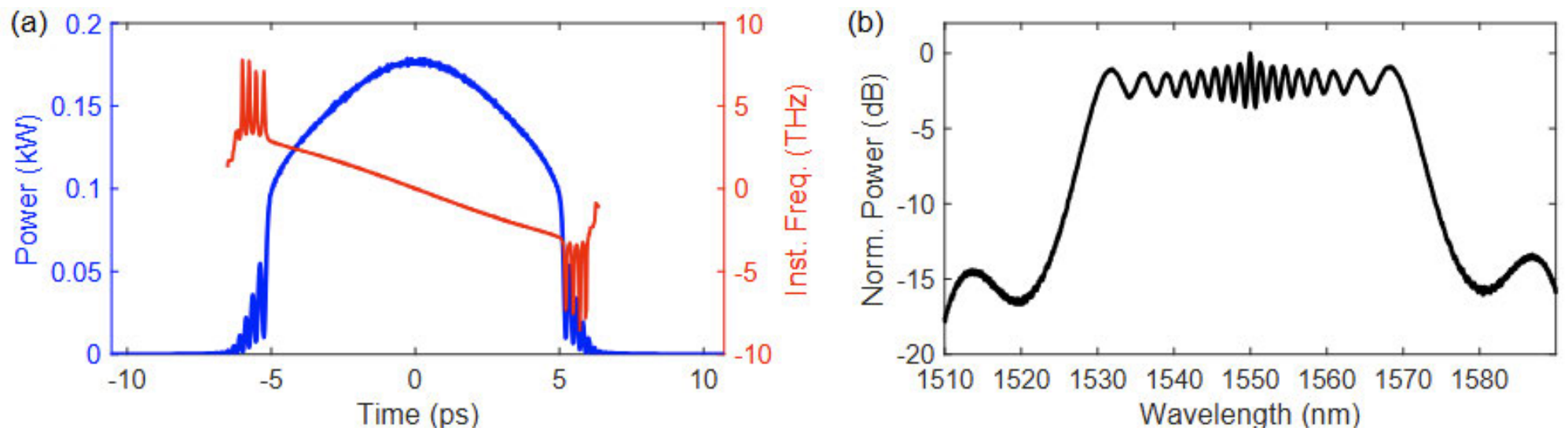

New Figure S26. Single-pass amplified pulse output from the gain section: (a): The pulse output in time domain, showing the instantaneous power and the instantaneous frequency. (b): The output power spectral density in frequency domain.

Referee #4: “- Back-reflections: specify the target return-loss needed for self-start, with supporting sim/measurement (today's parasitics appear ~-30 dB; what level enables noise-start?). Outline a realistic path (angled/AR edge couplers, etc.) and expected margin.”

Answer: We thank you for raising this important point.

First, we note that in the revised manuscript, we shift the emphasis to turn-key self-starting enabled by temporary pump-current modulation. This approach is experimentally robust with our present chip to fiber coupling technique using butt coupled UHNA fibers, which **have return-loss of approximately 25-30 dB**. In other words, in the revised starting approach, we do not require any additional engineering for lowering return loss to achieve turn-key self-seeding of mode-locking.

Regarding the specific question of pulse emergence from noise background without any perturbation. We have addressed this quantitatively by simulations that explicitly include facet back-reflection as a correction to the grating reflectivity. As shown in Figure R8, when pumping approximately 470 mW on-chip power from each side (a power level that is readily achievable with single butterfly packaged diodes), we find that the starting from noise becomes possible when the facet return loss is improved to approximately 42 dB to 43 dB. Compared to our current return-loss level, an improvement of 12 dB to 18 dB is required.

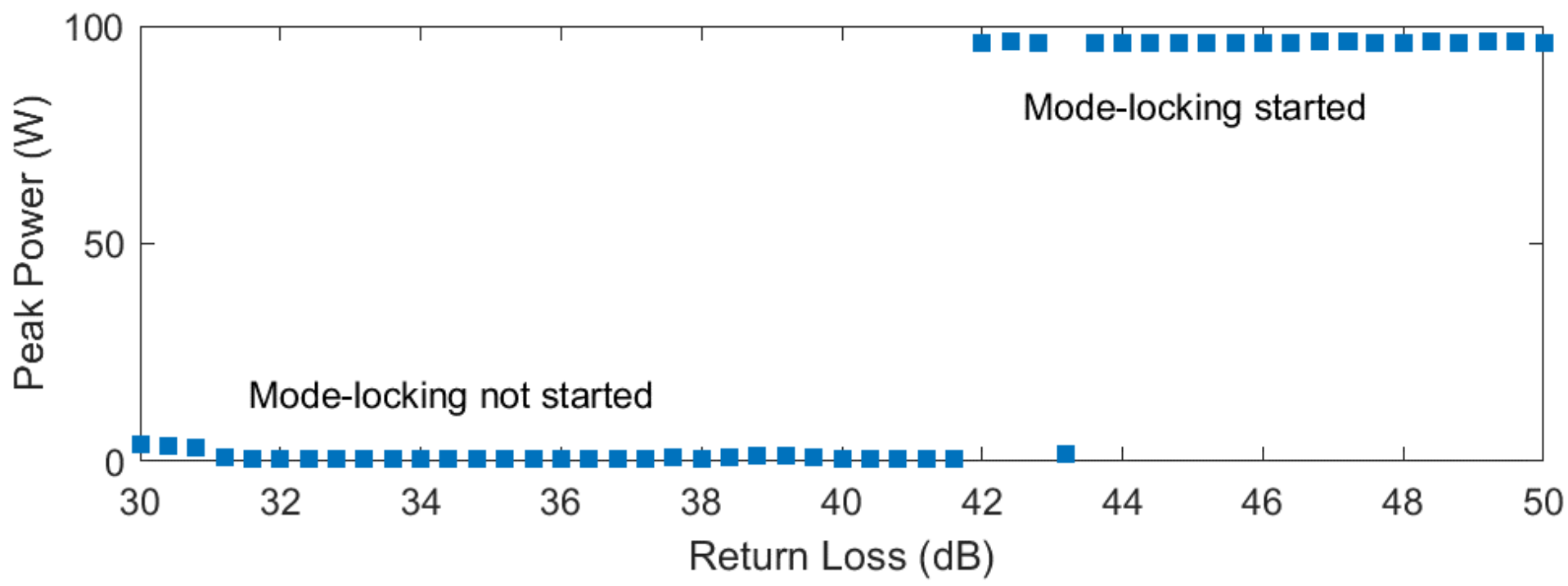

Figure R9 Simulated intracavity peak power after 50 round-trips at different return loss levels. Other parameters are kept same as described in the Supplementary Information S2

Such return-loss levels are realistic with standard photonic and packaging measures, for example using angled edge couplers (created during chip singulation by deep RIE) and matching angled-polished fiber array units (commercially available from Meisu Technologies in China). In comparison, commodity angle-polished fiber connectors (FC/APC or SC/APC) can routinely reach return loss better than 60 dB, providing sufficient margin for starting from noise.

Actions Taken: In the revised manuscript and Supplementary Information, we have shifted focus to the new self-seeding approach by pump current modulation, enabling robust “turn-key” self-starting without external MLL or complex off-chip optics. We have also revised the Supplementary Information S5 to include a discussion indicating that starting from noise will be possible for a facet return loss of more than 42 dB, and this is feasible with angled facet couplers.

Referee #4: “--

I believe that as as written, the reliance on complex off-chip optics for start-up undercuts the "fully integrated, reduced-complexity" claim expected for Nature. Demonstrating robust self-start (or tempering the claims and tightening quantitative comparisons) would lift the work to that bar. If it turns out that self starting of these oscillators is simply not possible (e.g. by the detailed analysis I've requested above), then the impact of this work would be significantly lessened and so this needs to be understood (via demonstrating self-starting oscillator) before publication.”

Answer: We appreciate this concluding assessment, and we agree with the central point: as originally written, the reliance on complex off-chip optics for start-up could undercut a "reduced-complexity" framing. We also appreciate the clear criterion you articulated, namely that demonstrating robust self-start (and/or tempering claims and tightening quantitative comparisons) is what would lift the work to the expected bar.

In the revised manuscript, we have addressed this point directly by demonstrating robust, turn-key initiation of mode-locked operation without any external ultrafast seed source and without any additional off-chip optical components for start-up. Mode-locking is initiated solely by temporary pump-current modulation using a commercially available pump-diode driver, after which the oscillator operates autonomously in the steady-state mode-locked regime. Within a wide pump power range, this procedure has a virtually 100% success rate. Importantly, this purely electrical initiation method *is compatible with our current interface return loss and does not require an extended cavity, external modulator, circulator, or seed shaping and gating.*

In parallel, we have tempered and clarified the manuscript language where appropriate and tightened the quantitative comparisons by explicitly defining the comparison class (PIC-based ultrafast sources) and revising the associated claims accordingly. We hope these changes directly address your concerns about integration and start-up complexity and, crucially, resolve the question of whether practical self-starting operation is achievable for this integrated Mamyshev oscillator architecture.

References:

[R3.1]: RP2350 microcontroller, Raspberry Pi Ltd.: https://www.raspberrypi.com/products/rp2350/

[R3.2]: ESP32-S3 dual-core XTensa MCU, Espressif Systems: https://www.espressif.com/en/products/socs/esp32-s3

[R3.3]: STM32F3 Series of mixed-signal MCUs, STMicroelectronics NV: https://www.st.com/en/microcontrollers-microprocessors/stm32f3-series.html

[R3.4] Samartsev, Igor, Andrey Bordenyuk, and Valentin Gapontsev. "Environmentally stable seed source for high power ultrafast laser." Components and Packaging for Laser Systems III. Vol. 10085. SPIE, 2017.

("The laser is self-starting." is claimed in the abstract, and the exactly same method to start is employed: "For the purpose of pulse initiation we modulate the pump source with a train of pulse of microsecond duration at tens of kilohertz repetition rate.")

[R3.5] Haig, Henry, et al. "Megawatt pulses from an all-fiber and self-starting femtosecond oscillator." Optics Letters 47.4 (2022): 762-765.

("self-starting." is claimed in the title, and the exactly same method to start is employed: "Specifically, we use a simple function generator to create a 70 kHz square wave signal that drives the pump power between 0 and 8 W.")

[R3.6] Wang, Chaoran, Xingliang Li, and Shumin Zhang. "Automated start-up and extinction dynamics of a Mamyshev oscillator based on a temperature-dependent filter." Laser & Photonics Reviews 17.7 (2023): 2201016.

("the narrow filter interval could produce self-starting" is claimed in the conclusion, and heater tuning is performed at each start)

[R3.7] Kruglov, V. I., et al. "Self-similar propagation of high-power parabolic pulses in optical fiber amplifiers." *Optics letters* **25.24** (2000): 1753-1755.

In the following, we provide a point-by-point response to the reviewer's comments. For clarity and ease of reading, the reviewer's original comments are shown in blue, our responses are provided in black, and the corresponding actions taken for the manuscript are highlighted in red.

Referee #1 (Remarks to the Author):

"The revised version represents a substantial improvement over the original submission, both in terms of technical completeness and system-level relevance. Such an extensive and careful revision is really appreciated.

As I noted in my previous review (Reviewer #1), the work is of excellent quality both scientifically and technologically. The demonstration of a self-starting, turn-key Mamyshev oscillator fully integrated on chip (except for the pump sources), delivering nJ-level femtosecond pulses at a repetition rate of 175 MHz, constitutes a clear and significant advance over the current state of the art in integrated ultrafast photonics. The added experimental evidence addressing self-starting operation convincingly resolves one of the major concerns raised in the first review process.

I also acknowledge the effort made to strengthen the breadth of impact by demonstrating an application in THz-TDS. This addition shows that the source can directly enable demanding system-level applications without external amplification, and the reported THz performance (bandwidth and dynamic range) is indeed competitive with established tabletop commercial systems. That said, my original concern regarding the scope of interdisciplinarity is not fully removed. While the THz-TDS experiment is a meaningful demonstration, in my view it showcases the capabilities of the laser source, rather than a fundamentally new result in THz science or in a discipline outside photonics. The core innovation and scientific depth of the manuscript are still strongly in ultrafast optics and integrated photonics. Nevertheless, I recognize that Nature often values the introduction of genuinely new technological capabilities that have the potential to propagate across multiple disciplines. Some of the references provided in the authors' response and published on Nature support this view. In this sense, the revised manuscript now makes a stronger and more credible case that the demonstrated platform can truly act as an enabling technology for applications in spectroscopy, sensing, and materials characterization beyond the photonics community.

Overall, I consider the manuscript scientifically sound, technically mature, and of high impact. While its suitability for Nature remains borderline in terms of interdisciplinarity, an aspect which the Editors are best positioned to evaluate, I believe that the combination of the unprecedented on-chip pulse energy, turn-key operation, and the demonstrated system-level application makes the manuscript worthy of consideration by Nature if the other reviewers share this positive assessment."

Answer: We thank the reviewer for the careful reassessment of the revised manuscript and for recognizing the potential of technological advance enabled by the integrated Mamyshev oscillator, particularly after the addition of the electrically initiated operation and the THz-TDS demonstration. We also appreciate the referee's thoughtful perspective on interdisciplinarity and the role of enabling technologies in Nature.

We agree that our terahertz time-domain spectroscopy (THz-TDS) experiment is not intended to claim a fundamentally new discovery in terahertz science. Rather, our goal is to provide a concrete system-level demonstration showing how the capability of the demonstrated laser source can translate into a demanding application outside integrated photonics.

We recognize that THz-TDS is a mature field with commercial instruments on the market for years, yet broader deployment remains constrained by system size, and cost, which are often determined by the ultrafast laser. By showing that our integrated source can directly drive a compact THz-TDS system

with bandwidth and dynamic range competitive with established tabletop systems, we aim to illustrate how this new class of integrated ultrafast lasers can reduce practical barriers to terahertz technologies. We view this THz-TDS demonstration as one representative example of the broad range of applications that mode-locked ultrafast lasers can enable.

Action taken: We refined the manuscript text in the description of the THz-TDS experiment and in particular, in the concluding sentence "These proof-of-concept demonstrations highlight the potential of integrated-MLL-driven THz-TDS as a path toward portable, cost-effective systems for wide industrial deployment.", in order to explicitly frame the THz-TDS results as an application-level validation of the laser's capability, while trying to avoid overclaiming.

Referee #2 (Remarks to the Author):

"We appreciate the authors for their detailed response to the previous reviewers' comments. The revised manuscript shows improvements compared to the previous version, particularly in the self-starting scheme and the demonstration of the terahertz measurement application. The further simplification of the laser start-up process and the further verification of the laser performance all contribute to a more solid foundation for this work. However, we still have the following questions/comments:"

Answer: We thank the reviewer for recognizing the improvements in the revised manuscript, particularly regarding the self-starting scheme and the system-level THz-TDS demonstration.

1) Regarding "New Tool": This study is another implementation mode for generating ultrafast laser with comparable performance, i.e., on-chip integration, while the integrated photonics platform itself is not novel and has been widely reported. Regarding the implementation principle, the authors acknowledge that "We believe that this integrated Mamyshev oscillator should follow the same theoretical principles as the fiber counterparts," although they add, "However, an integrated Mamyshev oscillator operates in a substantially different parameter regime," without further in-depth research to reveal new physical insights or general design principles.

Answer: We thank the reviewer for this point. We believe that the fundamental theoretical principle of a Mamyshev oscillator, or more widely all single spatial mode fiber-based or waveguide-based mode-locked lasers, is basically the same GNLSE and gain rate equations, which is indeed shared across implementations.

Our claim of a different parameter regime refers to the physical cavity parameters that are inherent to photonic integration (particularly the orders-of-magnitude different dispersion and nonlinearity scales, compared explicitly in Supplementary Note S2.D). At the same time, the integrated device can be meaningfully compared to fiber counterparts through using normalized parameters in the GNLSE (the soliton number N and the normalized gain $\hat{g}$). We hope this can provide more insight to the design principles.

We would also respectfully emphasize that we believe such integrated lasers can have significant prospect in applications, for both the greatly reduced the footprint and the cost, compared to other existing implementation modes. While rare-earth-doped integrated photonics platforms have been reported, achieving a viable path to practical integrated ultrafast laser with them has remained an open challenge for long.

Action taken: We added Supplementary Note S2.D to directly compare the cavity parameters of our integrated laser with representative fiber Mamyshev oscillators, and further examine the design principle using normalized parameters in the GNLSE.

2) Regarding "New Capability": The direct cavity-output pulse energy does represent a significant improve (2 order) over prior integrated sources, while this performance advantage may not be unique.

Other integrated source schemes could potentially achieve similar or superior performance for application through subsequent on-chip amplification, which may not introduce additional complexity.

Answer: We thank the reviewer and agree that "seed + amplifier" architectures are a complementary and important approach (as implied in the comments by Reviewer #4 in the last round). Our point is not that amplification is impossible, but that reaching nanojoule-class pulse energies on-chip via post-amplification is not presently straightforward given realistic constraints on seed properties and available integrated gain.

In particular, on-chip semiconductor and rare-earth-doped amplifiers typically provide limited small-signal gain (<30 dB) and limited average output power (<300 mW when using diode pumping). To reach nanojoule energies at the output under these constraints, one generally requires seed pulses with sufficiently high pulse energy typically at least tens of pJ at repetition rates <300 MHz, to have sufficient gain after saturated by the average output power. However, from the comparison in Supplementary Note S1, none of the existing sources to our knowledge can achieve this. Most reported integrated mode-locked sources operate at much higher repetition rates ($\mathcal{O}$(1 GHz) to $\mathcal{O}$(100 GHz)), which makes direct scaling to nJ via amplification impossible without additional system elements.

One feasible approach is using a pulse picker to reduce the repetition rate and use another stage of preamplification, which we believe is will inevitably introduce additional complexity (although is, in principle, within the current capability of photonic integration). In addition, we note that as described in Supplementary Note S1, our result shows a significantly superior coherence compared to all existing semiconductor based MLLs (and thus any amplified pulses seeded by them). The coherence degradation in the amplification process is also subject to further investigation.

Action taken: In the revised manuscript, we have added a footnote in Supplementary Note S11 to explicitly state why achieving comparable performance via on-chip amplification is nontrivial with today's integrated seed sources and amplifier constraints:

"Existing integrated ultrafast sources typically generate pulses at repetition rates of O(1 GHz) to O(100 GHz) and have relatively low pulse energies (Section S1). Pulse picking and an additional pre-amplification stage would likely be required to achieve a sufficiently low repetition rate to produce a pulse train comparable to the input used in this simulation."

3) Regarding "New Application": The authors list a series of applications for mode-locked ultrafast lasers (such as ranging, imaging, and spectroscopy), and add a new application on terahertz spectroscopy using their laser source, demonstrating its interdisciplinary practicality. The terahertz spectroscopy experiment remains, in essence, a demonstration of a new source's functionality, and does not move beyond the established paradigm. It does not demonstrate a unique, transformative impact on new applications.

Answer: We thank the reviewer and agree with the characterization that our THz-TDS experiment is not intended as a discovery in terahertz science. The purpose is to provide an application-level validation that the demonstrated laser is not only a laboratory ultrafast source, but can directly drive a complete measurement system relevant outside integrated photonics.

We respectfully disagree, however, that this lacks a unique or potentially transformative impact. *THz-TDS is a relatively mature field with commercial systems with state-of-the-art performance available. While in practice, the footprint and cost of existing solid-state or fiber-based ultrafast lasers is a cardinal issue for widespread application of THz-TDS technology, and we believe our work have proposed a direct solution to that.* In that sense, the novelty is not the THz measurement demonstration itself, but the credible route toward portable and low-cost THz-TDS systems.

Action taken: We revised the manuscript to more explicitly frame the THz-TDS results as a system-level validation of the laser's capability and to avoid overclaiming. In particular, we refined the concluding sentence to: "These proof-of-concept demonstrations highlight the potential of integrated-MLL-driven THz-TDS as a path toward portable, cost-effective systems for wide industrial deployment."

Other questions:

1. We thank the authors for their further explanation of the performance design, including parameters such as cavity length, dispersion, filters, and output coupling ratio, etc. In the rebuttal letter, the authors emphasize that their research focuses on high-energy integrated on-chip laser sources, and claimed that the integrated Mamyshev oscillator operates in a "new parameter regime" compare to the fiber Mamyshev oscillator. In this regard, for the first on-chip integrated Mamyshev study, I suggest that the authors provide more general design analysis or simulation guidelines which would be crucial for advancing this research direction, rather than choosing one method for demonstration due to limitations in experimental conditions.

Answer: We thank the reviewer for this constructive request and fully agree. In response, we expanded the Supplementary Note S2.D to include a more general design framework for translating the integrated Mamyshev oscillator to different waveguide platforms that have different properties and constraints. We have included an explicit comparison between the parameters in a typical fiber-based Mamyshev oscillator and two integrated laser designs.

Specifically, we present guidelines for design and provide discussions based on a normalized (unitless) form of the GNLSE that highlights the physics in the pulse propagation process. We additionally include a concrete example of a lower-power design on a different erbium-doped silicon nitride platform with reduced GVD to illustrate the design process.

Action taken: We added Supplementary Note S2.D detailing the design and simulation guidelines, directly comparing the physical and normalized parameters, as well as a concrete example aiming for a new lower powered design by using a 2 um x 600 nm erbium doped silicon nitride platform to reduce waveguide GVD.

In addition, we have provided our simulation code available in the supplementary Zenodo repository so readers can readily explore alternative platforms and constraints.

2. In S1, "selection of commercially available low-power table-top mode-locked lasers (MLLs) is also listed for reference". Comparing only to low-power commercial table-top lasers is of limited value. We suggest a direct and honest comparison with the best-reported performance of fiber mode-locked lasers (including fiber Mamyshev oscillators) on key metrics like pulse energy and duration to objectively contextualize the advantages and limitations of this work. A similar issue exists in S14 for the terahertz TDS system performance. Comparisons with the best-reported systems should be included.

Answer: We thank the reviewer for this comment and agree that a more complete comparison with fiber-based lasers can be useful, while noting that the modern pulsed lasers can scale extremely well in terms of energy and power (up to facility-scale laser systems), if the pump power and footprint is unconstrained. We have added more representative works to the comparisons in S1 that we believe can help the readers, particularly fiber-based Mamyshev oscillators and fiber-based solitonic lasers in S1.

For THz-TDS, we extended the comparison to include additional commercial systems and laboratory demonstrations aiming to better reflect the state-of-the-art. We also note that some laboratory demonstrations are focused on improved photoconductive antennas (e.g. Ref. [88]) or electro-optical crystals; our laser-driven system may plausibly benefit similarly if such emitters and detectors are adopted.

Action taken: We expanded Table S1 to include six representative entries for typical fiber-based lasers. We also expanded Table S4 by adding eight additional commercial systems and eleven laboratory demonstrations to better cover reported THz-TDS performance.

3. At line 213, please explicitly state the method for calculating thickness from the time delay. What's the minimum measurable thickness of the system?

Answer: We thank the reviewer for the request and have clarified the thickness extraction method and estimated the thickness resolution in Supplementary Note S3.H. We also note that given the reasonably high dynamic range of the measurement, deconvolution can be an effective approach to further improve the thickness resolution, although not being pursued here.

Action taken: We have added explicit statement on the thickness calculation in the Supplementary Note S3.H and estimated the minimum measurable thickness/resolution of the system, as following:

"To demonstrate thickness metrology, we insert a double-side-polished, high-resistivity float-zone silicon wafer into the THz beam path. The time delay between the main pulse and the reflected pulse is Δt_{rt} = 11.93 ps. This delay corresponds to the round-trip propagation time of the terahertz pulse inside the silicon wafer. Accordingly, the single-pass delay is $\Delta t_{sp} = \Delta t_{rt}/2$ = 5.965 ps. Using the refractive index of n_{Si} = 3.4173 [62], the wafer thickness d is obtained from $d = c\Delta t_{rt}/(2n_{Si})$ = 523.3 μm, where c is the speed of light. The minimum measurable thickness is set by the minimum resolvable separation between the main pulse and its internal-reflection replica. In our THz-TDS system, the 3 dB width of the main time-domain peak is $\Delta t_{rt,3dB}$ = 0.35 ps, and we take this value as an estimate of the minimum resolvable delay without using deconvolution. The corresponding thickness resolution is therefore $d_{min}=c\Delta t_{rt,3dB}/(2n_{Si})$ = 15.4 μm."

"4. Formatting suggestions:

1) In the SI, the page number "S1" and the section label "S1" are confusing. Please adjust.

2) Please ensure all SI sections are cited in the main text and consider reordering them to follow the citation sequence.

3) Please unify the reference formatting between the main text and the SI."

Answer: We thank the reviewer for these helpful formatting suggestions and have revised the Supplementary Notes accordingly.

Action taken:

1) We have changed all page numbers in the Supplementary Notes to SPxx to avoid confusion with the section labels.

2) We reordered the Supplementary Notes to better follow the main-text citation sequence, moved appropriate content to Extended Data Figures, and ensured each Supplementary section is cited.

3) We revised the reference formatting in the Supplementary Notes to more closely match the *Nature* format used in the main text.

Referee #3 (Remarks to the Author):

"I co-reviewed this manuscript with one of the reviewers who provided the listed reports."

Referee #4 (Remarks to the Author):

"The authors have carried out a substantial revision that addresses my principal concerns. In particular:

The most critical issue, the reliance on an external ML laser for initiation has been resolved by demonstrating a pump-current-modulation self-seeding approach that eliminates all off-chip optical components for start-up. The supporting data seem to be fairly convincing. The misleading full integrated language has been appropriately corrected.

The addition of the THz TDS demonstration provides a concrete application that strengthens the case.

The remaining technical characterizations have all been provided and are satisfactory."

Answer: We thank the reviewer for the careful evaluation and for acknowledging the revisions, including the self-seeding results and the THz-TDS demonstration. We are encouraged by your recognition of the technical rigor, and we are glad that these additions resolve your major concern.

"I have two minor remaining comments:

1. The authors use "self-starting" and "self-seeded", is this the right terminology for an electrically initiated process?"

Answer: We thank the reviewer for the helpful terminology suggestion. We agree that this can indeed be interpreted differently. While similar terms are sometimes used in the mode-locked laser literature to describe initiation by either pump intensity modulation, filter gap tuning, or actuated polarization rotation, we agree that, in our case, "self-starting" could be read as implying spontaneous emergence from noise without any deliberate initiation.

To describe the start-up process in a more precise way and to avoid overclaiming, we now consistently use the term "self-seeded" throughout the manuscript. This emphasizes that no external optical seed is required, while is not implying that stable pulsing emerges directly from noise.

Action taken: We replaced all instances of "self-starting" with "self-seeded" in the main text and Supplementary Note, and adjusted the phrasing accordingly.

"2. The headline compressed pulse duration of 147 fs is achieved using ~10 m of external single-mode fiber. The authors discuss on-chip compression via chirped Bragg gratings as feasible but do not demonstrate it. I would appreciate a brief explicit statement in the main text noting this... (if i understood it correctly)"

Answer: We thank the reviewer for pointing out that this should be stated explicitly in the main text. The 147 fs pulse duration reported in the manuscript is obtained after external dispersion compensation using approximately 10 m of single-mode fiber. While dispersion compensation can in principle be implemented on the Si3N4 platform (for example via chirped Bragg gratings), it is not implemented in the present device and remains a direction for future work. In addition, we note that redesigning the cavity/waveguides to reduce the net GVD is a complementary path that can simplify the compression requirement.

Action taken: We added an explicit statement in the main text: "Such dispersion compensation can be implemented on the Si_3N_4 platform using chirped Bragg gratings, although it is not implemented in the present device."

“These are minor points that do not affect the core conclusions. I am now fairly supportive of publication in Nature.”

Answer: We sincerely appreciate the reviewer's supportive assessment.
